# Mixed Lithium Amide–Lithium Halide Compounds: Unusual Halide-Deficient Amido Metal Anionic Crowns**

**DOI:** 10.1002/anie.201102023

**Published:** 2011-07-19

**Authors:** Alan R Kennedy, Robert E Mulvey, Charles T O'Hara, Gemma M Robertson, Stuart D Robertson

**Affiliations:** WestCHEM, Department of Pure and Applied Chemistry, University of Strathclyde295 Cathedral Street, Glasgow (UK); * WestCHEM, Department of Pure and Applied Chemistry, University of Strathclyde295 Cathedral Street, Glasgow (UK)

**Keywords:** amides, halides, inverse crowns, lithium, salt effect

Alkali metal halide salts can dramatically influence the reactivity/selectivity of organic transformations in either beneficial or detrimental ways.^1^ In many circumstances, the metal halide salt formed in situ in a metathesis reaction is dismissed as an innocent by-product. Recently, more cases have come to light where lithium halides affect organometallic reactions in a non-innocent, often dominant way. Knochel et al. has exploited this effect by adding stoichiometric amounts of LiCl to conventional Grignard or Hauser reagents to induce an enhanced reactivity with respect to that of monometallic magnesium reagents.^2^ Collum et al. presented the surprising and profound role that LiCl plays in a series of deprotonation^3^ and addition reactions,^4^ establishing that LiCl catalysis is detectable with miniscule quantities of LiCl, and that “striking accelerations” (70 fold) are elicited by less than 1.0 mol % LiCl for 1,4-addition reactions of lithium diisopropylamide to unsaturated esters.^4^ Despite this, firm structural evidence of the crucial halide-incorporated species that may be involved in these reactions is rare.1h, 5 In one example, we recently synthesized and characterized the magnesiate [(thf)_2_Li(μ-Cl)_2_Mg(TMP)(thf)] and found that it functions identically to Knochel’s in situ Grignard system (TMP=2,2,6,6-tetramethylpiperidide).^6^

Herein we start to deconvolute the complex chemistry at work when synthetically important lithium amides come into contact with a halide source. Pertinent to this work, we previously discovered that a hexane solution of NaHMDS and (−)-sparteine can react with adventitious water to yield the hydroxy-incorporated sodium sodiate, [{(−)-sparteine}Na(μ-HMDS)Na{(−)-sparteine}]^+^[Na_4_(μ-HMDS)_4_(OH)]^−^ (**1**; Scheme 1), where HMDS is 1,1,1,3,3,3-hexamethyldisilazide.^7^ Given that this diamine–NaHMDS system has formally captured monomeric NaOH, we envisaged that a similar LiHMDS system could capture sub-stoichiometric quantities of other salts, and particularly the Lewis amphoteric metal halides, which appear far more important than metal hydroxides for metal salt-enhanced reactions.

**Scheme 1:**
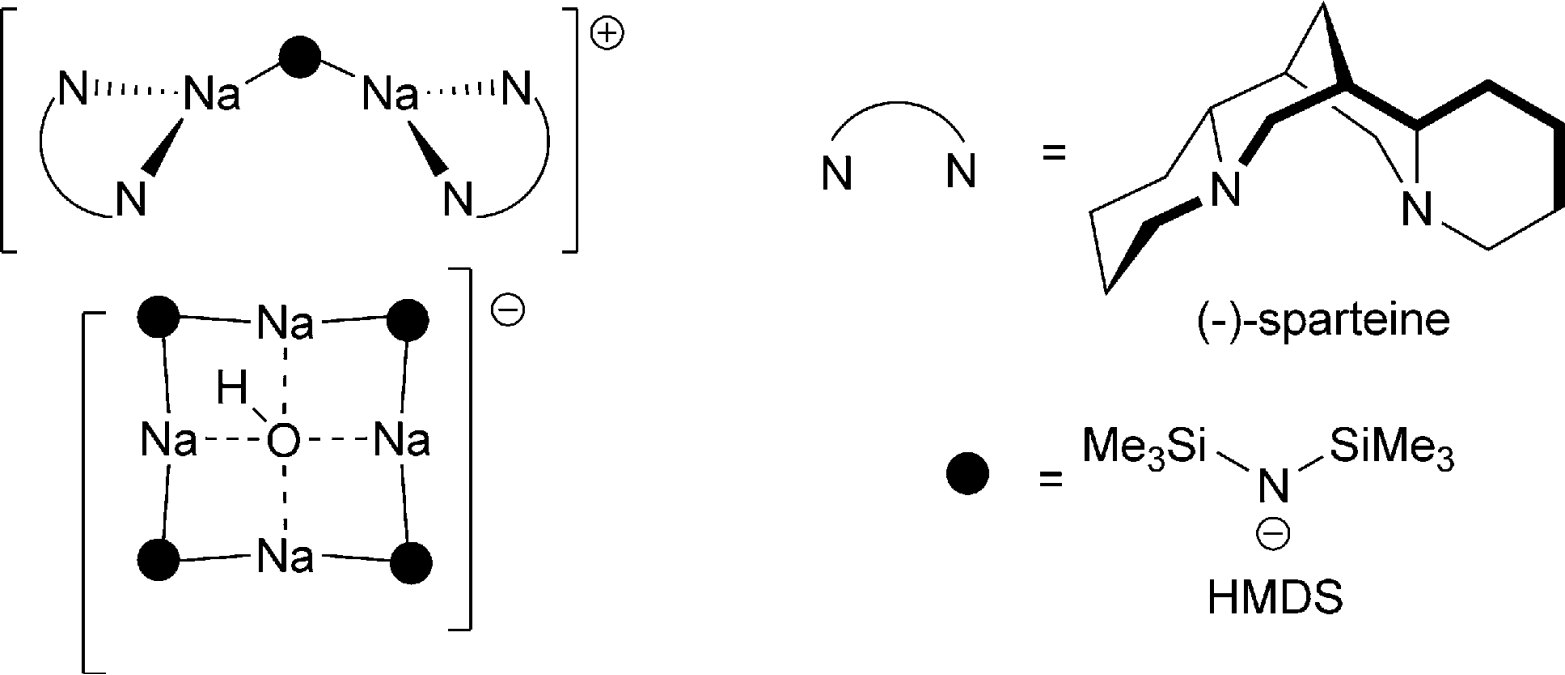
Molecular structure of the sodium sodiate [{(−)-sparteine}Na(μ-HMDS)Na{(−)-sparteine}]^+^[Na_4_(μ-HMDS)_4_(OH)]^−^ **1**.

We have investigated several approaches in reaching this goal. Firstly, by attempting direct combination (co-complexation) of LiHMDS and a diamine with sub-stoichiometric LiX (where X is Cl, Br, or I); secondly, by combining *n*BuLi with NH_4_X (ammonium salt route^8^) and then introducing superstoichiometric LiHMDS in the presence of a diamine; and, thirdly, by treating NEt_4_X (organoammonium salt route) in a similar manner to the previous approach (Scheme 2; Supporting Information, Scheme S1). Gratifyingly, these reactions provide us with an enhanced structural insight into the coordination of LiX with LiHMDS. For brevity, only the co-complexation route (for **2**–**4**) and ammonium salt route (for **5**) are discussed herein, although full details of the other routes are given in the Supporting Information.

**Scheme 2:**
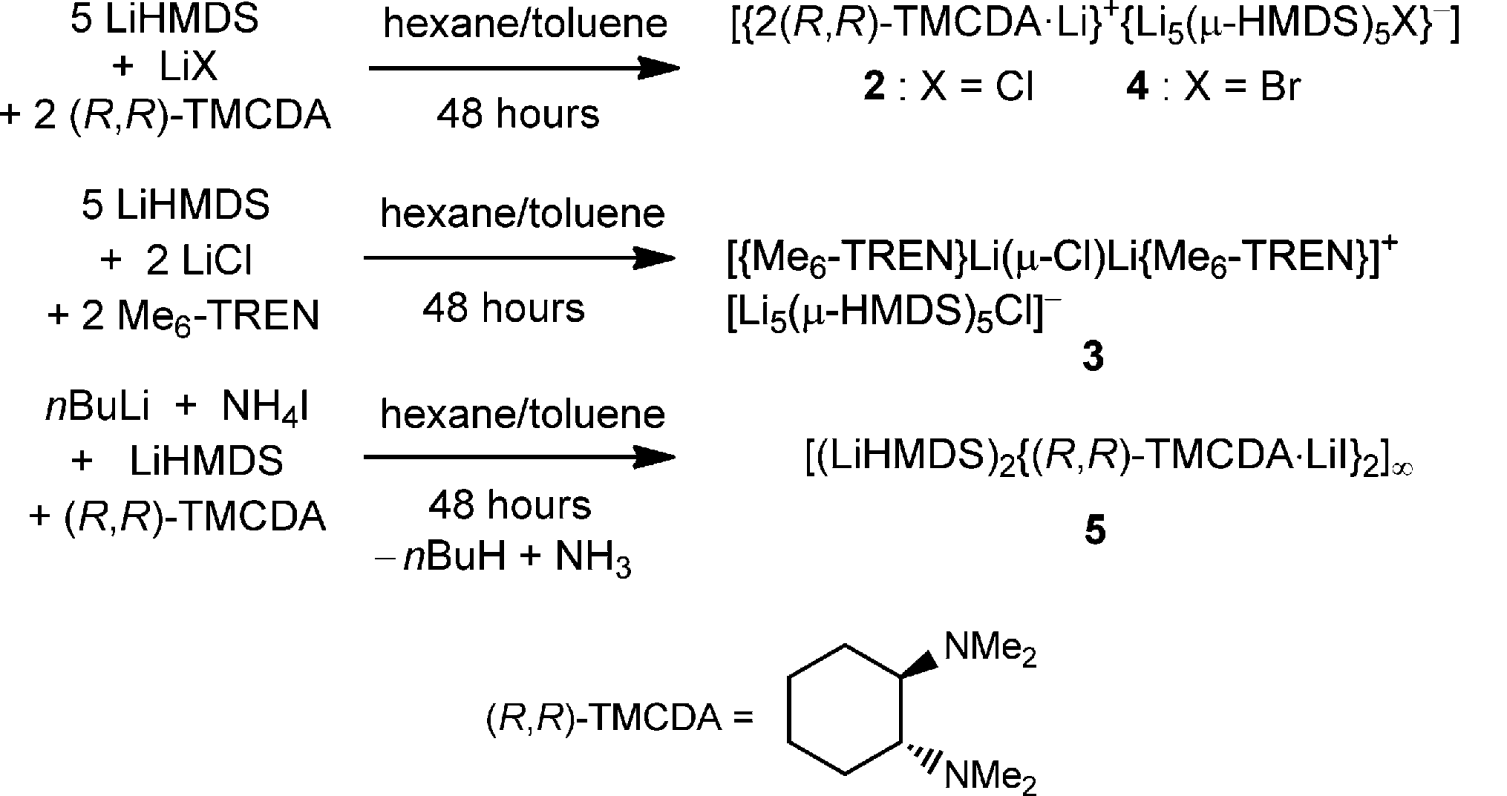
The synthesis of compounds **2**–**5**.

Our research focused on growing crystals suitable for X-ray analysis that could provide insight into species potentially present in lithium amide–halide-containing solutions used in organic transformations. The first reaction combined LiHMDS, LiCl, and the chiral diamine *N*,*N*,*N*′,*N*′-(1*R*,2*R*)-tetramethylcyclohexane-1,2-diamine ((*R*,*R*)-TMCDA), initially in a 1:1:1 stoichiometric ratio in hexane solution. A small crop of X-ray-quality crystalline material was afforded from this mixture after 24 h. X-ray crystallographic analysis^9^ revealed that despite the equimolar ratio of LiHMDS and LiCl in the reaction, crystallization of the lithium lithiate [{2(*R*,*R*)-TMCDA}Li]^+^[Li_5_(μ-HMDS)_5_Cl]^−^ **2** (Figure 1) resulted.

**Figure 1:**
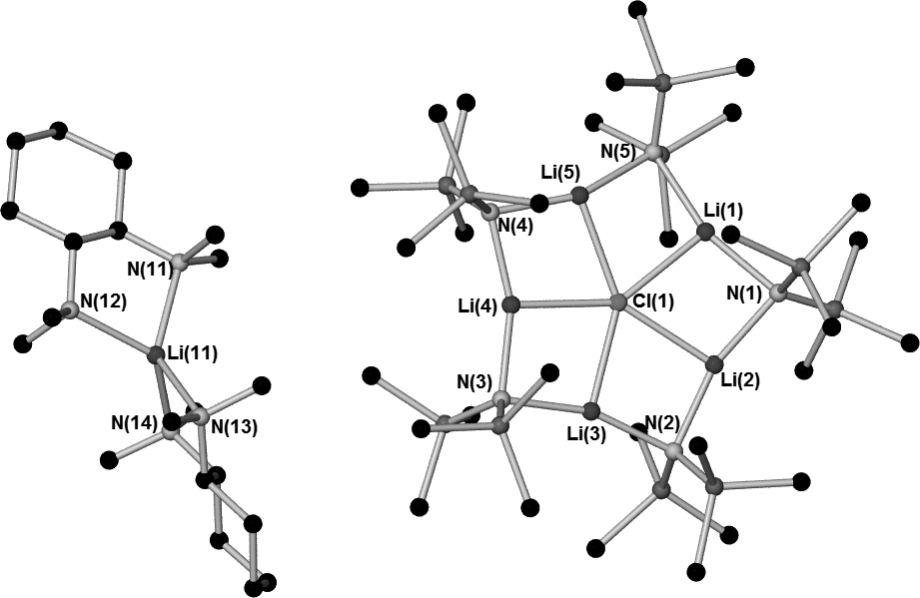
Molecular structure of [{2(*R*,*R*)-TMCDA}Li]^+^[Li_5_(μ-HMDS)_5_Cl]^−^ (**2**). Left: cation, right: anion.

Complex **2** can also be prepared by utilizing a rational stoichiometry, that is, a ratio of LiHMDS/LiCl/(*R*,*R*)-TMCDA of 5:1:2. In general, yields of crystalline product for all of the complexes discussed are low owing to their high solubility in hexane.^10^ Complex **2** exists as a solvent-separated ion pair with the cation comprising a distorted tetrahedral lithium atom (range of angles 83.1(3)–130.9(3)°; mean angle 110.6°) bound to two bidentate (*R*,*R*)-TMCDA ligands. The complex anion is a ten-membered Li_5_N_5_ ring of alternating metal and nitrogen atoms that hosts a chloride anion. This unexpected entity must be considered in the context of the well-developed structural chemistry of LiHMDS species. Donor-free LiHMDS exists as a trimer in the solid state,^11^ whilst solution studies by Collum and Lucht reveal that an equilibrium exists between a dimeric and a tetrameric species.^12^ To the best of our knowledge, a discrete ten-atom Li_5_N_5_ ring (or indeed of any lithium anion combination) has not been reported to date. Indeed, pentanuclear Li_5_ species of any compound class or architecture are exceptionally rare.^13^

Complex **2** can be considered as belonging to a new class of complexes called metal anionic crowns (MAC). Closely related to known inverse crown complexes,^14^ this type of complex has two important differences. Firstly, the MAC complexes are monometallic (specifically alkali metals to date), and secondly, they are ionic, solvent-separated ion pairs, unlike inverse crowns, which are heterobimetallic neutral entities. Therefore, **2** boasts a perfect inverse topological relationship to conventional crown ether complexes, which have the general formula [{crown}M]^+^[anion]^−^. The mean Li–Cl distance in **2** is 2.437 Å, which is longer than those in other neutral mixed amide–chloride lithium complexes (range of mean Li–Cl distances 2.342–2.361 Å),1h, 5a which is most likely due to the higher μ_5_-coordination of the clorine atom in **2**. The star-shaped ring of the anion of **2** is puckered at the N(1) atom (Figure 2). The remaining nine annular atoms are essentially planar (N(1) is situated 0.895(5) Å out of this plane). These data suggest that the cavity formed by a planar Li_5_N_5_ ring would be too large to adequately sequester the chloride anion. This thought prompted investigation of bromide capture (see below).

**Figure 2:**
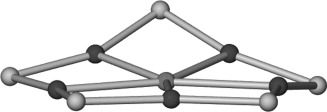
Alternative view of the anion of **2** showing the puckered nature of the Li_5_N_5_ ring.

When the potentially tetradentate donor tris[2-(dimethylamino)ethyl]amine (Me_6_-TREN) is utilized instead of (*R*,*R*)-TMCDA, the MAC that formed, [{Me_6_-TREN}Li(μ-Cl)Li{Me_6_-TREN}]^+^[Li_5_(μ-HMDS)_5_Cl]^−^ (**3**), has in terms of composition an identical anion to **2** but a different cation (Figure 3).^15^ Now an additional LiCl unit has been captured, thus bearing parallels with **1** where an additional monomeric NaHMDS unit has been trapped. All four nitrogen donor atoms of the Me_6_-TREN ligand coordinate to one lithium center, whose coordination sphere is completed by a chlorine atom locked in a linear Li–Cl–Li chain.^16^ MAC formation seems to be largely insensitive towards the sequestering amine, as (*R*,*R*)-TMCDA, Me_6_-TREN, and TMEDA (*N*,*N*,*N*′,*N*′-tetramethylethylenediamine)^17^ all give rise to this unusual anion.

**Figure 3:**
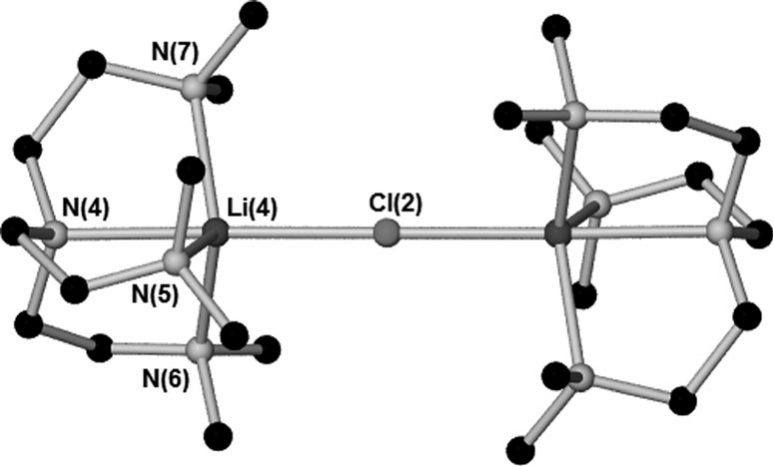
Molecular structure of the cation of **3**.

Turning to bromide capture, the method detailed above proved to be general, and the bromide-containing MAC [{2(*R*,*R*)-TMCDA}Li]^+^[Li_5_(μ-HMDS)_5_Br]^−^ (**4**) was successfully prepared. Unfortunately, the X-ray data obtained were of poor quality, thus precluding any discussion of structural parameters; however, atom connectivity was unambiguous. The most striking features of the anion of **4** (Figure 4) with respect to **2** is that the entire Li_5_N_5_ ring is planar, and the bromine atom, rather than occupying a position in the plane of the ring, is situated 0.4 Å above or below the plane (as it is disordered over both sites). Again the ultimate synthesis of **4** appears to be insensitive towards the stoichiometry of the reactants, and the yield of the complex can be improved by combining the reactants in the correct stoichiometry.

**Figure 4:**
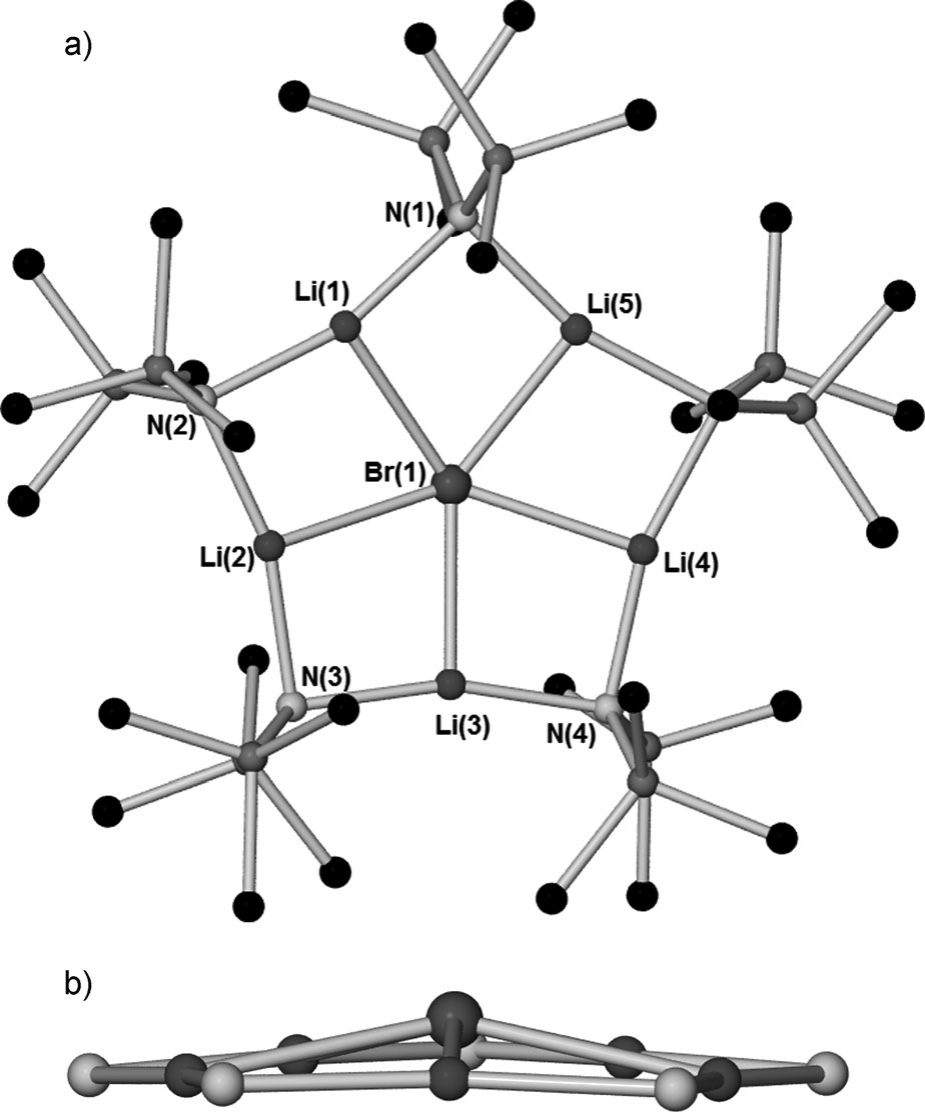
Molecular structure of [{Li_5_(μ-HMDS)_5_Br}^−^] (the anion of **4**) and an alternative view showing the relatively planar nature of the Li_5_N_5_ ring.

To try and expand the ring size of the anionic host, LiI was combined with 1 to 7 molar equivalents of LiHMDS in an effort to prepare an iodine-containing MAC. However a MAC complex was not isolated even when a vast deficit of LiI was utilized with respect to LiHMDS. Instead, crystals of polymeric species [{LiHMDS}_2_{(*R*,*R*)-TMCDA⋅LiI}_2_]_∞_ (**5**), comprising alternate (LiHMDS)_2_ and (LiI)_2_ units (that is, a 1:1 HMDS/I complex), were isolated (Figure 5). The polymer propagates through intermolecular Li⋅⋅⋅I contacts (mean distance 2.799 Å), rendering the iodide anions three-coordinate. In essence, the (LiI)_2_ units act as pseudo donors towards the LiHMDS dimers akin to conventional donors, such as THF.^18^ Lithium atoms bound to two N_HMDS_ atoms are distorted trigonal-planar, while those attached to two iodine atoms are distorted tetrahedral owing to additional coordination by bidentate (*R*,*R*)-TMCDA ligands.

**Figure 5:**
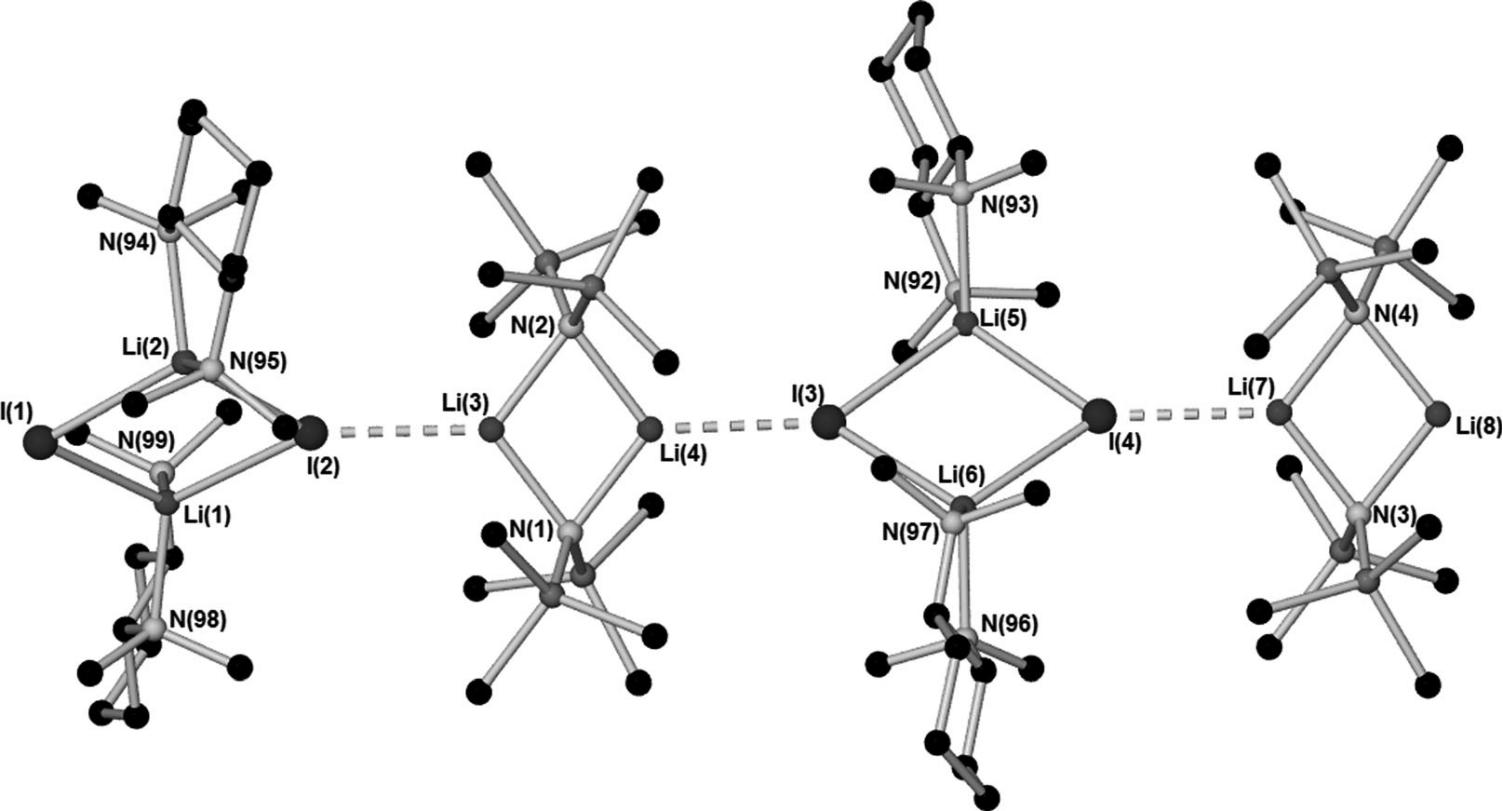
Section of polymeric structure of [{LiHMDS}_2_{(*R*,*R*)-TMCDA⋅LiI}_2_]_∞_ (**5**).

^1^H, ^13^C, and ^7^ Li NMR spectroscopic studies were conducted on C_6_D_6_ solutions of **2**–**5**. In all cases, ^7^Li spectra revealed two different lithium environments, in keeping with the solid state structures, and the expected donor amine to HMDS ratio in the respective ^1^H NMR spectra was observed. An interesting feature was observed in the ^1^H NMR spectrum of **4**. Two extremely broad Si–C*H*_3_ resonances were observed in arene solution, which is most likely due to conformational fluctuations of the Li_5_N_5_ ring and the fact that the bromide ion sits out of the ring plane, giving rise to inequivalency in the tetrahedrally-orientated TMS groups (Figure 4). In a [D_8_]THF solution of **4**, a single, much sharper resonance was observed.

To conclude, the surprising discoveries herein have stripped back another layer of the structural complexity covering simple lithium amide/lithium halide systems. With these new MAC complexes in hand, future research will explore their chemistry and any potential effects they might impart in conventional lithium amide deprotonation reactions.

## Experimental Section

Full general experimental details, synthetic details, and analytical data are given in the Supporting Information. The syntheses detailed below are optimized for crystal growth rather than yield of isolated product.

General synthesis of **2**–**4**: A flame-dried Schlenk tube was charged with lithium bis(trimethylsilyl)amide (0.837 g, 5 mmol) in a glovebox, after which dried hexanes (7.5 mL) was added and the mixture allowed to stir for 30 min. Lithium halide (1 mmol for **2** and **4**; 2 mmol for **3**) was then introduced and the mixture allowed to stir for a further 30 min. Two molar equivalents of diamine (*R*,*R*)-TMCDA or Me_6_-TREN were added and a color change from colorless to pale yellow was observed. This solution was gently heated and allowed to stir vigorously at ambient temperature for 72 h. For **3** and **4**, toluene was introduced to aid dissolution and crystallization. The resultant slightly cloudy solution was heated and filtered through Celite/glass wool and the ensuing clear solution was immediately placed in a freezer operating at −28 °C. After 48 h, a small crop of X-ray quality colorless crystals were deposited (**2**: 0.10 g, 8 %; **3**: 0.13 g, 9 %; **4**: 0.14 g, 11 %). Higher yields of non-crystalline product were obtained using methods outlined in the Supporting Information.

**5**: *n*-Butyllithium (0.63 mL of 1.6 m solution in hexanes, 1 mmol) was placed in a Schlenk tube. The hexane solvent was removed in vacuo and replaced with 5 mL of dried toluene. Two molar equivalents of (*R*,*R*)-TMCDA (0.38 mL, 2 mmol) were added to give a bright fluorescent red/orange solution which was allowed to stir for 30 min. Upon addition of one molar equivalent of ammonium iodide (0.145 g, 1 mmol), this color slowly dissipated with slight heating and stirring to yield a pale pink solution. The mixture was heated to reflux for one hour and the clear pale yellow solution was allowed to stir whilst cooling for 30 min. One molar equivalent of lithium bis(trimethylsilyl)amide (0.167 g, 1 mmol) was then introduced and the resultant slightly cloudy pale yellow solution heated slightly and allowed to stir at ambient temperature for 48 h. An additional 2.5 mL of dried toluene was then introduced, along with heating, and the solution immediately placed in a hot-water-filled Dewar flask. After 24 h, a crop of X-ray quality colorless crystals of **5** precipitated from the solution (0.40 g, 85 %).

## Supplementary material

Detailed facts of importance to specialist readers are published as ”Supporting Information”. Such documents are peer-reviewed, but not copy-edited or typeset. They are made available as submitted by the authors.

## References

[1a] Ashby EC, Noding SA (1979). J. Org. Chem.

[1b] Yamamoto Y, Yamada J-I (1988). J. Chem. Soc. Chem. Commun.

[1c] Murakata M, Nakajima M, Koga K (1990). J. Chem. Soc. Chem. Commun.

[1d] Hall PL, Gilchrist JH, Collum DB (1991). J. Am. Chem. Soc.

[1e] Hasegawa Y, Kawasaki H, Koga K (1993). Tetrahedron Lett.

[1f] Juaristi E, Beck AK, Hansen J, Matt T, Mukhopadhyay T, Simson M, Seebach D (1993). Synthesis.

[1g] Seebach D, Beck AK, Studer A (1995). Mod. Synth. Methods.

[1h] Henderson KW, Dorigo AE, Liu Q-Y, Williard PG, Schleyer PvonR, Bernstein PR (1996). J. Am. Chem. Soc.

[2a] Krasovskiy A, Knochel P (2004). Angew. Chem.

[b19] (2004). Angew. Chem. Int. Ed.

[2b] Krasovskiy A, Straub BF, Knochel P (2006). Angew. Chem.

[b20] (2006). Angew. Chem. Int. Ed.

[3] Gupta L, Hoepker AC, Singh KJ, Collum DB (2009). J. Org. Chem.

[4] Ma Y, Hoepker AC, Gupta L, Faggin MF, Collum DB (2010). J. Am. Chem. Soc.

[5a] Mair FS, Clegg W, O’Neil PA (1993). J. Am. Chem. Soc.

[5b] Stern D, Finkelmeier N, Stalke D (2011). Chem. Commun.

[6] García-Álvarez P, Graham DV, Hevia E, Kennedy AR, Klett J, Mulvey RE, O’Hara CT, Weatherstone S (2008). Angew. Chem.

[b21] (2008). Angew. Chem. Int. Ed.

[7] Clark NM, García-Álvarez P, Kennedy AR, O’Hara CT, Robertson GM (2009). Chem. Commun.

[8] Barr D, Snaith R, Wright DS, Mulvey RE, Wade K (1987). J. Am. Chem. Soc.

[9] http://www.ccdc.cam.ac.uk/data_request/cif.

[11] Mootz D, Zinnius A, Böttcher B (1969). Angew. Chem.

[b22] (1969). Angew. Chem. Int. Ed. Engl.

[12] Lucht BL, Collum DB (1999). Acc. Chem. Res.

[13a] De Vries TS, Goswami A, Liou LR, Gruver JM, Jayne E, Collum DB (2009). J. Am. Chem. Soc.

[13b] Barr D, Clegg W, Mulvey RE, Snaith R (1984). J. Chem. Soc. Chem. Commun.

[13c] Clegg W, Liddle ST, Mulvey RE, Robertson A (2000). Chem. Commun.

[14a] Mulvey RE (2009). Acc. Chem. Res.

[14b] Mulvey RE, Mongin F, Uchiyama M, Kondo Y (2007). Angew. Chem.

[b23] (2007). Angew. Chem. Int. Ed.

[14c] Wu JC, Pan XB, Tang N, Lin CC (2010). Inorg. Chem.

[16a] Buttrus NH, Eaborn C, Hitchcock PB, Smith JD, Stamper JG, Sullivan AC (1986). J. Chem. Soc. Chem. Commun.

[16b] Bazhenova TA, Kulikov AV, Shestakov AF, Shilov AE, Antipin MY, Lysenko KA, Struchkov YT, Makhaev VD (1995). J. Am. Chem. Soc.

[18a] Mack H, Frenzen G, Bendikov M, Eisen MS (1997). J. Organomet. Chem.

[18b] Engelhardt LM, Jolly BS, Junk PC, Raston CL, Skelton BW (1986). Aust. J. Chem.

