# Supplementary material for: Mixed Lithium Amide–Lithium Halide Compounds: Unusual Halide-Deficient Amido Metal Anionic Crowns
Source: Angew Chem Int Ed Engl. 2011 Jul 19;50(36):8375–8. doi: 10.1002/anie.201102023 (PMC3625747; doi:10.1002/anie.201102023)
Supplement: Supplementary file 1 [file anie0050-8375-SD1.pdf]

Angewandte Chemie
Eine Zeitschrift der Gesellschaft Deutscher Chemiker

Supporting Information

© Wiley-VCH 2011

69451 Weinheim, Germany

Mixed Lithium Amide–Lithium Halide Compounds: Unusual Halide-Deficient Amido Metal Anionic Crowns**

Alan R. Kennedy, Robert E. Mulvey, Charles T. O'Hara,* Gemma M. Robertson, and Stuart D. Robertson*

anie_201102023_sm_miscellaneous_information.pdf

Full experimental details

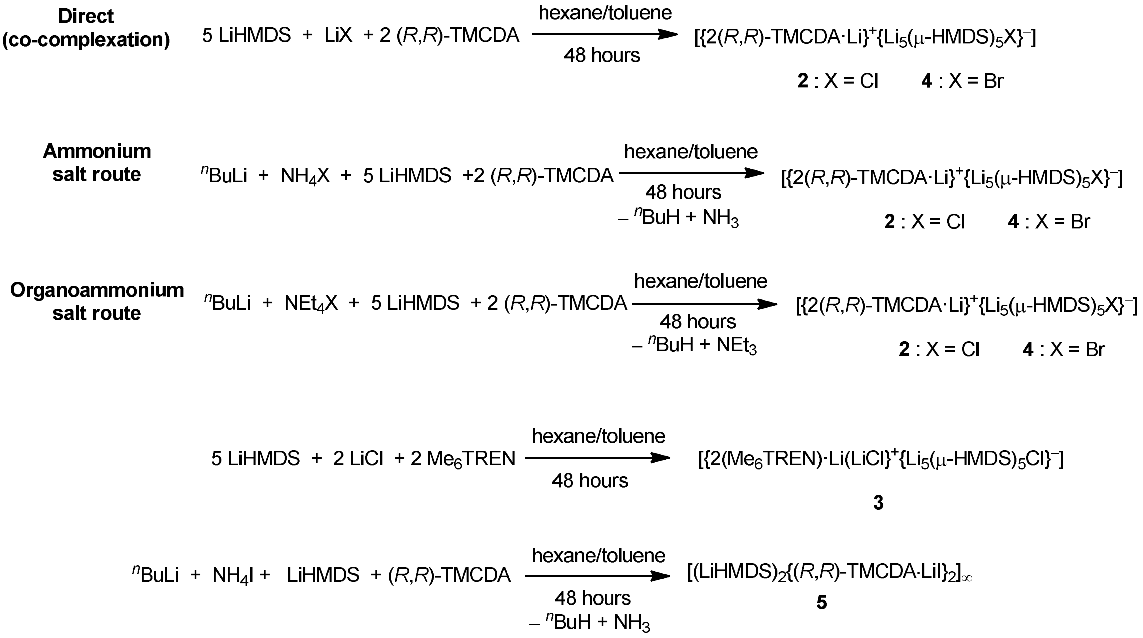

Scheme S1. Rational routes followed in the syntheses of **2**-**5**.

All reactions were performed under a protective argon atmosphere using standard Schlenk techniques. Hexane and toluene were dried by heating to reflux over sodium benzophenone ketyl and distilled under nitrogen prior to use. *n*-Butyllithium (1.6 M solution in hexanes), lithium bis(trimethylsilyl)amide, lithium chloride, lithium bromide and triethylamine hydrochloride were purchased from Aldrich and used as received. Ammonium chloride, ammonium bromide and ammonium iodide were purchased from Alfa Aesar and used as received. *N*,*N*,*N'*,*N'*-(1*R*,2*R*)-tetramethylcyclohexane-1,2-diamine and *tris*[2-dimethylamino)ethyl]amine were prepared according to literature methods (Alexakis *et al. Tetrahedron*, **2005**, *61*, 8939 and White *et al. Inorg. Chem.*, **2005**, *44*, 8125 respectively) and NMR spectra were recorded on a Bruker AV400 spectrometer operating at 400.03 MHz for ^{1}H, 100.59 MHz for ^{13}C and 155.47 MHz for ^{7}Li. Correlations between hydrogen atoms and carbon atoms were obtained through COSY and HSQC NMR spectroscopic methods. Single-crystal X-ray diffraction data were recorded on an Oxford Diffraction Gemini S diffractometer at 123K. Complexes **2-5** are extremely sensitive towards air and moisture. NMR spectroscopic analysis follows the synthetic procedures.

- ***Synthesis of [Li{(R,R)-TMCDA}$_2$]$^+$[Li$_5$(μ-HMDS)$_5$(μ^5-Cl)]$^-$ (2)***

A flame-dried Schlenk tube was charged with lithium bis(trimethylsilyl)amide (0.837 g, 5 mmol) in a glovebox, after which 7.5 mL of dried hexane was added and the mixture allowed to stir for 30 min. Lithium chloride (0.043 g, 1 mmol) was then introduced and the mixture allowed to stir for a further 30 min, after which two molar equivalents of (*R*,*R*)-TMCDA (0.38 mL, 2 mmol) were added and a clear to pale yellow colour change was observed. This suspension was heated slightly and allowed to vigorously stir at ambient temperature for 72 hours to ensure the majority of the LiCl salt had dissolved. The now cloudy white solution was heated and filtered through Celite and glass wool and the resultant clear solution immediately placed in a freezer operating at -28 °C. After 48 h, a small crop of X-ray quality colourless crystals of **2** were deposited (0.10 g, 8%).

Ammonium salt route: A flame-dried Schlenk tube was charged with *n*-butyllithium (0.63 mL of 1.6 M solution in hexanes, 1 mmol) and the hexane removed *in vacuo* and replaced with 5 mL of dried toluene. Two molar equivalents of (*R,R*)-TMCDA (0.38 mL, 2 mmol) were added to give a bright fluorescent red/orange solution which was allowed to stir for 30 min. On the addition of one molar equivalent of ammonium chloride (0.053 g, 1 mmol) this bright fluorescent red/orange colour slowly dissipated on stirring to yield a pale yellow solution. The mixture was heated to reflux for one hour and the clear solution allowed to stir whilst cooling for 30 min. Five molar equivalents of lithium bis(trimethylsilyl)amide (0.837 g, 5 mmol) were then introduced and the resultant pale yellow solution heated slightly and allowed to stir at ambient temperature for 48 hours before being placed in a freezer operating at -72 °C. Precipitation of a non-crystalline solid (0.94 g, 77%) was observed, which by NMR analysis was identical to the crystalline material.

$Et_3N.HCl$ route: A flame-dried Schlenk tube was charged with *n*-butyllithium (0.63 mL of 1.6 M solution in hexanes, 1 mmol) and the hexane removed in vacuo and replaced with 5 mL of dried toluene. Two molar equivalents of (*R,R*)-TMCDA (0.38 mL, 2 mmol) were added to give a bright fluorescent red/orange solution which was allowed to stir for 30 min. On the addition of one molar equivalent of triethylamine hydrochloride (0.138 g, 1mmol) this bright fluorescent red/orange colour slowly dissipated on stirring to yield a pale yellow/peach solution. The mixture was heated to reflux for one hour and the

clear yellow solution allowed to stir whilst cooling for 30 min. Five molar equivalents of lithium bis(trimethylsilyl)amide (0.837 g, 5 mmol) were then introduced affording a more intense yellow colour to the solution. The mixture was heated slightly and allowed to stir at ambient temperature for 48 hours. Toluene (5 mL) was then added to the now slightly cloudy yellow suspension and the solution heated and filtered through Celite and glass wool. The resultant clear yellow solution was concentrated by removal of some solvent *in vacuo* and immediately placed in a freezer operating at -72 °C. After 48 h, a crop of X-ray quality colourless crystals of **2** were deposited (0.58 g 48%).

- ***Synthesis of*** $[Li_2(TREN)_2(\mu_2\text{-}Cl)]^+[Li_5(\mu\text{-}HMDS)_5(\mu_5\text{-}Cl)]^-$ ***(3)***

A flame-dried Schlenk tube was charged with lithium bis(trimethylsilyl)amide (0.837 g, 5 mmol) in a glovebox, after which 7.5 mL of dried hexane was added and the mixture allowed to stir for 30 min. Lithium chloride (0.043 g, 1 mmol) was then introduced and the mixture allowed to stir for a further 30 min, after which Me_6-TREN (0.26 mL, 1 mmol) was added. The colour changes from clear to milky pale yellow to clear yellow. A yellow/orange oily precipitate was observed. This emulsion was heated slightly and allowed to vigorously stir at ambient temperature for 72 hours to ensure the majority of the LiCl salt had dissolved. The solvent was removed *in vacuo* and 10 mL of dried toluene were introduced, yielding a slightly cloudy yellow/orange solution, which on standing deposited a slightly oily precipitate. The solution was heated and filtered through Celite and glass wool. After 2 h at ambient temperature, a crop of X-ray quality colourless crystals of **3** precipitated from the solution (0.13 g, 9%).

- ***Synthesis of*** $[Li\{(R,R)\text{-}TMCDA\}_2]^+[Li_5(\mu\text{-}HMDS)_5(\mu_5\text{-}Br)]^-$ ***(4)***

A flame-dried Schlenk tube was charged with lithium bis(trimethylsilyl)amide (0.837 g, 5 mmol) in a glovebox, after which 7.5 mL of dried hexane was added and the mixture allowed to stir for 30 min. Lithium bromide (0.087 g, 1 mmol) was then introduced and the mixture allowed to stir for a further 30 min, after which two molar equivalents of (*R*,*R*)-TMCDA (0.38 mL, 2 mmol) were added and a clear to pale yellow colour change was observed. This suspension was heated slightly and allowed to vigorously stir at ambient temperature for 72 hours to ensure the majority of the LiBr salt had dissolved. The hexane

was removed *in vacuo* and 7 mL of dried toluene was introduced. The resultant cloudy pale yellow solution was heated and filtered through Celite and glass wool and the resultant clear yellow solution immediately placed in a freezer operating at -28 °C. After 48 h, a crop of X-ray quality colourless crystals of **3** were deposited (0.14 g 11%).

Ammonium salt route: A flame-dried Schlenk tube was charged with *n*-butyllithium (0.63 mL of 1.6 M solution in hexanes, 1 mmol) and the hexane removed in vacuo and replaced with 5 mL of dried toluene. Two molar equivalents of (*R,R*)-TMCDA (0.38 mL, 2 mmol) were added to give a bright fluorescent red/orange solution which was allowed to stir for 30 min. On the addition of one molar equivalent of ammonium bromide (0.098 g, 1 mmol) this bright fluorescent red/orange colour slowly dissipated on stirring to yield a pale pink/peach solution. The mixture was heated to reflux for one hour and the clear solution allowed to stir whilst cooling for 30 min. Five molar equivalents of lithium bis(trimethylsilyl)amide (0.837 g, 5 mmol) were then introduced and the resultant slightly cloudy pale yellow solution heated slightly and allowed to stir at ambient temperature for 24 hours. An additional 5 mL of dried toluene was then introduced and the solution allowed to stir at ambient temperature for a further 12 hours before being placed in a freezer operating at -28 °C. After 48 h, a crop of X-ray quality colourless crystals of **4** were deposited (0.50 g 40%).

- ***Synthesis of [{LiI·(R,R)-TMCDA}$_2${LiHMDS}$_2$]$_\infty$ (5)***

A flame-dried Schlenk tube was charged with n-butyllithium (0.63 mL of 1.6 M solution in hexanes, 1 mmol) and the hexane removed in vacuo and replaced with 5 mL of dried toluene. Two molar equivalents of (*R,R*)-TMCDA (0.38 mL, 2 mmol) were added to give a bright fluorescent red/orange solution which was allowed to stir for 30 min. On the addition of one molar equivalent of ammonium iodide (0.145 g, 1 mmol) this bright fluorescent red/orange colour slowly dissipated with slight heating and stirring to yield a pale pink/peach solution (Note: Schlenk covered with black plastic bag whilst stirring for any length of time to omit as much light as possible since ammonium iodide is light sensitive). The mixture was heated to reflux for one hour and the clear pale yellow solution allowed to stir whilst cooling for 30 min. Seven molar equivalents of lithium bis(trimethylsilyl)amide (1.171 g, 7 mmol) were then introduced and the

resultant slightly cloudy pale yellow solution heated slightly and allowed to stir at ambient temperature for 48 hours. An additional 2.5 mL of dried toluene was then introduced, along with heating, and the solution immediately placed in a hot water-filled Dewar flask. After 24 h, a crop of X-ray quality colourless crystals of **5** precipitated from the solution (0.41 g, 85%).

Microanalytical data: The samples were isolated *in-vacuo,* resulting in the loss of the solvent of crystallisation in the samples. As mentioned previously, complexes **2-5** are extremely sensitive to air and moisture which is a major cause of discrepancies in the analyses.

2: $C_{50}H_{134}ClLi_6N_9Si_{10}$: need, C49.24 H11.07 N10.34; found, C50.98 H10.52 N10.71%

3: $C_{50}H_{134}BrLi_6N_9Si_{10}$: need, C47.51 H10.68 N9.97; found, C47.65 H10.22 N10.12%

4: $C_{54}H_{150}Cl_2Li_7N_{13}Si_{10}$: need, C46.92 H10.94 N13.17; found, C47.00 H10.64 N12.74%

5: $C_{18}H_{46}ILi_2N_3Si_2$: need, C41.23 H8.81 N9.04, get C41.50 H8.92 N9.12%

NMR Spectroscopic data

(*R*,*R*)-TMCDA

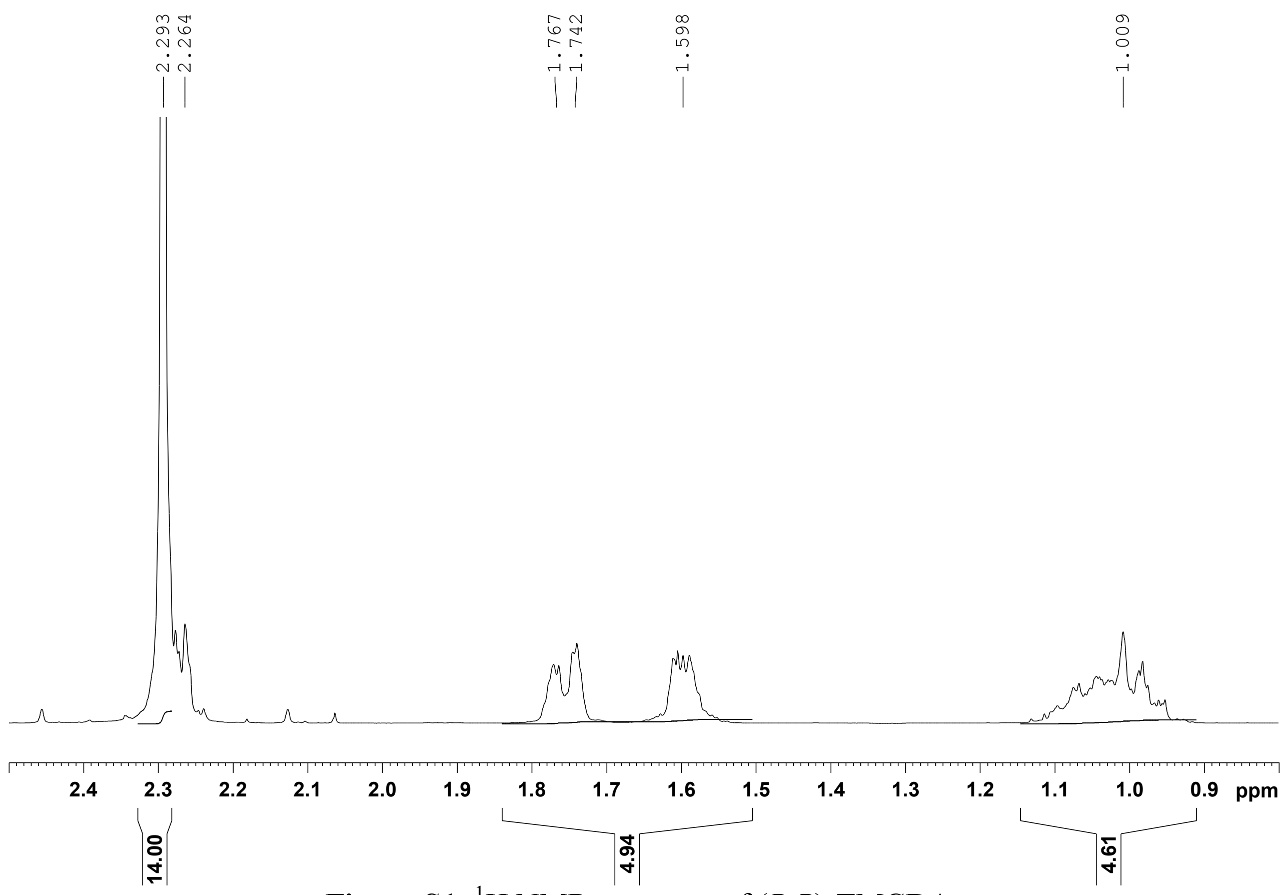

Figure S1: ^{1}H NMR spectrum of (*R*,*R*)-TMCDA.

^{1}H NMR (400.03 MHz, 300 K, C_6D_6): δ 2.29 [(*R*,*R*)TMCDA C*H*$_3$, 12H, s], 2.26 [(*R*,*R*)TMCDA α-*H*, 2H, s], 1.75 [(*R*,*R*)TMCDA β/γ-C*H*$_2$, 2H, br d], 1.60 [(*R*,*R*)TMCDA β/γ-C*H*$_2$, 2H, br m], 1.01 [(*R*,*R*)TMCDA β/γ-C*H*$_2$, 4H, br m].
^{13}C NMR (100.59 MHz, 300 K, C_6D_6): δ 64.34 [(*R*,*R*)TMCDA α-*C*H], 40.62 [(*R*,*R*)TMCDA *C*H$_3$], 25.96 [(*R*,*R*)TMCDA β/γ-*C*H$_2$], 25.67 [(*R*,*R*)TMCDA β/γ-*C*H$_2$].

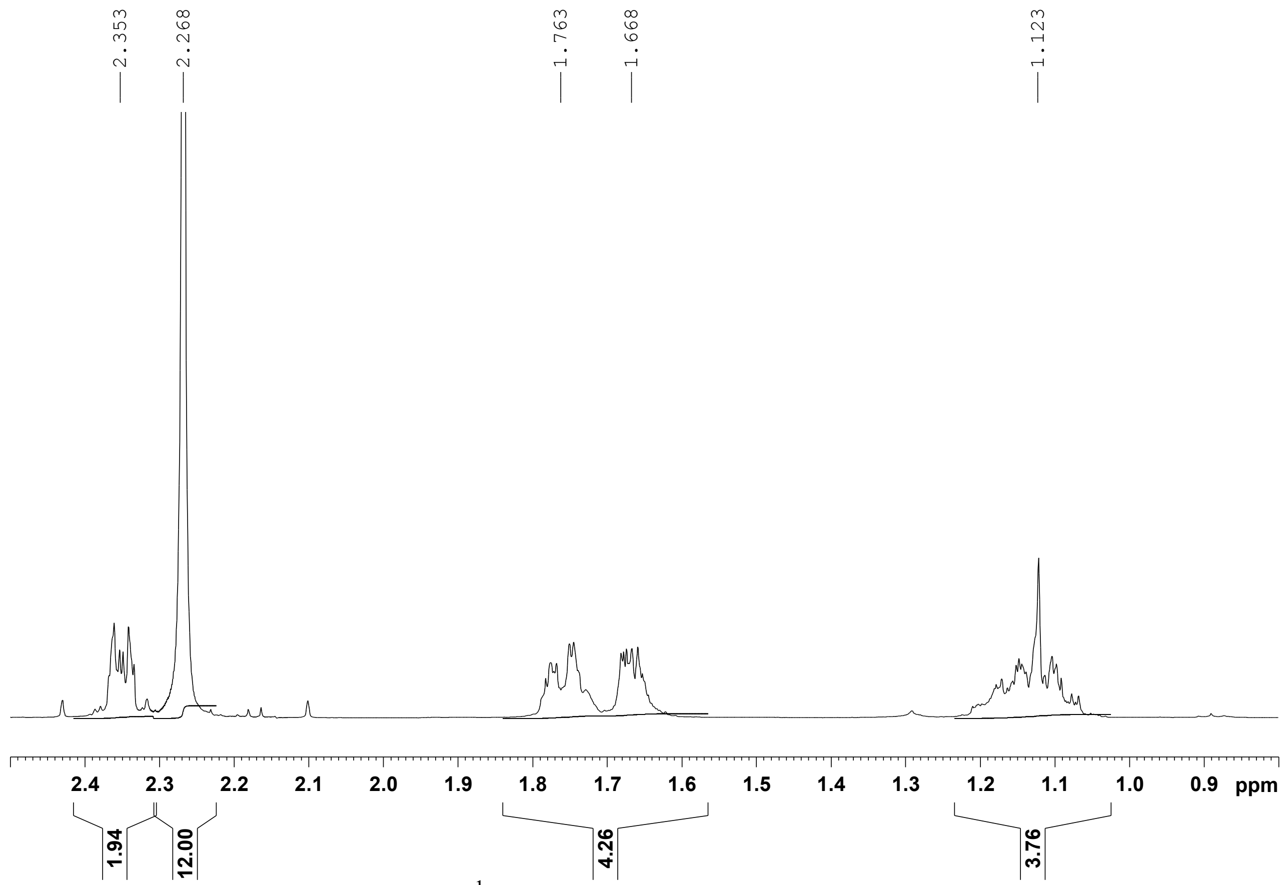

Figure S2: ^{1}H NMR spectrum of (*R*,*R*)-TMCDA.

^{1}H NMR (400.03 MHz, 300 K, D_8-thf): *δ* 2.35 [(*R*,*R*)TMCDA α-*H*, 2H, s], 2.27 [(*R*,*R*)TMCDA C*H*$_3$, 12H, s], 1.76 [(*R*,*R*)TMCDA β/γ-C*H*$_2$, 2H, br d], 1.69 [(*R*,*R*)TMCDA β/γ-C*H*$_2$, 2H, br m], 1.12 [(*R*,*R*)TMCDA β/γ-C*H*$_2$, 4H, br m].

^{13}C NMR (100.59 MHz, 300 K, D_8-thf): *δ* 65.00 [(*R*,*R*)TMCDA α-*C*H], 40.82 [(*R*,*R*)TMCDA *C*H$_3$], 26.54 [(*R*,*R*)TMCDA β/γ-*C*H$_2$].

Me_6-TREN

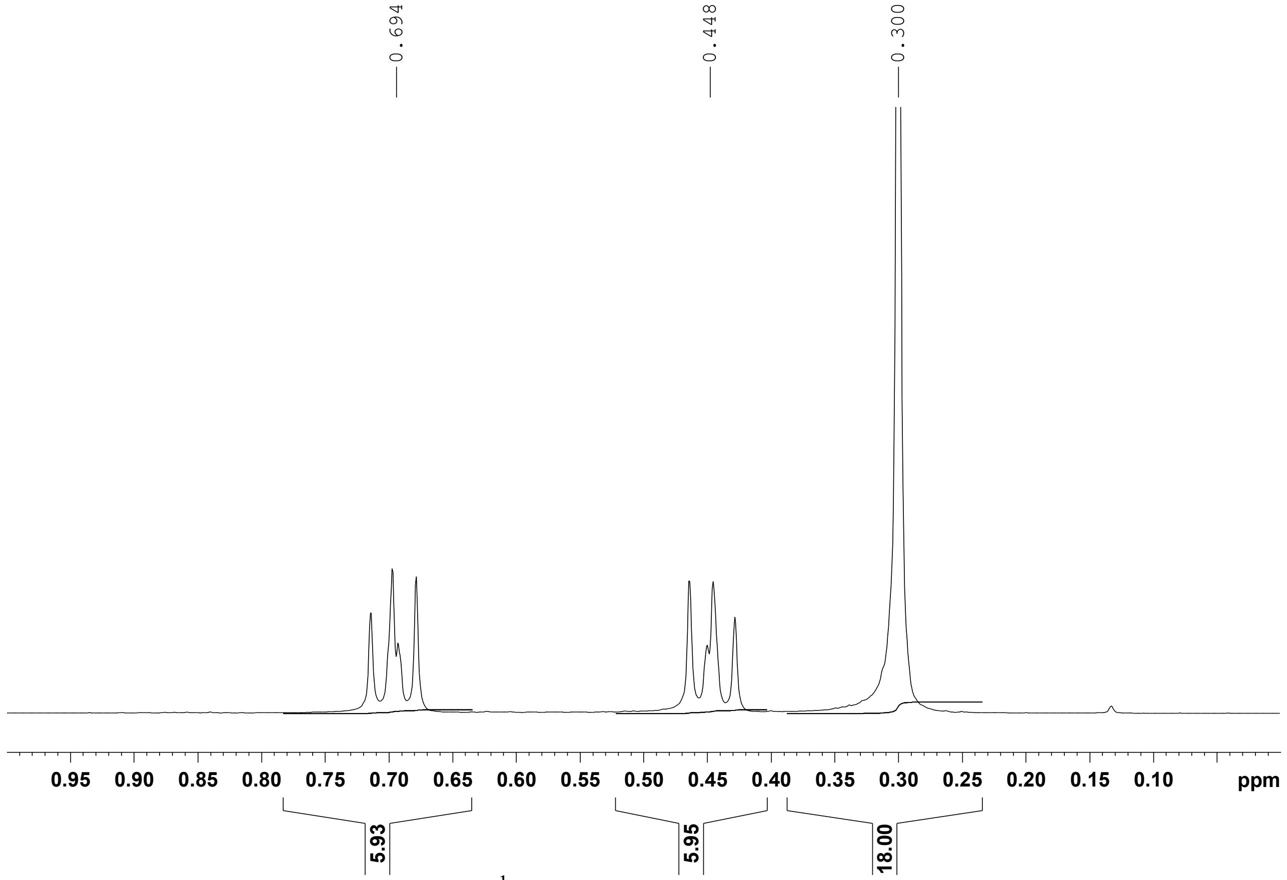

Figure S3: ^{1}H NMR spectrum of Me_6-TREN.

^{1}H NMR (400.03 MHz, 300 K, D_8-thf): δ 0.69 (Me_6-TREN α-CH_2, 6H, s), 0.45 (Me_6-TREN β-CH_2, 6H, s), 0.30 (Me_6-TREN CH_3, 18H, s).

^{13}C NMR (100.59 MHz, 300 K, D_8-thf): δ 59.28 (Me_6-TREN α-CH$_2$), 54.52 (Me_6-TREN β-CH$_2$), 46.31 (Me_6-TREN CH$_3$).

[Li{(*R*,*R*)-TMCDA}$_2$]$^+$[Li$_5$(μ-HMDS)$_5$(μ$_5$-Cl)]$^-$ (2)

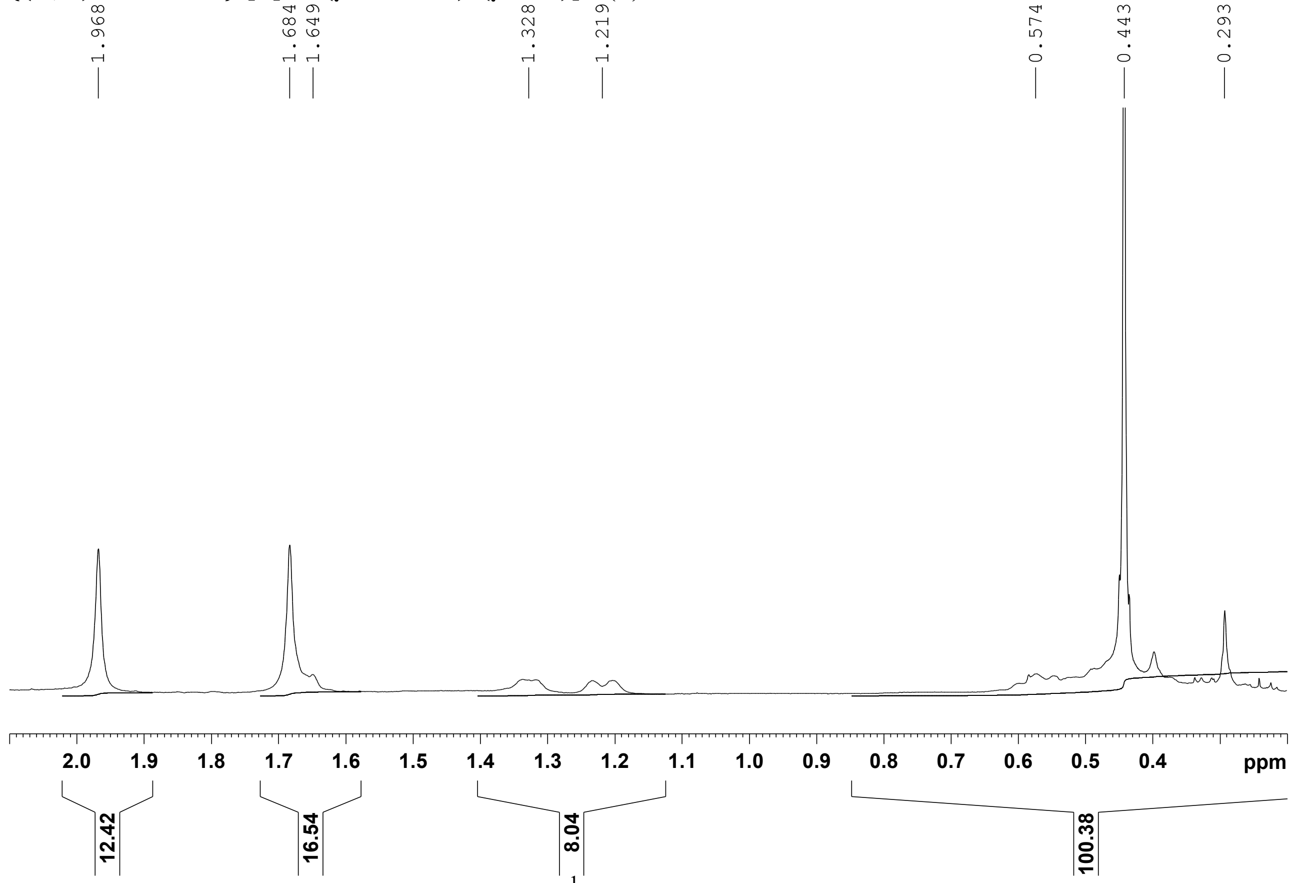

Figure S4: ^{1}H NMR spectrum of **2**.

^{1}H NMR (400.03 MHz, 300 K, C_6D_6): δ 1.97 [(*R*,*R*)TMCDA C*H*$_3$, 12H, s], 1.68 [(*R*,*R*)TMCDA C*H*$_3$, 12H, s], 1.65 [(*R*,*R*)TMCDA α-*H*, 4H, s], 1.33 [(*R*,*R*)TMCDA β/γ-C*H*$_2$, 4H, br d], 1.22 [(*R*,*R*)TMCDA β/γ-C*H*$_2$, 4H, br d], 0.57 [(*R*,*R*)TMCDA β/γ-C*H*$_2$, 8H, br m], 0.44 (SiC*H*$_3$, 90H, s).

^{13}C NMR (100.59 MHz, 300 K, C_6D_6): δ 63.80 [(*R*,*R*)TMCDA α-*C*H], 44.21 [(*R*,*R*)TMCDA *C*H$_3$], 35.81 [(*R*,*R*)TMCDA *C*H$_3$], 24.87 [(*R*,*R*)TMCDA β/γ-*C*H$_2$], 21.42 [(*R*,*R*)TMCDA β/γ-*C*H$_2$], 6.78 (Si*C*H$_3$).

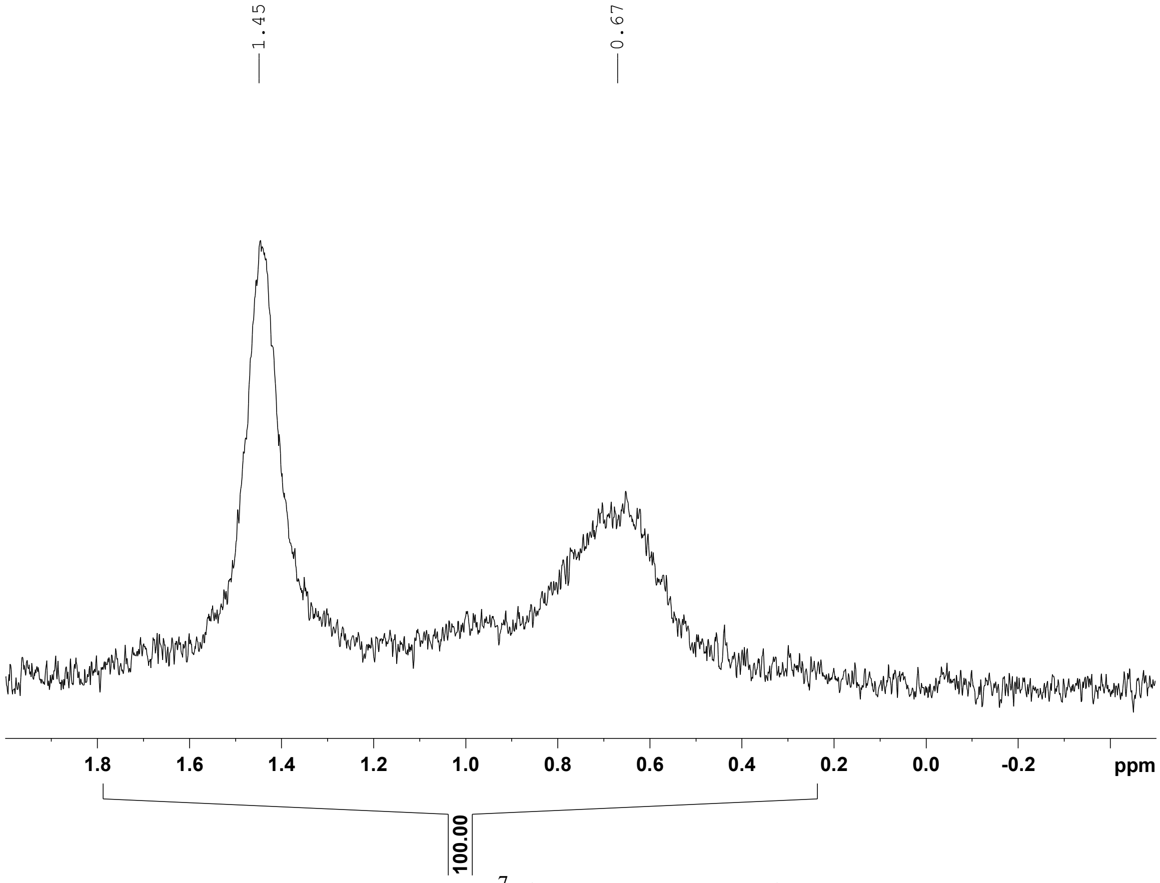

Figure S5: ^{7}Li NMR spectrum of **2**.

^{7}Li NMR (155.47 MHz, 300 K, C_6D_6): *δ* 1.45, 0.67.

$[Li_2(TREN)_2(\mu_2\text{-}Cl)]^+[Li_5(\mu\text{-}HMDS)_5(\mu_5\text{-}Cl)]^-$ (3)

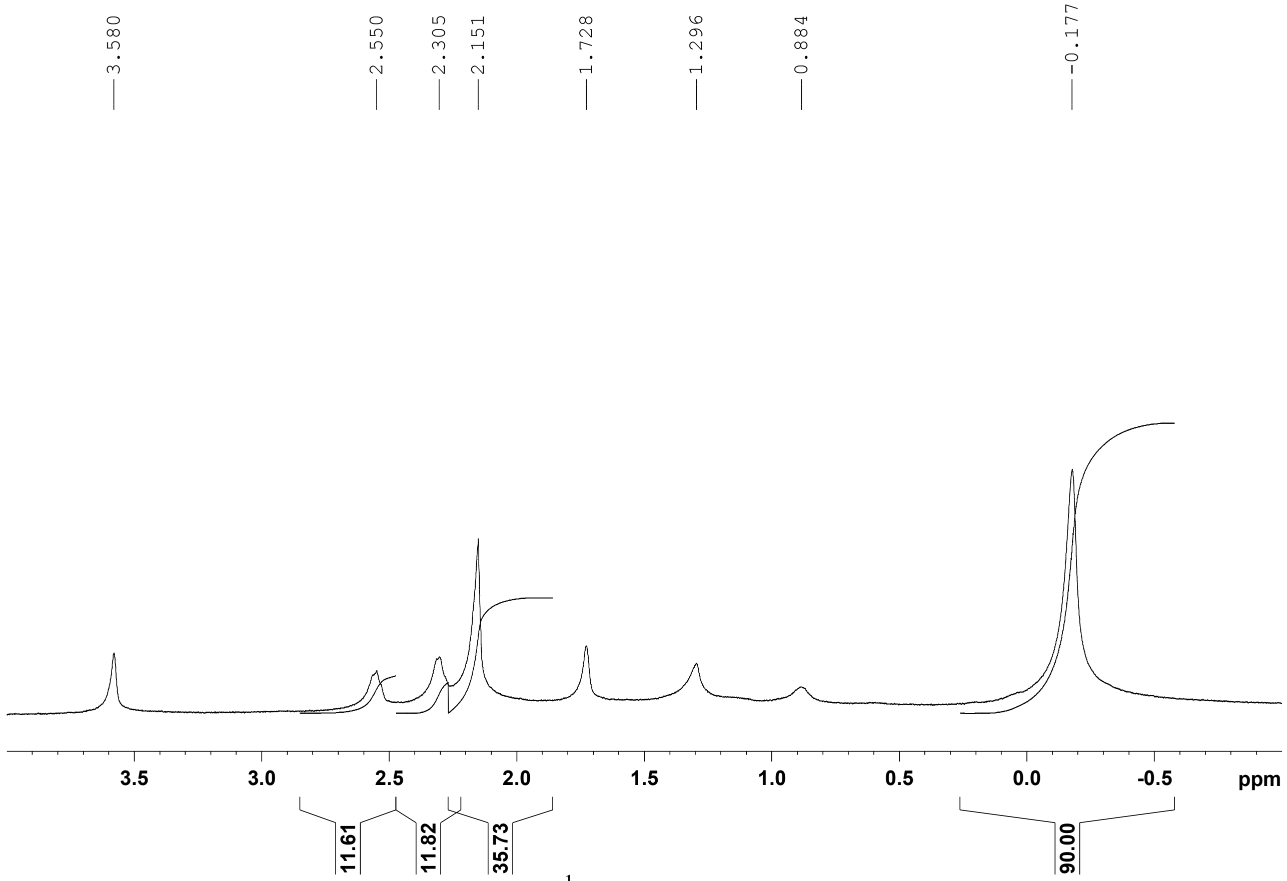

Figure S6: ^{1}H NMR spectrum of **3**.

^{1}H NMR (400.03 MHz, 300 K, D_8-thf): δ 2.55 (Me_6-TREN α-CH_2, 12H, s), 2.31 (Me_6-TREN β-CH_2, 12H, s), 2.15 Me_6-TREN CH_3, 12H, s), 1.30 (grease), 0.88 (grease), -0.18 (SiCH_3, 90H, s)
^{13}C NMR (100.59 MHz, 300 K, D_8-thf): δ 59.27 (Me_6-TREN α-CH_2), 54.51 (Me_6-TREN β-CH_2), 46.28 (Me_6-TREN CH_3), 6.48 (SiCH_3).

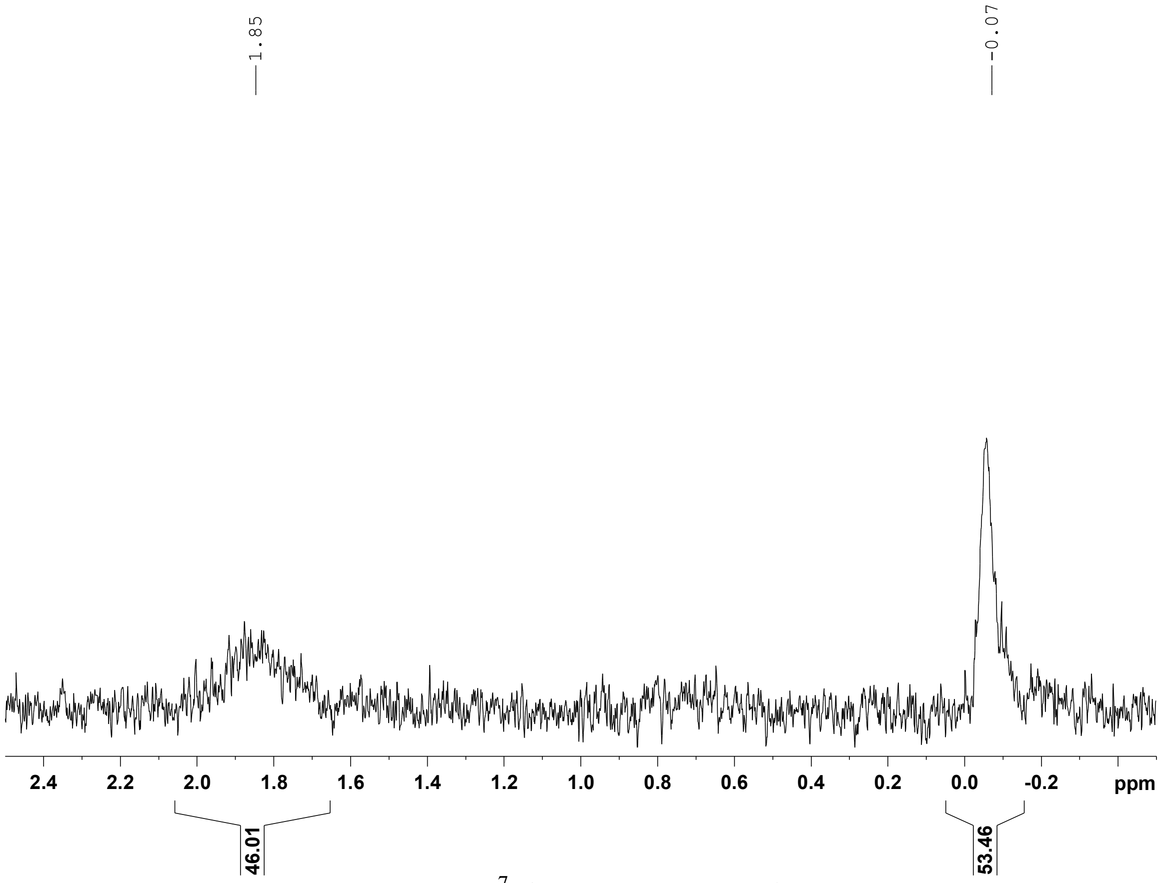

Figure S7: ^{7}Li NMR spectrum of **3**.

^{7}Li NMR (155.47 MHz, 300 K, C_6D_6): δ 1.85, -0.07.

$[Li\{(\boldsymbol{R},\boldsymbol{R})\text{-TMCDA}\}_2]^+[Li_5(\mu\text{-HMDS})_5(\mu_5\text{-Br})]^-$ (4)

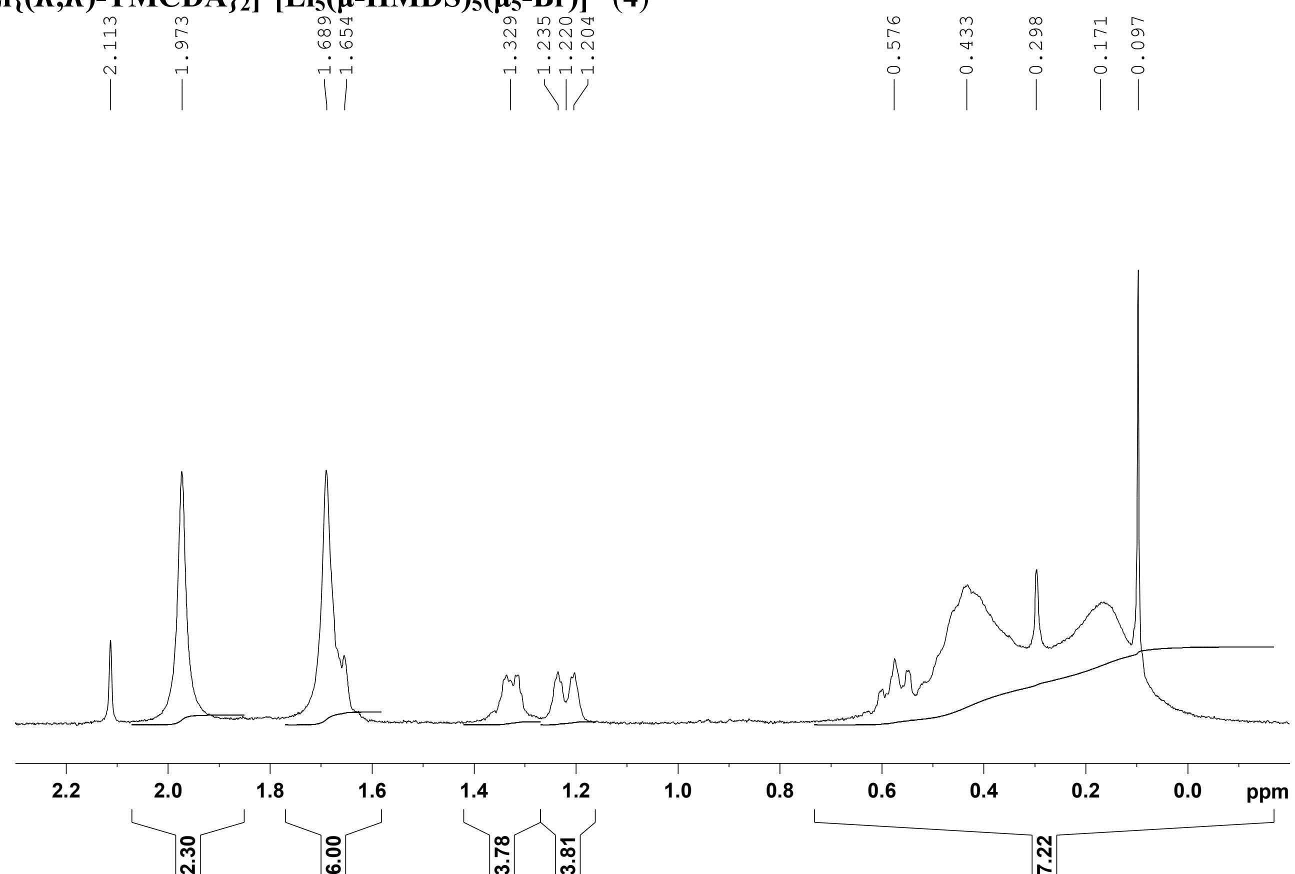

Figure S8: ^{1}H NMR spectrum of **4**. The Si*CH*$_3$ protons are split into two broad resonances, presumably due to dynamic ring fluctuations (see main text).

^{1}H NMR (400.03 MHz, 300 K, C_6D_6): 1.97 [(*R*,*R*)TMCDA C*H*$_3$, 12H, s], 1.69 [(*R*,*R*)TMCDA C*H*$_3$, 12H, s], 1.65 [(*R*,*R*)TMCDA α-*H*, 4H, s], 1.33 [(*R*,*R*)TMCDA β/γ-C*H*$_2$, 4H, br d], 1.22 [(*R*,*R*)TMCDA β/γ-C*H*$_2$, 4H, br d], 0.58 [(*R*,*R*)TMCDA β/γ-C*H*$_2$, 4H, br m], 0.43 [(*R*,*R*)TMCDA β/γ-C*H*$_2$, 4H, br s and SiC*H*$_3$, 45H, br s], 0.30 (silicon grease) , 0.17 (SiC*H*$_3$, 45H, s), 0.097 [HMDS(*H*), s] due to the high sensitivity of the complex towards adventitious H_2O.

^{13}C NMR (100.59 MHz, 300 K, C_6D_6): δ 63.79 [(*R*,*R*)TMCDA α-*C*H], 44.10 [(*R*,*R*)TMCDA *C*H$_3$], 35.76 [(*R*,*R*)TMCDA *C*H$_3$], 24.86 [(*R*,*R*)TMCDA β/γ-*C*H$_2$], 21.41 [(*R*,*R*)TMCDA β/γ-*C*H$_2$], 4.89 (Si*C*H$_3$).

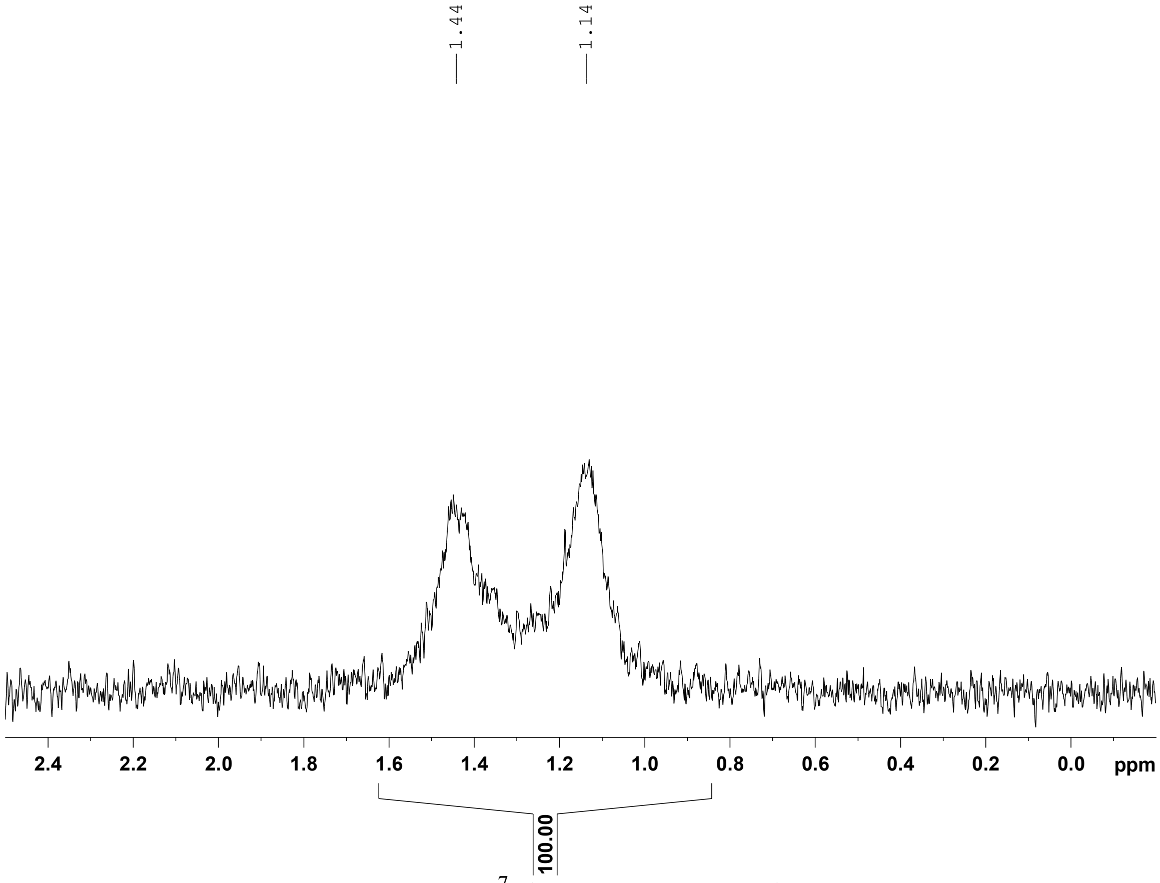

Figure S9: ^{7}Li NMR spectrum of **4**.

^{7}Li NMR (155.47 MHz, 300 K, C_6D_6): δ 1.44, 1.14.

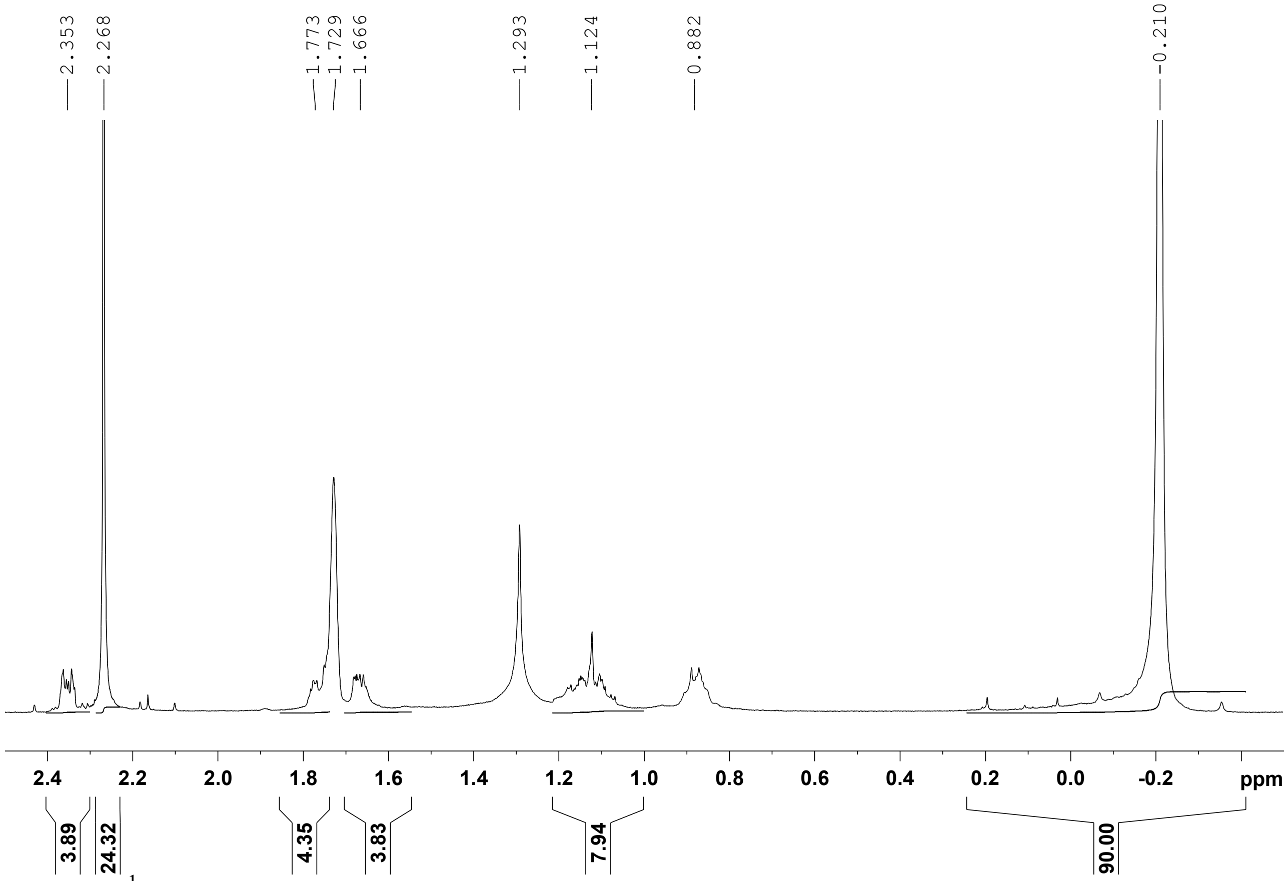

Figure S8: ^{1}H NMR spectrum of **4**. In THF, the SiCH_3 resonance is much sharper indicating a dramatic structural change.

^{1}H NMR (400.03 MHz, 300 K, D_8-thf): δ 2.35 [(*R*,*R*)-TMCDA, α-C*H*, 4H, m] 2.27 [(*R*,*R*)-TMCDA CH_3 24H, s], 1.77 [(*R*,*R*)-TMCDA β/γ-CH_2, 4H, br d], 1.67 [(*R*,*R*)-TMCDA β/γ-CH_2, 4H, br d], 1.12 [(*R*,*R*)-TMCDA β/γ-CH_2, 8H, br m], 0.88 (grease), -0.21 (SiCH_3, 90H, s).

^{13}C NMR (100.59 MHz, 300 K, D_8-thf): δ 65.00 [(*R*,*R*)-TMCDA α-*C*H], 40.81 [(*R*,*R*)TMCDA *C*H_3], 30.64 [(*R*,*R*)-TMCDA *C*H_3], 26.53 [(*R*,*R*)-TMCDA β/γ-*C*H_2], 25.85 [(*R*,*R*)-TMCDA β/γ-*C*H_2], 6.70 (Si*C*H_3).

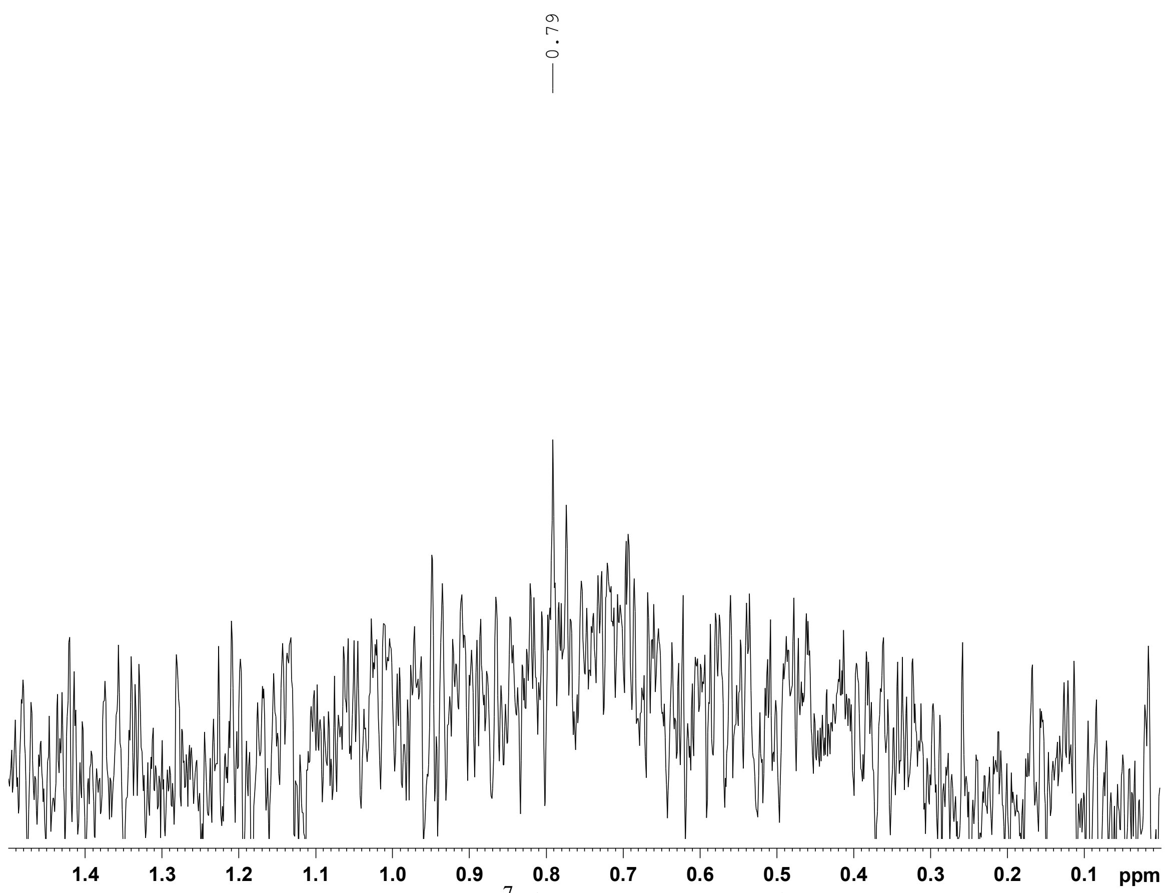

Figure S9: ^{7}Li NMR spectrum of **4**.

^{7}Li NMR (155.47 MHz, 300 K, D_8-thf): *δ* 0.79.

[{LiI·(*R*,*R*)-TMCDA}$_2${LiHMDS}$_2$]$_n$ (5)

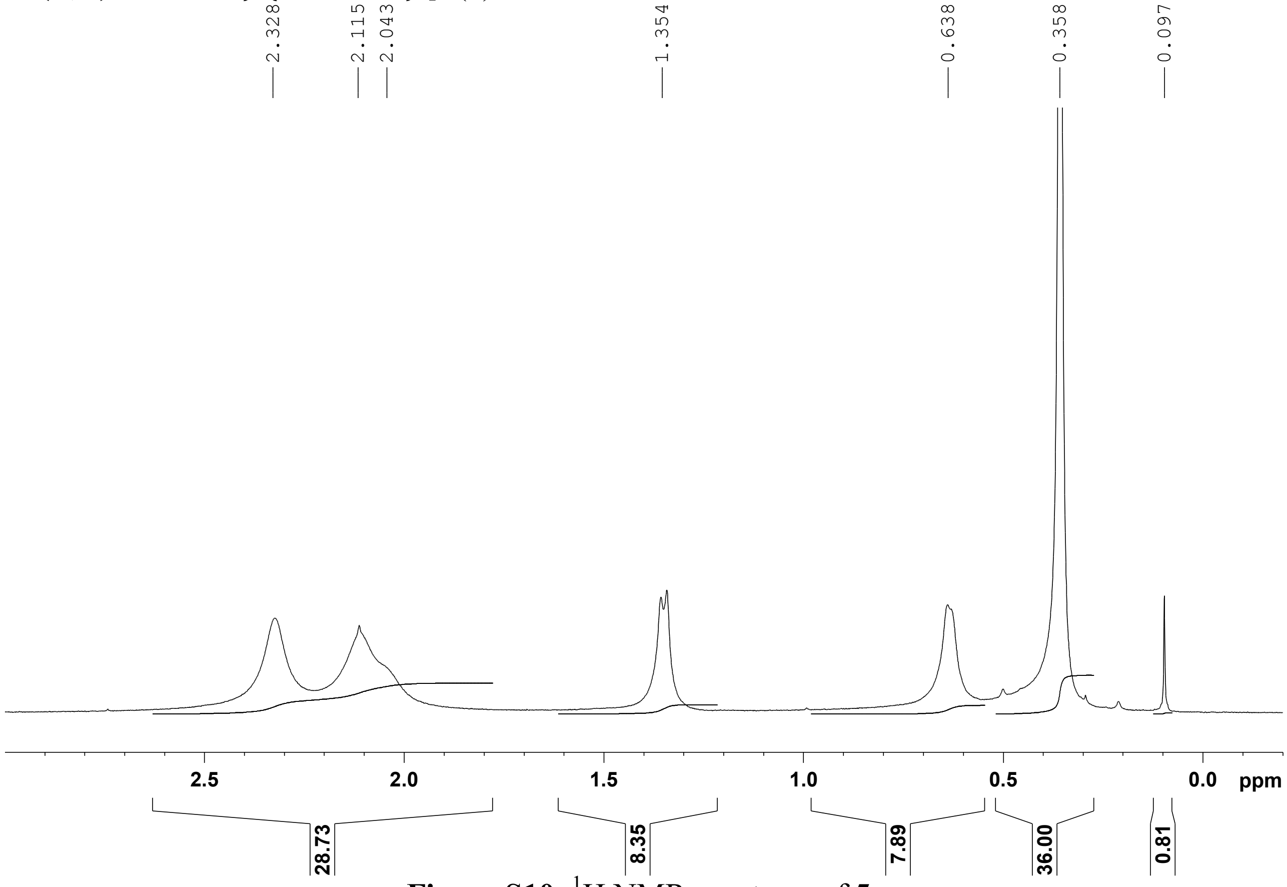

Figure S10: ^{1}H NMR spectrum of **5**.

^{1}H NMR (400.03 MHz, 300 K, C_6D_6): δ 2.33 [(*R*,*R*)-TMCDA C*H*$_3$, 12H, s], 2.12 [(*R*,*R*)-TMCDA C*H*$_3$, 12H, s], 2.04 [(*R*,*R*)-TMCDA α-*H*, 4H, s], 1.35 [(*R*,*R*)-TMCDA β/γ-C*H*$_2$, 8H, br d], 0.64 [(*R*,*R*)-TMCDA β/γ-C*H*$_2$, 8H, br d], 0.36 (SiC*H*$_3$, 36H, s) 0.097 [HMDS(*H*), s].

^{13}C NMR (100.59 MHz, 300 K, C_6D_6): δ 63.89 [(*R*,*R*)-TMCDA α-*C*H], 45.9 [(*R*,*R*)-TMCDA *C*H$_3$], 38.5 [(*R*,*R*)-TMCDA *C*H$_3$], 24.91 [(*R*,*R*)-TMCDA β/γ-*C*H$_2$], 21.70 [(*R*,*R*)TMCDA β/γ-*C*H$_2$], 5.91 (Si*C*H$_3$).

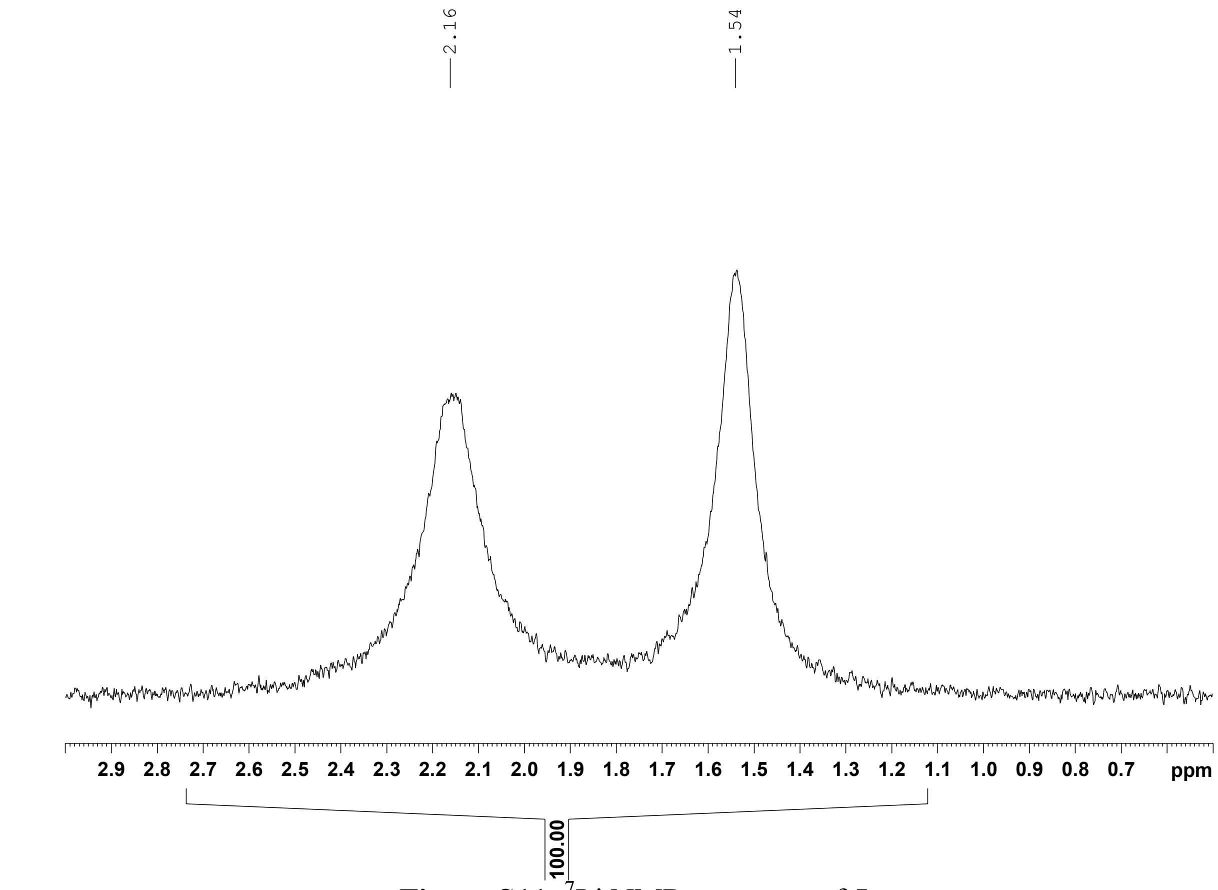

Figure S11: ^{7}Li NMR spectrum of **5**.

^{7}Li NMR (155.47 MHz, 300 K, C_6D_6): δ 2.16, 1.54.

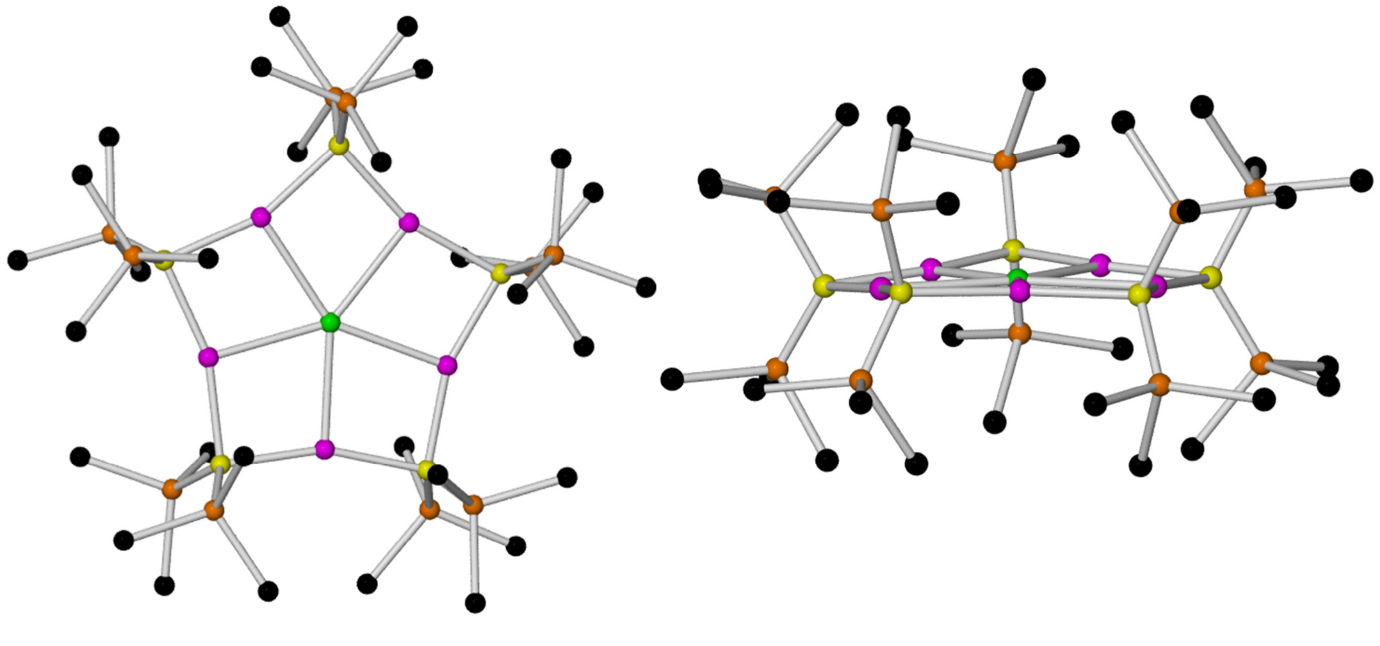

X-ray Data for **2**

Table 1. Crystal data and structure refinement for srgr1006.

Identification code	shelxl	
Empirical formula	C50 H134 Cl1 Li6 N9 Si10	
Formula weight	1219.65	
Temperature	123(2) K	
Wavelength	0.71073 Å	
Crystal system	Monoclinic	
Space group	P21	
Unit cell dimensions	a = 15.3460(4) Å	α= 90°.
	b = 27.9338(7) Å	β= 102.300(2)°.
	c = 19.0059(5) Å	γ = 90°.
Volume	7960.3(4) $Å^3$	
Z	4	
Density (calculated)	1.018 Mg/m^3	
Absorption coefficient	0.232 mm^{-1}	
F(000)	2688	
Crystal size	0.16 x 0.16 x 0.16 mm^3	
Theta range for data collection	3.10 to 28.00°.	
Index ranges	-20<=h<=19, -26<=k<=36, -25<=l<=25	
Reflections collected	42579	
Independent reflections	27286 [R(int) = 0.0317]	
Completeness to theta = 27.00°	99.8 %	
Absorption correction	Semi-empirical from equivalents	
Max. and min. transmission	1.00000 and 0.98218	
Refinement method	Full-matrix least-squares on F^2	
Data / restraints / parameters	27286 / 1 / 1445	
Goodness-of-fit on F^2	0.786	
Final R indices [I>2sigma(I)]	R1 = 0.0390, wR2 = 0.0640	
R indices (all data)	R1 = 0.0774, wR2 = 0.0683	
Absolute structure parameter	-0.08(5)	
Largest diff. peak and hole	0.310 and -0.314 e.$Å^{-3}$	

Table 2. Atomic coordinates (x 10^4) and equivalent isotropic displacement parameters ($Å^2$x 10^3) for srgr1006. U(eq) is defined as one third of the trace of the orthogonalized U^{ij} tensor.

	x	y	z	U(eq)
Li(1)	5350(4)	4196(3)	-750(3)	24(2)
Li(2)	5372(4)	3347(3)	91(4)	29(2)
Li(3)	5150(4)	3636(3)	1503(3)	25(2)
Li(4)	5137(4)	4666(3)	1534(3)	20(2)
Li(5)	5101(4)	5002(3)	107(3)	24(2)
Li(6)	9910(4)	6747(3)	4903(3)	26(2)
Li(7)	9815(4)	7087(3)	3454(3)	26(2)
Li(8)	9828(4)	8113(3)	3489(3)	30(2)
Li(9)	9681(4)	8393(3)	4921(3)	24(2)
Li(10)	9749(4)	7556(3)	5769(3)	26(2)
Li(11)	5045(4)	6051(3)	5253(3)	25(2)
Li(12)	9943(4)	5712(3)	-214(3)	23(2)
Cl(1)	5165(1)	4170(1)	499(1)	27(1)
Cl(2)	9866(1)	7578(1)	4510(1)	27(1)
Si(1)	5363(1)	3104(1)	-1452(1)	30(1)
Si(2)	6955(1)	3653(1)	-736(1)	27(1)
Si(3)	4150(1)	2738(1)	942(1)	25(1)
Si(4)	6125(1)	2665(1)	1252(1)	26(1)
Si(5)	4200(1)	4183(1)	2595(1)	31(1)
Si(6)	6158(1)	4066(1)	2908(1)	28(1)
Si(7)	4077(1)	5617(1)	879(1)	24(1)
Si(8)	6010(1)	5610(1)	1545(1)	23(1)
Si(9)	3857(1)	4761(1)	-1486(1)	23(1)
Si(10)	5525(1)	5320(1)	-1294(1)	24(1)
Si(11)	10870(1)	6134(1)	4090(1)	24(1)
Si(12)	8921(1)	6147(1)	3475(1)	24(1)
Si(13)	10678(1)	7542(1)	2333(1)	33(1)
Si(14)	8733(1)	7701(1)	2132(1)	25(1)
Si(15)	10862(1)	8991(1)	3996(1)	28(1)
Si(16)	8894(1)	9090(1)	3786(1)	25(1)
Si(17)	9777(1)	8652(1)	6461(1)	31(1)
Si(18)	8162(1)	8093(1)	5818(1)	31(1)
Si(19)	11194(1)	6969(1)	6485(1)	21(1)
Si(20)	9528(1)	6420(1)	6304(1)	24(1)
N(1)	5852(2)	3516(1)	-814(2)	21(1)

N(2)	5172(2)	2996(1)	993(2)	18(1)
N(3)	5169(2)	4139(2)	2294(1)	21(1)
N(4)	5080(2)	5325(1)	1073(2)	18(1)
N(5)	4900(2)	4895(1)	-990(2)	19(1)
N(6)	9872(2)	6423(1)	3922(2)	19(1)
N(7)	9748(2)	7602(2)	2681(1)	22(1)
N(8)	9840(2)	8753(1)	4007(2)	22(1)
N(9)	9255(2)	8235(2)	5858(2)	24(1)
N(10)	10149(2)	6850(1)	5999(2)	18(1)
N(11)	4853(2)	6486(2)	4308(2)	25(1)
N(12)	5121(2)	6741(2)	5797(2)	30(1)
N(13)	6051(2)	5543(1)	5670(1)	23(1)
N(14)	4148(2)	5447(1)	5253(2)	21(1)
N(15)	10832(2)	6294(1)	-398(2)	19(1)
N(16)	8969(2)	6252(1)	-388(1)	19(1)
N(17)	9943(2)	5074(2)	-803(2)	22(1)
N(18)	9998(2)	5220(1)	700(2)	20(1)
C(1)	4420(3)	2803(2)	-1140(2)	38(1)
C(2)	4888(3)	3372(2)	-2362(2)	46(1)
C(3)	6100(3)	2598(2)	-1632(2)	47(1)
C(4)	7781(2)	3209(2)	-266(2)	39(1)
C(5)	7272(3)	3783(2)	-1625(2)	40(1)
C(6)	7230(2)	4210(2)	-168(2)	32(1)
C(7)	3255(2)	3127(2)	384(2)	35(1)
C(8)	3869(2)	2673(2)	1859(2)	41(1)
C(9)	4002(3)	2124(2)	539(2)	36(1)
C(10)	6113(3)	2231(2)	2012(2)	44(2)
C(11)	6386(3)	2299(2)	497(2)	39(1)
C(12)	7106(2)	3071(2)	1547(2)	33(1)
C(13)	3215(2)	4030(2)	1865(2)	38(1)
C(14)	3991(3)	4812(2)	2879(2)	56(2)
C(15)	4109(3)	3791(2)	3373(2)	55(2)
C(16)	6315(3)	4437(2)	3753(2)	45(1)
C(17)	6313(3)	3434(2)	3249(2)	41(1)
C(18)	7106(2)	4224(2)	2467(2)	37(1)
C(19)	3158(2)	5172(2)	642(2)	29(1)
C(20)	3789(3)	5996(2)	1607(2)	44(2)
C(21)	3985(2)	6022(2)	83(2)	35(1)
C(22)	6125(3)	6260(2)	1343(2)	39(1)
C(23)	6048(3)	5588(2)	2537(2)	33(1)

C(24)	7037(2)	5299(2)	1374(2)	29(1)
C(25)	2972(2)	5208(2)	-1448(2)	38(1)
C(26)	3452(2)	4179(2)	-1165(2)	35(1)
C(27)	3848(3)	4674(2)	-2476(2)	33(1)
C(28)	4903(3)	5840(2)	-1817(2)	42(1)
C(29)	6274(2)	5081(2)	-1870(2)	38(1)
C(30)	6282(2)	5616(2)	-513(2)	35(1)
C(31)	11799(2)	6597(2)	4300(2)	32(1)
C(32)	10973(3)	5726(2)	4887(2)	33(1)
C(33)	11118(3)	5748(2)	3334(2)	38(1)
C(34)	8841(3)	6187(2)	2476(2)	34(1)
C(35)	7909(2)	6445(2)	3674(2)	33(1)
C(36)	8803(3)	5492(2)	3684(2)	37(1)
C(37)	10703(3)	7929(2)	1525(2)	55(2)
C(38)	11716(2)	7695(2)	3004(2)	41(1)
C(39)	10828(3)	6909(2)	2041(2)	51(2)
C(40)	8594(3)	8336(2)	1813(2)	37(1)
C(41)	7826(2)	7550(2)	2615(2)	38(1)
C(42)	8461(3)	7336(2)	1279(2)	42(1)
C(43)	11056(3)	9061(2)	3053(2)	44(1)
C(44)	11771(2)	8597(2)	4510(2)	36(1)
C(45)	11084(3)	9616(2)	4393(2)	42(1)
C(46)	8706(3)	9450(2)	4577(2)	41(1)
C(47)	8855(3)	9533(2)	3027(2)	39(1)
C(48)	7885(2)	8693(2)	3526(2)	33(1)
C(49)	10693(3)	8947(2)	6101(2)	42(1)
C(50)	9066(3)	9161(2)	6670(2)	48(1)
C(51)	10320(3)	8396(2)	7363(2)	47(1)
C(52)	7329(2)	8563(2)	5381(2)	40(1)
C(53)	7924(3)	7968(2)	6737(2)	43(1)
C(54)	7861(2)	7531(2)	5278(2)	40(1)
C(55)	11627(2)	7533(2)	6164(2)	32(1)
C(56)	11225(2)	7051(2)	7466(2)	33(1)
C(57)	12075(2)	6501(2)	6428(2)	29(1)
C(58)	8839(2)	6646(2)	6950(2)	36(1)
C(59)	10163(3)	5901(2)	6775(2)	35(1)
C(60)	8710(2)	6145(2)	5524(2)	32(1)
C(61)	5755(2)	6556(2)	4157(2)	38(1)
C(62)	4279(3)	6277(2)	3656(2)	42(1)
C(63)	4464(2)	6944(1)	4511(2)	24(1)

C(64)	4987(2)	7129(1)	5241(2)	26(1)
C(65)	4571(3)	7590(2)	5457(2)	38(1)
C(66)	4502(3)	7976(1)	4894(2)	48(1)
C(67)	3951(3)	7795(2)	4179(2)	52(1)
C(68)	4367(3)	7337(2)	3949(2)	38(1)
C(69)	4349(3)	6699(2)	6156(2)	47(1)
C(70)	5931(3)	6844(2)	6360(2)	52(1)
C(71)	6738(3)	5704(2)	6273(2)	43(1)
C(72)	6459(2)	5472(2)	5047(2)	35(1)
C(73)	3441(2)	5395(2)	4589(2)	36(1)
C(74)	3707(2)	5530(2)	5857(2)	30(1)
C(75)	5615(2)	5105(1)	5878(2)	23(1)
C(76)	4714(2)	5004(1)	5358(2)	24(1)
C(77)	4276(2)	4562(2)	5593(2)	28(1)
C(78)	4870(2)	4122(1)	5674(2)	36(1)
C(79)	5741(2)	4224(1)	6210(2)	38(1)
C(80)	6194(2)	4653(2)	5968(2)	31(1)
C(81)	11424(2)	6161(2)	-882(2)	28(1)
C(82)	11393(2)	6419(2)	302(2)	28(1)
C(83)	8381(2)	6127(2)	-1093(2)	33(1)
C(84)	8422(2)	6242(2)	167(2)	30(1)
C(85)	10230(2)	6691(1)	-721(2)	18(1)
C(86)	9407(2)	6725(1)	-388(2)	20(1)
C(87)	8787(2)	7129(2)	-738(2)	26(1)
C(88)	9259(2)	7612(1)	-708(2)	32(1)
C(89)	10057(2)	7575(1)	-1060(2)	32(1)
C(90)	10693(2)	7183(2)	-692(2)	27(1)
C(91)	10306(2)	5114(2)	-1454(2)	36(1)
C(92)	9008(2)	4901(1)	-1020(2)	34(1)
C(93)	10781(2)	5424(2)	1212(2)	30(1)
C(94)	9245(2)	5204(2)	1079(2)	36(1)
C(95)	10510(2)	4756(1)	-255(2)	25(1)
C(96)	10154(2)	4738(1)	432(2)	22(1)
C(97)	10747(3)	4410(2)	1005(2)	34(1)
C(98)	10833(3)	3914(2)	715(2)	47(1)
C(99)	11217(3)	3941(1)	54(2)	52(1)
C(100)	10644(2)	4250(2)	-527(2)	37(1)

Table 3. Bond lengths [Å] and angles [°] for srgr1006.

Li(1)-N(1)	2.065(9)
Li(1)-N(5)	2.090(9)
Li(1)-Cl(1)	2.451(6)
Li(1)-Li(5)	2.851(12)
Li(1)-Li(2)	2.857(12)
Li(1)-Si(9)	2.887(7)
Li(1)-Si(2)	2.887(7)
Li(2)-N(2)	2.053(8)
Li(2)-N(1)	2.065(8)
Li(2)-Cl(1)	2.468(8)
Li(2)-Li(3)	2.891(10)
Li(2)-Si(4)	2.957(8)
Li(2)-Si(1)	3.008(7)
Li(3)-N(2)	2.038(8)
Li(3)-N(3)	2.053(8)
Li(3)-Cl(1)	2.426(7)
Li(3)-Li(4)	2.877(11)
Li(3)-Si(3)	3.015(8)
Li(3)-Si(6)	3.034(7)
Li(3)-Si(5)	3.171(7)
Li(3)-Si(4)	3.182(8)
Li(4)-N(4)	2.033(9)
Li(4)-N(3)	2.054(8)
Li(4)-Cl(1)	2.414(7)
Li(4)-Li(5)	2.859(9)
Li(4)-Si(8)	2.955(8)
Li(4)-Si(5)	3.032(7)
Li(5)-N(4)	2.051(8)
Li(5)-N(5)	2.064(7)
Li(5)-Cl(1)	2.435(8)
Li(5)-Si(7)	2.926(8)
Li(5)-Si(10)	3.007(7)
Li(6)-N(10)	2.057(7)
Li(6)-N(6)	2.062(8)
Li(6)-Cl(2)	2.434(9)
Li(6)-Li(10)	2.837(12)
Li(6)-Li(7)	2.886(9)
Li(6)-Si(11)	2.908(8)

Li(6)-Si(20)	2.990(7)
Li(7)-N(7)	2.045(8)
Li(7)-N(6)	2.050(9)
Li(7)-Cl(2)	2.418(7)
Li(7)-Li(8)	2.867(12)
Li(7)-Si(12)	2.967(8)
Li(7)-Si(13)	3.020(7)
Li(8)-N(8)	2.041(10)
Li(8)-N(7)	2.080(9)
Li(8)-Cl(2)	2.439(7)
Li(8)-Li(9)	2.887(10)
Li(8)-Si(15)	2.969(9)
Li(8)-Si(14)	2.992(7)
Li(8)-Si(16)	3.188(9)
Li(9)-N(8)	2.068(7)
Li(9)-N(9)	2.071(7)
Li(9)-Cl(2)	2.444(8)
Li(9)-Li(10)	2.829(11)
Li(9)-Si(16)	2.966(7)
Li(9)-Si(17)	2.986(7)
Li(10)-N(9)	2.065(9)
Li(10)-N(10)	2.083(9)
Li(10)-Cl(2)	2.438(6)
Li(10)-Si(19)	2.859(7)
Li(10)-Si(18)	2.880(7)
Li(11)-N(13)	2.121(8)
Li(11)-N(11)	2.136(8)
Li(11)-N(14)	2.178(9)
Li(11)-N(12)	2.179(9)
Li(12)-N(16)	2.101(8)
Li(12)-N(17)	2.104(9)
Li(12)-N(15)	2.198(8)
Li(12)-N(18)	2.202(8)
Si(1)-N(1)	1.723(4)
Si(1)-C(1)	1.875(4)
Si(1)-C(2)	1.883(4)
Si(1)-C(3)	1.886(5)
Si(2)-N(1)	1.710(3)
Si(2)-C(4)	1.862(5)
Si(2)-C(6)	1.889(5)

Si(2)-C(5)	1.890(4)
Si(3)-N(2)	1.711(3)
Si(3)-C(9)	1.873(5)
Si(3)-C(8)	1.889(4)
Si(3)-C(7)	1.891(4)
Si(4)-N(2)	1.711(3)
Si(4)-C(12)	1.872(4)
Si(4)-C(11)	1.875(4)
Si(4)-C(10)	1.889(5)
Si(5)-N(3)	1.709(3)
Si(5)-C(15)	1.867(5)
Si(5)-C(13)	1.870(4)
Si(5)-C(14)	1.887(6)
Si(6)-N(3)	1.719(3)
Si(6)-C(18)	1.879(4)
Si(6)-C(17)	1.880(5)
Si(6)-C(16)	1.882(4)
Si(7)-N(4)	1.712(3)
Si(7)-C(19)	1.861(4)
Si(7)-C(20)	1.869(5)
Si(7)-C(21)	1.870(5)
Si(8)-N(4)	1.712(3)
Si(8)-C(23)	1.873(4)
Si(8)-C(22)	1.874(6)
Si(8)-C(24)	1.887(4)
Si(9)-N(5)	1.718(3)
Si(9)-C(25)	1.857(5)
Si(9)-C(26)	1.887(5)
Si(9)-C(27)	1.894(4)
Si(10)-N(5)	1.700(3)
Si(10)-C(29)	1.870(4)
Si(10)-C(30)	1.872(4)
Si(10)-C(28)	1.900(5)
Si(11)-N(6)	1.700(3)
Si(11)-C(32)	1.876(4)
Si(11)-C(33)	1.899(4)
Si(11)-C(31)	1.903(5)
Si(12)-N(6)	1.709(3)
Si(12)-C(35)	1.872(4)
Si(12)-C(34)	1.879(4)

Si(12)-C(36)	1.887(5)
Si(13)-N(7)	1.705(3)
Si(13)-C(38)	1.866(4)
Si(13)-C(39)	1.882(6)
Si(13)-C(37)	1.884(5)
Si(14)-N(7)	1.702(3)
Si(14)-C(41)	1.872(4)
Si(14)-C(40)	1.872(5)
Si(14)-C(42)	1.885(4)
Si(15)-N(8)	1.708(3)
Si(15)-C(44)	1.880(4)
Si(15)-C(43)	1.888(4)
Si(15)-C(45)	1.903(5)
Si(16)-N(8)	1.704(4)
Si(16)-C(48)	1.882(4)
Si(16)-C(46)	1.883(4)
Si(16)-C(47)	1.891(4)
Si(17)-N(9)	1.707(4)
Si(17)-C(51)	1.881(4)
Si(17)-C(49)	1.881(4)
Si(17)-C(50)	1.887(5)
Si(18)-N(9)	1.709(3)
Si(18)-C(54)	1.879(5)
Si(18)-C(53)	1.892(4)
Si(18)-C(52)	1.895(5)
Si(19)-N(10)	1.704(3)
Si(19)-C(55)	1.862(5)
Si(19)-C(56)	1.870(4)
Si(19)-C(57)	1.899(4)
Si(20)-N(10)	1.712(4)
Si(20)-C(59)	1.866(5)
Si(20)-C(60)	1.888(4)
Si(20)-C(58)	1.893(4)
N(11)-C(62)	1.479(5)
N(11)-C(61)	1.485(4)
N(11)-C(63)	1.496(5)
N(12)-C(70)	1.485(4)
N(12)-C(69)	1.492(4)
N(12)-C(64)	1.497(5)
N(13)-C(71)	1.453(4)

N(13)-C(72)	1.467(4)
N(13)-C(75)	1.490(4)
N(14)-C(74)	1.469(4)
N(14)-C(73)	1.486(4)
N(14)-C(76)	1.501(5)
N(15)-C(82)	1.465(4)
N(15)-C(81)	1.472(4)
N(15)-C(85)	1.490(4)
N(16)-C(86)	1.480(4)
N(16)-C(84)	1.481(4)
N(16)-C(83)	1.488(4)
N(17)-C(91)	1.467(4)
N(17)-C(92)	1.487(4)
N(17)-C(95)	1.497(4)
N(18)-C(96)	1.479(5)
N(18)-C(94)	1.487(4)
N(18)-C(93)	1.489(4)
C(1)-H(1A)	0.9800
C(1)-H(1B)	0.9800
C(1)-H(1C)	0.9800
C(2)-H(2A)	0.9800
C(2)-H(2B)	0.9800
C(2)-H(2C)	0.9800
C(3)-H(3A)	0.9800
C(3)-H(3B)	0.9800
C(3)-H(3C)	0.9800
C(4)-H(4A)	0.9800
C(4)-H(4B)	0.9800
C(4)-H(4C)	0.9800
C(5)-H(5A)	0.9800
C(5)-H(5B)	0.9800
C(5)-H(5C)	0.9800
C(6)-H(6A)	0.9800
C(6)-H(6B)	0.9800
C(6)-H(6C)	0.9800
C(7)-H(7A)	0.9800
C(7)-H(7B)	0.9800
C(7)-H(7C)	0.9800
C(8)-H(8A)	0.9800
C(8)-H(8B)	0.9800

C(8)-H(8C)	0.9800
C(9)-H(9A)	0.9800
C(9)-H(9B)	0.9800
C(9)-H(9C)	0.9800
C(10)-H(10A)	0.9800
C(10)-H(10B)	0.9800
C(10)-H(10C)	0.9800
C(11)-H(11A)	0.9800
C(11)-H(11B)	0.9800
C(11)-H(11C)	0.9800
C(12)-H(12A)	0.9800
C(12)-H(12B)	0.9800
C(12)-H(12C)	0.9800
C(13)-H(13A)	0.9800
C(13)-H(13B)	0.9800
C(13)-H(13C)	0.9800
C(14)-H(14A)	0.9800
C(14)-H(14B)	0.9800
C(14)-H(14C)	0.9800
C(15)-H(15A)	0.9800
C(15)-H(15B)	0.9800
C(15)-H(15C)	0.9800
C(16)-H(16A)	0.9800
C(16)-H(16B)	0.9800
C(16)-H(16C)	0.9800
C(17)-H(17A)	0.9800
C(17)-H(17B)	0.9800
C(17)-H(17C)	0.9800
C(18)-H(18A)	0.9800
C(18)-H(18B)	0.9800
C(18)-H(18C)	0.9800
C(19)-H(19A)	0.9800
C(19)-H(19B)	0.9800
C(19)-H(19C)	0.9800
C(20)-H(20A)	0.9800
C(20)-H(20B)	0.9800
C(20)-H(20C)	0.9800
C(21)-H(21A)	0.9800
C(21)-H(21B)	0.9800
C(21)-H(21C)	0.9800

C(22)-H(22A)	0.9800
C(22)-H(22B)	0.9800
C(22)-H(22C)	0.9800
C(23)-H(23A)	0.9800
C(23)-H(23B)	0.9800
C(23)-H(23C)	0.9800
C(24)-H(24A)	0.9800
C(24)-H(24B)	0.9800
C(24)-H(24C)	0.9800
C(25)-H(25A)	0.9800
C(25)-H(25B)	0.9800
C(25)-H(25C)	0.9800
C(26)-H(26A)	0.9800
C(26)-H(26B)	0.9800
C(26)-H(26C)	0.9800
C(27)-H(27A)	0.9800
C(27)-H(27B)	0.9800
C(27)-H(27C)	0.9800
C(28)-H(28A)	0.9800
C(28)-H(28B)	0.9800
C(28)-H(28C)	0.9800
C(29)-H(29A)	0.9800
C(29)-H(29B)	0.9800
C(29)-H(29C)	0.9800
C(30)-H(30A)	0.9800
C(30)-H(30B)	0.9800
C(30)-H(30C)	0.9800
C(31)-H(31A)	0.9800
C(31)-H(31B)	0.9800
C(31)-H(31C)	0.9800
C(32)-H(32A)	0.9800
C(32)-H(32B)	0.9800
C(32)-H(32C)	0.9800
C(33)-H(33A)	0.9800
C(33)-H(33B)	0.9800
C(33)-H(33C)	0.9800
C(34)-H(34A)	0.9800
C(34)-H(34B)	0.9800
C(34)-H(34C)	0.9800
C(35)-H(35A)	0.9800

C(35)-H(35B)	0.9800
C(35)-H(35C)	0.9800
C(36)-H(36A)	0.9800
C(36)-H(36B)	0.9800
C(36)-H(36C)	0.9800
C(37)-H(37A)	0.9800
C(37)-H(37B)	0.9800
C(37)-H(37C)	0.9800
C(38)-H(38A)	0.9800
C(38)-H(38B)	0.9800
C(38)-H(38C)	0.9800
C(39)-H(39A)	0.9800
C(39)-H(39B)	0.9800
C(39)-H(39C)	0.9800
C(40)-H(40A)	0.9800
C(40)-H(40B)	0.9800
C(40)-H(40C)	0.9800
C(41)-H(41A)	0.9800
C(41)-H(41B)	0.9800
C(41)-H(41C)	0.9800
C(42)-H(42A)	0.9800
C(42)-H(42B)	0.9800
C(42)-H(42C)	0.9800
C(43)-H(43A)	0.9800
C(43)-H(43B)	0.9800
C(43)-H(43C)	0.9800
C(44)-H(44A)	0.9800
C(44)-H(44B)	0.9800
C(44)-H(44C)	0.9800
C(45)-H(45A)	0.9800
C(45)-H(45B)	0.9800
C(45)-H(45C)	0.9800
C(46)-H(46A)	0.9800
C(46)-H(46B)	0.9800
C(46)-H(46C)	0.9800
C(47)-H(47A)	0.9800
C(47)-H(47B)	0.9800
C(47)-H(47C)	0.9800
C(48)-H(48A)	0.9800
C(48)-H(48B)	0.9800

C(48)-H(48C)	0.9800
C(49)-H(49A)	0.9800
C(49)-H(49B)	0.9800
C(49)-H(49C)	0.9800
C(50)-H(50A)	0.9800
C(50)-H(50B)	0.9800
C(50)-H(50C)	0.9800
C(51)-H(51A)	0.9800
C(51)-H(51B)	0.9800
C(51)-H(51C)	0.9800
C(52)-H(52A)	0.9800
C(52)-H(52B)	0.9800
C(52)-H(52C)	0.9800
C(53)-H(53A)	0.9800
C(53)-H(53B)	0.9800
C(53)-H(53C)	0.9800
C(54)-H(54A)	0.9800
C(54)-H(54B)	0.9800
C(54)-H(54C)	0.9800
C(55)-H(55A)	0.9800
C(55)-H(55B)	0.9800
C(55)-H(55C)	0.9800
C(56)-H(56A)	0.9800
C(56)-H(56B)	0.9800
C(56)-H(56C)	0.9800
C(57)-H(57A)	0.9800
C(57)-H(57B)	0.9800
C(57)-H(57C)	0.9800
C(58)-H(58A)	0.9800
C(58)-H(58B)	0.9800
C(58)-H(58C)	0.9800
C(59)-H(59A)	0.9800
C(59)-H(59B)	0.9800
C(59)-H(59C)	0.9800
C(60)-H(60A)	0.9800
C(60)-H(60B)	0.9800
C(60)-H(60C)	0.9800
C(61)-H(61A)	0.9800
C(61)-H(61B)	0.9800
C(61)-H(61C)	0.9800

C(62)-H(62A)	0.9800
C(62)-H(62B)	0.9800
C(62)-H(62C)	0.9800
C(63)-C(68)	1.517(6)
C(63)-C(64)	1.536(4)
C(63)-H(63)	1.0000
C(64)-C(65)	1.532(5)
C(64)-H(64)	1.0000
C(65)-C(66)	1.508(5)
C(65)-H(65A)	0.9900
C(65)-H(65B)	0.9900
C(66)-C(67)	1.524(5)
C(66)-H(66A)	0.9900
C(66)-H(66B)	0.9900
C(67)-C(68)	1.537(7)
C(67)-H(67A)	0.9900
C(67)-H(67B)	0.9900
C(68)-H(68A)	0.9900
C(68)-H(68B)	0.9900
C(69)-H(69A)	0.9800
C(69)-H(69B)	0.9800
C(69)-H(69C)	0.9800
C(70)-H(70A)	0.9800
C(70)-H(70B)	0.9800
C(70)-H(70C)	0.9800
C(71)-H(71A)	0.9800
C(71)-H(71B)	0.9800
C(71)-H(71C)	0.9800
C(72)-H(72A)	0.9800
C(72)-H(72B)	0.9800
C(72)-H(72C)	0.9800
C(73)-H(73A)	0.9800
C(73)-H(73B)	0.9800
C(73)-H(73C)	0.9800
C(74)-H(74A)	0.9800
C(74)-H(74B)	0.9800
C(74)-H(74C)	0.9800
C(75)-C(80)	1.532(5)
C(75)-C(76)	1.545(4)
C(75)-H(75)	1.0000

C(76)-C(77)	1.517(5)
C(76)-H(76)	1.0000
C(77)-C(78)	1.520(6)
C(77)-H(77A)	0.9900
C(77)-H(77B)	0.9900
C(78)-C(79)	1.525(4)
C(78)-H(78A)	0.9900
C(78)-H(78B)	0.9900
C(79)-C(80)	1.505(5)
C(79)-H(79A)	0.9900
C(79)-H(79B)	0.9900
C(80)-H(80A)	0.9900
C(80)-H(80B)	0.9900
C(81)-H(81A)	0.9800
C(81)-H(81B)	0.9800
C(81)-H(81C)	0.9800
C(82)-H(82A)	0.9800
C(82)-H(82B)	0.9800
C(82)-H(82C)	0.9800
C(83)-H(83A)	0.9800
C(83)-H(83B)	0.9800
C(83)-H(83C)	0.9800
C(84)-H(84A)	0.9800
C(84)-H(84B)	0.9800
C(84)-H(84C)	0.9800
C(85)-C(86)	1.532(4)
C(85)-C(90)	1.543(6)
C(85)-H(85)	1.0000
C(86)-C(87)	1.535(5)
C(86)-H(86)	1.0000
C(87)-C(88)	1.525(5)
C(87)-H(87A)	0.9900
C(87)-H(87B)	0.9900
C(88)-C(89)	1.518(4)
C(88)-H(88A)	0.9900
C(88)-H(88B)	0.9900
C(89)-C(90)	1.532(5)
C(89)-H(89A)	0.9900
C(89)-H(89B)	0.9900
C(90)-H(90A)	0.9900

C(90)-H(90B)	0.9900
C(91)-H(91A)	0.9800
C(91)-H(91B)	0.9800
C(91)-H(91C)	0.9800
C(92)-H(92A)	0.9800
C(92)-H(92B)	0.9800
C(92)-H(92C)	0.9800
C(93)-H(93A)	0.9800
C(93)-H(93B)	0.9800
C(93)-H(93C)	0.9800
C(94)-H(94A)	0.9800
C(94)-H(94B)	0.9800
C(94)-H(94C)	0.9800
C(95)-C(96)	1.521(4)
C(95)-C(100)	1.531(5)
C(95)-H(95)	1.0000
C(96)-C(97)	1.558(5)
C(96)-H(96)	1.0000
C(97)-C(98)	1.506(6)
C(97)-H(97A)	0.9900
C(97)-H(97B)	0.9900
C(98)-C(99)	1.498(5)
C(98)-H(98A)	0.9900
C(98)-H(98B)	0.9900
C(99)-C(100)	1.525(5)
C(99)-H(99A)	0.9900
C(99)-H(99B)	0.9900
C(100)-H(100)	0.9900
C(100)-H(101)	0.9900

N(1)-Li(1)-N(5)	163.3(3)
N(1)-Li(1)-Cl(1)	98.7(3)
N(5)-Li(1)-Cl(1)	98.0(3)
N(1)-Li(1)-Li(5)	149.0(3)
N(5)-Li(1)-Li(5)	46.3(2)
Cl(1)-Li(1)-Li(5)	54.0(2)
N(1)-Li(1)-Li(2)	46.2(2)
N(5)-Li(1)-Li(2)	149.6(3)
Cl(1)-Li(1)-Li(2)	54.8(2)
Li(5)-Li(1)-Li(2)	108.8(3)

N(1)-Li(1)-Si(9)	138.0(3)
N(5)-Li(1)-Si(9)	36.10(13)
Cl(1)-Li(1)-Si(9)	104.0(2)
Li(5)-Li(1)-Si(9)	69.9(2)
Li(2)-Li(1)-Si(9)	129.7(3)
N(1)-Li(1)-Si(2)	35.76(14)
N(5)-Li(1)-Si(2)	137.2(3)
Cl(1)-Li(1)-Si(2)	104.5(2)
Li(5)-Li(1)-Si(2)	128.8(2)
Li(2)-Li(1)-Si(2)	69.5(2)
Si(9)-Li(1)-Si(2)	151.5(2)
N(2)-Li(2)-N(1)	161.4(4)
N(2)-Li(2)-Cl(1)	97.5(3)
N(1)-Li(2)-Cl(1)	98.2(3)
N(2)-Li(2)-Li(1)	151.3(4)
N(1)-Li(2)-Li(1)	46.2(2)
Cl(1)-Li(2)-Li(1)	54.2(2)
N(2)-Li(2)-Li(3)	44.8(2)
N(1)-Li(2)-Li(3)	147.5(4)
Cl(1)-Li(2)-Li(3)	53.1(2)
Li(1)-Li(2)-Li(3)	107.3(3)
N(2)-Li(2)-Si(4)	34.30(15)
N(1)-Li(2)-Si(4)	128.1(3)
Cl(1)-Li(2)-Si(4)	114.9(2)
Li(1)-Li(2)-Si(4)	155.3(3)
Li(3)-Li(2)-Si(4)	65.9(2)
N(2)-Li(2)-Si(1)	137.4(4)
N(1)-Li(2)-Si(1)	33.63(15)
Cl(1)-Li(2)-Si(1)	122.8(3)
Li(1)-Li(2)-Si(1)	69.2(2)
Li(3)-Li(2)-Si(1)	172.4(3)
Si(4)-Li(2)-Si(1)	120.2(3)
N(2)-Li(3)-N(3)	161.8(4)
N(2)-Li(3)-Cl(1)	99.3(3)
N(3)-Li(3)-Cl(1)	98.8(3)
N(2)-Li(3)-Li(4)	152.6(4)
N(3)-Li(3)-Li(4)	45.5(2)
Cl(1)-Li(3)-Li(4)	53.3(2)
N(2)-Li(3)-Li(2)	45.2(2)
N(3)-Li(3)-Li(2)	152.2(4)

Cl(1)-Li(3)-Li(2)	54.5(2)
Li(4)-Li(3)-Li(2)	107.5(3)
N(2)-Li(3)-Si(3)	32.91(14)
N(3)-Li(3)-Si(3)	139.0(3)
Cl(1)-Li(3)-Si(3)	108.8(2)
Li(4)-Li(3)-Si(3)	146.5(3)
Li(2)-Li(3)-Si(3)	65.9(2)
N(2)-Li(3)-Si(6)	135.2(3)
N(3)-Li(3)-Si(6)	32.86(13)
Cl(1)-Li(3)-Si(6)	111.1(3)
Li(4)-Li(3)-Si(6)	65.8(2)
Li(2)-Li(3)-Si(6)	142.0(3)
Si(3)-Li(3)-Si(6)	140.0(3)
N(2)-Li(3)-Si(5)	142.2(3)
N(3)-Li(3)-Si(5)	29.33(13)
Cl(1)-Li(3)-Si(5)	107.6(3)
Li(4)-Li(3)-Si(5)	59.9(2)
Li(2)-Li(3)-Si(5)	154.6(3)
Si(3)-Li(3)-Si(5)	111.2(2)
Si(6)-Li(3)-Si(5)	56.82(12)
N(2)-Li(3)-Si(4)	28.94(13)
N(3)-Li(3)-Si(4)	139.4(3)
Cl(1)-Li(3)-Si(4)	108.8(2)
Li(4)-Li(3)-Si(4)	149.4(3)
Li(2)-Li(3)-Si(4)	58.0(2)
Si(3)-Li(3)-Si(4)	57.18(14)
Si(6)-Li(3)-Si(4)	107.6(2)
Si(5)-Li(3)-Si(4)	143.6(3)
N(4)-Li(4)-N(3)	160.8(4)
N(4)-Li(4)-Cl(1)	100.0(3)
N(3)-Li(4)-Cl(1)	99.2(3)
N(4)-Li(4)-Li(5)	45.8(2)
N(3)-Li(4)-Li(5)	153.4(4)
Cl(1)-Li(4)-Li(5)	54.2(2)
N(4)-Li(4)-Li(3)	153.7(3)
N(3)-Li(4)-Li(3)	45.5(2)
Cl(1)-Li(4)-Li(3)	53.7(2)
Li(5)-Li(4)-Li(3)	107.9(3)
N(4)-Li(4)-Si(8)	34.22(15)
N(3)-Li(4)-Si(8)	133.7(3)

Cl(1)-Li(4)-Si(8)	115.5(2)
Li(5)-Li(4)-Si(8)	68.4(2)
Li(3)-Li(4)-Si(8)	152.5(3)
N(4)-Li(4)-Si(5)	135.0(3)
N(3)-Li(4)-Si(5)	32.60(13)
Cl(1)-Li(4)-Si(5)	112.3(3)
Li(5)-Li(4)-Si(5)	149.4(3)
Li(3)-Li(4)-Si(5)	64.8(2)
Si(8)-Li(4)-Si(5)	132.2(2)
N(4)-Li(5)-N(5)	160.0(5)
N(4)-Li(5)-Cl(1)	98.8(3)
N(5)-Li(5)-Cl(1)	99.2(3)
N(4)-Li(5)-Li(1)	152.6(4)
N(5)-Li(5)-Li(1)	47.0(2)
Cl(1)-Li(5)-Li(1)	54.6(2)
N(4)-Li(5)-Li(4)	45.3(2)
N(5)-Li(5)-Li(4)	151.6(4)
Cl(1)-Li(5)-Li(4)	53.5(2)
Li(1)-Li(5)-Li(4)	107.8(3)
N(4)-Li(5)-Si(7)	34.96(15)
N(5)-Li(5)-Si(7)	127.6(3)
Cl(1)-Li(5)-Si(7)	113.5(2)
Li(1)-Li(5)-Si(7)	154.4(3)
Li(4)-Li(5)-Si(7)	67.7(2)
N(4)-Li(5)-Si(10)	135.2(4)
N(5)-Li(5)-Si(10)	33.00(15)
Cl(1)-Li(5)-Si(10)	123.4(3)
Li(1)-Li(5)-Si(10)	69.3(2)
Li(4)-Li(5)-Si(10)	166.7(3)
Si(7)-Li(5)-Si(10)	120.8(3)
N(10)-Li(6)-N(6)	160.3(5)
N(10)-Li(6)-Cl(2)	99.5(4)
N(6)-Li(6)-Cl(2)	98.5(3)
N(10)-Li(6)-Li(10)	47.1(2)
N(6)-Li(6)-Li(10)	152.3(4)
Cl(2)-Li(6)-Li(10)	54.5(2)
N(10)-Li(6)-Li(7)	151.9(5)
N(6)-Li(6)-Li(7)	45.3(2)
Cl(2)-Li(6)-Li(7)	53.3(2)
Li(10)-Li(6)-Li(7)	107.4(4)

N(10)-Li(6)-Si(11)	128.2(3)
N(6)-Li(6)-Si(11)	35.03(15)
Cl(2)-Li(6)-Si(11)	112.5(2)
Li(10)-Li(6)-Si(11)	153.3(3)
Li(7)-Li(6)-Si(11)	67.4(2)
N(10)-Li(6)-Si(20)	33.64(15)
N(6)-Li(6)-Si(20)	134.2(4)
Cl(2)-Li(6)-Si(20)	124.5(3)
Li(10)-Li(6)-Si(20)	70.7(2)
Li(7)-Li(6)-Si(20)	166.1(3)
Si(11)-Li(6)-Si(20)	120.7(3)
N(7)-Li(7)-N(6)	160.0(4)
N(7)-Li(7)-Cl(2)	100.6(4)
N(6)-Li(7)-Cl(2)	99.3(3)
N(7)-Li(7)-Li(8)	46.5(2)
N(6)-Li(7)-Li(8)	153.5(4)
Cl(2)-Li(7)-Li(8)	54.2(2)
N(7)-Li(7)-Li(6)	154.4(4)
N(6)-Li(7)-Li(6)	45.6(2)
Cl(2)-Li(7)-Li(6)	53.7(2)
Li(8)-Li(7)-Li(6)	107.9(3)
N(7)-Li(7)-Si(12)	132.9(3)
N(6)-Li(7)-Si(12)	34.00(15)
Cl(2)-Li(7)-Si(12)	115.1(3)
Li(8)-Li(7)-Si(12)	152.3(3)
Li(6)-Li(7)-Si(12)	68.0(2)
N(7)-Li(7)-Si(13)	32.69(14)
N(6)-Li(7)-Si(13)	134.6(3)
Cl(2)-Li(7)-Si(13)	113.9(3)
Li(8)-Li(7)-Si(13)	66.0(2)
Li(6)-Li(7)-Si(13)	150.6(3)
Si(12)-Li(7)-Si(13)	131.0(3)
N(8)-Li(8)-N(7)	161.8(4)
N(8)-Li(8)-Cl(2)	99.1(3)
N(7)-Li(8)-Cl(2)	98.9(4)
N(8)-Li(8)-Li(7)	152.6(4)
N(7)-Li(8)-Li(7)	45.5(3)
Cl(2)-Li(8)-Li(7)	53.5(2)
N(8)-Li(8)-Li(9)	45.7(2)
N(7)-Li(8)-Li(9)	151.4(4)

Cl(2)-Li(8)-Li(9)	53.8(2)
Li(7)-Li(8)-Li(9)	107.0(3)
N(8)-Li(8)-Si(15)	33.85(16)
N(7)-Li(8)-Si(15)	138.5(3)
Cl(2)-Li(8)-Si(15)	109.1(2)
Li(7)-Li(8)-Si(15)	146.6(3)
Li(9)-Li(8)-Si(15)	67.3(2)
N(8)-Li(8)-Si(14)	134.3(3)
N(7)-Li(8)-Si(14)	33.48(13)
Cl(2)-Li(8)-Si(14)	111.4(3)
Li(7)-Li(8)-Si(14)	66.1(2)
Li(9)-Li(8)-Si(14)	140.8(3)
Si(15)-Li(8)-Si(14)	139.5(3)
N(8)-Li(8)-Si(16)	28.60(15)
N(7)-Li(8)-Si(16)	139.4(3)
Cl(2)-Li(8)-Si(16)	108.6(2)
Li(7)-Li(8)-Si(16)	149.2(3)
Li(9)-Li(8)-Si(16)	58.2(2)
Si(15)-Li(8)-Si(16)	57.61(17)
Si(14)-Li(8)-Si(16)	106.9(2)
N(8)-Li(9)-N(9)	160.3(4)
N(8)-Li(9)-Cl(2)	98.2(3)
N(9)-Li(9)-Cl(2)	99.0(3)
N(8)-Li(9)-Li(10)	152.2(4)
N(9)-Li(9)-Li(10)	46.7(2)
Cl(2)-Li(9)-Li(10)	54.5(2)
N(8)-Li(9)-Li(8)	45.0(2)
N(9)-Li(9)-Li(8)	148.8(4)
Cl(2)-Li(9)-Li(8)	53.7(2)
Li(10)-Li(9)-Li(8)	108.2(4)
N(8)-Li(9)-Si(16)	34.01(14)
N(9)-Li(9)-Si(16)	127.7(3)
Cl(2)-Li(9)-Si(16)	115.8(2)
Li(10)-Li(9)-Si(16)	156.4(3)
Li(8)-Li(9)-Si(16)	66.0(2)
N(8)-Li(9)-Si(17)	135.7(4)
N(9)-Li(9)-Si(17)	33.69(15)
Cl(2)-Li(9)-Si(17)	123.6(3)
Li(10)-Li(9)-Si(17)	69.8(2)
Li(8)-Li(9)-Si(17)	172.7(3)

Si(16)-Li(9)-Si(17)	118.8(3)
N(9)-Li(10)-N(10)	161.8(4)
N(9)-Li(10)-Cl(2)	99.4(3)
N(10)-Li(10)-Cl(2)	98.6(3)
N(9)-Li(10)-Li(9)	46.9(2)
N(10)-Li(10)-Li(9)	151.1(3)
Cl(2)-Li(10)-Li(9)	54.7(2)
N(9)-Li(10)-Li(6)	149.2(3)
N(10)-Li(10)-Li(6)	46.4(2)
Cl(2)-Li(10)-Li(6)	54.3(2)
Li(9)-Li(10)-Li(6)	109.0(3)
N(9)-Li(10)-Si(19)	138.5(3)
N(10)-Li(10)-Si(19)	36.22(14)
Cl(2)-Li(10)-Si(19)	105.6(2)
Li(9)-Li(10)-Si(19)	132.5(3)
Li(6)-Li(10)-Si(19)	70.4(2)
N(9)-Li(10)-Si(18)	35.89(15)
N(10)-Li(10)-Si(18)	134.5(3)
Cl(2)-Li(10)-Si(18)	105.0(2)
Li(9)-Li(10)-Si(18)	70.0(2)
Li(6)-Li(10)-Si(18)	127.8(3)
Si(19)-Li(10)-Si(18)	149.3(2)
N(13)-Li(11)-N(11)	130.8(3)
N(13)-Li(11)-N(14)	83.8(3)
N(11)-Li(11)-N(14)	117.8(3)
N(13)-Li(11)-N(12)	116.9(3)
N(11)-Li(11)-N(12)	83.1(3)
N(14)-Li(11)-N(12)	130.9(3)
N(16)-Li(12)-N(17)	127.3(3)
N(16)-Li(12)-N(15)	83.6(3)
N(17)-Li(12)-N(15)	118.1(3)
N(16)-Li(12)-N(18)	118.6(3)
N(17)-Li(12)-N(18)	83.5(3)
N(15)-Li(12)-N(18)	131.7(3)
Li(4)-Cl(1)-Li(3)	72.9(2)
Li(4)-Cl(1)-Li(5)	72.3(2)
Li(3)-Cl(1)-Li(5)	145.1(2)
Li(4)-Cl(1)-Li(1)	143.0(3)
Li(3)-Cl(1)-Li(1)	143.4(3)
Li(5)-Cl(1)-Li(1)	71.4(3)

Li(4)-Cl(1)-Li(2)	144.7(3)
Li(3)-Cl(1)-Li(2)	72.4(2)
Li(5)-Cl(1)-Li(2)	142.4(2)
Li(1)-Cl(1)-Li(2)	71.0(3)
Li(7)-Cl(2)-Li(6)	73.0(3)
Li(7)-Cl(2)-Li(10)	143.5(3)
Li(6)-Cl(2)-Li(10)	71.2(3)
Li(7)-Cl(2)-Li(8)	72.3(3)
Li(6)-Cl(2)-Li(8)	145.3(3)
Li(10)-Cl(2)-Li(8)	143.3(3)
Li(7)-Cl(2)-Li(9)	144.0(2)
Li(6)-Cl(2)-Li(9)	142.0(2)
Li(10)-Cl(2)-Li(9)	70.8(3)
Li(8)-Cl(2)-Li(9)	72.5(3)
N(1)-Si(1)-C(1)	108.99(18)
N(1)-Si(1)-C(2)	114.0(2)
C(1)-Si(1)-C(2)	107.2(2)
N(1)-Si(1)-C(3)	116.04(18)
C(1)-Si(1)-C(3)	104.8(2)
C(2)-Si(1)-C(3)	105.2(2)
C(1)-Si(1)-Li(2)	68.63(19)
C(2)-Si(1)-Li(2)	136.6(2)
C(3)-Si(1)-Li(2)	117.8(2)
N(1)-Si(2)-C(4)	116.9(2)
N(1)-Si(2)-C(6)	109.34(17)
C(4)-Si(2)-C(6)	103.2(2)
N(1)-Si(2)-C(5)	113.83(16)
C(4)-Si(2)-C(5)	106.15(19)
C(6)-Si(2)-C(5)	106.5(2)
C(4)-Si(2)-Li(1)	147.91(18)
C(6)-Si(2)-Li(1)	70.08(19)
C(5)-Si(2)-Li(1)	105.81(19)
N(2)-Si(3)-C(9)	116.01(19)
N(2)-Si(3)-C(8)	112.00(17)
C(9)-Si(3)-C(8)	105.0(2)
N(2)-Si(3)-C(7)	109.7(2)
C(9)-Si(3)-C(7)	106.7(2)
C(8)-Si(3)-C(7)	107.0(2)
C(9)-Si(3)-Li(3)	156.20(19)
C(8)-Si(3)-Li(3)	87.3(2)

C(7)-Si(3)-Li(3)	88.5(2)
N(2)-Si(4)-C(12)	110.0(2)
N(2)-Si(4)-C(11)	112.51(17)
C(12)-Si(4)-C(11)	105.7(2)
N(2)-Si(4)-C(10)	114.86(18)
C(12)-Si(4)-C(10)	107.3(2)
C(11)-Si(4)-C(10)	105.9(3)
C(12)-Si(4)-Li(2)	89.7(2)
C(11)-Si(4)-Li(2)	83.8(2)
C(10)-Si(4)-Li(2)	156.68(19)
C(12)-Si(4)-Li(3)	79.2(2)
C(11)-Si(4)-Li(3)	139.8(2)
C(10)-Si(4)-Li(3)	110.6(2)
Li(2)-Si(4)-Li(3)	56.06(19)
N(3)-Si(5)-C(15)	116.2(2)
N(3)-Si(5)-C(13)	110.97(16)
C(15)-Si(5)-C(13)	105.7(2)
N(3)-Si(5)-C(14)	112.0(2)
C(15)-Si(5)-C(14)	105.9(2)
C(13)-Si(5)-C(14)	105.3(2)
C(15)-Si(5)-Li(4)	155.94(19)
C(13)-Si(5)-Li(4)	91.73(19)
C(14)-Si(5)-Li(4)	84.7(2)
C(15)-Si(5)-Li(3)	111.0(2)
C(13)-Si(5)-Li(3)	79.39(18)
C(14)-Si(5)-Li(3)	139.9(2)
Li(4)-Si(5)-Li(3)	55.22(19)
N(3)-Si(6)-C(18)	108.97(17)
N(3)-Si(6)-C(17)	112.0(2)
C(18)-Si(6)-C(17)	108.7(2)
N(3)-Si(6)-C(16)	116.63(19)
C(18)-Si(6)-C(16)	106.3(2)
C(17)-Si(6)-C(16)	103.8(2)
C(18)-Si(6)-Li(3)	89.93(19)
C(17)-Si(6)-Li(3)	86.2(2)
C(16)-Si(6)-Li(3)	156.49(19)
N(4)-Si(7)-C(19)	109.5(2)
N(4)-Si(7)-C(20)	117.07(17)
C(19)-Si(7)-C(20)	105.8(2)
N(4)-Si(7)-C(21)	111.75(17)

C(19)-Si(7)-C(21)	106.57(18)
C(20)-Si(7)-C(21)	105.5(2)
C(19)-Si(7)-Li(5)	86.9(2)
C(20)-Si(7)-Li(5)	160.26(18)
C(21)-Si(7)-Li(5)	84.5(2)
N(4)-Si(8)-C(23)	110.88(18)
N(4)-Si(8)-C(22)	116.57(18)
C(23)-Si(8)-C(22)	104.7(2)
N(4)-Si(8)-C(24)	109.20(19)
C(23)-Si(8)-C(24)	108.07(18)
C(22)-Si(8)-C(24)	107.0(2)
C(23)-Si(8)-Li(4)	84.0(2)
C(22)-Si(8)-Li(4)	157.83(18)
C(24)-Si(8)-Li(4)	89.0(2)
N(5)-Si(9)-C(25)	115.8(2)
N(5)-Si(9)-C(26)	110.10(17)
C(25)-Si(9)-C(26)	105.8(2)
N(5)-Si(9)-C(27)	112.41(17)
C(25)-Si(9)-C(27)	105.90(19)
C(26)-Si(9)-C(27)	106.2(2)
N(5)-Si(9)-Li(1)	45.8(2)
C(25)-Si(9)-Li(1)	149.32(18)
C(26)-Si(9)-Li(1)	69.8(2)
C(27)-Si(9)-Li(1)	104.39(19)
N(5)-Si(10)-C(29)	114.3(2)
N(5)-Si(10)-C(30)	109.75(18)
C(29)-Si(10)-C(30)	105.44(18)
N(5)-Si(10)-C(28)	117.01(18)
C(29)-Si(10)-C(28)	105.6(2)
C(30)-Si(10)-C(28)	103.6(2)
C(29)-Si(10)-Li(5)	133.6(2)
C(30)-Si(10)-Li(5)	69.07(19)
C(28)-Si(10)-Li(5)	120.7(2)
N(6)-Si(11)-C(32)	110.90(17)
N(6)-Si(11)-C(33)	116.43(17)
C(32)-Si(11)-C(33)	105.6(2)
N(6)-Si(11)-C(31)	108.8(2)
C(32)-Si(11)-C(31)	108.25(19)
C(33)-Si(11)-C(31)	106.5(2)
C(32)-Si(11)-Li(6)	83.9(2)

C(33)-Si(11)-Li(6)	160.40(18)
C(31)-Si(11)-Li(6)	86.0(2)
N(6)-Si(12)-C(35)	110.9(2)
N(6)-Si(12)-C(34)	109.87(18)
C(35)-Si(12)-C(34)	107.37(18)
N(6)-Si(12)-C(36)	116.30(18)
C(35)-Si(12)-C(36)	105.6(2)
C(34)-Si(12)-C(36)	106.4(2)
C(35)-Si(12)-Li(7)	90.8(2)
C(34)-Si(12)-Li(7)	82.2(2)
C(36)-Si(12)-Li(7)	157.74(19)
N(7)-Si(13)-C(38)	112.06(17)
N(7)-Si(13)-C(39)	111.8(2)
C(38)-Si(13)-C(39)	106.0(2)
N(7)-Si(13)-C(37)	115.0(2)
C(38)-Si(13)-C(37)	105.2(2)
C(39)-Si(13)-C(37)	106.0(2)
C(38)-Si(13)-Li(7)	93.04(19)
C(39)-Si(13)-Li(7)	84.9(2)
C(37)-Si(13)-Li(7)	154.8(2)
N(7)-Si(14)-C(41)	109.99(17)
N(7)-Si(14)-C(40)	112.2(2)
C(41)-Si(14)-C(40)	109.0(2)
N(7)-Si(14)-C(42)	116.47(19)
C(41)-Si(14)-C(42)	104.4(2)
C(40)-Si(14)-C(42)	104.2(2)
C(41)-Si(14)-Li(8)	90.16(18)
C(40)-Si(14)-Li(8)	85.4(2)
C(42)-Si(14)-Li(8)	158.5(2)
N(8)-Si(15)-C(44)	110.4(2)
N(8)-Si(15)-C(43)	112.51(17)
C(44)-Si(15)-C(43)	107.7(2)
N(8)-Si(15)-C(45)	116.1(2)
C(44)-Si(15)-C(45)	106.2(2)
C(43)-Si(15)-C(45)	103.3(2)
C(44)-Si(15)-Li(8)	88.4(2)
C(43)-Si(15)-Li(8)	87.7(2)
C(45)-Si(15)-Li(8)	157.6(2)
N(8)-Si(16)-C(48)	110.5(2)
N(8)-Si(16)-C(46)	111.44(17)

C(48)-Si(16)-C(46)	105.4(2)
N(8)-Si(16)-C(47)	115.86(18)
C(48)-Si(16)-C(47)	107.13(19)
C(46)-Si(16)-C(47)	105.9(2)
C(48)-Si(16)-Li(9)	89.4(2)
C(46)-Si(16)-Li(9)	83.0(2)
C(47)-Si(16)-Li(9)	157.86(19)
C(48)-Si(16)-Li(8)	80.0(2)
C(46)-Si(16)-Li(8)	138.61(19)
C(47)-Si(16)-Li(8)	111.6(2)
Li(9)-Si(16)-Li(8)	55.82(18)
N(9)-Si(17)-C(51)	114.0(2)
N(9)-Si(17)-C(49)	109.63(18)
C(51)-Si(17)-C(49)	106.1(2)
N(9)-Si(17)-C(50)	116.40(18)
C(51)-Si(17)-C(50)	104.9(2)
C(49)-Si(17)-C(50)	104.9(2)
C(51)-Si(17)-Li(9)	136.2(2)
C(49)-Si(17)-Li(9)	68.56(19)
C(50)-Si(17)-Li(9)	118.62(19)
N(9)-Si(18)-C(54)	110.07(18)
N(9)-Si(18)-C(53)	112.41(17)
C(54)-Si(18)-C(53)	106.1(2)
N(9)-Si(18)-C(52)	115.0(2)
C(54)-Si(18)-C(52)	106.0(2)
C(53)-Si(18)-C(52)	106.7(2)
N(9)-Si(18)-Li(10)	45.1(2)
C(54)-Si(18)-Li(10)	70.0(2)
C(53)-Si(18)-Li(10)	105.3(2)
C(52)-Si(18)-Li(10)	147.48(19)
N(10)-Si(19)-C(55)	110.32(17)
N(10)-Si(19)-C(56)	112.66(16)
C(55)-Si(19)-C(56)	106.8(2)
N(10)-Si(19)-C(57)	115.7(2)
C(55)-Si(19)-C(57)	104.8(2)
C(56)-Si(19)-C(57)	105.88(19)
N(10)-Si(19)-Li(10)	46.2(2)
C(55)-Si(19)-Li(10)	69.8(2)
C(56)-Si(19)-Li(10)	104.9(2)
C(57)-Si(19)-Li(10)	148.96(18)

N(10)-Si(20)-C(59)	115.86(17)
N(10)-Si(20)-C(60)	110.16(17)
C(59)-Si(20)-C(60)	104.7(2)
N(10)-Si(20)-C(58)	114.4(2)
C(59)-Si(20)-C(58)	104.8(2)
C(60)-Si(20)-C(58)	106.02(18)
C(59)-Si(20)-Li(6)	118.9(2)
C(60)-Si(20)-Li(6)	69.45(19)
C(58)-Si(20)-Li(6)	135.9(2)
Si(2)-N(1)-Si(1)	119.07(19)
Si(2)-N(1)-Li(1)	99.4(3)
Si(1)-N(1)-Li(1)	122.9(2)
Si(2)-N(1)-Li(2)	120.0(2)
Si(1)-N(1)-Li(2)	104.8(3)
Li(1)-N(1)-Li(2)	87.5(3)
Si(4)-N(2)-Si(3)	120.4(2)
Si(4)-N(2)-Li(3)	115.9(2)
Si(3)-N(2)-Li(3)	106.8(2)
Si(4)-N(2)-Li(2)	103.2(2)
Si(3)-N(2)-Li(2)	116.9(2)
Li(3)-N(2)-Li(2)	89.9(3)
Si(5)-N(3)-Si(6)	119.10(16)
Si(5)-N(3)-Li(3)	114.6(3)
Si(6)-N(3)-Li(3)	106.8(3)
Si(5)-N(3)-Li(4)	107.0(3)
Si(6)-N(3)-Li(4)	116.6(3)
Li(3)-N(3)-Li(4)	89.0(3)
Si(8)-N(4)-Si(7)	120.1(2)
Si(8)-N(4)-Li(4)	103.9(2)
Si(7)-N(4)-Li(4)	118.6(3)
Si(8)-N(4)-Li(5)	120.2(2)
Si(7)-N(4)-Li(5)	101.7(2)
Li(4)-N(4)-Li(5)	88.9(3)
Si(10)-N(5)-Si(9)	119.53(18)
Si(10)-N(5)-Li(5)	105.6(3)
Si(9)-N(5)-Li(5)	120.5(2)
Si(10)-N(5)-Li(1)	122.8(2)
Si(9)-N(5)-Li(1)	98.1(2)
Li(5)-N(5)-Li(1)	86.7(3)
Si(11)-N(6)-Si(12)	121.3(2)

Si(11)-N(6)-Li(7)	117.6(3)
Si(12)-N(6)-Li(7)	103.8(2)
Si(11)-N(6)-Li(6)	100.8(2)
Si(12)-N(6)-Li(6)	120.3(2)
Li(7)-N(6)-Li(6)	89.1(3)
Si(14)-N(7)-Si(13)	120.72(16)
Si(14)-N(7)-Li(7)	117.0(3)
Si(13)-N(7)-Li(7)	106.9(3)
Si(14)-N(7)-Li(8)	104.1(3)
Si(13)-N(7)-Li(8)	115.6(3)
Li(7)-N(7)-Li(8)	88.0(3)
Si(16)-N(8)-Si(15)	121.2(2)
Si(16)-N(8)-Li(8)	116.4(2)
Si(15)-N(8)-Li(8)	104.4(3)
Si(16)-N(8)-Li(9)	103.3(2)
Si(15)-N(8)-Li(9)	118.2(2)
Li(8)-N(8)-Li(9)	89.3(3)
Si(17)-N(9)-Si(18)	120.31(19)
Si(17)-N(9)-Li(10)	123.6(2)
Si(18)-N(9)-Li(10)	99.0(3)
Si(17)-N(9)-Li(9)	104.0(3)
Si(18)-N(9)-Li(9)	119.8(2)
Li(10)-N(9)-Li(9)	86.3(3)
Si(19)-N(10)-Si(20)	118.76(18)
Si(19)-N(10)-Li(6)	121.4(2)
Si(20)-N(10)-Li(6)	104.6(3)
Si(19)-N(10)-Li(10)	97.5(2)
Si(20)-N(10)-Li(10)	125.1(2)
Li(6)-N(10)-Li(10)	86.5(3)
C(62)-N(11)-C(61)	107.7(3)
C(62)-N(11)-C(63)	110.8(3)
C(61)-N(11)-C(63)	112.2(4)
C(62)-N(11)-Li(11)	115.6(4)
C(61)-N(11)-Li(11)	105.6(3)
C(63)-N(11)-Li(11)	105.1(3)
C(70)-N(12)-C(69)	108.0(3)
C(70)-N(12)-C(64)	109.7(3)
C(69)-N(12)-C(64)	112.1(3)
C(70)-N(12)-Li(11)	117.8(3)
C(69)-N(12)-Li(11)	100.4(3)

C(64)-N(12)-Li(11)	108.6(3)
C(71)-N(13)-C(72)	108.4(3)
C(71)-N(13)-C(75)	109.6(3)
C(72)-N(13)-C(75)	113.3(3)
C(71)-N(13)-Li(11)	115.9(3)
C(72)-N(13)-Li(11)	101.0(3)
C(75)-N(13)-Li(11)	108.5(3)
C(74)-N(14)-C(73)	107.8(3)
C(74)-N(14)-C(76)	112.1(3)
C(73)-N(14)-C(76)	109.1(3)
C(74)-N(14)-Li(11)	106.0(3)
C(73)-N(14)-Li(11)	115.1(3)
C(76)-N(14)-Li(11)	106.9(3)
C(82)-N(15)-C(81)	107.9(3)
C(82)-N(15)-C(85)	112.9(3)
C(81)-N(15)-C(85)	109.9(3)
C(82)-N(15)-Li(12)	107.2(3)
C(81)-N(15)-Li(12)	113.6(3)
C(85)-N(15)-Li(12)	105.4(3)
C(86)-N(16)-C(84)	110.1(3)
C(86)-N(16)-C(83)	113.4(3)
C(84)-N(16)-C(83)	108.1(3)
C(86)-N(16)-Li(12)	109.4(3)
C(84)-N(16)-Li(12)	111.5(3)
C(83)-N(16)-Li(12)	104.3(3)
C(91)-N(17)-C(92)	108.0(3)
C(91)-N(17)-C(95)	111.3(3)
C(92)-N(17)-C(95)	112.6(3)
C(91)-N(17)-Li(12)	115.2(3)
C(92)-N(17)-Li(12)	108.3(3)
C(95)-N(17)-Li(12)	101.5(3)
C(96)-N(18)-C(94)	109.7(3)
C(96)-N(18)-C(93)	114.0(3)
C(94)-N(18)-C(93)	107.3(3)
C(96)-N(18)-Li(12)	106.2(3)
C(94)-N(18)-Li(12)	119.9(3)
C(93)-N(18)-Li(12)	99.7(3)
Si(1)-C(1)-H(1A)	109.5
Si(1)-C(1)-H(1B)	109.5
H(1A)-C(1)-H(1B)	109.5

Si(1)-C(1)-H(1C)	109.5
H(1A)-C(1)-H(1C)	109.5
H(1B)-C(1)-H(1C)	109.5
Si(1)-C(2)-H(2A)	109.5
Si(1)-C(2)-H(2B)	109.5
H(2A)-C(2)-H(2B)	109.5
Si(1)-C(2)-H(2C)	109.5
H(2A)-C(2)-H(2C)	109.5
H(2B)-C(2)-H(2C)	109.5
Si(1)-C(3)-H(3A)	109.5
Si(1)-C(3)-H(3B)	109.5
H(3A)-C(3)-H(3B)	109.5
Si(1)-C(3)-H(3C)	109.5
H(3A)-C(3)-H(3C)	109.5
H(3B)-C(3)-H(3C)	109.5
Si(2)-C(4)-H(4A)	109.5
Si(2)-C(4)-H(4B)	109.5
H(4A)-C(4)-H(4B)	109.5
Si(2)-C(4)-H(4C)	109.5
H(4A)-C(4)-H(4C)	109.5
H(4B)-C(4)-H(4C)	109.5
Si(2)-C(5)-H(5A)	109.5
Si(2)-C(5)-H(5B)	109.5
H(5A)-C(5)-H(5B)	109.5
Si(2)-C(5)-H(5C)	109.5
H(5A)-C(5)-H(5C)	109.5
H(5B)-C(5)-H(5C)	109.5
Si(2)-C(6)-H(6A)	109.5
Si(2)-C(6)-H(6B)	109.5
H(6A)-C(6)-H(6B)	109.5
Si(2)-C(6)-H(6C)	109.5
H(6A)-C(6)-H(6C)	109.5
H(6B)-C(6)-H(6C)	109.5
Si(3)-C(7)-H(7A)	109.5
Si(3)-C(7)-H(7B)	109.5
H(7A)-C(7)-H(7B)	109.5
Si(3)-C(7)-H(7C)	109.5
H(7A)-C(7)-H(7C)	109.5
H(7B)-C(7)-H(7C)	109.5
Si(3)-C(8)-H(8A)	109.5

Si(3)-C(8)-H(8B)	109.5
H(8A)-C(8)-H(8B)	109.5
Si(3)-C(8)-H(8C)	109.5
H(8A)-C(8)-H(8C)	109.5
H(8B)-C(8)-H(8C)	109.5
Si(3)-C(9)-H(9A)	109.5
Si(3)-C(9)-H(9B)	109.5
H(9A)-C(9)-H(9B)	109.5
Si(3)-C(9)-H(9C)	109.5
H(9A)-C(9)-H(9C)	109.5
H(9B)-C(9)-H(9C)	109.5
Si(4)-C(10)-H(10A)	109.5
Si(4)-C(10)-H(10B)	109.5
H(10A)-C(10)-H(10B)	109.5
Si(4)-C(10)-H(10C)	109.5
H(10A)-C(10)-H(10C)	109.5
H(10B)-C(10)-H(10C)	109.5
Si(4)-C(11)-H(11A)	109.5
Si(4)-C(11)-H(11B)	109.5
H(11A)-C(11)-H(11B)	109.5
Si(4)-C(11)-H(11C)	109.5
H(11A)-C(11)-H(11C)	109.5
H(11B)-C(11)-H(11C)	109.5
Si(4)-C(12)-H(12A)	109.5
Si(4)-C(12)-H(12B)	109.5
H(12A)-C(12)-H(12B)	109.5
Si(4)-C(12)-H(12C)	109.5
H(12A)-C(12)-H(12C)	109.5
H(12B)-C(12)-H(12C)	109.5
Si(5)-C(13)-H(13A)	109.5
Si(5)-C(13)-H(13B)	109.5
H(13A)-C(13)-H(13B)	109.5
Si(5)-C(13)-H(13C)	109.5
H(13A)-C(13)-H(13C)	109.5
H(13B)-C(13)-H(13C)	109.5
Si(5)-C(14)-H(14A)	109.5
Si(5)-C(14)-H(14B)	109.5
H(14A)-C(14)-H(14B)	109.5
Si(5)-C(14)-H(14C)	109.5
H(14A)-C(14)-H(14C)	109.5

H(14B)-C(14)-H(14C)	109.5
Si(5)-C(15)-H(15A)	109.5
Si(5)-C(15)-H(15B)	109.5
H(15A)-C(15)-H(15B)	109.5
Si(5)-C(15)-H(15C)	109.5
H(15A)-C(15)-H(15C)	109.5
H(15B)-C(15)-H(15C)	109.5
Si(6)-C(16)-H(16A)	109.5
Si(6)-C(16)-H(16B)	109.5
H(16A)-C(16)-H(16B)	109.5
Si(6)-C(16)-H(16C)	109.5
H(16A)-C(16)-H(16C)	109.5
H(16B)-C(16)-H(16C)	109.5
Si(6)-C(17)-H(17A)	109.5
Si(6)-C(17)-H(17B)	109.5
H(17A)-C(17)-H(17B)	109.5
Si(6)-C(17)-H(17C)	109.5
H(17A)-C(17)-H(17C)	109.5
H(17B)-C(17)-H(17C)	109.5
Si(6)-C(18)-H(18A)	109.5
Si(6)-C(18)-H(18B)	109.5
H(18A)-C(18)-H(18B)	109.5
Si(6)-C(18)-H(18C)	109.5
H(18A)-C(18)-H(18C)	109.5
H(18B)-C(18)-H(18C)	109.5
Si(7)-C(19)-H(19A)	109.5
Si(7)-C(19)-H(19B)	109.5
H(19A)-C(19)-H(19B)	109.5
Si(7)-C(19)-H(19C)	109.5
H(19A)-C(19)-H(19C)	109.5
H(19B)-C(19)-H(19C)	109.5
Si(7)-C(20)-H(20A)	109.5
Si(7)-C(20)-H(20B)	109.5
H(20A)-C(20)-H(20B)	109.5
Si(7)-C(20)-H(20C)	109.5
H(20A)-C(20)-H(20C)	109.5
H(20B)-C(20)-H(20C)	109.5
Si(7)-C(21)-H(21A)	109.5
Si(7)-C(21)-H(21B)	109.5
H(21A)-C(21)-H(21B)	109.5

Si(7)-C(21)-H(21C)	109.5
H(21A)-C(21)-H(21C)	109.5
H(21B)-C(21)-H(21C)	109.5
Si(8)-C(22)-H(22A)	109.5
Si(8)-C(22)-H(22B)	109.5
H(22A)-C(22)-H(22B)	109.5
Si(8)-C(22)-H(22C)	109.5
H(22A)-C(22)-H(22C)	109.5
H(22B)-C(22)-H(22C)	109.5
Si(8)-C(23)-H(23A)	109.5
Si(8)-C(23)-H(23B)	109.5
H(23A)-C(23)-H(23B)	109.5
Si(8)-C(23)-H(23C)	109.5
H(23A)-C(23)-H(23C)	109.5
H(23B)-C(23)-H(23C)	109.5
Si(8)-C(24)-H(24A)	109.5
Si(8)-C(24)-H(24B)	109.5
H(24A)-C(24)-H(24B)	109.5
Si(8)-C(24)-H(24C)	109.5
H(24A)-C(24)-H(24C)	109.5
H(24B)-C(24)-H(24C)	109.5
Si(9)-C(25)-H(25A)	109.5
Si(9)-C(25)-H(25B)	109.5
H(25A)-C(25)-H(25B)	109.5
Si(9)-C(25)-H(25C)	109.5
H(25A)-C(25)-H(25C)	109.5
H(25B)-C(25)-H(25C)	109.5
Si(9)-C(26)-H(26A)	109.5
Si(9)-C(26)-H(26B)	109.5
H(26A)-C(26)-H(26B)	109.5
Si(9)-C(26)-H(26C)	109.5
H(26A)-C(26)-H(26C)	109.5
H(26B)-C(26)-H(26C)	109.5
Si(9)-C(27)-H(27A)	109.5
Si(9)-C(27)-H(27B)	109.5
H(27A)-C(27)-H(27B)	109.5
Si(9)-C(27)-H(27C)	109.5
H(27A)-C(27)-H(27C)	109.5
H(27B)-C(27)-H(27C)	109.5
Si(10)-C(28)-H(28A)	109.5

Si(10)-C(28)-H(28B)	109.5
H(28A)-C(28)-H(28B)	109.5
Si(10)-C(28)-H(28C)	109.5
H(28A)-C(28)-H(28C)	109.5
H(28B)-C(28)-H(28C)	109.5
Si(10)-C(29)-H(29A)	109.5
Si(10)-C(29)-H(29B)	109.5
H(29A)-C(29)-H(29B)	109.5
Si(10)-C(29)-H(29C)	109.5
H(29A)-C(29)-H(29C)	109.5
H(29B)-C(29)-H(29C)	109.5
Si(10)-C(30)-H(30A)	109.5
Si(10)-C(30)-H(30B)	109.5
H(30A)-C(30)-H(30B)	109.5
Si(10)-C(30)-H(30C)	109.5
H(30A)-C(30)-H(30C)	109.5
H(30B)-C(30)-H(30C)	109.5
Si(11)-C(31)-H(31A)	109.5
Si(11)-C(31)-H(31B)	109.5
H(31A)-C(31)-H(31B)	109.5
Si(11)-C(31)-H(31C)	109.5
H(31A)-C(31)-H(31C)	109.5
H(31B)-C(31)-H(31C)	109.5
Si(11)-C(32)-H(32A)	109.5
Si(11)-C(32)-H(32B)	109.5
H(32A)-C(32)-H(32B)	109.5
Si(11)-C(32)-H(32C)	109.5
H(32A)-C(32)-H(32C)	109.5
H(32B)-C(32)-H(32C)	109.5
Si(11)-C(33)-H(33A)	109.5
Si(11)-C(33)-H(33B)	109.5
H(33A)-C(33)-H(33B)	109.5
Si(11)-C(33)-H(33C)	109.5
H(33A)-C(33)-H(33C)	109.5
H(33B)-C(33)-H(33C)	109.5
Si(12)-C(34)-H(34A)	109.5
Si(12)-C(34)-H(34B)	109.5
H(34A)-C(34)-H(34B)	109.5
Si(12)-C(34)-H(34C)	109.5
H(34A)-C(34)-H(34C)	109.5

H(34B)-C(34)-H(34C)	109.5
Si(12)-C(35)-H(35A)	109.5
Si(12)-C(35)-H(35B)	109.5
H(35A)-C(35)-H(35B)	109.5
Si(12)-C(35)-H(35C)	109.5
H(35A)-C(35)-H(35C)	109.5
H(35B)-C(35)-H(35C)	109.5
Si(12)-C(36)-H(36A)	109.5
Si(12)-C(36)-H(36B)	109.5
H(36A)-C(36)-H(36B)	109.5
Si(12)-C(36)-H(36C)	109.5
H(36A)-C(36)-H(36C)	109.5
H(36B)-C(36)-H(36C)	109.5
Si(13)-C(37)-H(37A)	109.5
Si(13)-C(37)-H(37B)	109.5
H(37A)-C(37)-H(37B)	109.5
Si(13)-C(37)-H(37C)	109.5
H(37A)-C(37)-H(37C)	109.5
H(37B)-C(37)-H(37C)	109.5
Si(13)-C(38)-H(38A)	109.5
Si(13)-C(38)-H(38B)	109.5
H(38A)-C(38)-H(38B)	109.5
Si(13)-C(38)-H(38C)	109.5
H(38A)-C(38)-H(38C)	109.5
H(38B)-C(38)-H(38C)	109.5
Si(13)-C(39)-H(39A)	109.5
Si(13)-C(39)-H(39B)	109.5
H(39A)-C(39)-H(39B)	109.5
Si(13)-C(39)-H(39C)	109.5
H(39A)-C(39)-H(39C)	109.5
H(39B)-C(39)-H(39C)	109.5
Si(14)-C(40)-H(40A)	109.5
Si(14)-C(40)-H(40B)	109.5
H(40A)-C(40)-H(40B)	109.5
Si(14)-C(40)-H(40C)	109.5
H(40A)-C(40)-H(40C)	109.5
H(40B)-C(40)-H(40C)	109.5
Si(14)-C(41)-H(41A)	109.5
Si(14)-C(41)-H(41B)	109.5
H(41A)-C(41)-H(41B)	109.5

Si(14)-C(41)-H(41C)	109.5
H(41A)-C(41)-H(41C)	109.5
H(41B)-C(41)-H(41C)	109.5
Si(14)-C(42)-H(42A)	109.5
Si(14)-C(42)-H(42B)	109.5
H(42A)-C(42)-H(42B)	109.5
Si(14)-C(42)-H(42C)	109.5
H(42A)-C(42)-H(42C)	109.5
H(42B)-C(42)-H(42C)	109.5
Si(15)-C(43)-H(43A)	109.5
Si(15)-C(43)-H(43B)	109.5
H(43A)-C(43)-H(43B)	109.5
Si(15)-C(43)-H(43C)	109.5
H(43A)-C(43)-H(43C)	109.5
H(43B)-C(43)-H(43C)	109.5
Si(15)-C(44)-H(44A)	109.5
Si(15)-C(44)-H(44B)	109.5
H(44A)-C(44)-H(44B)	109.5
Si(15)-C(44)-H(44C)	109.5
H(44A)-C(44)-H(44C)	109.5
H(44B)-C(44)-H(44C)	109.5
Si(15)-C(45)-H(45A)	109.5
Si(15)-C(45)-H(45B)	109.5
H(45A)-C(45)-H(45B)	109.5
Si(15)-C(45)-H(45C)	109.5
H(45A)-C(45)-H(45C)	109.5
H(45B)-C(45)-H(45C)	109.5
Si(16)-C(46)-H(46A)	109.5
Si(16)-C(46)-H(46B)	109.5
H(46A)-C(46)-H(46B)	109.5
Si(16)-C(46)-H(46C)	109.5
H(46A)-C(46)-H(46C)	109.5
H(46B)-C(46)-H(46C)	109.5
Si(16)-C(47)-H(47A)	109.5
Si(16)-C(47)-H(47B)	109.5
H(47A)-C(47)-H(47B)	109.5
Si(16)-C(47)-H(47C)	109.5
H(47A)-C(47)-H(47C)	109.5
H(47B)-C(47)-H(47C)	109.5
Si(16)-C(48)-H(48A)	109.5

Si(16)-C(48)-H(48B)	109.5
H(48A)-C(48)-H(48B)	109.5
Si(16)-C(48)-H(48C)	109.5
H(48A)-C(48)-H(48C)	109.5
H(48B)-C(48)-H(48C)	109.5
Si(17)-C(49)-H(49A)	109.5
Si(17)-C(49)-H(49B)	109.5
H(49A)-C(49)-H(49B)	109.5
Si(17)-C(49)-H(49C)	109.5
H(49A)-C(49)-H(49C)	109.5
H(49B)-C(49)-H(49C)	109.5
Si(17)-C(50)-H(50A)	109.5
Si(17)-C(50)-H(50B)	109.5
H(50A)-C(50)-H(50B)	109.5
Si(17)-C(50)-H(50C)	109.5
H(50A)-C(50)-H(50C)	109.5
H(50B)-C(50)-H(50C)	109.5
Si(17)-C(51)-H(51A)	109.5
Si(17)-C(51)-H(51B)	109.5
H(51A)-C(51)-H(51B)	109.5
Si(17)-C(51)-H(51C)	109.5
H(51A)-C(51)-H(51C)	109.5
H(51B)-C(51)-H(51C)	109.5
Si(18)-C(52)-H(52A)	109.5
Si(18)-C(52)-H(52B)	109.5
H(52A)-C(52)-H(52B)	109.5
Si(18)-C(52)-H(52C)	109.5
H(52A)-C(52)-H(52C)	109.5
H(52B)-C(52)-H(52C)	109.5
Si(18)-C(53)-H(53A)	109.5
Si(18)-C(53)-H(53B)	109.5
H(53A)-C(53)-H(53B)	109.5
Si(18)-C(53)-H(53C)	109.5
H(53A)-C(53)-H(53C)	109.5
H(53B)-C(53)-H(53C)	109.5
Si(18)-C(54)-H(54A)	109.5
Si(18)-C(54)-H(54B)	109.5
H(54A)-C(54)-H(54B)	109.5
Si(18)-C(54)-H(54C)	109.5
H(54A)-C(54)-H(54C)	109.5

H(54B)-C(54)-H(54C)	109.5
Si(19)-C(55)-H(55A)	109.5
Si(19)-C(55)-H(55B)	109.5
H(55A)-C(55)-H(55B)	109.5
Si(19)-C(55)-H(55C)	109.5
H(55A)-C(55)-H(55C)	109.5
H(55B)-C(55)-H(55C)	109.5
Si(19)-C(56)-H(56A)	109.5
Si(19)-C(56)-H(56B)	109.5
H(56A)-C(56)-H(56B)	109.5
Si(19)-C(56)-H(56C)	109.5
H(56A)-C(56)-H(56C)	109.5
H(56B)-C(56)-H(56C)	109.5
Si(19)-C(57)-H(57A)	109.5
Si(19)-C(57)-H(57B)	109.5
H(57A)-C(57)-H(57B)	109.5
Si(19)-C(57)-H(57C)	109.5
H(57A)-C(57)-H(57C)	109.5
H(57B)-C(57)-H(57C)	109.5
Si(20)-C(58)-H(58A)	109.5
Si(20)-C(58)-H(58B)	109.5
H(58A)-C(58)-H(58B)	109.5
Si(20)-C(58)-H(58C)	109.5
H(58A)-C(58)-H(58C)	109.5
H(58B)-C(58)-H(58C)	109.5
Si(20)-C(59)-H(59A)	109.5
Si(20)-C(59)-H(59B)	109.5
H(59A)-C(59)-H(59B)	109.5
Si(20)-C(59)-H(59C)	109.5
H(59A)-C(59)-H(59C)	109.5
H(59B)-C(59)-H(59C)	109.5
Si(20)-C(60)-H(60A)	109.5
Si(20)-C(60)-H(60B)	109.5
H(60A)-C(60)-H(60B)	109.5
Si(20)-C(60)-H(60C)	109.5
H(60A)-C(60)-H(60C)	109.5
H(60B)-C(60)-H(60C)	109.5
N(11)-C(61)-H(61A)	109.5
N(11)-C(61)-H(61B)	109.5
H(61A)-C(61)-H(61B)	109.5

N(11)-C(61)-H(61C)	109.5
H(61A)-C(61)-H(61C)	109.5
H(61B)-C(61)-H(61C)	109.5
N(11)-C(62)-H(62A)	109.5
N(11)-C(62)-H(62B)	109.5
H(62A)-C(62)-H(62B)	109.5
N(11)-C(62)-H(62C)	109.5
H(62A)-C(62)-H(62C)	109.5
H(62B)-C(62)-H(62C)	109.5
N(11)-C(63)-C(68)	114.9(3)
N(11)-C(63)-C(64)	111.1(3)
C(68)-C(63)-C(64)	110.3(3)
N(11)-C(63)-H(63)	106.7
C(68)-C(63)-H(63)	106.7
C(64)-C(63)-H(63)	106.7
N(12)-C(64)-C(65)	114.9(3)
N(12)-C(64)-C(63)	111.1(3)
C(65)-C(64)-C(63)	110.9(3)
N(12)-C(64)-H(64)	106.4
C(65)-C(64)-H(64)	106.4
C(63)-C(64)-H(64)	106.4
C(66)-C(65)-C(64)	112.4(3)
C(66)-C(65)-H(65A)	109.1
C(64)-C(65)-H(65A)	109.1
C(66)-C(65)-H(65B)	109.1
C(64)-C(65)-H(65B)	109.1
H(65A)-C(65)-H(65B)	107.9
C(65)-C(66)-C(67)	109.8(4)
C(65)-C(66)-H(66A)	109.7
C(67)-C(66)-H(66A)	109.7
C(65)-C(66)-H(66B)	109.7
C(67)-C(66)-H(66B)	109.7
H(66A)-C(66)-H(66B)	108.2
C(66)-C(67)-C(68)	110.1(4)
C(66)-C(67)-H(67A)	109.6
C(68)-C(67)-H(67A)	109.6
C(66)-C(67)-H(67B)	109.6
C(68)-C(67)-H(67B)	109.6
H(67A)-C(67)-H(67B)	108.1
C(63)-C(68)-C(67)	112.8(4)

C(63)-C(68)-H(68A)	109.0
C(67)-C(68)-H(68A)	109.0
C(63)-C(68)-H(68B)	109.0
C(67)-C(68)-H(68B)	109.0
H(68A)-C(68)-H(68B)	107.8
N(12)-C(69)-H(69A)	109.5
N(12)-C(69)-H(69B)	109.5
H(69A)-C(69)-H(69B)	109.5
N(12)-C(69)-H(69C)	109.5
H(69A)-C(69)-H(69C)	109.5
H(69B)-C(69)-H(69C)	109.5
N(12)-C(70)-H(70A)	109.5
N(12)-C(70)-H(70B)	109.5
H(70A)-C(70)-H(70B)	109.5
N(12)-C(70)-H(70C)	109.5
H(70A)-C(70)-H(70C)	109.5
H(70B)-C(70)-H(70C)	109.5
N(13)-C(71)-H(71A)	109.5
N(13)-C(71)-H(71B)	109.5
H(71A)-C(71)-H(71B)	109.5
N(13)-C(71)-H(71C)	109.5
H(71A)-C(71)-H(71C)	109.5
H(71B)-C(71)-H(71C)	109.5
N(13)-C(72)-H(72A)	109.5
N(13)-C(72)-H(72B)	109.5
H(72A)-C(72)-H(72B)	109.5
N(13)-C(72)-H(72C)	109.5
H(72A)-C(72)-H(72C)	109.5
H(72B)-C(72)-H(72C)	109.5
N(14)-C(73)-H(73A)	109.5
N(14)-C(73)-H(73B)	109.5
H(73A)-C(73)-H(73B)	109.5
N(14)-C(73)-H(73C)	109.5
H(73A)-C(73)-H(73C)	109.5
H(73B)-C(73)-H(73C)	109.5
N(14)-C(74)-H(74A)	109.5
N(14)-C(74)-H(74B)	109.5
H(74A)-C(74)-H(74B)	109.5
N(14)-C(74)-H(74C)	109.5
H(74A)-C(74)-H(74C)	109.5

H(74B)-C(74)-H(74C)	109.5
N(13)-C(75)-C(80)	115.1(3)
N(13)-C(75)-C(76)	111.8(2)
C(80)-C(75)-C(76)	109.9(3)
N(13)-C(75)-H(75)	106.5
C(80)-C(75)-H(75)	106.5
C(76)-C(75)-H(75)	106.5
N(14)-C(76)-C(77)	115.5(3)
N(14)-C(76)-C(75)	110.4(3)
C(77)-C(76)-C(75)	110.6(3)
N(14)-C(76)-H(76)	106.6
C(77)-C(76)-H(76)	106.6
C(75)-C(76)-H(76)	106.6
C(76)-C(77)-C(78)	113.2(3)
C(76)-C(77)-H(77A)	108.9
C(78)-C(77)-H(77A)	108.9
C(76)-C(77)-H(77B)	108.9
C(78)-C(77)-H(77B)	108.9
H(77A)-C(77)-H(77B)	107.7
C(77)-C(78)-C(79)	109.7(3)
C(77)-C(78)-H(78A)	109.7
C(79)-C(78)-H(78A)	109.7
C(77)-C(78)-H(78B)	109.7
C(79)-C(78)-H(78B)	109.7
H(78A)-C(78)-H(78B)	108.2
C(80)-C(79)-C(78)	110.0(3)
C(80)-C(79)-H(79A)	109.7
C(78)-C(79)-H(79A)	109.7
C(80)-C(79)-H(79B)	109.7
C(78)-C(79)-H(79B)	109.7
H(79A)-C(79)-H(79B)	108.2
C(79)-C(80)-C(75)	113.2(3)
C(79)-C(80)-H(80A)	108.9
C(75)-C(80)-H(80A)	108.9
C(79)-C(80)-H(80B)	108.9
C(75)-C(80)-H(80B)	108.9
H(80A)-C(80)-H(80B)	107.7
N(15)-C(81)-H(81A)	109.5
N(15)-C(81)-H(81B)	109.5
H(81A)-C(81)-H(81B)	109.5

N(15)-C(81)-H(81C)	109.5
H(81A)-C(81)-H(81C)	109.5
H(81B)-C(81)-H(81C)	109.5
N(15)-C(82)-H(82A)	109.5
N(15)-C(82)-H(82B)	109.5
H(82A)-C(82)-H(82B)	109.5
N(15)-C(82)-H(82C)	109.5
H(82A)-C(82)-H(82C)	109.5
H(82B)-C(82)-H(82C)	109.5
N(16)-C(83)-H(83A)	109.5
N(16)-C(83)-H(83B)	109.5
H(83A)-C(83)-H(83B)	109.5
N(16)-C(83)-H(83C)	109.5
H(83A)-C(83)-H(83C)	109.5
H(83B)-C(83)-H(83C)	109.5
N(16)-C(84)-H(84A)	109.5
N(16)-C(84)-H(84B)	109.5
H(84A)-C(84)-H(84B)	109.5
N(16)-C(84)-H(84C)	109.5
H(84A)-C(84)-H(84C)	109.5
H(84B)-C(84)-H(84C)	109.5
N(15)-C(85)-C(86)	111.6(3)
N(15)-C(85)-C(90)	114.2(3)
C(86)-C(85)-C(90)	110.1(3)
N(15)-C(85)-H(85)	106.8
C(86)-C(85)-H(85)	106.8
C(90)-C(85)-H(85)	106.8
N(16)-C(86)-C(85)	111.1(3)
N(16)-C(86)-C(87)	114.6(3)
C(85)-C(86)-C(87)	110.8(3)
N(16)-C(86)-H(86)	106.6
C(85)-C(86)-H(86)	106.6
C(87)-C(86)-H(86)	106.6
C(88)-C(87)-C(86)	112.9(3)
C(88)-C(87)-H(87A)	109.0
C(86)-C(87)-H(87A)	109.0
C(88)-C(87)-H(87B)	109.0
C(86)-C(87)-H(87B)	109.0
H(87A)-C(87)-H(87B)	107.8
C(89)-C(88)-C(87)	110.1(3)

C(89)-C(88)-H(88A)	109.6
C(87)-C(88)-H(88A)	109.6
C(89)-C(88)-H(88B)	109.6
C(87)-C(88)-H(88B)	109.6
H(88A)-C(88)-H(88B)	108.1
C(88)-C(89)-C(90)	110.0(3)
C(88)-C(89)-H(89A)	109.7
C(90)-C(89)-H(89A)	109.7
C(88)-C(89)-H(89B)	109.7
C(90)-C(89)-H(89B)	109.7
H(89A)-C(89)-H(89B)	108.2
C(89)-C(90)-C(85)	112.1(3)
C(89)-C(90)-H(90A)	109.2
C(85)-C(90)-H(90A)	109.2
C(89)-C(90)-H(90B)	109.2
C(85)-C(90)-H(90B)	109.2
H(90A)-C(90)-H(90B)	107.9
N(17)-C(91)-H(91A)	109.5
N(17)-C(91)-H(91B)	109.5
H(91A)-C(91)-H(91B)	109.5
N(17)-C(91)-H(91C)	109.5
H(91A)-C(91)-H(91C)	109.5
H(91B)-C(91)-H(91C)	109.5
N(17)-C(92)-H(92A)	109.5
N(17)-C(92)-H(92B)	109.5
H(92A)-C(92)-H(92B)	109.5
N(17)-C(92)-H(92C)	109.5
H(92A)-C(92)-H(92C)	109.5
H(92B)-C(92)-H(92C)	109.5
N(18)-C(93)-H(93A)	109.5
N(18)-C(93)-H(93B)	109.5
H(93A)-C(93)-H(93B)	109.5
N(18)-C(93)-H(93C)	109.5
H(93A)-C(93)-H(93C)	109.5
H(93B)-C(93)-H(93C)	109.5
N(18)-C(94)-H(94A)	109.5
N(18)-C(94)-H(94B)	109.5
H(94A)-C(94)-H(94B)	109.5
N(18)-C(94)-H(94C)	109.5
H(94A)-C(94)-H(94C)	109.5

H(94B)-C(94)-H(94C)	109.5
N(17)-C(95)-C(96)	110.8(3)
N(17)-C(95)-C(100)	114.4(3)
C(96)-C(95)-C(100)	110.9(3)
N(17)-C(95)-H(95)	106.8
C(96)-C(95)-H(95)	106.8
C(100)-C(95)-H(95)	106.8
N(18)-C(96)-C(95)	112.3(3)
N(18)-C(96)-C(97)	114.1(3)
C(95)-C(96)-C(97)	110.9(3)
N(18)-C(96)-H(96)	106.3
C(95)-C(96)-H(96)	106.3
C(97)-C(96)-H(96)	106.3
C(98)-C(97)-C(96)	111.6(3)
C(98)-C(97)-H(97A)	109.3
C(96)-C(97)-H(97A)	109.3
C(98)-C(97)-H(97B)	109.3
C(96)-C(97)-H(97B)	109.3
H(97A)-C(97)-H(97B)	108.0
C(99)-C(98)-C(97)	110.0(4)
C(99)-C(98)-H(98A)	109.7
C(97)-C(98)-H(98A)	109.7
C(99)-C(98)-H(98B)	109.7
C(97)-C(98)-H(98B)	109.7
H(98A)-C(98)-H(98B)	108.2
C(98)-C(99)-C(100)	111.4(3)
C(98)-C(99)-H(99A)	109.3
C(100)-C(99)-H(99A)	109.3
C(98)-C(99)-H(99B)	109.3
C(100)-C(99)-H(99B)	109.3
H(99A)-C(99)-H(99B)	108.0
C(99)-C(100)-C(95)	112.1(3)
C(99)-C(100)-H(100)	109.2
C(95)-C(100)-H(100)	109.2
C(99)-C(100)-H(101)	109.2
C(95)-C(100)-H(101)	109.2
H(100)-C(100)-H(101)	107.9

Symmetry transformations used to generate equivalent atoms:

Table 4. Anisotropic displacement parameters ($Å^2 x 10^3$) for srgr1006. The anisotropic displacement factor exponent takes the form: $-2\pi^2[h^2 a^{*2}U^{11} + ... + 2 h k a^* b^* U^{12}]$

	U^{11}	U^{22}	U^{33}	U^{23}	U^{13}	U^{12}
Li(1)	27(3)	28(5)	16(3)	-1(3)	4(2)	-8(3)
Li(2)	26(4)	24(5)	35(4)	-10(4)	1(3)	5(4)
Li(3)	35(4)	10(4)	31(4)	-5(3)	10(3)	0(3)
Li(4)	25(4)	19(5)	16(4)	-3(3)	4(3)	2(3)
Li(5)	23(4)	22(6)	28(4)	-1(3)	5(3)	-2(3)
Li(6)	31(4)	24(6)	23(4)	1(3)	8(3)	-4(4)
Li(7)	25(4)	27(6)	24(4)	1(3)	2(3)	-1(4)
Li(8)	25(4)	46(6)	20(4)	13(4)	3(3)	-1(4)
Li(9)	32(4)	20(5)	20(4)	11(3)	7(3)	3(3)
Li(10)	28(4)	26(5)	25(4)	-3(3)	5(3)	0(3)
Li(11)	31(4)	26(5)	22(4)	2(3)	14(3)	9(3)
Li(12)	22(4)	23(5)	22(4)	-1(3)	-1(3)	-6(3)
Cl(1)	51(1)	14(1)	19(1)	-1(1)	15(1)	2(1)
Cl(2)	50(1)	15(1)	18(1)	1(1)	12(1)	2(1)
Si(1)	41(1)	24(1)	26(1)	-5(1)	10(1)	-1(1)
Si(2)	28(1)	28(1)	29(1)	-2(1)	15(1)	0(1)
Si(3)	23(1)	23(1)	29(1)	4(1)	7(1)	-1(1)
Si(4)	23(1)	18(1)	34(1)	6(1)	0(1)	-1(1)
Si(5)	34(1)	37(1)	25(1)	-2(1)	15(1)	-3(1)
Si(6)	34(1)	29(1)	20(1)	4(1)	5(1)	0(1)
Si(7)	21(1)	19(1)	28(1)	-4(1)	1(1)	3(1)
Si(8)	20(1)	19(1)	27(1)	-5(1)	2(1)	-2(1)
Si(9)	22(1)	25(1)	22(1)	-3(1)	2(1)	-1(1)
Si(10)	27(1)	22(1)	22(1)	6(1)	4(1)	-5(1)
Si(11)	24(1)	21(1)	24(1)	-5(1)	2(1)	3(1)
Si(12)	26(1)	19(1)	24(1)	0(1)	-2(1)	-4(1)
Si(13)	34(1)	44(1)	23(1)	-3(1)	11(1)	-3(1)
Si(14)	34(1)	22(1)	18(1)	1(1)	2(1)	2(1)
Si(15)	29(1)	21(1)	34(1)	2(1)	6(1)	-3(1)
Si(16)	29(1)	16(1)	28(1)	2(1)	4(1)	0(1)
Si(17)	48(1)	22(1)	21(1)	-5(1)	3(1)	8(1)
Si(18)	32(1)	35(1)	29(1)	2(1)	14(1)	11(1)
Si(19)	21(1)	21(1)	20(1)	-2(1)	3(1)	-2(1)
Si(20)	27(1)	26(1)	21(1)	2(1)	6(1)	-6(1)
N(1)	23(2)	18(2)	23(2)	-3(2)	7(1)	4(2)

N(2)	23(2)	13(2)	18(2)	2(2)	4(1)	2(2)
N(3)	27(2)	20(2)	16(2)	-1(2)	5(1)	-2(2)
N(4)	17(2)	15(2)	20(2)	-2(2)	-1(1)	0(2)
N(5)	20(2)	18(3)	18(2)	2(2)	4(1)	-3(2)
N(6)	21(2)	15(2)	20(2)	-4(2)	6(1)	-2(2)
N(7)	28(2)	27(3)	12(2)	1(2)	6(1)	-2(2)
N(8)	25(2)	16(3)	24(2)	1(2)	2(1)	-3(2)
N(9)	29(2)	24(3)	20(2)	-1(2)	7(1)	7(2)
N(10)	18(2)	20(3)	15(2)	1(2)	1(1)	0(2)
N(11)	23(2)	25(2)	30(2)	-5(2)	10(1)	-2(2)
N(12)	43(2)	22(2)	26(2)	-7(2)	13(2)	-3(2)
N(13)	21(2)	22(2)	25(2)	-3(1)	3(1)	-1(1)
N(14)	22(2)	18(2)	24(2)	0(1)	7(1)	2(1)
N(15)	16(2)	23(2)	18(2)	3(1)	0(1)	2(1)
N(16)	16(1)	20(2)	20(2)	-4(1)	4(1)	-3(1)
N(17)	24(2)	24(2)	20(2)	0(1)	7(1)	-4(2)
N(18)	31(2)	17(2)	15(2)	0(1)	10(1)	2(2)
C(1)	41(2)	30(4)	40(3)	-6(2)	2(2)	-8(2)
C(2)	68(3)	42(4)	26(2)	-9(2)	2(2)	-6(3)
C(3)	64(3)	29(3)	51(3)	-20(2)	20(2)	-2(3)
C(4)	34(2)	43(4)	44(3)	0(2)	20(2)	8(2)
C(5)	45(2)	40(3)	39(2)	3(2)	22(2)	3(2)
C(6)	30(2)	28(3)	39(2)	1(2)	11(2)	-4(2)
C(7)	26(2)	37(3)	40(2)	0(2)	4(2)	1(2)
C(8)	36(2)	48(4)	41(3)	17(3)	12(2)	3(3)
C(9)	33(2)	25(3)	48(3)	2(2)	2(2)	-14(2)
C(10)	35(3)	43(4)	49(3)	24(3)	-5(2)	-2(3)
C(11)	27(2)	25(3)	62(3)	-9(2)	6(2)	5(2)
C(12)	35(2)	31(3)	33(2)	3(2)	6(2)	-10(2)
C(13)	29(2)	37(3)	48(2)	-2(2)	8(2)	2(2)
C(14)	53(3)	71(5)	47(3)	-27(3)	22(2)	2(3)
C(15)	50(3)	89(5)	32(3)	7(3)	19(2)	-16(3)
C(16)	60(3)	47(4)	24(2)	1(2)	0(2)	-9(2)
C(17)	53(3)	34(4)	37(3)	10(2)	13(2)	10(2)
C(18)	32(2)	32(3)	45(3)	9(2)	4(2)	-3(2)
C(19)	24(2)	26(3)	36(2)	4(2)	8(2)	3(2)
C(20)	34(3)	46(4)	48(3)	-11(3)	1(2)	17(2)
C(21)	24(2)	24(4)	52(3)	0(2)	-2(2)	-3(2)
C(22)	36(3)	31(4)	44(3)	-6(2)	-3(2)	-12(2)
C(23)	30(2)	39(4)	25(2)	-7(2)	-3(2)	0(2)

C(24)	21(2)	39(4)	26(2)	5(2)	0(2)	-7(2)
C(25)	26(2)	42(4)	41(3)	-9(2)	-4(2)	4(2)
C(26)	24(2)	43(4)	39(3)	-9(2)	6(2)	-12(2)
C(27)	37(3)	40(4)	20(2)	-2(2)	1(2)	0(2)
C(28)	51(3)	34(4)	41(3)	13(2)	7(2)	-2(3)
C(29)	43(3)	42(4)	31(2)	7(2)	14(2)	-7(2)
C(30)	32(2)	34(4)	39(3)	1(2)	9(2)	-12(2)
C(31)	22(2)	42(4)	29(2)	-2(2)	-1(2)	3(2)
C(32)	30(2)	25(4)	40(3)	10(2)	-4(2)	4(2)
C(33)	38(3)	33(4)	37(3)	-14(2)	-1(2)	13(2)
C(34)	29(2)	36(3)	34(3)	-13(2)	-1(2)	-3(2)
C(35)	24(2)	41(4)	31(2)	1(2)	-4(2)	-5(2)
C(36)	41(3)	19(3)	46(3)	-2(2)	-1(2)	-8(2)
C(37)	43(3)	89(5)	35(3)	13(3)	17(2)	-20(3)
C(38)	38(2)	50(4)	39(2)	-1(2)	16(2)	7(2)
C(39)	45(3)	56(4)	58(3)	-25(3)	23(2)	4(2)
C(40)	44(3)	37(4)	29(2)	10(2)	6(2)	2(2)
C(41)	32(2)	39(4)	41(2)	2(2)	3(2)	1(2)
C(42)	52(3)	44(3)	25(2)	0(2)	-2(2)	10(2)
C(43)	42(3)	45(4)	51(3)	16(3)	20(2)	-5(3)
C(44)	25(2)	45(4)	38(2)	2(2)	7(2)	1(2)
C(45)	37(3)	30(4)	57(3)	-4(2)	7(2)	-9(2)
C(46)	48(3)	26(3)	49(3)	-10(2)	7(2)	10(2)
C(47)	40(3)	24(3)	53(3)	18(2)	6(2)	1(2)
C(48)	33(2)	31(3)	34(2)	0(2)	10(2)	4(2)
C(49)	53(3)	28(4)	37(3)	-4(2)	-8(2)	-6(2)
C(50)	70(3)	43(4)	29(2)	-7(2)	8(2)	18(3)
C(51)	73(3)	37(4)	29(3)	2(2)	10(2)	16(3)
C(52)	34(2)	58(4)	31(2)	8(2)	15(2)	15(2)
C(53)	48(3)	43(4)	45(3)	7(2)	28(2)	12(2)
C(54)	21(2)	62(4)	38(2)	-1(2)	8(2)	-11(2)
C(55)	29(2)	29(3)	35(2)	2(2)	1(2)	-6(2)
C(56)	31(2)	35(4)	34(3)	-8(2)	7(2)	-4(2)
C(57)	22(2)	38(3)	25(2)	-6(2)	0(2)	4(2)
C(58)	33(2)	47(4)	30(2)	4(2)	14(2)	-6(2)
C(59)	44(3)	26(3)	33(3)	11(2)	4(2)	-10(2)
C(60)	31(2)	30(4)	33(3)	1(2)	4(2)	-11(2)
C(61)	51(3)	34(3)	37(2)	10(2)	26(2)	9(2)
C(62)	57(3)	41(3)	28(2)	-6(2)	8(2)	-1(2)
C(63)	17(2)	20(2)	35(2)	-3(2)	8(2)	0(2)

C(64)	27(2)	18(2)	33(2)	2(2)	10(2)	-2(2)
C(65)	44(2)	19(2)	50(2)	-11(2)	13(2)	-5(2)
C(66)	63(3)	19(2)	61(3)	-5(2)	13(2)	8(2)
C(67)	67(3)	36(3)	48(3)	5(2)	-4(2)	3(3)
C(68)	49(3)	18(3)	44(3)	1(2)	3(2)	10(2)
C(69)	79(3)	27(2)	45(3)	-7(2)	36(2)	-5(2)
C(70)	72(3)	46(3)	30(2)	-7(2)	-5(2)	-5(2)
C(71)	37(2)	45(3)	44(3)	5(2)	5(2)	-5(2)
C(72)	29(2)	37(3)	40(2)	2(2)	6(2)	5(2)
C(73)	28(2)	47(3)	29(2)	-2(2)	-3(2)	2(2)
C(74)	35(2)	23(3)	36(2)	-4(2)	17(2)	-1(2)
C(75)	27(2)	23(2)	21(2)	-1(2)	8(1)	2(2)
C(76)	25(2)	26(2)	20(2)	-7(2)	7(2)	0(2)
C(77)	29(2)	21(3)	31(2)	-4(2)	3(2)	-6(2)
C(78)	44(2)	22(2)	44(2)	3(2)	11(2)	-2(2)
C(79)	47(2)	23(2)	44(2)	3(2)	7(2)	8(2)
C(80)	30(2)	31(3)	31(2)	2(2)	3(2)	10(2)
C(81)	20(2)	28(3)	37(2)	4(2)	11(2)	3(2)
C(82)	25(2)	30(3)	27(2)	6(2)	2(2)	1(2)
C(83)	29(2)	30(3)	40(2)	0(2)	6(2)	2(2)
C(84)	30(2)	22(2)	40(2)	2(2)	14(2)	4(2)
C(85)	22(2)	15(2)	19(2)	-2(2)	6(1)	-2(1)
C(86)	22(2)	14(2)	24(2)	-3(1)	7(1)	0(1)
C(87)	30(2)	20(2)	32(2)	6(2)	16(2)	5(2)
C(88)	44(2)	15(2)	40(2)	3(2)	16(2)	6(2)
C(89)	39(2)	20(2)	39(2)	3(2)	12(2)	-1(2)
C(90)	29(2)	25(3)	29(2)	1(2)	14(2)	-2(2)
C(91)	45(2)	40(3)	27(2)	-13(2)	14(2)	-6(2)
C(92)	32(2)	24(2)	40(2)	2(2)	-6(2)	-1(2)
C(93)	45(2)	19(2)	25(2)	-5(2)	8(2)	4(2)
C(94)	33(2)	47(3)	34(2)	10(2)	18(2)	11(2)
C(95)	23(2)	24(2)	27(2)	-6(2)	4(1)	0(2)
C(96)	21(2)	16(2)	29(2)	-3(2)	4(2)	-3(2)
C(97)	43(3)	34(4)	22(2)	3(2)	2(2)	3(2)
C(98)	60(3)	17(3)	54(3)	-2(2)	-9(2)	17(2)
C(99)	63(3)	29(2)	59(3)	-16(2)	0(2)	24(2)
C(100)	46(2)	21(3)	44(2)	-12(2)	10(2)	6(2)

Table 5. Hydrogen coordinates (x 10^4) and isotropic displacement parameters (Å^{2}x 10 ^{3}) for srgr1006.

	x	y	z	U(eq)
H(1A)	4632	2684	-648	57
H(1B)	4198	2536	-1461	57
H(1C)	3938	3034	-1146	57
H(2A)	4427	3606	-2319	70
H(2B)	4625	3118	-2697	70
H(2C)	5364	3531	-2545	70
H(3A)	6629	2729	-1774	70
H(3B)	5770	2396	-2021	70
H(3C)	6284	2405	-1194	70
H(4A)	7735	3184	239	58
H(4B)	8384	3312	-290	58
H(4C)	7659	2896	-499	58
H(5A)	7188	3494	-1924	59
H(5B)	7898	3882	-1537	59
H(5C)	6893	4040	-1874	59
H(6A)	6911	4484	-423	48
H(6B)	7874	4269	-77	48
H(6C)	7047	4165	292	48
H(7A)	3387	3465	502	52
H(7B)	2675	3045	487	52
H(7C)	3239	3075	-129	52
H(8A)	4181	2394	2105	62
H(8B)	3224	2630	1802	62
H(8C)	4058	2961	2144	62
H(9A)	4148	2129	61	54
H(9B)	3382	2022	494	54
H(9C)	4399	1899	850	54
H(10A)	5958	2402	2419	66
H(10B)	6705	2086	2165	66
H(10C)	5671	1980	1847	66
H(11A)	5939	2046	366	58
H(11B)	6978	2154	650	58
H(11C)	6379	2506	78	58
H(12A)	7032	3358	1243	50

H(12B)	7653	2903	1503	50
H(12C)	7146	3165	2050	50
H(13A)	3278	4184	1415	57
H(13B)	2669	4144	1999	57
H(13C)	3183	3682	1798	57
H(14A)	4472	4909	3280	83
H(14B)	3420	4824	3031	83
H(14C)	3971	5030	2472	83
H(15A)	4216	3458	3256	83
H(15B)	3510	3821	3472	83
H(15C)	4553	3890	3799	83
H(16A)	6232	4776	3625	68
H(16B)	6918	4387	4041	68
H(16C)	5877	4340	4032	68
H(17A)	5846	3355	3510	61
H(17B)	6899	3402	3573	61
H(17C)	6275	3214	2841	61
H(18A)	6953	4132	1958	56
H(18B)	7644	4053	2708	56
H(18C)	7214	4570	2506	56
H(19A)	3350	4908	371	43
H(19B)	2632	5327	346	43
H(19C)	3008	5048	1083	43
H(20A)	3780	5798	2030	66
H(20B)	3199	6140	1435	66
H(20C)	4235	6250	1737	66
H(21A)	4478	6252	173	52
H(21B)	3417	6194	3	52
H(21C)	4014	5831	-344	52
H(22A)	6104	6301	827	58
H(22B)	6696	6380	1620	58
H(22C)	5635	6440	1475	58
H(23A)	5590	5801	2651	49
H(23B)	6637	5692	2801	49
H(23C)	5936	5260	2676	49
H(24A)	6913	4956	1296	44
H(24B)	7530	5341	1792	44
H(24C)	7201	5436	946	44
H(25A)	2903	5242	-950	57
H(25B)	2407	5101	-1751	57

H(25C)	3139	5518	-1624	57
H(26A)	3869	3922	-1214	53
H(26B)	2860	4103	-1456	53
H(26C)	3415	4210	-658	53
H(27A)	3912	4986	-2697	50
H(27B)	3283	4527	-2715	50
H(27C)	4345	4466	-2527	50
H(28A)	4505	5720	-2255	63
H(28B)	5332	6065	-1949	63
H(28C)	4550	6005	-1518	63
H(29A)	6717	4867	-1584	57
H(29B)	6578	5348	-2051	57
H(29C)	5918	4904	-2277	57
H(30A)	5938	5711	-157	52
H(30B)	6553	5900	-681	52
H(30C)	6752	5391	-292	52
H(31A)	11852	6762	3856	48
H(31B)	12364	6437	4508	48
H(31C)	11661	6831	4645	48
H(32A)	10850	5906	5297	50
H(32B)	11578	5595	5011	50
H(32C)	10543	5464	4769	50
H(33A)	10698	5479	3247	56
H(33B)	11729	5625	3470	56
H(33C)	11054	5940	2894	56
H(34A)	8860	6524	2336	51
H(34B)	8279	6044	2223	51
H(34C)	9342	6015	2349	51
H(35A)	7658	6246	4007	50
H(35B)	7463	6488	3225	50
H(35C)	8075	6759	3895	50
H(36A)	9316	5315	3588	56
H(36B)	8254	5366	3380	56
H(36C)	8777	5456	4192	56
H(37A)	10170	7867	1149	82
H(37B)	11237	7855	1341	82
H(37C)	10715	8267	1666	82
H(38A)	11737	8041	3091	62
H(38B)	12238	7598	2817	62
H(38C)	11719	7526	3456	62

H(39A)	10875	6693	2455	77
H(39B)	11373	6887	1852	77
H(39C)	10314	6816	1665	77
H(40A)	8793	8552	2222	55
H(40B)	7964	8397	1601	55
H(40C)	8951	8391	1451	55
H(41A)	7723	7204	2595	57
H(41B)	7277	7716	2385	57
H(41C)	8004	7652	3119	57
H(42A)	8921	7387	1000	63
H(42B)	7880	7435	993	63
H(42C)	8440	6996	1401	63
H(43A)	10922	8758	2792	66
H(43B)	11680	9148	3076	66
H(43C)	10666	9313	2801	66
H(44A)	11738	8592	5019	54
H(44B)	12353	8723	4464	54
H(44C)	11697	8272	4315	54
H(45A)	10615	9834	4151	63
H(45B)	11664	9729	4322	63
H(45C)	11088	9606	4909	63
H(46A)	8616	9233	4961	62
H(46B)	8176	9652	4427	62
H(46C)	9227	9653	4756	62
H(47A)	9398	9727	3123	59
H(47B)	8334	9741	2992	59
H(47C)	8811	9359	2573	59
H(48A)	7843	8574	3036	49
H(48B)	7347	8877	3548	49
H(48C)	7939	8423	3861	49
H(49A)	11131	8706	6033	63
H(49B)	10986	9190	6444	63
H(49C)	10440	9100	5639	63
H(50A)	8783	9322	6222	71
H(50B)	9440	9389	6991	71
H(50C)	8604	9035	6906	71
H(51A)	9867	8242	7579	70
H(51B)	10606	8655	7679	70
H(51C)	10770	8160	7302	70
H(52A)	7428	8859	5664	60

H(52B)	6722	8447	5363	60
H(52C)	7407	8626	4891	60
H(53A)	8373	7746	6999	64
H(53B)	7331	7825	6682	64
H(53C)	7947	8268	7007	64
H(54A)	7864	7595	4771	60
H(54B)	7265	7425	5319	60
H(54C)	8297	7280	5461	60
H(55A)	11650	7499	5654	47
H(55B)	12228	7596	6447	47
H(55C)	11232	7799	6220	47
H(56A)	10821	7311	7531	49
H(56B)	11833	7130	7718	49
H(56C)	11036	6754	7665	49
H(57A)	11904	6195	6611	44
H(57B)	12650	6604	6719	44
H(57C)	12124	6463	5926	44
H(58A)	9235	6771	7385	53
H(58B)	8482	6383	7081	53
H(58C)	8442	6902	6719	53
H(59A)	10501	5747	6453	53
H(59B)	9747	5669	6908	53
H(59C)	10576	6013	7210	53
H(60A)	8299	6392	5284	47
H(60B)	8372	5892	5703	47
H(60C)	9036	6009	5180	47
H(61A)	5730	6804	3789	57
H(61B)	5962	6255	3983	57
H(61C)	6170	6654	4600	57
H(62A)	4300	6480	3239	63
H(62B)	3664	6258	3721	63
H(62C)	4494	5956	3575	63
H(63)	3849	6867	4575	28
H(64)	5594	7217	5169	31
H(65A)	3968	7518	5537	45
H(65B)	4937	7708	5917	45
H(66A)	5106	8066	4834	57
H(66B)	4216	8263	5049	57
H(67A)	3928	8045	3805	63
H(67B)	3334	7729	4229	63

H(68A)	3990	7218	3493	46
H(68B)	4963	7413	3856	46
H(69A)	4307	6991	6435	70
H(69B)	4436	6422	6479	70
H(69C)	3798	6658	5791	70
H(70A)	6457	6837	6145	77
H(70B)	5994	6602	6740	77
H(70C)	5875	7161	6566	77
H(71A)	7204	5459	6388	64
H(71B)	6474	5758	6693	64
H(71C)	6999	6003	6144	64
H(72A)	6694	5777	4914	53
H(72B)	6009	5350	4640	53
H(72C)	6946	5239	5170	53
H(73A)	3043	5131	4651	54
H(73B)	3717	5326	4180	54
H(73C)	3097	5692	4498	54
H(74A)	3289	5268	5881	45
H(74B)	3380	5834	5785	45
H(74C)	4156	5543	6308	45
H(75)	5476	5172	6360	28
H(76)	4852	4927	4880	28
H(77A)	3720	4494	5234	33
H(77B)	4110	4629	6059	33
H(78A)	4998	4035	5201	44
H(78B)	4561	3849	5846	44
H(79A)	5617	4286	6692	46
H(79B)	6139	3942	6246	46
H(80A)	6356	4578	5503	37
H(80B)	6753	4717	6325	37
H(81A)	11829	6427	-916	42
H(81B)	11066	6089	-1362	42
H(81C)	11773	5878	-692	42
H(82A)	11739	6138	507	42
H(82B)	11014	6523	628	42
H(82C)	11799	6678	240	42
H(83A)	8165	5798	-1075	50
H(83B)	8720	6154	-1474	50
H(83C)	7873	6347	-1194	50
H(84A)	8807	6296	643	45

H(84B)	8130	5930	160	45
H(84C)	7968	6494	66	45
H(85)	10011	6611	-1242	22
H(86)	9627	6816	128	23
H(87A)	8539	7046	-1248	31
H(87B)	8284	7157	-491	31
H(88A)	8839	7856	-961	38
H(88B)	9460	7713	-200	38
H(89A)	10374	7885	-1021	38
H(89B)	9851	7498	-1577	38
H(90A)	10927	7273	-183	32
H(90B)	11205	7160	-931	32
H(91A)	10195	4816	-1731	55
H(91B)	10950	5173	-1318	55
H(91C)	10017	5381	-1749	55
H(92A)	8692	5087	-1433	51
H(92B)	8708	4938	-617	51
H(92C)	9008	4562	-1154	51
H(93A)	10888	5241	1663	44
H(93B)	10663	5759	1311	44
H(93C)	11309	5405	1000	44
H(94A)	8709	5084	749	54
H(94B)	9131	5526	1242	54
H(94C)	9398	4989	1496	54
H(95)	11113	4907	-127	30
H(96)	9557	4579	302	26
H(97A)	11347	4554	1155	40
H(97B)	10482	4389	1435	40
H(98A)	11225	3718	1086	56
H(98B)	10239	3760	595	56
H(99A)	11262	3615	-137	63
H(99B)	11826	4077	183	63
H(100)	10055	4096	-690	44
H(101)	10931	4272	-945	44

X-ray Data for **3 (PAGE 74 – 167)**

Table 1. Crystal data and structure refinement for srgr1016.

Identification code	srgr1016	
Empirical formula	C61 H158 Cl2 Li7 N13 Si10	
Formula weight	1474.38	
Temperature	123(2) K	
Wavelength	0.71073 Å	
Crystal system	Monoclinic	
Space group	C 2/c	
Unit cell dimensions	a = 17.8926(4) Å	α= 90°.
	b = 19.5064(4) Å	β= 94.934(2)°.
	c = 27.4603(7) Å	γ = 90°.
Volume	9548.7(4) $Å^3$	
Z	4	
Density (calculated)	1.026 Mg/m^3	
Absorption coefficient	0.232 mm^{-1}	
F(000)	3240	
Crystal size	0.20 x 0.10 x 0.04 mm^3	
Theta range for data collection	3.06 to 29.58°.	
Index ranges	-22<=h<=24, -26<=k<=25, -36<=l<=31	
Reflections collected	28913	
Independent reflections	11885 [R(int) = 0.0292]	
Completeness to theta = 27.00°	99.7 %	
Absorption correction	Semi-empirical from equivalents	
Max. and min. transmission	1.00000 and 0.93998	
Refinement method	Full-matrix least-squares on F^2	
Data / restraints / parameters	11885 / 100 / 464	
Goodness-of-fit on F^2	1.022	
Final R indices [I>2sigma(I)]	R1 = 0.0425, wR2 = 0.0942	
R indices (all data)	R1 = 0.0671, wR2 = 0.1067	
Largest diff. peak and hole	0.321 and -0.238 e.$Å^{-3}$	

Table 2. Atomic coordinates (x 10^4) and equivalent isotropic displacement parameters ($Å^2$x 10^3) for srgr1016. U(eq) is defined as one third of the trace of the orthogonalized U^{ij} tensor.

	x	y	z	U(eq)
Li(1)	10636(2)	4402(1)	1794(1)	26(1)
Li(2)	10434(2)	5801(1)	2081(1)	25(1)
Li(3)	10000	3535(2)	2500	28(1)
Li(4)	5890(1)	4612(1)	646(1)	23(1)
Cl(1)	10000	4793(1)	2500	39(1)
Cl(2)	5000	5000	0	30(1)
Si(1)	11372(1)	3049(1)	2004(1)	29(1)
Si(2)	9906(1)	2954(1)	1429(1)	25(1)
Si(3)	11894(1)	5580(1)	1630(1)	28(1)
Si(4)	10533(1)	5383(1)	958(1)	23(1)
Si(5)	9364(1)	6993(1)	2123(1)	21(1)
N(1)	10486(1)	3363(1)	1864(1)	23(1)
N(2)	10971(1)	5345(1)	1537(1)	22(1)
N(3)	10000	6548(1)	2500	18(1)
N(4)	6684(1)	4242(1)	1272(1)	24(1)
N(5)	5120(1)	4629(1)	1254(1)	27(1)
N(6)	6198(1)	3605(1)	347(1)	25(1)
N(7)	6751(1)	5406(1)	628(1)	25(1)
C(1)	11758(1)	3391(1)	2614(1)	38(1)
C(2)	11452(1)	2087(1)	2045(1)	48(1)
C(3)	12035(1)	3330(1)	1550(1)	48(1)
C(4)	9460(1)	2160(1)	1667(1)	42(1)
C(5)	9124(1)	3530(1)	1178(1)	35(1)
C(6)	10360(1)	2656(1)	874(1)	38(1)
C(7)	12011(1)	6535(1)	1679(1)	40(1)
C(8)	12340(1)	5197(1)	2213(1)	42(1)
C(9)	12507(1)	5311(1)	1141(1)	46(1)
C(10)	10758(1)	4615(1)	584(1)	35(1)
C(11)	10757(1)	6150(1)	582(1)	36(1)
C(12)	9486(1)	5394(1)	986(1)	32(1)
C(13)	8975(1)	7797(1)	2386(1)	35(1)
C(14)	9783(1)	7276(1)	1551(1)	32(1)
C(15)	8535(1)	6439(1)	1929(1)	34(1)
C(20)	4366(1)	4349(1)	1122(1)	41(1)
C(21)	5041(1)	5353(1)	1384(1)	39(1)

C(22)	5489(1)	4238(1)	1662(1)	31(1)
C(23)	6328(1)	4365(1)	1727(1)	30(1)
C(24)	5533(1)	3185(1)	403(1)	33(1)
C(25)	6349(1)	3593(1)	-170(1)	37(1)
C(26)	6852(1)	3346(1)	654(1)	30(1)
C(27)	6809(1)	3505(1)	1192(1)	29(1)
C(28)	6433(1)	6098(1)	556(1)	34(1)
C(29)	7204(1)	5256(1)	222(1)	36(1)
C(30)	7210(1)	5374(1)	1097(1)	29(1)
C(31)	7378(1)	4641(1)	1256(1)	30(1)
C(1S)	7047(1)	7710(2)	-95(1)	34(1)
C(2S)	7403(2)	7170(2)	-312(1)	37(1)
C(3S)	8095(2)	6937(1)	-108(1)	50(1)
C(4S)	8431(1)	7244(2)	313(1)	51(1)
C(5S)	8075(2)	7784(2)	529(1)	41(1)
C(6S)	7383(2)	8017(1)	326(1)	34(1)
C(7S)	6300(3)	7975(3)	-316(2)	53(1)

Table 3. Bond lengths [Å] and angles [°] for srgr1016.

Li(1)-N(1)	2.057(3)
Li(1)-N(2)	2.076(3)
Li(1)-Cl(1)	2.452(3)
Li(1)-Li(2)	2.871(4)
Li(1)-Li(3)	2.881(4)
Li(1)-Si(4)	2.981(3)
Li(1)-Si(1)	2.985(3)
Li(2)-N(2)	2.049(3)
Li(2)-N(3)	2.050(3)
Li(2)-Cl(1)	2.438(3)
Li(2)-Li(2)#1	2.884(5)
Li(2)-Si(3)	3.017(3)
Li(2)-Si(5)	3.021(3)
Li(3)-N(1)#1	2.0439(15)
Li(3)-N(1)	2.0439(15)
Li(3)-Cl(1)	2.455(4)
Li(3)-Li(1)#1	2.881(4)
Li(3)-Si(1)#1	3.0587(13)
Li(3)-Si(1)	3.0588(13)
Li(3)-Si(2)#1	3.1424(15)
Li(3)-Si(2)	3.1424(15)
Li(4)-N(7)	2.186(3)
Li(4)-N(6)	2.217(3)
Li(4)-N(4)	2.254(3)
Li(4)-N(5)	2.257(3)
Li(4)-Cl(2)	2.401(3)
Cl(1)-Li(2)#1	2.438(3)
Cl(1)-Li(1)#1	2.452(3)
Cl(2)-Li(4)#2	2.401(3)
Si(1)-N(1)	1.7135(13)
Si(1)-C(3)	1.875(2)
Si(1)-C(1)	1.8773(19)
Si(1)-C(2)	1.884(2)
Si(2)-N(1)	1.7107(14)
Si(2)-C(5)	1.8792(17)
Si(2)-C(6)	1.8797(19)
Si(2)-C(4)	1.8833(19)
Si(3)-N(2)	1.7126(13)

Si(3)-C(7)	1.8771(19)
Si(3)-C(8)	1.880(2)
Si(3)-C(9)	1.8818(19)
Si(4)-N(2)	1.7114(14)
Si(4)-C(12)	1.8802(17)
Si(4)-C(10)	1.8804(17)
Si(4)-C(11)	1.8806(18)
Si(5)-N(3)	1.7091(10)
Si(5)-C(15)	1.8750(17)
Si(5)-C(14)	1.8798(18)
Si(5)-C(13)	1.8832(17)
N(3)-Si(5)#1	1.7091(10)
N(3)-Li(2)#1	2.050(3)
N(4)-C(23)	1.468(2)
N(4)-C(31)	1.470(2)
N(4)-C(27)	1.474(2)
N(5)-C(22)	1.463(2)
N(5)-C(21)	1.465(2)
N(5)-C(20)	1.471(2)
N(6)-C(24)	1.464(2)
N(6)-C(25)	1.466(2)
N(6)-C(26)	1.473(2)
N(7)-C(29)	1.462(2)
N(7)-C(30)	1.469(2)
N(7)-C(28)	1.472(2)
C(1)-H(1A)	0.9800
C(1)-H(1B)	0.9800
C(1)-H(1C)	0.9800
C(2)-H(2A)	0.9800
C(2)-H(2B)	0.9800
C(2)-H(2C)	0.9800
C(3)-H(3A)	0.9800
C(3)-H(3B)	0.9800
C(3)-H(3C)	0.9800
C(4)-H(4A)	0.9800
C(4)-H(4B)	0.9800
C(4)-H(4C)	0.9800
C(5)-H(5A)	0.9800
C(5)-H(5B)	0.9800
C(5)-H(5C)	0.9800

C(6)-H(6A)	0.9800
C(6)-H(6B)	0.9800
C(6)-H(6C)	0.9800
C(7)-H(7A)	0.9800
C(7)-H(7B)	0.9800
C(7)-H(7C)	0.9800
C(8)-H(8A)	0.9800
C(8)-H(8B)	0.9800
C(8)-H(8C)	0.9800
C(9)-H(9A)	0.9800
C(9)-H(9B)	0.9800
C(9)-H(9C)	0.9800
C(10)-H(10A)	0.9800
C(10)-H(10B)	0.9800
C(10)-H(10C)	0.9800
C(11)-H(11A)	0.9800
C(11)-H(11B)	0.9800
C(11)-H(11C)	0.9800
C(12)-H(12A)	0.9800
C(12)-H(12B)	0.9800
C(12)-H(12C)	0.9800
C(13)-H(13A)	0.9800
C(13)-H(13B)	0.9800
C(13)-H(13C)	0.9800
C(14)-H(14A)	0.9800
C(14)-H(14B)	0.9800
C(14)-H(14C)	0.9800
C(15)-H(15A)	0.9800
C(15)-H(15B)	0.9800
C(15)-H(15C)	0.9800
C(20)-H(20A)	0.9800
C(20)-H(20B)	0.9800
C(20)-H(20C)	0.9800
C(21)-H(21A)	0.9800
C(21)-H(21B)	0.9800
C(21)-H(21C)	0.9800
C(22)-C(23)	1.517(2)
C(22)-H(22A)	0.9900
C(22)-H(22B)	0.9900
C(23)-H(23A)	0.9900

C(23)-H(23B)	0.9900
C(24)-H(24A)	0.9800
C(24)-H(24B)	0.9800
C(24)-H(24C)	0.9800
C(25)-H(25A)	0.9800
C(25)-H(25B)	0.9800
C(25)-H(25C)	0.9800
C(26)-C(27)	1.519(2)
C(26)-H(26A)	0.9900
C(26)-H(26B)	0.9900
C(27)-H(27A)	0.9900
C(27)-H(27B)	0.9900
C(28)-H(28A)	0.9800
C(28)-H(28B)	0.9800
C(28)-H(28C)	0.9800
C(29)-H(29A)	0.9800
C(29)-H(29B)	0.9800
C(29)-H(29C)	0.9800
C(30)-C(31)	1.516(2)
C(30)-H(30A)	0.9900
C(30)-H(30B)	0.9900
C(31)-H(31A)	0.9900
C(31)-H(31B)	0.9900
C(1S)-C(2S)	1.3900
C(1S)-C(6S)	1.3900
C(1S)-C(7S)	1.512(5)
C(2S)-C(3S)	1.3900
C(2S)-H(2S)	0.9500
C(3S)-C(4S)	1.3900
C(3S)-H(3S)	0.9500
C(4S)-C(5S)	1.3900
C(4S)-H(4S)	0.9500
C(5S)-C(6S)	1.3900
C(5S)-H(5S)	0.9500
C(6S)-H(6S)	0.9500
C(7S)-H(7S1)	0.9800
C(7S)-H(7S2)	0.9800
C(7S)-H(7S3)	0.9800
N(1)-Li(1)-N(2)	161.56(16)

N(1)-Li(1)-Cl(1)	99.16(11)
N(2)-Li(1)-Cl(1)	99.26(11)
N(1)-Li(1)-Li(2)	152.94(14)
N(2)-Li(1)-Li(2)	45.50(8)
Cl(1)-Li(1)-Li(2)	53.82(8)
N(1)-Li(1)-Li(3)	45.19(9)
N(2)-Li(1)-Li(3)	153.08(14)
Cl(1)-Li(1)-Li(3)	54.10(8)
Li(2)-Li(1)-Li(3)	107.90(12)
N(1)-Li(1)-Si(4)	134.83(13)
N(2)-Li(1)-Si(4)	33.95(6)
Cl(1)-Li(1)-Si(4)	114.00(10)
Li(2)-Li(1)-Si(4)	66.45(8)
Li(3)-Li(1)-Si(4)	153.29(11)
N(1)-Li(1)-Si(1)	33.79(6)
N(2)-Li(1)-Si(1)	135.49(12)
Cl(1)-Li(1)-Si(1)	110.43(10)
Li(2)-Li(1)-Si(1)	148.34(12)
Li(3)-Li(1)-Si(1)	62.83(8)
Si(4)-Li(1)-Si(1)	135.54(10)
N(2)-Li(2)-N(3)	160.44(16)
N(2)-Li(2)-Cl(1)	100.50(11)
N(3)-Li(2)-Cl(1)	99.03(11)
N(2)-Li(2)-Li(1)	46.28(8)
N(3)-Li(2)-Li(1)	153.13(14)
Cl(1)-Li(2)-Li(1)	54.27(8)
N(2)-Li(2)-Li(2)#1	154.23(8)
N(3)-Li(2)-Li(2)#1	45.29(8)
Cl(1)-Li(2)-Li(2)#1	53.74(6)
Li(1)-Li(2)-Li(2)#1	107.95(8)
N(2)-Li(2)-Si(3)	33.01(6)
N(3)-Li(2)-Si(3)	135.63(12)
Cl(1)-Li(2)-Si(3)	113.61(9)
Li(1)-Li(2)-Si(3)	67.53(8)
Li(2)#1-Li(2)-Si(3)	150.67(16)
N(2)-Li(2)-Si(5)	134.50(13)
N(3)-Li(2)-Si(5)	32.84(5)
Cl(1)-Li(2)-Si(5)	111.99(9)
Li(1)-Li(2)-Si(5)	146.84(12)
Li(2)#1-Li(2)-Si(5)	65.38(7)

Si(3)-Li(2)-Si(5)	134.40(9)
N(1)#1-Li(3)-N(1)	161.1(2)
N(1)#1-Li(3)-Cl(1)	99.44(11)
N(1)-Li(3)-Cl(1)	99.44(11)
N(1)#1-Li(3)-Li(1)#1	45.56(8)
N(1)-Li(3)-Li(1)#1	153.23(18)
Cl(1)-Li(3)-Li(1)#1	54.01(8)
N(1)#1-Li(3)-Li(1)	153.23(18)
N(1)-Li(3)-Li(1)	45.56(8)
Cl(1)-Li(3)-Li(1)	54.01(8)
Li(1)#1-Li(3)-Li(1)	108.01(16)
N(1)#1-Li(3)-Si(1)#1	32.05(4)
N(1)-Li(3)-Si(1)#1	138.25(12)
Cl(1)-Li(3)-Si(1)#1	108.04(7)
Li(1)#1-Li(3)-Si(1)#1	60.25(5)
Li(1)-Li(3)-Si(1)#1	149.33(9)
N(1)#1-Li(3)-Si(1)	138.25(12)
N(1)-Li(3)-Si(1)	32.05(4)
Cl(1)-Li(3)-Si(1)	108.04(7)
Li(1)#1-Li(3)-Si(1)	149.33(9)
Li(1)-Li(3)-Si(1)	60.25(5)
Si(1)#1-Li(3)-Si(1)	143.92(14)
N(1)#1-Li(3)-Si(2)#1	29.99(5)
N(1)-Li(3)-Si(2)#1	138.42(13)
Cl(1)-Li(3)-Si(2)#1	111.12(7)
Li(1)#1-Li(3)-Si(2)#1	64.81(6)
Li(1)-Li(3)-Si(2)#1	148.12(8)
Si(1)#1-Li(3)-Si(2)#1	56.80(3)
Si(1)-Li(3)-Si(2)#1	108.93(7)
N(1)#1-Li(3)-Si(2)	138.42(13)
N(1)-Li(3)-Si(2)	29.99(5)
Cl(1)-Li(3)-Si(2)	111.12(7)
Li(1)#1-Li(3)-Si(2)	148.12(8)
Li(1)-Li(3)-Si(2)	64.81(6)
Si(1)#1-Li(3)-Si(2)	108.93(7)
Si(1)-Li(3)-Si(2)	56.80(3)
Si(2)#1-Li(3)-Si(2)	137.76(13)
N(7)-Li(4)-N(6)	114.92(13)
N(7)-Li(4)-N(4)	81.12(10)
N(6)-Li(4)-N(4)	80.72(10)

N(7)-Li(4)-N(5)	118.85(13)
N(6)-Li(4)-N(5)	118.32(12)
N(4)-Li(4)-N(5)	79.87(10)
N(7)-Li(4)-Cl(2)	100.61(11)
N(6)-Li(4)-Cl(2)	100.10(11)
N(4)-Li(4)-Cl(2)	177.49(14)
N(5)-Li(4)-Cl(2)	97.67(10)
Li(2)#1-Cl(1)-Li(2)	72.52(13)
Li(2)#1-Cl(1)-Li(1)#1	71.91(9)
Li(2)-Cl(1)-Li(1)#1	144.28(9)
Li(2)#1-Cl(1)-Li(1)	144.28(9)
Li(2)-Cl(1)-Li(1)	71.91(9)
Li(1)#1-Cl(1)-Li(1)	143.78(13)
Li(2)#1-Cl(1)-Li(3)	143.74(6)
Li(2)-Cl(1)-Li(3)	143.74(6)
Li(1)#1-Cl(1)-Li(3)	71.89(6)
Li(1)-Cl(1)-Li(3)	71.89(6)
Li(4)#2-Cl(2)-Li(4)	180.00(15)
N(1)-Si(1)-C(3)	111.86(8)
N(1)-Si(1)-C(1)	109.61(8)
C(3)-Si(1)-C(1)	106.52(9)
N(1)-Si(1)-C(2)	115.62(8)
C(3)-Si(1)-C(2)	106.38(10)
C(1)-Si(1)-C(2)	106.33(10)
C(3)-Si(1)-Li(1)	84.63(9)
C(1)-Si(1)-Li(1)	88.90(9)
C(2)-Si(1)-Li(1)	157.19(9)
C(3)-Si(1)-Li(3)	141.54(10)
C(1)-Si(1)-Li(3)	74.96(6)
C(2)-Si(1)-Li(3)	109.96(10)
Li(1)-Si(1)-Li(3)	56.92(8)
N(1)-Si(2)-C(5)	111.43(8)
N(1)-Si(2)-C(6)	115.56(8)
C(5)-Si(2)-C(6)	104.38(9)
N(1)-Si(2)-C(4)	112.99(8)
C(5)-Si(2)-C(4)	107.15(9)
C(6)-Si(2)-C(4)	104.58(9)
C(5)-Si(2)-Li(3)	96.09(8)
C(6)-Si(2)-Li(3)	151.38(6)
C(4)-Si(2)-Li(3)	87.84(9)

N(2)-Si(3)-C(7)	112.13(8)
N(2)-Si(3)-C(8)	110.72(8)
C(7)-Si(3)-C(8)	107.31(9)
N(2)-Si(3)-C(9)	115.56(9)
C(7)-Si(3)-C(9)	105.06(10)
C(8)-Si(3)-C(9)	105.48(10)
C(7)-Si(3)-Li(2)	85.65(8)
C(8)-Si(3)-Li(2)	91.51(9)
C(9)-Si(3)-Li(2)	155.71(9)
N(2)-Si(4)-C(12)	109.94(8)
N(2)-Si(4)-C(10)	111.69(8)
C(12)-Si(4)-C(10)	107.00(8)
N(2)-Si(4)-C(11)	116.26(8)
C(12)-Si(4)-C(11)	105.93(8)
C(10)-Si(4)-C(11)	105.47(8)
C(12)-Si(4)-Li(1)	88.37(8)
C(10)-Si(4)-Li(1)	84.68(8)
C(11)-Si(4)-Li(1)	158.64(8)
N(3)-Si(5)-C(15)	110.37(8)
N(3)-Si(5)-C(14)	111.28(6)
C(15)-Si(5)-C(14)	107.11(8)
N(3)-Si(5)-C(13)	116.07(7)
C(15)-Si(5)-C(13)	106.27(8)
C(14)-Si(5)-C(13)	105.21(9)
C(15)-Si(5)-Li(2)	91.91(8)
C(14)-Si(5)-Li(2)	83.96(8)
C(13)-Si(5)-Li(2)	155.69(8)
Si(2)-N(1)-Si(1)	119.01(7)
Si(2)-N(1)-Li(3)	113.34(8)
Si(1)-N(1)-Li(3)	108.67(8)
Si(2)-N(1)-Li(1)	118.05(11)
Si(1)-N(1)-Li(1)	104.32(10)
Li(3)-N(1)-Li(1)	89.25(14)
Si(4)-N(2)-Si(3)	119.14(8)
Si(4)-N(2)-Li(2)	116.83(10)
Si(3)-N(2)-Li(2)	106.32(10)
Si(4)-N(2)-Li(1)	103.40(10)
Si(3)-N(2)-Li(1)	119.31(10)
Li(2)-N(2)-Li(1)	88.23(12)
Si(5)#1-N(3)-Si(5)	118.88(10)

Si(5)#1-N(3)-Li(2)#1	106.60(8)
Si(5)-N(3)-Li(2)#1	115.90(8)
Si(5)#1-N(3)-Li(2)	115.90(8)
Si(5)-N(3)-Li(2)	106.60(8)
Li(2)#1-N(3)-Li(2)	89.43(16)
C(23)-N(4)-C(31)	111.69(13)
C(23)-N(4)-C(27)	111.62(13)
C(31)-N(4)-C(27)	111.89(13)
C(23)-N(4)-Li(4)	107.78(12)
C(31)-N(4)-Li(4)	106.55(12)
C(27)-N(4)-Li(4)	106.97(12)
C(22)-N(5)-C(21)	111.28(15)
C(22)-N(5)-C(20)	109.78(14)
C(21)-N(5)-C(20)	108.25(14)
C(22)-N(5)-Li(4)	107.09(12)
C(21)-N(5)-Li(4)	105.76(13)
C(20)-N(5)-Li(4)	114.65(13)
C(24)-N(6)-C(25)	108.24(14)
C(24)-N(6)-C(26)	111.11(13)
C(25)-N(6)-C(26)	110.17(14)
C(24)-N(6)-Li(4)	103.13(12)
C(25)-N(6)-Li(4)	116.40(13)
C(26)-N(6)-Li(4)	107.60(12)
C(29)-N(7)-C(30)	110.73(13)
C(29)-N(7)-C(28)	108.34(14)
C(30)-N(7)-C(28)	109.73(13)
C(29)-N(7)-Li(4)	108.26(13)
C(30)-N(7)-Li(4)	106.98(12)
C(28)-N(7)-Li(4)	112.81(12)
Si(1)-C(1)-H(1A)	109.5
Si(1)-C(1)-H(1B)	109.5
H(1A)-C(1)-H(1B)	109.5
Si(1)-C(1)-H(1C)	109.5
H(1A)-C(1)-H(1C)	109.5
H(1B)-C(1)-H(1C)	109.5
Si(1)-C(2)-H(2A)	109.5
Si(1)-C(2)-H(2B)	109.5
H(2A)-C(2)-H(2B)	109.5
Si(1)-C(2)-H(2C)	109.5
H(2A)-C(2)-H(2C)	109.5

H(2B)-C(2)-H(2C)	109.5
Si(1)-C(3)-H(3A)	109.5
Si(1)-C(3)-H(3B)	109.5
H(3A)-C(3)-H(3B)	109.5
Si(1)-C(3)-H(3C)	109.5
H(3A)-C(3)-H(3C)	109.5
H(3B)-C(3)-H(3C)	109.5
Si(2)-C(4)-H(4A)	109.5
Si(2)-C(4)-H(4B)	109.5
H(4A)-C(4)-H(4B)	109.5
Si(2)-C(4)-H(4C)	109.5
H(4A)-C(4)-H(4C)	109.5
H(4B)-C(4)-H(4C)	109.5
Si(2)-C(5)-H(5A)	109.5
Si(2)-C(5)-H(5B)	109.5
H(5A)-C(5)-H(5B)	109.5
Si(2)-C(5)-H(5C)	109.5
H(5A)-C(5)-H(5C)	109.5
H(5B)-C(5)-H(5C)	109.5
Si(2)-C(6)-H(6A)	109.5
Si(2)-C(6)-H(6B)	109.5
H(6A)-C(6)-H(6B)	109.5
Si(2)-C(6)-H(6C)	109.5
H(6A)-C(6)-H(6C)	109.5
H(6B)-C(6)-H(6C)	109.5
Si(3)-C(7)-H(7A)	109.5
Si(3)-C(7)-H(7B)	109.5
H(7A)-C(7)-H(7B)	109.5
Si(3)-C(7)-H(7C)	109.5
H(7A)-C(7)-H(7C)	109.5
H(7B)-C(7)-H(7C)	109.5
Si(3)-C(8)-H(8A)	109.5
Si(3)-C(8)-H(8B)	109.5
H(8A)-C(8)-H(8B)	109.5
Si(3)-C(8)-H(8C)	109.5
H(8A)-C(8)-H(8C)	109.5
H(8B)-C(8)-H(8C)	109.5
Si(3)-C(9)-H(9A)	109.5
Si(3)-C(9)-H(9B)	109.5
H(9A)-C(9)-H(9B)	109.5

Si(3)-C(9)-H(9C)	109.5
H(9A)-C(9)-H(9C)	109.5
H(9B)-C(9)-H(9C)	109.5
Si(4)-C(10)-H(10A)	109.5
Si(4)-C(10)-H(10B)	109.5
H(10A)-C(10)-H(10B)	109.5
Si(4)-C(10)-H(10C)	109.5
H(10A)-C(10)-H(10C)	109.5
H(10B)-C(10)-H(10C)	109.5
Si(4)-C(11)-H(11A)	109.5
Si(4)-C(11)-H(11B)	109.5
H(11A)-C(11)-H(11B)	109.5
Si(4)-C(11)-H(11C)	109.5
H(11A)-C(11)-H(11C)	109.5
H(11B)-C(11)-H(11C)	109.5
Si(4)-C(12)-H(12A)	109.5
Si(4)-C(12)-H(12B)	109.5
H(12A)-C(12)-H(12B)	109.5
Si(4)-C(12)-H(12C)	109.5
H(12A)-C(12)-H(12C)	109.5
H(12B)-C(12)-H(12C)	109.5
Si(5)-C(13)-H(13A)	109.5
Si(5)-C(13)-H(13B)	109.5
H(13A)-C(13)-H(13B)	109.5
Si(5)-C(13)-H(13C)	109.5
H(13A)-C(13)-H(13C)	109.5
H(13B)-C(13)-H(13C)	109.5
Si(5)-C(14)-H(14A)	109.5
Si(5)-C(14)-H(14B)	109.5
H(14A)-C(14)-H(14B)	109.5
Si(5)-C(14)-H(14C)	109.5
H(14A)-C(14)-H(14C)	109.5
H(14B)-C(14)-H(14C)	109.5
Si(5)-C(15)-H(15A)	109.5
Si(5)-C(15)-H(15B)	109.5
H(15A)-C(15)-H(15B)	109.5
Si(5)-C(15)-H(15C)	109.5
H(15A)-C(15)-H(15C)	109.5
H(15B)-C(15)-H(15C)	109.5
N(5)-C(20)-H(20A)	109.5

N(5)-C(20)-H(20B)	109.5
H(20A)-C(20)-H(20B)	109.5
N(5)-C(20)-H(20C)	109.5
H(20A)-C(20)-H(20C)	109.5
H(20B)-C(20)-H(20C)	109.5
N(5)-C(21)-H(21A)	109.5
N(5)-C(21)-H(21B)	109.5
H(21A)-C(21)-H(21B)	109.5
N(5)-C(21)-H(21C)	109.5
H(21A)-C(21)-H(21C)	109.5
H(21B)-C(21)-H(21C)	109.5
N(5)-C(22)-C(23)	112.45(14)
N(5)-C(22)-H(22A)	109.1
C(23)-C(22)-H(22A)	109.1
N(5)-C(22)-H(22B)	109.1
C(23)-C(22)-H(22B)	109.1
H(22A)-C(22)-H(22B)	107.8
N(4)-C(23)-C(22)	111.74(14)
N(4)-C(23)-H(23A)	109.3
C(22)-C(23)-H(23A)	109.3
N(4)-C(23)-H(23B)	109.3
C(22)-C(23)-H(23B)	109.3
H(23A)-C(23)-H(23B)	107.9
N(6)-C(24)-H(24A)	109.5
N(6)-C(24)-H(24B)	109.5
H(24A)-C(24)-H(24B)	109.5
N(6)-C(24)-H(24C)	109.5
H(24A)-C(24)-H(24C)	109.5
H(24B)-C(24)-H(24C)	109.5
N(6)-C(25)-H(25A)	109.5
N(6)-C(25)-H(25B)	109.5
H(25A)-C(25)-H(25B)	109.5
N(6)-C(25)-H(25C)	109.5
H(25A)-C(25)-H(25C)	109.5
H(25B)-C(25)-H(25C)	109.5
N(6)-C(26)-C(27)	112.51(14)
N(6)-C(26)-H(26A)	109.1
C(27)-C(26)-H(26A)	109.1
N(6)-C(26)-H(26B)	109.1
C(27)-C(26)-H(26B)	109.1

H(26A)-C(26)-H(26B)	107.8
N(4)-C(27)-C(26)	111.47(14)
N(4)-C(27)-H(27A)	109.3
C(26)-C(27)-H(27A)	109.3
N(4)-C(27)-H(27B)	109.3
C(26)-C(27)-H(27B)	109.3
H(27A)-C(27)-H(27B)	108.0
N(7)-C(28)-H(28A)	109.5
N(7)-C(28)-H(28B)	109.5
H(28A)-C(28)-H(28B)	109.5
N(7)-C(28)-H(28C)	109.5
H(28A)-C(28)-H(28C)	109.5
H(28B)-C(28)-H(28C)	109.5
N(7)-C(29)-H(29A)	109.5
N(7)-C(29)-H(29B)	109.5
H(29A)-C(29)-H(29B)	109.5
N(7)-C(29)-H(29C)	109.5
H(29A)-C(29)-H(29C)	109.5
H(29B)-C(29)-H(29C)	109.5
N(7)-C(30)-C(31)	112.02(13)
N(7)-C(30)-H(30A)	109.2
C(31)-C(30)-H(30A)	109.2
N(7)-C(30)-H(30B)	109.2
C(31)-C(30)-H(30B)	109.2
H(30A)-C(30)-H(30B)	107.9
N(4)-C(31)-C(30)	111.18(13)
N(4)-C(31)-H(31A)	109.4
C(30)-C(31)-H(31A)	109.4
N(4)-C(31)-H(31B)	109.4
C(30)-C(31)-H(31B)	109.4
H(31A)-C(31)-H(31B)	108.0
C(2S)-C(1S)-C(6S)	120.0
C(2S)-C(1S)-C(7S)	120.6(3)
C(6S)-C(1S)-C(7S)	119.4(3)
C(1S)-C(2S)-C(3S)	120.0
C(1S)-C(2S)-H(2S)	120.0
C(3S)-C(2S)-H(2S)	120.0
C(4S)-C(3S)-C(2S)	120.0
C(4S)-C(3S)-H(3S)	120.0
C(2S)-C(3S)-H(3S)	120.0

C(5S)-C(4S)-C(3S)	120.0
C(5S)-C(4S)-H(4S)	120.0
C(3S)-C(4S)-H(4S)	120.0
C(4S)-C(5S)-C(6S)	120.0
C(4S)-C(5S)-H(5S)	120.0
C(6S)-C(5S)-H(5S)	120.0
C(5S)-C(6S)-C(1S)	120.0
C(5S)-C(6S)-H(6S)	120.0
C(1S)-C(6S)-H(6S)	120.0

Symmetry transformations used to generate equivalent atoms:

#1 -x+2,y,-z+1/2 #2 -x+1,-y+1,-z

Table 4. Anisotropic displacement parameters ($Å^2$x 10^3) for srgr1016. The anisotropic displacement factor exponent takes the form: $-2\pi^2[h^2 a^{*2}U^{11} + ... + 2 h k a^* b^* U^{12}]$

	U^{11}	U^{22}	U^{33}	U^{23}	U^{13}	U^{12}
Li(1)	30(1)	23(1)	23(2)	0(1)	3(1)	3(1)
Li(2)	27(1)	23(1)	25(2)	1(1)	7(1)	3(1)
Li(3)	29(2)	22(2)	31(2)	0	1(2)	0
Li(4)	23(1)	21(1)	24(1)	3(1)	-4(1)	0(1)
Cl(1)	69(1)	15(1)	39(1)	0	36(1)	0
Cl(2)	29(1)	31(1)	28(1)	8(1)	-10(1)	1(1)
Si(1)	24(1)	36(1)	26(1)	-7(1)	-4(1)	10(1)
Si(2)	25(1)	21(1)	29(1)	-6(1)	-3(1)	4(1)
Si(3)	21(1)	36(1)	26(1)	5(1)	4(1)	1(1)
Si(4)	27(1)	25(1)	18(1)	1(1)	4(1)	4(1)
Si(5)	21(1)	20(1)	21(1)	-3(1)	-4(1)	2(1)
N(1)	23(1)	21(1)	25(1)	-5(1)	0(1)	4(1)
N(2)	23(1)	25(1)	17(1)	2(1)	5(1)	3(1)
N(3)	18(1)	17(1)	20(1)	0	1(1)	0
N(4)	25(1)	24(1)	23(1)	1(1)	-5(1)	1(1)
N(5)	27(1)	28(1)	27(1)	5(1)	-1(1)	6(1)
N(6)	30(1)	22(1)	21(1)	1(1)	-4(1)	0(1)
N(7)	24(1)	21(1)	29(1)	2(1)	-3(1)	-1(1)
C(1)	31(1)	51(1)	31(1)	-3(1)	-6(1)	-2(1)
C(2)	53(1)	42(1)	46(1)	-8(1)	-9(1)	28(1)
C(3)	27(1)	82(2)	35(1)	-7(1)	0(1)	10(1)
C(4)	44(1)	26(1)	56(1)	-3(1)	1(1)	-3(1)
C(5)	26(1)	37(1)	42(1)	2(1)	-6(1)	3(1)
C(6)	44(1)	34(1)	34(1)	-16(1)	-2(1)	5(1)
C(7)	38(1)	41(1)	41(1)	8(1)	1(1)	-10(1)
C(8)	36(1)	48(1)	40(1)	9(1)	-9(1)	2(1)
C(9)	30(1)	61(1)	51(1)	10(1)	19(1)	9(1)
C(10)	44(1)	36(1)	26(1)	-6(1)	5(1)	8(1)
C(11)	48(1)	35(1)	25(1)	7(1)	6(1)	3(1)
C(12)	29(1)	32(1)	34(1)	-5(1)	-2(1)	4(1)
C(13)	36(1)	29(1)	38(1)	-7(1)	-8(1)	13(1)
C(14)	44(1)	27(1)	25(1)	5(1)	-5(1)	-4(1)
C(15)	25(1)	40(1)	35(1)	-10(1)	-4(1)	-2(1)
C(20)	27(1)	46(1)	48(1)	6(1)	3(1)	3(1)
C(21)	47(1)	30(1)	41(1)	2(1)	8(1)	10(1)

C(22)	38(1)	30(1)	24(1)	5(1)	3(1)	4(1)
C(23)	37(1)	31(1)	20(1)	3(1)	-6(1)	4(1)
C(24)	36(1)	29(1)	34(1)	2(1)	-5(1)	-5(1)
C(25)	51(1)	35(1)	27(1)	0(1)	3(1)	-3(1)
C(26)	32(1)	24(1)	32(1)	-1(1)	-3(1)	7(1)
C(27)	31(1)	25(1)	29(1)	4(1)	-8(1)	7(1)
C(28)	36(1)	21(1)	45(1)	4(1)	3(1)	0(1)
C(29)	40(1)	33(1)	35(1)	2(1)	6(1)	-1(1)
C(30)	25(1)	29(1)	32(1)	-5(1)	-6(1)	-6(1)
C(31)	24(1)	36(1)	29(1)	0(1)	-10(1)	0(1)
C(1S)	36(2)	40(2)	26(2)	9(2)	4(2)	-4(2)
C(2S)	46(3)	39(2)	27(2)	-9(2)	18(2)	-11(2)
C(3S)	63(3)	36(2)	55(3)	0(2)	30(2)	2(2)
C(4S)	39(3)	63(3)	52(3)	22(3)	17(2)	19(2)
C(5S)	37(2)	54(2)	30(2)	1(2)	-2(2)	0(2)
C(6S)	36(2)	34(2)	32(2)	1(2)	7(2)	3(2)
C(7S)	41(3)	76(4)	39(3)	8(3)	-6(2)	-2(3)

Table 5. Hydrogen coordinates (x 10^4) and isotropic displacement parameters ($Å^2$x 10 ^3) for srgr1016.

	x	y	z	U(eq)
H(1A)	12296	3291	2663	57
H(1B)	11499	3172	2873	57
H(1C)	11680	3887	2624	57
H(2A)	11958	1962	2180	71
H(2B)	11354	1889	1717	71
H(2C)	11084	1911	2258	71
H(3A)	12547	3195	1666	72
H(3B)	12010	3830	1514	72
H(3C)	11894	3113	1234	72
H(4A)	9103	1970	1411	63
H(4B)	9195	2278	1953	63
H(4C)	9850	1820	1759	63
H(5A)	8724	3253	1010	53
H(5B)	9315	3855	947	53
H(5C)	8924	3783	1447	53
H(6A)	10003	2379	668	56
H(6B)	10803	2378	977	56
H(6C)	10512	3054	689	56
H(7A)	12536	6644	1780	60
H(7B)	11690	6714	1922	60
H(7C)	11868	6745	1361	60
H(8A)	12806	5441	2313	63
H(8B)	12450	4712	2160	63
H(8C)	11994	5238	2470	63
H(9A)	13019	5477	1223	69
H(9B)	12310	5506	827	69
H(9C)	12511	4810	1117	69
H(10A)	10425	4607	282	53
H(10B)	10686	4196	771	53
H(10C)	11280	4642	504	53
H(11A)	10442	6143	272	53
H(11B)	11287	6135	516	53
H(11C)	10660	6570	761	53
H(12A)	9240	5273	665	48

H(12B)	9327	5853	1078	48
H(12C)	9347	5061	1230	48
H(13A)	8626	8017	2139	53
H(13B)	9388	8112	2483	53
H(13C)	8710	7680	2672	53
H(14A)	9418	7557	1353	49
H(14B)	9912	6872	1363	49
H(14C)	10236	7546	1639	49
H(15A)	8236	6657	1656	50
H(15B)	8226	6380	2204	50
H(15C)	8710	5990	1826	50
H(20A)	4063	4392	1401	61
H(20B)	4407	3865	1034	61
H(20C)	4127	4605	844	61
H(21A)	4720	5392	1655	58
H(21B)	4814	5606	1101	58
H(21C)	5537	5545	1484	58
H(22A)	5397	3743	1603	37
H(22B)	5265	4365	1966	37
H(23A)	6422	4845	1833	35
H(23B)	6557	4059	1986	35
H(24A)	5624	2715	296	50
H(24B)	5102	3377	203	50
H(24C)	5426	3182	747	50
H(25A)	6432	3120	-271	56
H(25B)	6798	3867	-214	56
H(25C)	5919	3786	-369	56
H(26A)	7314	3553	544	36
H(26B)	6887	2843	611	36
H(27A)	6393	3239	1316	35
H(27B)	7282	3362	1378	35
H(28A)	6840	6436	571	51
H(28B)	6101	6196	814	51
H(28C)	6145	6124	236	51
H(29A)	7606	5596	217	54
H(29B)	6886	5275	-87	54
H(29C)	7422	4797	265	54
H(30A)	6943	5608	1350	35
H(30B)	7688	5619	1067	35
H(31A)	7700	4423	1024	36

H(31B)	7656	4642	1583	36
H(2S)	7173	6961	-599	44
H(3S)	8338	6569	-256	60
H(4S)	8903	7085	452	61
H(5S)	8304	7993	816	49
H(6S)	7140	8385	474	40
H(7S1)	6350	8131	-651	79
H(7S2)	6138	8359	-120	79
H(7S3)	5926	7607	-321	79

X-ray Data for **4** **(PAGE 95 – 166)**

Table 1. Crystal data and structure refinement for srgr1014.

Identification code	srgr1014	
Empirical formula	C135 H308 Br2 Li12 N18 Si20	
Formula weight	2988.86	
Temperature	123(2) K	
Wavelength	0.71073 Å	
Crystal system	Triclinic	
Space group	P 1	
Unit cell dimensions	a = 12.3602(3) Å	α= 79.344(2)°.
	b = 19.5652(5) Å	β= 76.458(2)°.
	c = 20.4985(4) Å	γ = 87.979(2)°.
Volume	4736.06(19) $Å^3$	
Z	1	
Density (calculated)	1.048 Mg/m^3	
Absorption coefficient	0.598 mm^{-1}	
F(000)	1630	
Crystal size	0.30 x 0.30 x 0.04 mm^3	
Theta range for data collection	4.08 to 26.00°.	
Index ranges	-15<=h<=15, -24<=k<=24, -25<=l<=25	
Reflections collected	59223	
Independent reflections	35427 [R(int) = 0.0359]	
Completeness to theta = 26.00°	99.6 %	
Absorption correction	Analytical	
Max. and min. transmission	0.889 and 0.732	
Refinement method	Full-matrix least-squares on F^2	
Data / restraints / parameters	35427 / 94 / 1685	
Goodness-of-fit on F^2	1.009	
Final R indices [I>2sigma(I)]	R1 = 0.0696, wR2 = 0.1975	
R indices (all data)	R1 = 0.0933, wR2 = 0.2050	
Absolute structure parameter	0.102(7)	
Largest diff. peak and hole	1.290 and -0.659 e.$Å^{-3}$	

Table 2. Atomic coordinates (x 10^4) and equivalent isotropic displacement parameters ($Å^2$x 10^3) for srgr1014. U(eq) is defined as one third of the trace of the orthogonalized U^{ij} tensor.

	x	y	z	U(eq)
Li(1)	9084(9)	8093(5)	6670(6)	38(2)
Li(2)	10730(8)	7085(5)	7094(5)	35(2)
Li(3)	10759(9)	7166(5)	8517(5)	38(2)
Li(4)	9007(8)	8171(5)	9003(5)	30(2)
Li(5)	8017(9)	8759(5)	7853(5)	35(2)
Li(6)	10741(9)	11911(6)	11003(5)	40(2)
Li(7)	9215(9)	12943(5)	11590(5)	35(2)
Li(8)	9616(9)	12975(5)	12972(5)	38(2)
Li(9)	11316(7)	11983(5)	13234(5)	30(2)
Li(10)	12082(8)	11299(6)	12003(5)	38(2)
Li(11)	5533(8)	17819(6)	12951(5)	37(2)
Li(12)	4498(9)	12296(5)	16941(5)	37(2)
Br(1)	9775(1)	8026(1)	7762(1)	40(1)
Br(1A)	9322(3)	7725(2)	7876(2)	40(1)
Br(2)	10305(1)	12015(1)	12270(1)	37(1)
Br(2A)	10815(6)	12320(4)	12123(3)	37(1)
Si(1)	6558(1)	8529(1)	6929(1)	35(1)
Si(2)	8350(1)	9588(1)	6343(1)	35(1)
Si(3)	11270(1)	7788(1)	5629(1)	30(1)
Si(4)	9397(2)	6794(1)	5978(1)	38(1)
Si(5)	12929(1)	6870(1)	7674(1)	30(1)
Si(6)	11307(1)	5727(1)	7876(1)	35(1)
Si(7)	9601(1)	6771(1)	9954(1)	33(1)
Si(8)	10969(1)	8070(1)	9655(1)	34(1)
Si(9)	6533(1)	8471(1)	9394(1)	36(1)
Si(10)	8015(1)	9704(1)	8897(1)	36(1)
Si(11)	13410(2)	11511(1)	10555(1)	38(1)
Si(12)	11548(2)	10487(1)	10883(1)	39(1)
Si(13)	10281(2)	13178(1)	10039(1)	35(1)
Si(14)	8349(2)	12215(1)	10643(1)	38(1)
Si(15)	8848(1)	14348(1)	12066(1)	33(1)
Si(16)	7280(1)	13232(1)	12850(1)	40(1)
Si(17)	11196(2)	13419(1)	13737(1)	44(1)
Si(18)	9691(2)	12244(1)	14478(1)	48(1)
Si(19)	12079(1)	10386(1)	13369(1)	37(1)

Si(20)	13753(1)	11521(1)	12935(1)	31(1)
N(1)	7874(4)	8822(2)	6861(2)	28(1)
N(2)	10151(4)	7382(2)	6214(2)	28(1)
N(3)	11594(4)	6597(2)	7797(3)	31(1)
N(4)	10139(4)	7525(3)	9412(2)	33(1)
N(5)	7727(4)	8862(2)	8874(2)	30(1)
N(6)	12102(4)	11219(3)	11011(3)	35(1)
N(7)	9557(4)	12579(3)	10702(2)	32(1)
N(8)	8586(4)	13488(2)	12380(2)	29(1)
N(9)	10447(4)	12710(3)	13732(2)	34(1)
N(10)	12461(4)	11193(2)	12951(2)	31(1)
N(11)	5629(4)	18242(3)	13825(2)	34(1)
N(12)	6447(4)	18743(2)	12400(2)	32(1)
N(13)	4019(4)	17520(3)	12643(3)	40(1)
N(14)	5950(4)	16782(3)	12917(3)	34(1)
N(15)	3482(4)	11403(3)	17440(3)	43(1)
N(16)	4501(5)	11905(3)	16021(3)	48(2)
N(17)	6020(4)	12498(3)	17180(3)	35(1)
N(18)	4060(4)	13313(3)	17124(3)	40(1)
C(1)	6469(6)	7577(4)	7301(3)	45(2)
C(2)	6091(6)	8638(4)	6105(3)	48(2)
C(3)	5480(6)	8989(4)	7505(3)	46(2)
C(4)	8906(6)	9462(4)	5447(3)	45(2)
C(5)	9509(7)	9937(4)	6637(4)	57(2)
C(6)	7260(7)	10322(4)	6307(4)	55(2)
C(7)	11609(5)	8597(3)	5892(3)	42(2)
C(8)	12542(5)	7209(4)	5569(3)	43(2)
C(9)	11097(6)	8051(4)	4722(3)	43(2)
C(10)	8353(5)	7228(4)	5499(4)	46(2)
C(11)	10244(6)	6204(4)	5409(4)	54(2)
C(12)	8603(6)	6190(3)	6733(4)	50(2)
C(13)	13078(5)	7809(3)	7245(3)	39(2)
C(14)	13312(5)	6822(4)	8520(3)	44(2)
C(15)	14041(5)	6391(4)	7135(4)	44(2)
C(16)	9763(6)	5553(4)	8263(4)	53(2)
C(17)	12086(5)	5103(3)	8422(4)	42(2)
C(18)	11632(6)	5437(3)	7024(3)	43(2)
C(19)	10663(6)	6046(4)	9927(4)	48(2)
C(20)	8382(5)	6478(4)	9683(4)	44(2)
C(21)	9093(6)	6833(4)	10889(3)	47(2)

C(22)	10086(6)	8640(4)	10214(4)	47(2)
C(23)	12012(6)	7641(4)	10168(4)	47(2)
C(24)	11862(6)	8638(4)	8904(4)	48(2)
C(25)	5265(5)	9052(4)	9497(4)	54(2)
C(26)	6714(6)	8137(4)	10284(3)	49(2)
C(27)	6150(6)	7710(3)	9072(3)	42(2)
C(28)	9533(6)	9891(4)	8561(4)	53(2)
C(29)	7238(6)	10349(4)	8362(4)	50(2)
C(30)	7672(7)	9953(4)	9780(4)	54(2)
C(31)	14545(6)	11017(4)	10903(4)	57(2)
C(32)	13767(6)	11437(5)	9628(4)	64(2)
C(33)	13532(6)	12484(4)	10551(4)	56(2)
C(34)	10402(6)	10154(4)	11628(4)	50(2)
C(35)	12537(6)	9748(4)	10785(4)	52(2)
C(36)	10932(7)	10648(4)	10100(4)	55(2)
C(37)	9408(6)	13805(4)	9578(4)	55(2)
C(38)	11186(6)	12777(4)	9354(4)	51(2)
C(39)	11177(6)	13738(4)	10336(4)	55(2)
C(40)	8048(6)	11376(3)	11249(4)	45(2)
C(41)	8359(6)	12003(4)	9810(3)	47(2)
C(42)	7104(6)	12798(4)	10839(4)	53(2)
C(43)	10364(6)	14571(4)	11848(5)	56(2)
C(44)	8345(7)	14619(4)	11261(4)	57(2)
C(45)	8167(6)	14964(4)	12641(4)	54(2)
C(46)	7131(6)	12257(3)	12896(4)	46(2)
C(47)	6057(6)	13660(4)	12549(4)	51(2)
C(48)	7004(7)	13398(4)	13759(4)	59(2)
C(49)	12013(7)	13377(5)	14407(4)	62(2)
C(50)	12269(7)	13616(4)	12889(5)	66(2)
C(51)	10322(8)	14183(4)	13830(5)	68(2)
C(52)	8979(8)	12794(5)	15125(4)	69(2)
C(53)	8613(7)	11704(5)	14342(4)	64(2)
C(54)	10602(7)	11630(5)	14943(4)	69(2)
C(55)	12498(6)	10098(4)	14195(4)	49(2)
C(56)	10547(6)	10265(4)	13546(4)	50(2)
C(57)	12680(7)	9714(4)	12810(4)	53(2)
C(58)	14900(6)	10887(4)	12798(4)	49(2)
C(59)	14207(6)	12265(3)	12181(4)	46(2)
C(60)	13782(6)	11847(4)	13726(4)	50(2)
C(61)	6580(5)	18736(3)	13584(3)	32(1)

C(62)	6702(5)	19206(4)	14084(3)	40(2)
C(63)	7675(7)	19704(4)	13839(4)	57(2)
C(64)	7658(7)	20138(4)	13177(4)	51(2)
C(65)	7544(6)	19691(3)	12647(3)	41(2)
C(66)	6548(5)	19193(3)	12895(3)	33(1)
C(67)	5791(7)	17714(4)	14413(4)	51(2)
C(68)	4556(6)	18587(4)	14046(4)	47(2)
C(69)	7521(5)	18487(3)	12038(4)	41(2)
C(70)	5917(6)	19173(3)	11875(3)	46(2)
C(71)	4126(5)	16772(3)	12604(3)	35(1)
C(72)	3048(6)	16378(4)	12745(5)	54(2)
C(73)	3198(6)	15613(4)	12648(5)	56(2)
C(74)	3884(6)	15257(4)	13127(4)	53(2)
C(75)	5006(6)	15611(3)	13016(4)	53(2)
C(76)	4896(5)	16399(3)	13041(3)	39(2)
C(77)	3134(6)	17641(4)	13226(4)	57(2)
C(78)	3784(6)	17915(4)	12013(4)	49(2)
C(79)	6640(6)	16770(4)	12236(4)	46(2)
C(80)	6599(5)	16465(3)	13434(4)	41(2)
C(81)	3247(6)	11059(4)	16911(4)	46(2)
C(82)	2887(7)	10307(4)	17152(5)	58(2)
C(83)	2649(8)	9967(5)	16597(5)	75(3)
C(84)	3633(7)	10022(4)	16012(5)	64(2)
C(85)	3935(7)	10795(4)	15713(4)	60(2)
C(86)	4198(6)	11155(4)	16274(4)	46(2)
C(87)	4162(6)	10968(4)	17856(4)	50(2)
C(88)	2459(6)	11578(4)	17939(4)	56(2)
C(89)	3654(8)	12294(4)	15684(4)	66(2)
C(90)	5574(8)	11985(4)	15535(4)	70(2)
C(91)	6149(5)	13254(3)	16911(4)	42(2)
C(92)	7190(7)	13584(4)	16989(5)	63(2)
C(93)	7255(7)	14369(4)	16728(4)	63(2)
C(94)	6215(7)	14728(4)	17081(5)	63(2)
C(95)	5185(7)	14424(4)	16947(4)	53(2)
C(96)	5093(5)	13644(3)	17199(3)	40(2)
C(97)	5928(7)	12327(4)	17922(4)	55(2)
C(98)	6954(6)	12097(4)	16858(4)	60(2)
C(99)	3131(7)	13347(4)	17720(4)	63(2)
C(100)	3695(7)	13658(5)	16524(5)	64(2)
C(300)	16233(8)	15771(4)	8807(5)	71(2)

C(301)	15929(6)	15019(4)	8932(4)	53(2)
C(302)	14838(6)	14792(4)	9003(4)	53(2)
C(303)	14543(7)	14125(5)	9083(4)	60(2)
C(304)	15363(7)	13609(4)	9111(4)	55(2)
C(305)	16467(7)	13820(5)	9049(4)	62(2)
C(306)	16709(6)	14524(4)	8974(4)	55(2)
C(310)	10030(20)	6240(9)	2878(14)	105(7)
C(311)	10062(11)	6984(4)	2875(8)	78(6)
C(312)	9613(11)	7260(6)	3457(5)	57(5)
C(313)	9632(9)	7975(6)	3430(4)	45(3)
C(314)	10099(10)	8414(4)	2820(6)	50(4)
C(315)	10548(9)	8138(7)	2238(4)	51(4)
C(316)	10529(10)	7423(7)	2265(6)	50(4)
C(320)	3432(12)	15153(7)	15084(8)	79(4)
C(321)	4565(8)	15243(6)	15105(5)	50(2)
C(322)	5483(10)	14809(6)	14839(6)	58(3)
C(323)	6588(10)	14999(6)	14855(6)	58(3)
C(324)	6816(11)	15491(6)	15128(7)	71(3)
C(325)	5936(9)	15881(6)	15373(6)	58(3)
C(326)	4806(10)	15759(5)	15374(5)	39(2)
C(331)	4455(7)	4976(4)	10868(4)	56(2)
C(330)	4501(7)	4180(4)	10966(5)	68
C(332)	3510(9)	5294(5)	10797(6)	83(3)
C(333)	3377(9)	5985(5)	10758(6)	85(3)
C(334)	4251(9)	6374(5)	10743(5)	77(2)
C(335)	5225(8)	6138(5)	10767(5)	73(2)
C(336)	5388(8)	5375(6)	10857(5)	80(2)
C(340)	670(20)	13994(11)	16114(11)	109(6)
C(341)	414(9)	12753(5)	16554(4)	42(4)
C(342)	25(9)	12201(4)	17083(5)	64(4)
C(344)	-416(9)	12324(5)	17739(4)	50(4)
C(345)	-466(10)	13000(6)	17866(4)	61(4)
C(346)	-77(11)	13552(4)	17337(5)	64(5)
C(343)	364(9)	13428(4)	16681(5)	53(4)
C(400)	9841(10)	6922(4)	3140(6)	46(4)
C(401)	10305(10)	7045(6)	2442(6)	49(4)
C(402)	10577(11)	7720(8)	2097(4)	79(6)
C(403)	10387(13)	8271(5)	2449(8)	64(5)
C(404)	9923(13)	8148(6)	3147(8)	93(6)
C(405)	9650(10)	7473(8)	3493(4)	50(4)

C(410)	44(13)	13065(6)	17297(7)	96(7)
C(411)	-479(12)	12608(7)	17881(6)	69(5)
C(412)	-560(13)	11905(7)	17865(7)	97(7)
C(413)	-119(13)	11658(6)	17264(8)	85(6)
C(414)	404(15)	12115(8)	16680(7)	119(9)
C(415)	485(15)	12818(8)	16696(6)	128(12)
C(428)	2929(18)	14942(19)	15153(18)	60
C(420)	4158(12)	14997(11)	15044(12)	48(7)
C(421)	5080(16)	14613(9)	14795(11)	58(3)
C(422)	6140(13)	14824(10)	14799(10)	29(5)
C(423)	6278(16)	15419(11)	15051(12)	94(14)
C(424)	5360(20)	15803(9)	15300(12)	54(8)
C(425)	4296(17)	15592(10)	15296(11)	52(7)
C(408)	9660(20)	6196(9)	3534(12)	100(7)
C(418)	-60(20)	13813(8)	17317(12)	109(6)

Table 3. Bond lengths [Å] and angles [°] for srgr1014.

Li(1)-N(1)	2.052(12)
Li(1)-N(2)	2.079(12)
Li(1)-Br(1A)	2.525(11)
Li(1)-Br(1)	2.556(11)
Li(1)-Li(2)	2.948(15)
Li(1)-Li(5)	2.978(15)
Li(1)-Si(2)	3.034(11)
Li(1)-Si(4)	3.103(11)
Li(1)-Si(3)	3.142(11)
Li(1)-Si(1)	3.156(11)
Li(2)-N(3)	2.067(12)
Li(2)-N(2)	2.072(11)
Li(2)-Br(1A)	2.539(10)
Li(2)-Br(1)	2.583(10)
Li(2)-Li(3)	2.958(15)
Li(2)-Si(3)	2.992(10)
Li(2)-Si(6)	2.994(10)
Li(3)-N(4)	2.056(11)
Li(3)-N(3)	2.072(11)
Li(3)-Br(1)	2.545(12)
Li(3)-Br(1A)	2.550(12)
Li(3)-Si(7)	2.930(10)
Li(3)-Si(5)	2.933(10)
Li(3)-Li(4)	2.994(14)
Li(4)-N(4)	2.072(11)
Li(4)-N(5)	2.077(11)
Li(4)-Br(1A)	2.559(10)
Li(4)-Br(1)	2.563(9)
Li(4)-Li(5)	2.936(14)
Li(4)-Si(8)	3.016(10)
Li(4)-Si(9)	3.040(10)
Li(4)-Si(10)	3.189(10)
Li(5)-N(1)	2.063(11)
Li(5)-N(5)	2.084(11)
Li(5)-Br(1A)	2.542(10)
Li(5)-Br(1)	2.550(10)
Li(5)-Si(1)	3.004(11)
Li(5)-Si(10)	3.076(11)

Li(5)-Si(2)	3.160(9)
Li(6)-N(7)	2.054(12)
Li(6)-N(6)	2.123(12)
Li(6)-Br(2)	2.572(10)
Li(6)-Br(2A)	2.589(12)
Li(6)-Li(7)	2.940(15)
Li(6)-Si(12)	2.961(11)
Li(6)-Li(10)	2.988(16)
Li(6)-Si(13)	2.998(11)
Li(7)-N(7)	2.025(11)
Li(7)-N(8)	2.086(11)
Li(7)-Br(2)	2.612(10)
Li(7)-Br(2A)	2.634(12)
Li(7)-Li(8)	3.000(15)
Li(7)-Si(14)	3.009(11)
Li(7)-Si(15)	3.068(10)
Li(7)-Si(13)	3.098(10)
Li(8)-N(9)	2.040(12)
Li(8)-N(8)	2.070(12)
Li(8)-Br(2A)	2.521(12)
Li(8)-Br(2)	2.576(11)
Li(8)-Li(9)	2.885(14)
Li(8)-Si(16)	2.973(11)
Li(8)-Si(17)	3.013(11)
Li(8)-Si(18)	3.174(10)
Li(9)-N(9)	2.033(11)
Li(9)-N(10)	2.127(10)
Li(9)-Br(2A)	2.465(10)
Li(9)-Br(2)	2.563(9)
Li(9)-Si(18)	2.968(9)
Li(9)-Li(10)	3.016(14)
Li(9)-Si(20)	3.068(9)
Li(9)-Si(17)	3.154(10)
Li(10)-N(6)	2.063(11)
Li(10)-N(10)	2.075(12)
Li(10)-Br(2A)	2.506(12)
Li(10)-Br(2)	2.567(10)
Li(10)-Si(11)	2.999(10)
Li(10)-Si(19)	3.029(11)
Li(11)-N(14)	2.084(12)

Li(11)-N(11)	2.137(12)
Li(11)-N(12)	2.148(11)
Li(11)-N(13)	2.238(12)
Li(12)-N(17)	2.120(12)
Li(12)-N(18)	2.121(12)
Li(12)-N(15)	2.137(12)
Li(12)-N(16)	2.161(12)
Br(1)-Br(1A)	0.790(3)
Br(2)-Br(2A)	0.845(8)
Si(1)-N(1)	1.711(5)
Si(1)-C(1)	1.874(7)
Si(1)-C(2)	1.882(6)
Si(1)-C(3)	1.885(7)
Si(2)-N(1)	1.703(5)
Si(2)-C(4)	1.862(6)
Si(2)-C(5)	1.873(8)
Si(2)-C(6)	1.938(7)
Si(3)-N(2)	1.712(4)
Si(3)-C(7)	1.855(7)
Si(3)-C(9)	1.895(6)
Si(3)-C(8)	1.899(7)
Si(4)-N(2)	1.708(5)
Si(4)-C(12)	1.858(7)
Si(4)-C(10)	1.885(7)
Si(4)-C(11)	1.905(7)
Si(5)-N(3)	1.700(5)
Si(5)-C(13)	1.880(6)
Si(5)-C(14)	1.887(7)
Si(5)-C(15)	1.889(7)
Si(6)-N(3)	1.720(5)
Si(6)-C(18)	1.886(7)
Si(6)-C(17)	1.895(6)
Si(6)-C(16)	1.903(7)
Si(7)-N(4)	1.723(5)
Si(7)-C(20)	1.865(7)
Si(7)-C(19)	1.897(7)
Si(7)-C(21)	1.898(6)
Si(8)-N(4)	1.718(5)
Si(8)-C(24)	1.866(7)
Si(8)-C(22)	1.884(7)

Si(8)-C(23)	1.924(7)
Si(9)-N(5)	1.715(5)
Si(9)-C(27)	1.853(7)
Si(9)-C(26)	1.885(7)
Si(9)-C(25)	1.898(7)
Si(10)-N(5)	1.710(5)
Si(10)-C(28)	1.864(7)
Si(10)-C(29)	1.902(7)
Si(10)-C(30)	1.908(7)
Si(11)-N(6)	1.724(5)
Si(11)-C(32)	1.878(8)
Si(11)-C(31)	1.878(8)
Si(11)-C(33)	1.914(7)
Si(12)-N(6)	1.699(5)
Si(12)-C(34)	1.857(7)
Si(12)-C(35)	1.869(7)
Si(12)-C(36)	1.905(7)
Si(13)-N(7)	1.714(5)
Si(13)-C(39)	1.855(7)
Si(13)-C(38)	1.858(7)
Si(13)-C(37)	1.875(8)
Si(14)-N(7)	1.715(5)
Si(14)-C(41)	1.826(7)
Si(14)-C(40)	1.859(6)
Si(14)-C(42)	1.894(7)
Si(15)-N(8)	1.701(5)
Si(15)-C(43)	1.869(7)
Si(15)-C(44)	1.877(7)
Si(15)-C(45)	1.881(7)
Si(16)-N(8)	1.713(5)
Si(16)-C(47)	1.878(7)
Si(16)-C(48)	1.900(8)
Si(16)-C(46)	1.904(7)
Si(17)-N(9)	1.698(5)
Si(17)-C(51)	1.825(8)
Si(17)-C(49)	1.876(8)
Si(17)-C(50)	1.907(8)
Si(18)-N(9)	1.708(5)
Si(18)-C(53)	1.831(9)
Si(18)-C(52)	1.898(8)

Si(18)-C(54)	1.899(8)
Si(19)-N(10)	1.677(5)
Si(19)-C(56)	1.858(7)
Si(19)-C(55)	1.867(7)
Si(19)-C(57)	1.919(8)
Si(20)-N(10)	1.733(5)
Si(20)-C(58)	1.856(7)
Si(20)-C(60)	1.857(7)
Si(20)-C(59)	1.909(7)
N(11)-C(68)	1.479(8)
N(11)-C(61)	1.481(8)
N(11)-C(67)	1.482(8)
N(12)-C(69)	1.480(8)
N(12)-C(66)	1.490(8)
N(12)-C(70)	1.501(8)
N(13)-C(78)	1.463(9)
N(13)-C(77)	1.467(9)
N(13)-C(71)	1.481(8)
N(14)-C(79)	1.460(8)
N(14)-C(76)	1.473(8)
N(14)-C(80)	1.507(8)
N(15)-C(81)	1.464(9)
N(15)-C(87)	1.475(9)
N(15)-C(88)	1.502(9)
N(16)-C(90)	1.454(10)
N(16)-C(86)	1.492(9)
N(16)-C(89)	1.498(9)
N(17)-C(98)	1.468(9)
N(17)-C(97)	1.474(9)
N(17)-C(91)	1.480(8)
N(18)-C(100)	1.449(10)
N(18)-C(99)	1.478(9)
N(18)-C(96)	1.504(8)
C(1)-H(1A)	0.9800
C(1)-H(1B)	0.9800
C(1)-H(1C)	0.9800
C(2)-H(2A)	0.9800
C(2)-H(2B)	0.9800
C(2)-H(2C)	0.9800
C(3)-H(3A)	0.9800

C(3)-H(3B)	0.9800
C(3)-H(3C)	0.9800
C(4)-H(4A)	0.9800
C(4)-H(4B)	0.9800
C(4)-H(4C)	0.9800
C(5)-H(5A)	0.9800
C(5)-H(5B)	0.9800
C(5)-H(5C)	0.9800
C(6)-H(6A)	0.9800
C(6)-H(6B)	0.9800
C(6)-H(6C)	0.9800
C(7)-H(7A)	0.9800
C(7)-H(7B)	0.9800
C(7)-H(7C)	0.9800
C(8)-H(8A)	0.9800
C(8)-H(8B)	0.9800
C(8)-H(8C)	0.9800
C(9)-H(9A)	0.9800
C(9)-H(9B)	0.9800
C(9)-H(9C)	0.9800
C(10)-H(10A)	0.9800
C(10)-H(10B)	0.9800
C(10)-H(10C)	0.9800
C(11)-H(11A)	0.9800
C(11)-H(11B)	0.9800
C(11)-H(11C)	0.9800
C(12)-H(12A)	0.9800
C(12)-H(12B)	0.9800
C(12)-H(12C)	0.9800
C(13)-H(13A)	0.9800
C(13)-H(13B)	0.9800
C(13)-H(13C)	0.9800
C(14)-H(14A)	0.9800
C(14)-H(14B)	0.9800
C(14)-H(14C)	0.9800
C(15)-H(15A)	0.9800
C(15)-H(15B)	0.9800
C(15)-H(15C)	0.9800
C(16)-H(16A)	0.9800
C(16)-H(16B)	0.9800

C(16)-H(16C)	0.9800
C(17)-H(17A)	0.9800
C(17)-H(17B)	0.9800
C(17)-H(17C)	0.9800
C(18)-H(18A)	0.9800
C(18)-H(18B)	0.9800
C(18)-H(18C)	0.9800
C(19)-H(19A)	0.9800
C(19)-H(19B)	0.9800
C(19)-H(19C)	0.9800
C(20)-H(20A)	0.9800
C(20)-H(20B)	0.9800
C(20)-H(20C)	0.9800
C(21)-H(21A)	0.9800
C(21)-H(21B)	0.9800
C(21)-H(21C)	0.9800
C(22)-H(22A)	0.9800
C(22)-H(22B)	0.9800
C(22)-H(22C)	0.9800
C(23)-H(23A)	0.9800
C(23)-H(23B)	0.9800
C(23)-H(23C)	0.9800
C(24)-H(24A)	0.9800
C(24)-H(24B)	0.9800
C(24)-H(24C)	0.9800
C(25)-H(25A)	0.9800
C(25)-H(25B)	0.9800
C(25)-H(25C)	0.9800
C(26)-H(26A)	0.9800
C(26)-H(26B)	0.9800
C(26)-H(26C)	0.9800
C(27)-H(27A)	0.9800
C(27)-H(27B)	0.9800
C(27)-H(27C)	0.9800
C(28)-H(28A)	0.9800
C(28)-H(28B)	0.9800
C(28)-H(28C)	0.9800
C(29)-H(29A)	0.9800
C(29)-H(29B)	0.9800
C(29)-H(29C)	0.9800

C(30)-H(30A)	0.9800
C(30)-H(30B)	0.9800
C(30)-H(30C)	0.9800
C(31)-H(31A)	0.9800
C(31)-H(31B)	0.9800
C(31)-H(31C)	0.9800
C(32)-H(32A)	0.9800
C(32)-H(32B)	0.9800
C(32)-H(32C)	0.9800
C(33)-H(33A)	0.9800
C(33)-H(33B)	0.9800
C(33)-H(33C)	0.9800
C(34)-H(34A)	0.9800
C(34)-H(34B)	0.9800
C(34)-H(34C)	0.9800
C(35)-H(35A)	0.9800
C(35)-H(35B)	0.9800
C(35)-H(35C)	0.9800
C(36)-H(36A)	0.9800
C(36)-H(36B)	0.9800
C(36)-H(36C)	0.9800
C(37)-H(37A)	0.9800
C(37)-H(37B)	0.9800
C(37)-H(37C)	0.9800
C(38)-H(38A)	0.9800
C(38)-H(38B)	0.9800
C(38)-H(38C)	0.9800
C(39)-H(39A)	0.9800
C(39)-H(39B)	0.9800
C(39)-H(39C)	0.9800
C(40)-H(40A)	0.9800
C(40)-H(40B)	0.9800
C(40)-H(40C)	0.9800
C(41)-H(41A)	0.9800
C(41)-H(41B)	0.9800
C(41)-H(41C)	0.9800
C(42)-H(42A)	0.9800
C(42)-H(42B)	0.9800
C(42)-H(42C)	0.9800
C(43)-H(43A)	0.9800

C(43)-H(43B)	0.9800
C(43)-H(43C)	0.9800
C(44)-H(44A)	0.9800
C(44)-H(44B)	0.9800
C(44)-H(44C)	0.9800
C(45)-H(45A)	0.9800
C(45)-H(45B)	0.9800
C(45)-H(45C)	0.9800
C(46)-H(46A)	0.9800
C(46)-H(46B)	0.9800
C(46)-H(46C)	0.9800
C(47)-H(47A)	0.9800
C(47)-H(47B)	0.9800
C(47)-H(47C)	0.9800
C(48)-H(48A)	0.9800
C(48)-H(48B)	0.9800
C(48)-H(48C)	0.9800
C(49)-H(49A)	0.9800
C(49)-H(49B)	0.9800
C(49)-H(49C)	0.9800
C(50)-H(50A)	0.9800
C(50)-H(50B)	0.9800
C(50)-H(50C)	0.9800
C(51)-H(51A)	0.9800
C(51)-H(51B)	0.9800
C(51)-H(51C)	0.9800
C(52)-H(52A)	0.9800
C(52)-H(52B)	0.9800
C(52)-H(52C)	0.9800
C(53)-H(53A)	0.9800
C(53)-H(53B)	0.9800
C(53)-H(53C)	0.9800
C(54)-H(54A)	0.9800
C(54)-H(54B)	0.9800
C(54)-H(54C)	0.9800
C(55)-H(55A)	0.9800
C(55)-H(55B)	0.9800
C(55)-H(55C)	0.9800
C(56)-H(56A)	0.9800
C(56)-H(56B)	0.9800

C(56)-H(56C)	0.9800
C(57)-H(57A)	0.9800
C(57)-H(57B)	0.9800
C(57)-H(57C)	0.9800
C(58)-H(58A)	0.9800
C(58)-H(58B)	0.9800
C(58)-H(58C)	0.9800
C(59)-H(59A)	0.9800
C(59)-H(59B)	0.9800
C(59)-H(59C)	0.9800
C(60)-H(60A)	0.9800
C(60)-H(60B)	0.9800
C(60)-H(60C)	0.9800
C(61)-C(66)	1.533(8)
C(61)-C(62)	1.534(9)
C(61)-H(61)	1.0000
C(62)-C(63)	1.505(10)
C(62)-H(62A)	0.9900
C(62)-H(62B)	0.9900
C(63)-C(64)	1.466(10)
C(63)-H(63A)	0.9900
C(63)-H(63B)	0.9900
C(64)-C(65)	1.547(9)
C(64)-H(64A)	0.9900
C(64)-H(64B)	0.9900
C(65)-C(66)	1.527(9)
C(65)-H(65A)	0.9900
C(65)-H(65B)	0.9900
C(66)-H(66)	1.0000
C(67)-H(67A)	0.9800
C(67)-H(67B)	0.9800
C(67)-H(67C)	0.9800
C(68)-H(68A)	0.9800
C(68)-H(68B)	0.9800
C(68)-H(68C)	0.9800
C(69)-H(69A)	0.9800
C(69)-H(69B)	0.9800
C(69)-H(69C)	0.9800
C(70)-H(70A)	0.9800
C(70)-H(70B)	0.9800

C(70)-H(70C)	0.9800
C(71)-C(72)	1.504(9)
C(71)-C(76)	1.531(9)
C(71)-H(71)	1.0000
C(72)-C(73)	1.546(10)
C(72)-H(72A)	0.9900
C(72)-H(72B)	0.9900
C(73)-C(74)	1.503(11)
C(73)-H(73A)	0.9900
C(73)-H(73B)	0.9900
C(74)-C(75)	1.522(10)
C(74)-H(74A)	0.9900
C(74)-H(74B)	0.9900
C(75)-C(76)	1.551(9)
C(75)-H(75A)	0.9900
C(75)-H(75B)	0.9900
C(76)-H(76)	1.0000
C(77)-H(77A)	0.9800
C(77)-H(77B)	0.9800
C(77)-H(77C)	0.9800
C(78)-H(78A)	0.9800
C(78)-H(78B)	0.9800
C(78)-H(78C)	0.9800
C(79)-H(79A)	0.9800
C(79)-H(79B)	0.9800
C(79)-H(79C)	0.9800
C(80)-H(80A)	0.9800
C(80)-H(80B)	0.9800
C(80)-H(80C)	0.9800
C(81)-C(82)	1.512(10)
C(81)-C(86)	1.524(10)
C(81)-H(81)	1.0000
C(82)-C(83)	1.507(12)
C(82)-H(82A)	0.9900
C(82)-H(82B)	0.9900
C(83)-C(84)	1.485(13)
C(83)-H(83A)	0.9900
C(83)-H(83B)	0.9900
C(84)-C(85)	1.546(12)
C(84)-H(84A)	0.9900

C(84)-H(84B)	0.9900
C(85)-C(86)	1.554(9)
C(85)-H(85A)	0.9900
C(85)-H(85B)	0.9900
C(86)-H(86)	1.0000
C(87)-H(87A)	0.9800
C(87)-H(87B)	0.9800
C(87)-H(87C)	0.9800
C(88)-H(88A)	0.9800
C(88)-H(88B)	0.9800
C(88)-H(88C)	0.9800
C(89)-H(89A)	0.9800
C(89)-H(89B)	0.9800
C(89)-H(89C)	0.9800
C(90)-H(90A)	0.9800
C(90)-H(90B)	0.9800
C(90)-H(90C)	0.9800
C(91)-C(92)	1.514(10)
C(91)-C(96)	1.539(9)
C(91)-H(91)	1.0000
C(92)-C(93)	1.527(11)
C(92)-H(92A)	0.9900
C(92)-H(92B)	0.9900
C(93)-C(94)	1.535(13)
C(93)-H(93A)	0.9900
C(93)-H(93B)	0.9900
C(94)-C(95)	1.526(11)
C(94)-H(94A)	0.9900
C(94)-H(94B)	0.9900
C(95)-C(96)	1.517(9)
C(95)-H(95A)	0.9900
C(95)-H(95B)	0.9900
C(96)-H(96)	1.0000
C(97)-H(97A)	0.9800
C(97)-H(97B)	0.9800
C(97)-H(97C)	0.9800
C(98)-H(98A)	0.9800
C(98)-H(98B)	0.9800
C(98)-H(98C)	0.9800
C(99)-H(99A)	0.9800

C(99)-H(99B)	0.9800
C(99)-H(99C)	0.9800
C(100)-H(10D)	0.9800
C(100)-H(10E)	0.9800
C(100)-H(10F)	0.9800
C(300)-C(301)	1.492(11)
C(300)-H(30D)	0.9800
C(300)-H(30E)	0.9800
C(300)-H(30F)	0.9800
C(301)-C(306)	1.349(11)
C(301)-C(302)	1.402(10)
C(302)-C(303)	1.337(11)
C(302)-H(302)	0.9500
C(303)-C(304)	1.408(12)
C(303)-H(303)	0.9500
C(304)-C(305)	1.409(12)
C(304)-H(304)	0.9500
C(305)-C(306)	1.393(11)
C(305)-H(305)	0.9500
C(306)-H(306)	0.9500
C(310)-C(311)	1.457(15)
C(310)-H(31D)	0.9800
C(310)-H(31E)	0.9800
C(310)-H(31F)	0.9800
C(311)-C(312)	1.3900
C(311)-C(316)	1.3900
C(312)-C(313)	1.3900
C(312)-H(312)	0.9500
C(313)-C(314)	1.3900
C(313)-H(313)	0.9500
C(314)-C(315)	1.3900
C(314)-H(314)	0.9500
C(315)-C(316)	1.3900
C(315)-H(315)	0.9500
C(316)-H(316)	0.9500
C(320)-C(321)	1.430(17)
C(320)-H(32D)	0.9800
C(320)-H(32E)	0.9800
C(320)-H(32F)	0.9800
C(320)-H(42D)	1.1840

C(321)-C(326)	1.309(14)
C(321)-C(322)	1.455(14)
C(322)-C(323)	1.437(16)
C(322)-H(322)	0.9500
C(323)-C(324)	1.269(17)
C(323)-H(323)	0.9500
C(324)-C(325)	1.359(16)
C(324)-H(324)	0.9500
C(325)-C(326)	1.425(15)
C(325)-H(325)	0.9500
C(326)-H(326)	0.9500
C(331)-C(332)	1.330(13)
C(331)-C(336)	1.409(13)
C(331)-C(330)	1.532(12)
C(330)-H(33D)	0.9800
C(330)-H(33E)	0.9800
C(330)-H(33F)	0.9800
C(332)-C(333)	1.346(13)
C(332)-H(332)	0.9500
C(333)-C(334)	1.334(13)
C(333)-H(333)	0.9500
C(334)-C(335)	1.283(13)
C(334)-H(334)	0.9500
C(335)-C(336)	1.481(14)
C(335)-H(335)	0.9500
C(336)-H(336)	0.9500
C(340)-C(343)	1.439(16)
C(340)-H(34D)	0.9800
C(340)-H(34E)	0.9800
C(340)-H(34F)	0.9800
C(341)-C(342)	1.3900
C(341)-C(343)	1.3900
C(341)-H(341)	0.9500
C(342)-C(344)	1.3900
C(342)-H(342)	0.9500
C(344)-C(345)	1.3900
C(344)-H(344)	0.9500
C(345)-C(346)	1.3900
C(345)-H(345)	0.9500
C(346)-C(343)	1.3900

C(346)-H(346)	0.9500
C(400)-C(401)	1.3900
C(400)-C(405)	1.3900
C(400)-C(408)	1.494(16)
C(401)-C(402)	1.3900
C(401)-H(401)	0.9500
C(402)-C(403)	1.3900
C(402)-H(402)	0.9500
C(403)-C(404)	1.3900
C(403)-H(403)	0.9500
C(404)-C(405)	1.3900
C(404)-H(404)	0.9500
C(405)-H(405)	0.9500
C(410)-C(411)	1.3900
C(410)-C(415)	1.3900
C(410)-C(418)	1.472(12)
C(411)-C(412)	1.3900
C(411)-H(411)	0.9500
C(412)-C(413)	1.3900
C(412)-H(412)	0.9500
C(413)-C(414)	1.3900
C(413)-H(413)	0.9500
C(414)-C(415)	1.3900
C(414)-H(414)	0.9500
C(415)-H(415)	0.9500
C(428)-C(420)	1.488(18)
C(428)-H(42D)	0.9800
C(428)-H(42E)	0.9800
C(428)-H(42F)	0.9800
C(420)-C(421)	1.3900
C(420)-C(425)	1.3900
C(421)-C(422)	1.3900
C(421)-H(421)	0.9500
C(422)-C(423)	1.3900
C(422)-H(422)	0.9500
C(423)-C(424)	1.3900
C(423)-H(423)	0.9500
C(424)-C(425)	1.3900
C(424)-H(424)	0.9500
C(425)-H(425)	0.9500

C(408)-H(40D)	0.9800
C(408)-H(40E)	0.9800
C(408)-H(40F)	0.9800
C(418)-H(41D)	0.9800
C(418)-H(41E)	0.9800
C(418)-H(41F)	0.9800

N(1)-Li(1)-N(2)	163.2(6)
N(1)-Li(1)-Br(1A)	97.6(4)
N(2)-Li(1)-Br(1A)	98.6(4)
N(1)-Li(1)-Br(1)	97.3(4)
N(2)-Li(1)-Br(1)	99.5(4)
Br(1A)-Li(1)-Br(1)	17.87(11)
N(1)-Li(1)-Li(2)	152.0(6)
N(2)-Li(1)-Li(2)	44.7(3)
Br(1A)-Li(1)-Li(2)	54.6(3)
Br(1)-Li(1)-Li(2)	55.4(3)
N(1)-Li(1)-Li(5)	43.8(3)
N(2)-Li(1)-Li(5)	152.8(6)
Br(1A)-Li(1)-Li(5)	54.3(3)
Br(1)-Li(1)-Li(5)	54.2(3)
Li(2)-Li(1)-Li(5)	108.2(4)
N(1)-Li(1)-Si(2)	32.4(2)
N(2)-Li(1)-Si(2)	139.5(5)
Br(1A)-Li(1)-Si(2)	114.1(4)
Br(1)-Li(1)-Si(2)	104.6(4)
Li(2)-Li(1)-Si(2)	149.6(4)
Li(5)-Li(1)-Si(2)	63.4(3)
N(1)-Li(1)-Si(4)	136.7(5)
N(2)-Li(1)-Si(4)	31.2(2)
Br(1A)-Li(1)-Si(4)	108.2(4)
Br(1)-Li(1)-Si(4)	117.7(4)
Li(2)-Li(1)-Si(4)	65.0(3)
Li(5)-Li(1)-Si(4)	149.4(4)
Si(2)-Li(1)-Si(4)	137.3(4)
N(1)-Li(1)-Si(3)	143.4(5)
N(2)-Li(1)-Si(3)	30.45(19)
Br(1A)-Li(1)-Si(3)	109.9(4)
Br(1)-Li(1)-Si(3)	101.7(3)
Li(2)-Li(1)-Si(3)	58.7(3)

Li(5)-Li(1)-Si(3)	148.2(4)
Si(2)-Li(1)-Si(3)	111.6(3)
Si(4)-Li(1)-Si(3)	56.30(19)
N(1)-Li(1)-Si(1)	29.8(2)
N(2)-Li(1)-Si(1)	140.2(5)
Br(1A)-Li(1)-Si(1)	101.5(3)
Br(1)-Li(1)-Si(1)	109.9(4)
Li(2)-Li(1)-Si(1)	146.2(4)
Li(5)-Li(1)-Si(1)	58.6(3)
Si(2)-Li(1)-Si(1)	57.25(19)
Si(4)-Li(1)-Si(1)	109.2(3)
Si(3)-Li(1)-Si(1)	148.2(4)
N(3)-Li(2)-N(2)	162.9(6)
N(3)-Li(2)-Br(1A)	98.5(4)
N(2)-Li(2)-Br(1A)	98.3(4)
N(3)-Li(2)-Br(1)	98.0(4)
N(2)-Li(2)-Br(1)	98.9(4)
Br(1A)-Li(2)-Br(1)	17.71(10)
N(3)-Li(2)-Li(1)	152.2(5)
N(2)-Li(2)-Li(1)	44.8(3)
Br(1A)-Li(2)-Li(1)	54.2(3)
Br(1)-Li(2)-Li(1)	54.5(3)
N(3)-Li(2)-Li(3)	44.5(3)
N(2)-Li(2)-Li(3)	152.6(5)
Br(1A)-Li(2)-Li(3)	54.6(3)
Br(1)-Li(2)-Li(3)	54.2(3)
Li(1)-Li(2)-Li(3)	107.8(4)
N(3)-Li(2)-Si(3)	137.1(4)
N(2)-Li(2)-Si(3)	33.71(19)
Br(1A)-Li(2)-Si(3)	114.2(4)
Br(1)-Li(2)-Si(3)	105.1(3)
Li(1)-Li(2)-Si(3)	63.9(3)
Li(3)-Li(2)-Si(3)	147.0(4)
N(3)-Li(2)-Si(6)	33.9(2)
N(2)-Li(2)-Si(6)	134.9(5)
Br(1A)-Li(2)-Si(6)	110.0(3)
Br(1)-Li(2)-Si(6)	118.7(3)
Li(1)-Li(2)-Si(6)	151.2(4)
Li(3)-Li(2)-Si(6)	67.4(3)
Si(3)-Li(2)-Si(6)	135.5(3)

N(4)-Li(3)-N(3)	163.5(7)
N(4)-Li(3)-Br(1)	97.4(4)
N(3)-Li(3)-Br(1)	99.1(4)
N(4)-Li(3)-Br(1A)	97.5(4)
N(3)-Li(3)-Br(1A)	98.0(4)
Br(1)-Li(3)-Br(1A)	17.83(11)
N(4)-Li(3)-Si(7)	35.2(2)
N(3)-Li(3)-Si(7)	132.8(5)
Br(1)-Li(3)-Si(7)	116.7(4)
Br(1A)-Li(3)-Si(7)	107.0(4)
N(4)-Li(3)-Si(5)	138.4(5)
N(3)-Li(3)-Si(5)	34.6(2)
Br(1)-Li(3)-Si(5)	107.4(3)
Br(1A)-Li(3)-Si(5)	116.2(4)
Si(7)-Li(3)-Si(5)	135.8(4)
N(4)-Li(3)-Li(2)	151.7(6)
N(3)-Li(3)-Li(2)	44.3(3)
Br(1)-Li(3)-Li(2)	55.4(3)
Br(1A)-Li(3)-Li(2)	54.3(3)
Si(7)-Li(3)-Li(2)	145.3(4)
Si(5)-Li(3)-Li(2)	65.8(3)
N(4)-Li(3)-Li(4)	43.7(3)
N(3)-Li(3)-Li(4)	152.3(6)
Br(1)-Li(3)-Li(4)	54.4(3)
Br(1A)-Li(3)-Li(4)	54.3(3)
Si(7)-Li(3)-Li(4)	65.9(3)
Si(5)-Li(3)-Li(4)	150.9(4)
Li(2)-Li(3)-Li(4)	108.1(5)
N(4)-Li(4)-N(5)	163.9(5)
N(4)-Li(4)-Br(1A)	96.8(4)
N(5)-Li(4)-Br(1A)	98.9(4)
N(4)-Li(4)-Br(1)	96.5(4)
N(5)-Li(4)-Br(1)	99.6(4)
Br(1A)-Li(4)-Br(1)	17.74(10)
N(4)-Li(4)-Li(5)	150.9(5)
N(5)-Li(4)-Li(5)	45.2(3)
Br(1A)-Li(4)-Li(5)	54.6(3)
Br(1)-Li(4)-Li(5)	54.7(3)
N(4)-Li(4)-Li(3)	43.3(3)
N(5)-Li(4)-Li(3)	152.7(5)

Br(1A)-Li(4)-Li(3)	54.0(3)
Br(1)-Li(4)-Li(3)	53.9(3)
Li(5)-Li(4)-Li(3)	107.6(4)
N(4)-Li(4)-Si(8)	33.4(2)
N(5)-Li(4)-Si(8)	137.7(4)
Br(1A)-Li(4)-Si(8)	115.9(3)
Br(1)-Li(4)-Si(8)	106.6(3)
Li(5)-Li(4)-Si(8)	149.0(4)
Li(3)-Li(4)-Si(8)	65.2(3)
N(4)-Li(4)-Si(9)	137.2(4)
N(5)-Li(4)-Si(9)	32.8(2)
Br(1A)-Li(4)-Si(9)	105.7(3)
Br(1)-Li(4)-Si(9)	115.7(4)
Li(5)-Li(4)-Si(9)	65.1(3)
Li(3)-Li(4)-Si(9)	146.6(4)
Si(8)-Li(4)-Si(9)	137.7(3)
N(4)-Li(4)-Si(10)	143.1(4)
N(5)-Li(4)-Si(10)	29.2(2)
Br(1A)-Li(4)-Si(10)	112.5(3)
Br(1)-Li(4)-Si(10)	104.7(3)
Li(5)-Li(4)-Si(10)	60.1(3)
Li(3)-Li(4)-Si(10)	150.6(4)
Si(8)-Li(4)-Si(10)	110.4(3)
Si(9)-Li(4)-Si(10)	56.63(17)
N(1)-Li(5)-N(5)	163.0(6)
N(1)-Li(5)-Br(1A)	96.8(4)
N(5)-Li(5)-Br(1A)	99.2(4)
N(1)-Li(5)-Br(1)	97.2(4)
N(5)-Li(5)-Br(1)	99.8(4)
Br(1A)-Li(5)-Br(1)	17.85(10)
N(1)-Li(5)-Li(4)	151.7(5)
N(5)-Li(5)-Li(4)	45.0(3)
Br(1A)-Li(5)-Li(4)	55.1(3)
Br(1)-Li(5)-Li(4)	55.2(3)
N(1)-Li(5)-Li(1)	43.5(3)
N(5)-Li(5)-Li(1)	152.9(5)
Br(1A)-Li(5)-Li(1)	53.8(3)
Br(1)-Li(5)-Li(1)	54.4(3)
Li(4)-Li(5)-Li(1)	108.2(4)
N(1)-Li(5)-Si(1)	33.4(2)

N(5)-Li(5)-Si(1)	134.5(5)
Br(1A)-Li(5)-Si(1)	105.3(4)
Br(1)-Li(5)-Si(1)	114.9(4)
Li(4)-Li(5)-Si(1)	147.4(4)
Li(1)-Li(5)-Si(1)	63.7(3)
N(1)-Li(5)-Si(10)	140.1(4)
N(5)-Li(5)-Si(10)	31.9(2)
Br(1A)-Li(5)-Si(10)	116.7(4)
Br(1)-Li(5)-Si(10)	108.3(4)
Li(4)-Li(5)-Si(10)	64.0(3)
Li(1)-Li(5)-Si(10)	153.5(4)
Si(1)-Li(5)-Si(10)	136.8(4)
N(1)-Li(5)-Si(2)	29.56(19)
N(5)-Li(5)-Si(2)	144.2(5)
Br(1A)-Li(5)-Si(2)	109.6(3)
Br(1)-Li(5)-Si(2)	101.3(3)
Li(4)-Li(5)-Si(2)	148.4(4)
Li(1)-Li(5)-Si(2)	59.2(3)
Si(1)-Li(5)-Si(2)	57.48(18)
Si(10)-Li(5)-Si(2)	113.1(3)
N(7)-Li(6)-N(6)	163.6(6)
N(7)-Li(6)-Br(2)	98.6(4)
N(6)-Li(6)-Br(2)	97.7(4)
N(7)-Li(6)-Br(2A)	99.6(4)
N(6)-Li(6)-Br(2A)	95.4(4)
Br(2)-Li(6)-Br(2A)	18.84(19)
N(7)-Li(6)-Li(7)	43.5(3)
N(6)-Li(6)-Li(7)	151.8(5)
Br(2)-Li(6)-Li(7)	56.1(3)
Br(2A)-Li(6)-Li(7)	56.5(3)
N(7)-Li(6)-Si(12)	138.6(5)
N(6)-Li(6)-Si(12)	34.3(2)
Br(2)-Li(6)-Si(12)	108.3(4)
Br(2A)-Li(6)-Si(12)	116.1(4)
Li(7)-Li(6)-Si(12)	154.7(4)
N(7)-Li(6)-Li(10)	152.2(5)
N(6)-Li(6)-Li(10)	43.6(3)
Br(2)-Li(6)-Li(10)	54.4(3)
Br(2A)-Li(6)-Li(10)	52.8(3)
Li(7)-Li(6)-Li(10)	108.7(4)

Si(12)-Li(6)-Li(10)	65.5(3)
N(7)-Li(6)-Si(13)	33.5(2)
N(6)-Li(6)-Si(13)	134.4(4)
Br(2)-Li(6)-Si(13)	115.8(4)
Br(2A)-Li(6)-Si(13)	106.5(4)
Li(7)-Li(6)-Si(13)	62.9(3)
Si(12)-Li(6)-Si(13)	135.9(4)
Li(10)-Li(6)-Si(13)	146.3(5)
N(7)-Li(7)-N(8)	165.0(6)
N(7)-Li(7)-Br(2)	98.1(4)
N(8)-Li(7)-Br(2)	96.7(4)
N(7)-Li(7)-Br(2A)	98.9(4)
N(8)-Li(7)-Br(2A)	95.9(4)
Br(2)-Li(7)-Br(2A)	18.53(19)
N(7)-Li(7)-Li(6)	44.3(3)
N(8)-Li(7)-Li(6)	150.7(5)
Br(2)-Li(7)-Li(6)	54.8(3)
Br(2A)-Li(7)-Li(6)	55.0(3)
N(7)-Li(7)-Li(8)	151.4(5)
N(8)-Li(7)-Li(8)	43.6(3)
Br(2)-Li(7)-Li(8)	54.1(3)
Br(2A)-Li(7)-Li(8)	52.7(3)
Li(6)-Li(7)-Li(8)	107.1(4)
N(7)-Li(7)-Si(14)	33.1(2)
N(8)-Li(7)-Si(14)	138.4(5)
Br(2)-Li(7)-Si(14)	106.6(3)
Br(2A)-Li(7)-Si(14)	116.8(4)
Li(6)-Li(7)-Si(14)	65.5(3)
Li(8)-Li(7)-Si(14)	150.5(4)
N(7)-Li(7)-Si(15)	138.3(5)
N(8)-Li(7)-Si(15)	31.9(2)
Br(2)-Li(7)-Si(15)	116.2(4)
Br(2A)-Li(7)-Si(15)	106.6(4)
Li(6)-Li(7)-Si(15)	149.6(4)
Li(8)-Li(7)-Si(15)	64.1(3)
Si(14)-Li(7)-Si(15)	136.4(4)
N(7)-Li(7)-Si(13)	30.9(2)
N(8)-Li(7)-Si(13)	141.4(5)
Br(2)-Li(7)-Si(13)	111.3(3)
Br(2A)-Li(7)-Si(13)	102.6(3)

Li(6)-Li(7)-Si(13)	59.5(3)
Li(8)-Li(7)-Si(13)	144.9(4)
Si(14)-Li(7)-Si(13)	58.30(19)
Si(15)-Li(7)-Si(13)	109.7(3)
N(9)-Li(8)-N(8)	161.8(6)
N(9)-Li(8)-Br(2A)	97.5(4)
N(8)-Li(8)-Br(2A)	99.8(4)
N(9)-Li(8)-Br(2)	100.0(4)
N(8)-Li(8)-Br(2)	98.2(4)
Br(2A)-Li(8)-Br(2)	19.0(2)
N(9)-Li(8)-Li(9)	44.8(3)
N(8)-Li(8)-Li(9)	153.2(5)
Br(2A)-Li(8)-Li(9)	53.7(3)
Br(2)-Li(8)-Li(9)	55.6(3)
N(9)-Li(8)-Si(16)	137.8(5)
N(8)-Li(8)-Si(16)	34.1(2)
Br(2A)-Li(8)-Si(16)	116.5(4)
Br(2)-Li(8)-Si(16)	104.8(4)
Li(9)-Li(8)-Si(16)	148.1(4)
N(9)-Li(8)-Li(7)	153.7(5)
N(8)-Li(8)-Li(7)	44.0(3)
Br(2A)-Li(8)-Li(7)	56.2(3)
Br(2)-Li(8)-Li(7)	55.2(3)
Li(9)-Li(8)-Li(7)	109.2(4)
Si(16)-Li(8)-Li(7)	64.9(3)
N(9)-Li(8)-Si(17)	32.6(2)
N(8)-Li(8)-Si(17)	135.1(5)
Br(2A)-Li(8)-Si(17)	104.4(4)
Br(2)-Li(8)-Si(17)	116.3(4)
Li(9)-Li(8)-Si(17)	64.6(3)
Si(16)-Li(8)-Si(17)	138.6(4)
Li(7)-Li(8)-Si(17)	145.3(4)
N(9)-Li(8)-Si(18)	29.1(2)
N(8)-Li(8)-Si(18)	143.6(5)
Br(2A)-Li(8)-Si(18)	109.8(4)
Br(2)-Li(8)-Si(18)	102.9(3)
Li(9)-Li(8)-Si(18)	58.4(3)
Si(16)-Li(8)-Si(18)	110.8(3)
Li(7)-Li(8)-Si(18)	151.7(4)
Si(17)-Li(8)-Si(18)	57.0(2)

N(9)-Li(9)-N(10)	163.3(5)
N(9)-Li(9)-Br(2A)	99.5(4)
N(10)-Li(9)-Br(2A)	96.5(4)
N(9)-Li(9)-Br(2)	100.6(4)
N(10)-Li(9)-Br(2)	96.1(4)
Br(2A)-Li(9)-Br(2)	19.2(2)
N(9)-Li(9)-Li(8)	45.0(3)
N(10)-Li(9)-Li(8)	151.6(5)
Br(2A)-Li(9)-Li(8)	55.6(3)
Br(2)-Li(9)-Li(8)	56.1(3)
N(9)-Li(9)-Si(18)	33.8(2)
N(10)-Li(9)-Si(18)	137.3(4)
Br(2A)-Li(9)-Si(18)	118.5(4)
Br(2)-Li(9)-Si(18)	109.2(3)
Li(8)-Li(9)-Si(18)	65.7(3)
N(9)-Li(9)-Li(10)	152.8(5)
N(10)-Li(9)-Li(10)	43.5(3)
Br(2A)-Li(9)-Li(10)	53.3(3)
Br(2)-Li(9)-Li(10)	54.1(3)
Li(8)-Li(9)-Li(10)	108.2(4)
Si(18)-Li(9)-Li(10)	153.7(4)
N(9)-Li(9)-Si(20)	135.1(4)
N(10)-Li(9)-Si(20)	33.09(19)
Br(2A)-Li(9)-Si(20)	106.8(3)
Br(2)-Li(9)-Si(20)	116.0(3)
Li(8)-Li(9)-Si(20)	150.3(4)
Si(18)-Li(9)-Si(20)	134.2(3)
Li(10)-Li(9)-Si(20)	63.7(3)
N(9)-Li(9)-Si(17)	29.2(2)
N(10)-Li(9)-Si(17)	141.3(4)
Br(2A)-Li(9)-Si(17)	101.9(3)
Br(2)-Li(9)-Si(17)	112.1(3)
Li(8)-Li(9)-Si(17)	59.7(3)
Si(18)-Li(9)-Si(17)	57.67(19)
Li(10)-Li(9)-Si(17)	143.5(4)
Si(20)-Li(9)-Si(17)	108.4(3)
N(6)-Li(10)-N(10)	163.3(6)
N(6)-Li(10)-Br(2A)	99.5(5)
N(10)-Li(10)-Br(2A)	96.6(4)
N(6)-Li(10)-Br(2)	99.4(4)

N(10)-Li(10)-Br(2)	97.2(4)
Br(2A)-Li(10)-Br(2)	19.1(2)
N(6)-Li(10)-Li(6)	45.3(3)
N(10)-Li(10)-Li(6)	151.4(5)
Br(2A)-Li(10)-Li(6)	55.4(3)
Br(2)-Li(10)-Li(6)	54.5(3)
N(6)-Li(10)-Si(11)	33.8(2)
N(10)-Li(10)-Si(11)	135.1(5)
Br(2A)-Li(10)-Si(11)	107.6(4)
Br(2)-Li(10)-Si(11)	117.8(4)
Li(6)-Li(10)-Si(11)	67.3(3)
N(6)-Li(10)-Li(9)	151.5(6)
N(10)-Li(10)-Li(9)	44.8(3)
Br(2A)-Li(10)-Li(9)	52.0(3)
Br(2)-Li(10)-Li(9)	53.9(3)
Li(6)-Li(10)-Li(9)	106.7(4)
Si(11)-Li(10)-Li(9)	144.3(5)
N(6)-Li(10)-Si(19)	140.3(5)
N(10)-Li(10)-Si(19)	31.9(2)
Br(2A)-Li(10)-Si(19)	112.0(4)
Br(2)-Li(10)-Si(19)	102.8(3)
Li(6)-Li(10)-Si(19)	146.6(4)
Si(11)-Li(10)-Si(19)	139.3(4)
Li(9)-Li(10)-Si(19)	64.2(3)
N(14)-Li(11)-N(11)	119.7(5)
N(14)-Li(11)-N(12)	128.8(5)
N(11)-Li(11)-N(12)	83.5(4)
N(14)-Li(11)-N(13)	82.8(4)
N(11)-Li(11)-N(13)	128.6(5)
N(12)-Li(11)-N(13)	119.4(5)
N(17)-Li(12)-N(18)	85.0(4)
N(17)-Li(12)-N(15)	122.9(5)
N(18)-Li(12)-N(15)	122.7(5)
N(17)-Li(12)-N(16)	120.3(5)
N(18)-Li(12)-N(16)	126.5(5)
N(15)-Li(12)-N(16)	84.3(5)
Br(1A)-Br(1)-Li(3)	81.4(3)
Br(1A)-Br(1)-Li(5)	80.5(3)
Li(3)-Br(1)-Li(5)	139.8(3)
Br(1A)-Br(1)-Li(1)	78.9(3)

Li(3)-Br(1)-Li(1)	138.6(3)
Li(5)-Br(1)-Li(1)	71.4(3)
Br(1A)-Br(1)-Li(4)	80.9(3)
Li(3)-Br(1)-Li(4)	71.8(3)
Li(5)-Br(1)-Li(4)	70.1(3)
Li(1)-Br(1)-Li(4)	138.7(3)
Br(1A)-Br(1)-Li(2)	77.9(3)
Li(3)-Br(1)-Li(2)	70.4(3)
Li(5)-Br(1)-Li(2)	138.6(3)
Li(1)-Br(1)-Li(2)	70.0(3)
Li(4)-Br(1)-Li(2)	138.8(3)
Br(1)-Br(1A)-Li(1)	83.2(3)
Br(1)-Br(1A)-Li(2)	84.3(3)
Li(1)-Br(1A)-Li(2)	71.2(3)
Br(1)-Br(1A)-Li(5)	81.6(3)
Li(1)-Br(1A)-Li(5)	72.0(3)
Li(2)-Br(1A)-Li(5)	141.8(3)
Br(1)-Br(1A)-Li(3)	80.7(4)
Li(1)-Br(1A)-Li(3)	140.2(4)
Li(2)-Br(1A)-Li(3)	71.1(3)
Li(5)-Br(1A)-Li(3)	140.0(4)
Br(1)-Br(1A)-Li(4)	81.4(3)
Li(1)-Br(1A)-Li(4)	140.8(4)
Li(2)-Br(1A)-Li(4)	141.8(4)
Li(5)-Br(1A)-Li(4)	70.3(3)
Li(3)-Br(1A)-Li(4)	71.7(3)
Br(2A)-Br(2)-Li(9)	73.8(4)
Br(2A)-Br(2)-Li(10)	76.3(4)
Li(9)-Br(2)-Li(10)	72.0(3)
Br(2A)-Br(2)-Li(6)	81.7(4)
Li(9)-Br(2)-Li(6)	139.5(3)
Li(10)-Br(2)-Li(6)	71.1(3)
Br(2A)-Br(2)-Li(8)	76.8(4)
Li(9)-Br(2)-Li(8)	68.3(3)
Li(10)-Br(2)-Li(8)	136.8(4)
Li(6)-Br(2)-Li(8)	136.3(3)
Br(2A)-Br(2)-Li(7)	82.2(4)
Li(9)-Br(2)-Li(7)	136.0(3)
Li(10)-Br(2)-Li(7)	136.9(3)
Li(6)-Br(2)-Li(7)	69.1(3)

Li(8)-Br(2)-Li(7)	70.6(3)
Br(2)-Br(2A)-Li(9)	87.0(5)
Br(2)-Br(2A)-Li(10)	84.5(5)
Li(9)-Br(2A)-Li(10)	74.7(4)
Br(2)-Br(2A)-Li(8)	84.2(5)
Li(9)-Br(2A)-Li(8)	70.7(4)
Li(10)-Br(2A)-Li(8)	144.0(4)
Br(2)-Br(2A)-Li(6)	79.4(5)
Li(9)-Br(2A)-Li(6)	144.8(4)
Li(10)-Br(2A)-Li(6)	71.8(4)
Li(8)-Br(2A)-Li(6)	138.5(4)
Br(2)-Br(2A)-Li(7)	79.3(5)
Li(9)-Br(2A)-Li(7)	140.4(4)
Li(10)-Br(2A)-Li(7)	139.2(4)
Li(8)-Br(2A)-Li(7)	71.1(4)
Li(6)-Br(2A)-Li(7)	68.5(4)
N(1)-Si(1)-C(1)	108.9(3)
N(1)-Si(1)-C(2)	115.6(3)
C(1)-Si(1)-C(2)	107.0(3)
N(1)-Si(1)-C(3)	111.7(3)
C(1)-Si(1)-C(3)	108.1(3)
C(2)-Si(1)-C(3)	105.2(3)
C(1)-Si(1)-Li(5)	90.3(3)
C(2)-Si(1)-Li(5)	156.2(3)
C(3)-Si(1)-Li(5)	83.9(3)
C(1)-Si(1)-Li(1)	77.6(3)
C(2)-Si(1)-Li(1)	109.3(3)
C(3)-Si(1)-Li(1)	141.6(3)
Li(5)-Si(1)-Li(1)	57.7(3)
N(1)-Si(2)-C(4)	110.9(3)
N(1)-Si(2)-C(5)	109.7(3)
C(4)-Si(2)-C(5)	107.8(3)
N(1)-Si(2)-C(6)	115.5(3)
C(4)-Si(2)-C(6)	106.3(3)
C(5)-Si(2)-C(6)	106.3(4)
C(4)-Si(2)-Li(1)	82.7(3)
C(5)-Si(2)-Li(1)	93.1(3)
C(6)-Si(2)-Li(1)	154.3(3)
C(4)-Si(2)-Li(5)	140.2(3)
C(5)-Si(2)-Li(5)	77.6(3)

C(6)-Si(2)-Li(5)	109.8(3)
Li(1)-Si(2)-Li(5)	57.4(3)
N(2)-Si(3)-C(7)	109.6(3)
N(2)-Si(3)-C(9)	116.1(3)
C(7)-Si(3)-C(9)	106.2(3)
N(2)-Si(3)-C(8)	111.0(3)
C(7)-Si(3)-C(8)	108.1(3)
C(9)-Si(3)-C(8)	105.4(3)
C(7)-Si(3)-Li(2)	89.4(3)
C(9)-Si(3)-Li(2)	157.8(3)
C(8)-Si(3)-Li(2)	83.8(3)
C(7)-Si(3)-Li(1)	76.4(3)
C(9)-Si(3)-Li(1)	110.3(3)
C(8)-Si(3)-Li(1)	141.1(3)
Li(2)-Si(3)-Li(1)	57.4(3)
N(2)-Si(4)-C(12)	111.3(3)
N(2)-Si(4)-C(10)	112.2(3)
C(12)-Si(4)-C(10)	106.9(3)
N(2)-Si(4)-C(11)	115.5(3)
C(12)-Si(4)-C(11)	104.8(4)
C(10)-Si(4)-C(11)	105.6(3)
C(12)-Si(4)-Li(1)	95.4(3)
C(10)-Si(4)-Li(1)	84.5(3)
C(11)-Si(4)-Li(1)	153.3(3)
N(3)-Si(5)-C(13)	110.0(3)
N(3)-Si(5)-C(14)	110.4(3)
C(13)-Si(5)-C(14)	106.4(3)
N(3)-Si(5)-C(15)	116.8(3)
C(13)-Si(5)-C(15)	105.8(3)
C(14)-Si(5)-C(15)	107.0(3)
C(13)-Si(5)-Li(3)	90.0(3)
C(14)-Si(5)-Li(3)	80.2(3)
C(15)-Si(5)-Li(3)	159.5(3)
N(3)-Si(6)-C(18)	112.0(3)
N(3)-Si(6)-C(17)	116.0(3)
C(18)-Si(6)-C(17)	104.9(3)
N(3)-Si(6)-C(16)	109.8(3)
C(18)-Si(6)-C(16)	106.9(3)
C(17)-Si(6)-C(16)	106.7(3)
C(18)-Si(6)-Li(2)	84.8(3)

C(17)-Si(6)-Li(2)	157.6(3)
C(16)-Si(6)-Li(2)	89.1(3)
N(4)-Si(7)-C(20)	109.1(3)
N(4)-Si(7)-C(19)	111.5(3)
C(20)-Si(7)-C(19)	107.9(3)
N(4)-Si(7)-C(21)	116.1(3)
C(20)-Si(7)-C(21)	106.3(3)
C(19)-Si(7)-C(21)	105.6(3)
C(20)-Si(7)-Li(3)	89.4(3)
C(19)-Si(7)-Li(3)	82.3(3)
C(21)-Si(7)-Li(3)	158.7(3)
N(4)-Si(8)-C(24)	111.8(3)
N(4)-Si(8)-C(22)	110.2(3)
C(24)-Si(8)-C(22)	108.6(3)
N(4)-Si(8)-C(23)	117.0(3)
C(24)-Si(8)-C(23)	103.7(3)
C(22)-Si(8)-C(23)	105.0(3)
C(24)-Si(8)-Li(4)	92.9(3)
C(22)-Si(8)-Li(4)	83.1(3)
C(23)-Si(8)-Li(4)	157.8(3)
N(5)-Si(9)-C(27)	110.9(3)
N(5)-Si(9)-C(26)	111.4(3)
C(27)-Si(9)-C(26)	106.6(3)
N(5)-Si(9)-C(25)	115.1(3)
C(27)-Si(9)-C(25)	106.7(3)
C(26)-Si(9)-C(25)	105.6(3)
C(27)-Si(9)-Li(4)	92.9(3)
C(26)-Si(9)-Li(4)	83.0(3)
C(25)-Si(9)-Li(4)	154.8(3)
N(5)-Si(10)-C(28)	110.4(3)
N(5)-Si(10)-C(29)	112.0(3)
C(28)-Si(10)-C(29)	107.7(3)
N(5)-Si(10)-C(30)	115.8(3)
C(28)-Si(10)-C(30)	104.9(4)
C(29)-Si(10)-C(30)	105.6(3)
C(28)-Si(10)-Li(5)	91.7(3)
C(29)-Si(10)-Li(5)	85.7(3)
C(30)-Si(10)-Li(5)	155.5(3)
C(28)-Si(10)-Li(4)	78.7(3)
C(29)-Si(10)-Li(4)	141.5(3)

C(30)-Si(10)-Li(4)	109.3(3)
Li(5)-Si(10)-Li(4)	55.9(3)
N(6)-Si(11)-C(32)	116.0(3)
N(6)-Si(11)-C(31)	112.3(3)
C(32)-Si(11)-C(31)	104.0(4)
N(6)-Si(11)-C(33)	109.1(3)
C(32)-Si(11)-C(33)	104.5(4)
C(31)-Si(11)-C(33)	110.7(4)
C(32)-Si(11)-Li(10)	157.4(3)
C(31)-Si(11)-Li(10)	86.2(3)
C(33)-Si(11)-Li(10)	90.2(3)
N(6)-Si(12)-C(34)	109.6(3)
N(6)-Si(12)-C(35)	115.0(3)
C(34)-Si(12)-C(35)	105.6(4)
N(6)-Si(12)-C(36)	112.5(3)
C(34)-Si(12)-C(36)	107.0(4)
C(35)-Si(12)-C(36)	106.6(4)
C(34)-Si(12)-Li(6)	88.1(3)
C(35)-Si(12)-Li(6)	159.4(3)
C(36)-Si(12)-Li(6)	83.1(3)
N(7)-Si(13)-C(39)	111.4(3)
N(7)-Si(13)-C(38)	113.2(3)
C(39)-Si(13)-C(38)	107.6(4)
N(7)-Si(13)-C(37)	115.5(3)
C(39)-Si(13)-C(37)	104.5(4)
C(38)-Si(13)-C(37)	104.0(4)
C(39)-Si(13)-Li(6)	92.5(3)
C(38)-Si(13)-Li(6)	86.1(3)
C(37)-Si(13)-Li(6)	156.2(3)
C(39)-Si(13)-Li(7)	78.6(3)
C(38)-Si(13)-Li(7)	143.6(3)
C(37)-Si(13)-Li(7)	109.0(3)
Li(6)-Si(13)-Li(7)	57.6(3)
N(7)-Si(14)-C(41)	115.3(3)
N(7)-Si(14)-C(40)	110.5(3)
C(41)-Si(14)-C(40)	104.8(3)
N(7)-Si(14)-C(42)	112.4(3)
C(41)-Si(14)-C(42)	105.6(3)
C(40)-Si(14)-C(42)	107.7(3)
C(41)-Si(14)-Li(7)	153.7(3)

C(40)-Si(14)-Li(7)	95.3(3)
C(42)-Si(14)-Li(7)	83.6(3)
N(8)-Si(15)-C(43)	112.9(3)
N(8)-Si(15)-C(44)	110.4(3)
C(43)-Si(15)-C(44)	106.7(4)
N(8)-Si(15)-C(45)	116.0(3)
C(43)-Si(15)-C(45)	105.1(3)
C(44)-Si(15)-C(45)	105.2(4)
C(43)-Si(15)-Li(7)	94.1(3)
C(44)-Si(15)-Li(7)	83.5(3)
C(45)-Si(15)-Li(7)	155.3(3)
N(8)-Si(16)-C(47)	117.8(3)
N(8)-Si(16)-C(48)	113.1(3)
C(47)-Si(16)-C(48)	102.1(4)
N(8)-Si(16)-C(46)	108.4(3)
C(47)-Si(16)-C(46)	107.2(3)
C(48)-Si(16)-C(46)	107.7(3)
C(47)-Si(16)-Li(8)	159.8(3)
C(48)-Si(16)-Li(8)	85.5(3)
C(46)-Si(16)-Li(8)	87.9(3)
N(9)-Si(17)-C(51)	112.0(4)
N(9)-Si(17)-C(49)	118.7(3)
C(51)-Si(17)-C(49)	103.9(4)
N(9)-Si(17)-C(50)	108.1(3)
C(51)-Si(17)-C(50)	108.5(4)
C(49)-Si(17)-C(50)	105.1(4)
C(51)-Si(17)-Li(8)	87.0(4)
C(49)-Si(17)-Li(8)	158.9(4)
C(50)-Si(17)-Li(8)	87.9(4)
C(51)-Si(17)-Li(9)	142.3(4)
C(49)-Si(17)-Li(9)	110.0(3)
C(50)-Si(17)-Li(9)	78.2(3)
Li(8)-Si(17)-Li(9)	55.7(3)
N(9)-Si(18)-C(53)	112.4(3)
N(9)-Si(18)-C(52)	114.4(3)
C(53)-Si(18)-C(52)	107.1(4)
N(9)-Si(18)-C(54)	111.7(3)
C(53)-Si(18)-C(54)	106.3(4)
C(52)-Si(18)-C(54)	104.4(4)
C(53)-Si(18)-Li(9)	93.0(3)

C(52)-Si(18)-Li(9)	154.6(4)
C(54)-Si(18)-Li(9)	84.0(3)
C(53)-Si(18)-Li(8)	81.0(3)
C(52)-Si(18)-Li(8)	111.2(3)
C(54)-Si(18)-Li(8)	139.8(3)
Li(9)-Si(18)-Li(8)	55.9(3)
N(10)-Si(19)-C(56)	111.1(3)
N(10)-Si(19)-C(55)	116.8(3)
C(56)-Si(19)-C(55)	107.1(3)
N(10)-Si(19)-C(57)	110.5(3)
C(56)-Si(19)-C(57)	104.5(3)
C(55)-Si(19)-C(57)	106.0(3)
C(56)-Si(19)-Li(10)	90.8(3)
C(55)-Si(19)-Li(10)	156.8(3)
C(57)-Si(19)-Li(10)	82.9(3)
N(10)-Si(20)-C(58)	113.8(3)
N(10)-Si(20)-C(60)	112.7(3)
C(58)-Si(20)-C(60)	107.4(3)
N(10)-Si(20)-C(59)	111.3(3)
C(58)-Si(20)-C(59)	103.0(3)
C(60)-Si(20)-C(59)	107.9(3)
C(58)-Si(20)-Li(9)	155.3(3)
C(60)-Si(20)-Li(9)	84.0(3)
C(59)-Si(20)-Li(9)	93.7(3)
Si(2)-N(1)-Si(1)	120.7(3)
Si(2)-N(1)-Li(1)	107.4(4)
Si(1)-N(1)-Li(1)	113.7(4)
Si(2)-N(1)-Li(5)	113.7(4)
Si(1)-N(1)-Li(5)	105.1(4)
Li(1)-N(1)-Li(5)	92.7(5)
Si(4)-N(2)-Si(3)	119.0(3)
Si(4)-N(2)-Li(2)	118.5(4)
Si(3)-N(2)-Li(2)	104.1(3)
Si(4)-N(2)-Li(1)	109.7(4)
Si(3)-N(2)-Li(1)	111.6(4)
Li(2)-N(2)-Li(1)	90.5(4)
Si(5)-N(3)-Si(6)	120.0(3)
Si(5)-N(3)-Li(2)	115.9(4)
Si(6)-N(3)-Li(2)	104.1(4)
Si(5)-N(3)-Li(3)	101.6(4)

Si(6)-N(3)-Li(3)	120.9(4)
Li(2)-N(3)-Li(3)	91.2(5)
Si(8)-N(4)-Si(7)	120.6(3)
Si(8)-N(4)-Li(3)	117.9(4)
Si(7)-N(4)-Li(3)	101.3(4)
Si(8)-N(4)-Li(4)	105.0(4)
Si(7)-N(4)-Li(4)	116.0(4)
Li(3)-N(4)-Li(4)	93.0(5)
Si(10)-N(5)-Si(9)	119.4(3)
Si(10)-N(5)-Li(4)	114.4(4)
Si(9)-N(5)-Li(4)	106.2(3)
Si(10)-N(5)-Li(5)	107.9(4)
Si(9)-N(5)-Li(5)	115.4(4)
Li(4)-N(5)-Li(5)	89.8(4)
Si(12)-N(6)-Si(11)	120.9(3)
Si(12)-N(6)-Li(10)	117.4(4)
Si(11)-N(6)-Li(10)	104.4(4)
Si(12)-N(6)-Li(6)	101.0(4)
Si(11)-N(6)-Li(6)	118.8(4)
Li(10)-N(6)-Li(6)	91.1(5)
Si(13)-N(7)-Si(14)	120.4(3)
Si(13)-N(7)-Li(7)	111.7(4)
Si(14)-N(7)-Li(7)	106.8(4)
Si(13)-N(7)-Li(6)	105.1(4)
Si(14)-N(7)-Li(6)	117.1(4)
Li(7)-N(7)-Li(6)	92.2(4)
Si(15)-N(8)-Si(16)	119.0(3)
Si(15)-N(8)-Li(8)	116.9(4)
Si(16)-N(8)-Li(8)	103.2(4)
Si(15)-N(8)-Li(7)	107.8(4)
Si(16)-N(8)-Li(7)	114.6(4)
Li(8)-N(8)-Li(7)	92.4(4)
Si(17)-N(9)-Si(18)	120.5(3)
Si(17)-N(9)-Li(9)	115.1(4)
Si(18)-N(9)-Li(9)	104.7(4)
Si(17)-N(9)-Li(8)	107.1(4)
Si(18)-N(9)-Li(8)	115.4(4)
Li(9)-N(9)-Li(8)	90.2(4)
Si(19)-N(10)-Si(20)	120.1(3)
Si(19)-N(10)-Li(10)	107.2(4)

Si(20)-N(10)-Li(10)	114.7(4)
Si(19)-N(10)-Li(9)	114.8(4)
Si(20)-N(10)-Li(9)	104.9(3)
Li(10)-N(10)-Li(9)	91.7(4)
C(68)-N(11)-C(61)	112.8(5)
C(68)-N(11)-C(67)	107.3(5)
C(61)-N(11)-C(67)	110.7(5)
C(68)-N(11)-Li(11)	107.1(5)
C(61)-N(11)-Li(11)	104.9(4)
C(67)-N(11)-Li(11)	114.0(5)
C(69)-N(12)-C(66)	114.4(5)
C(69)-N(12)-C(70)	107.7(5)
C(66)-N(12)-C(70)	107.0(5)
C(69)-N(12)-Li(11)	104.7(4)
C(66)-N(12)-Li(11)	108.6(4)
C(70)-N(12)-Li(11)	114.6(5)
C(78)-N(13)-C(77)	109.3(6)
C(78)-N(13)-C(71)	110.1(5)
C(77)-N(13)-C(71)	111.6(5)
C(78)-N(13)-Li(11)	117.7(5)
C(77)-N(13)-Li(11)	101.3(5)
C(71)-N(13)-Li(11)	106.6(5)
C(79)-N(14)-C(76)	111.9(5)
C(79)-N(14)-C(80)	108.5(5)
C(76)-N(14)-C(80)	109.9(5)
C(79)-N(14)-Li(11)	106.5(5)
C(76)-N(14)-Li(11)	106.6(5)
C(80)-N(14)-Li(11)	113.5(5)
C(81)-N(15)-C(87)	112.1(6)
C(81)-N(15)-C(88)	113.9(6)
C(87)-N(15)-C(88)	105.5(6)
C(81)-N(15)-Li(12)	107.7(5)
C(87)-N(15)-Li(12)	104.8(5)
C(88)-N(15)-Li(12)	112.5(5)
C(90)-N(16)-C(86)	111.1(6)
C(90)-N(16)-C(89)	108.2(6)
C(86)-N(16)-C(89)	112.2(6)
C(90)-N(16)-Li(12)	113.0(6)
C(86)-N(16)-Li(12)	103.6(5)
C(89)-N(16)-Li(12)	108.7(6)

C(98)-N(17)-C(97)	107.0(6)
C(98)-N(17)-C(91)	112.5(5)
C(97)-N(17)-C(91)	112.6(5)
C(98)-N(17)-Li(12)	113.2(5)
C(97)-N(17)-Li(12)	110.9(5)
C(91)-N(17)-Li(12)	100.7(5)
C(100)-N(18)-C(99)	107.1(6)
C(100)-N(18)-C(96)	112.1(5)
C(99)-N(18)-C(96)	110.7(6)
C(100)-N(18)-Li(12)	104.9(6)
C(99)-N(18)-Li(12)	115.0(5)
C(96)-N(18)-Li(12)	106.9(5)
Si(1)-C(1)-H(1A)	109.5
Si(1)-C(1)-H(1B)	109.5
H(1A)-C(1)-H(1B)	109.5
Si(1)-C(1)-H(1C)	109.5
H(1A)-C(1)-H(1C)	109.5
H(1B)-C(1)-H(1C)	109.5
Si(1)-C(2)-H(2A)	109.5
Si(1)-C(2)-H(2B)	109.5
H(2A)-C(2)-H(2B)	109.5
Si(1)-C(2)-H(2C)	109.5
H(2A)-C(2)-H(2C)	109.5
H(2B)-C(2)-H(2C)	109.5
Si(1)-C(3)-H(3A)	109.5
Si(1)-C(3)-H(3B)	109.5
H(3A)-C(3)-H(3B)	109.5
Si(1)-C(3)-H(3C)	109.5
H(3A)-C(3)-H(3C)	109.5
H(3B)-C(3)-H(3C)	109.5
Si(2)-C(4)-H(4A)	109.5
Si(2)-C(4)-H(4B)	109.5
H(4A)-C(4)-H(4B)	109.5
Si(2)-C(4)-H(4C)	109.5
H(4A)-C(4)-H(4C)	109.5
H(4B)-C(4)-H(4C)	109.5
Si(2)-C(5)-H(5A)	109.5
Si(2)-C(5)-H(5B)	109.5
H(5A)-C(5)-H(5B)	109.5
Si(2)-C(5)-H(5C)	109.5

H(5A)-C(5)-H(5C)	109.5
H(5B)-C(5)-H(5C)	109.5
Si(2)-C(6)-H(6A)	109.5
Si(2)-C(6)-H(6B)	109.5
H(6A)-C(6)-H(6B)	109.5
Si(2)-C(6)-H(6C)	109.5
H(6A)-C(6)-H(6C)	109.5
H(6B)-C(6)-H(6C)	109.5
Si(3)-C(7)-H(7A)	109.5
Si(3)-C(7)-H(7B)	109.5
H(7A)-C(7)-H(7B)	109.5
Si(3)-C(7)-H(7C)	109.5
H(7A)-C(7)-H(7C)	109.5
H(7B)-C(7)-H(7C)	109.5
Si(3)-C(8)-H(8A)	109.5
Si(3)-C(8)-H(8B)	109.5
H(8A)-C(8)-H(8B)	109.5
Si(3)-C(8)-H(8C)	109.5
H(8A)-C(8)-H(8C)	109.5
H(8B)-C(8)-H(8C)	109.5
Si(3)-C(9)-H(9A)	109.5
Si(3)-C(9)-H(9B)	109.5
H(9A)-C(9)-H(9B)	109.5
Si(3)-C(9)-H(9C)	109.5
H(9A)-C(9)-H(9C)	109.5
H(9B)-C(9)-H(9C)	109.5
Si(4)-C(10)-H(10A)	109.5
Si(4)-C(10)-H(10B)	109.5
H(10A)-C(10)-H(10B)	109.5
Si(4)-C(10)-H(10C)	109.5
H(10A)-C(10)-H(10C)	109.5
H(10B)-C(10)-H(10C)	109.5
Si(4)-C(11)-H(11A)	109.5
Si(4)-C(11)-H(11B)	109.5
H(11A)-C(11)-H(11B)	109.5
Si(4)-C(11)-H(11C)	109.5
H(11A)-C(11)-H(11C)	109.5
H(11B)-C(11)-H(11C)	109.5
Si(4)-C(12)-H(12A)	109.5
Si(4)-C(12)-H(12B)	109.5

H(12A)-C(12)-H(12B)	109.5
Si(4)-C(12)-H(12C)	109.5
H(12A)-C(12)-H(12C)	109.5
H(12B)-C(12)-H(12C)	109.5
Si(5)-C(13)-H(13A)	109.5
Si(5)-C(13)-H(13B)	109.5
H(13A)-C(13)-H(13B)	109.5
Si(5)-C(13)-H(13C)	109.5
H(13A)-C(13)-H(13C)	109.5
H(13B)-C(13)-H(13C)	109.5
Si(5)-C(14)-H(14A)	109.5
Si(5)-C(14)-H(14B)	109.5
H(14A)-C(14)-H(14B)	109.5
Si(5)-C(14)-H(14C)	109.5
H(14A)-C(14)-H(14C)	109.5
H(14B)-C(14)-H(14C)	109.5
Si(5)-C(15)-H(15A)	109.5
Si(5)-C(15)-H(15B)	109.5
H(15A)-C(15)-H(15B)	109.5
Si(5)-C(15)-H(15C)	109.5
H(15A)-C(15)-H(15C)	109.5
H(15B)-C(15)-H(15C)	109.5
Si(6)-C(16)-H(16A)	109.5
Si(6)-C(16)-H(16B)	109.5
H(16A)-C(16)-H(16B)	109.5
Si(6)-C(16)-H(16C)	109.5
H(16A)-C(16)-H(16C)	109.5
H(16B)-C(16)-H(16C)	109.5
Si(6)-C(17)-H(17A)	109.5
Si(6)-C(17)-H(17B)	109.5
H(17A)-C(17)-H(17B)	109.5
Si(6)-C(17)-H(17C)	109.5
H(17A)-C(17)-H(17C)	109.5
H(17B)-C(17)-H(17C)	109.5
Si(6)-C(18)-H(18A)	109.5
Si(6)-C(18)-H(18B)	109.5
H(18A)-C(18)-H(18B)	109.5
Si(6)-C(18)-H(18C)	109.5
H(18A)-C(18)-H(18C)	109.5
H(18B)-C(18)-H(18C)	109.5

Si(7)-C(19)-H(19A)	109.5
Si(7)-C(19)-H(19B)	109.5
H(19A)-C(19)-H(19B)	109.5
Si(7)-C(19)-H(19C)	109.5
H(19A)-C(19)-H(19C)	109.5
H(19B)-C(19)-H(19C)	109.5
Si(7)-C(20)-H(20A)	109.5
Si(7)-C(20)-H(20B)	109.5
H(20A)-C(20)-H(20B)	109.5
Si(7)-C(20)-H(20C)	109.5
H(20A)-C(20)-H(20C)	109.5
H(20B)-C(20)-H(20C)	109.5
Si(7)-C(21)-H(21A)	109.5
Si(7)-C(21)-H(21B)	109.5
H(21A)-C(21)-H(21B)	109.5
Si(7)-C(21)-H(21C)	109.5
H(21A)-C(21)-H(21C)	109.5
H(21B)-C(21)-H(21C)	109.5
Si(8)-C(22)-H(22A)	109.5
Si(8)-C(22)-H(22B)	109.5
H(22A)-C(22)-H(22B)	109.5
Si(8)-C(22)-H(22C)	109.5
H(22A)-C(22)-H(22C)	109.5
H(22B)-C(22)-H(22C)	109.5
Si(8)-C(23)-H(23A)	109.5
Si(8)-C(23)-H(23B)	109.5
H(23A)-C(23)-H(23B)	109.5
Si(8)-C(23)-H(23C)	109.5
H(23A)-C(23)-H(23C)	109.5
H(23B)-C(23)-H(23C)	109.5
Si(8)-C(24)-H(24A)	109.5
Si(8)-C(24)-H(24B)	109.5
H(24A)-C(24)-H(24B)	109.5
Si(8)-C(24)-H(24C)	109.5
H(24A)-C(24)-H(24C)	109.5
H(24B)-C(24)-H(24C)	109.5
Si(9)-C(25)-H(25A)	109.5
Si(9)-C(25)-H(25B)	109.5
H(25A)-C(25)-H(25B)	109.5
Si(9)-C(25)-H(25C)	109.5

H(25A)-C(25)-H(25C)	109.5
H(25B)-C(25)-H(25C)	109.5
Si(9)-C(26)-H(26A)	109.5
Si(9)-C(26)-H(26B)	109.5
H(26A)-C(26)-H(26B)	109.5
Si(9)-C(26)-H(26C)	109.5
H(26A)-C(26)-H(26C)	109.5
H(26B)-C(26)-H(26C)	109.5
Si(9)-C(27)-H(27A)	109.5
Si(9)-C(27)-H(27B)	109.5
H(27A)-C(27)-H(27B)	109.5
Si(9)-C(27)-H(27C)	109.5
H(27A)-C(27)-H(27C)	109.5
H(27B)-C(27)-H(27C)	109.5
Si(10)-C(28)-H(28A)	109.5
Si(10)-C(28)-H(28B)	109.5
H(28A)-C(28)-H(28B)	109.5
Si(10)-C(28)-H(28C)	109.5
H(28A)-C(28)-H(28C)	109.5
H(28B)-C(28)-H(28C)	109.5
Si(10)-C(29)-H(29A)	109.5
Si(10)-C(29)-H(29B)	109.5
H(29A)-C(29)-H(29B)	109.5
Si(10)-C(29)-H(29C)	109.5
H(29A)-C(29)-H(29C)	109.5
H(29B)-C(29)-H(29C)	109.5
Si(10)-C(30)-H(30A)	109.5
Si(10)-C(30)-H(30B)	109.5
H(30A)-C(30)-H(30B)	109.5
Si(10)-C(30)-H(30C)	109.5
H(30A)-C(30)-H(30C)	109.5
H(30B)-C(30)-H(30C)	109.5
Si(11)-C(31)-H(31A)	109.5
Si(11)-C(31)-H(31B)	109.5
H(31A)-C(31)-H(31B)	109.5
Si(11)-C(31)-H(31C)	109.5
H(31A)-C(31)-H(31C)	109.5
H(31B)-C(31)-H(31C)	109.5
Si(11)-C(32)-H(32A)	109.5
Si(11)-C(32)-H(32B)	109.5

H(32A)-C(32)-H(32B)	109.5
Si(11)-C(32)-H(32C)	109.5
H(32A)-C(32)-H(32C)	109.5
H(32B)-C(32)-H(32C)	109.5
Si(11)-C(33)-H(33A)	109.5
Si(11)-C(33)-H(33B)	109.5
H(33A)-C(33)-H(33B)	109.5
Si(11)-C(33)-H(33C)	109.5
H(33A)-C(33)-H(33C)	109.5
H(33B)-C(33)-H(33C)	109.5
Si(12)-C(34)-H(34A)	109.5
Si(12)-C(34)-H(34B)	109.5
H(34A)-C(34)-H(34B)	109.5
Si(12)-C(34)-H(34C)	109.5
H(34A)-C(34)-H(34C)	109.5
H(34B)-C(34)-H(34C)	109.5
Si(12)-C(35)-H(35A)	109.5
Si(12)-C(35)-H(35B)	109.5
H(35A)-C(35)-H(35B)	109.5
Si(12)-C(35)-H(35C)	109.5
H(35A)-C(35)-H(35C)	109.5
H(35B)-C(35)-H(35C)	109.5
Si(12)-C(36)-H(36A)	109.5
Si(12)-C(36)-H(36B)	109.5
H(36A)-C(36)-H(36B)	109.5
Si(12)-C(36)-H(36C)	109.5
H(36A)-C(36)-H(36C)	109.5
H(36B)-C(36)-H(36C)	109.5
Si(13)-C(37)-H(37A)	109.5
Si(13)-C(37)-H(37B)	109.5
H(37A)-C(37)-H(37B)	109.5
Si(13)-C(37)-H(37C)	109.5
H(37A)-C(37)-H(37C)	109.5
H(37B)-C(37)-H(37C)	109.5
Si(13)-C(38)-H(38A)	109.5
Si(13)-C(38)-H(38B)	109.5
H(38A)-C(38)-H(38B)	109.5
Si(13)-C(38)-H(38C)	109.5
H(38A)-C(38)-H(38C)	109.5
H(38B)-C(38)-H(38C)	109.5

Si(13)-C(39)-H(39A)	109.5
Si(13)-C(39)-H(39B)	109.5
H(39A)-C(39)-H(39B)	109.5
Si(13)-C(39)-H(39C)	109.5
H(39A)-C(39)-H(39C)	109.5
H(39B)-C(39)-H(39C)	109.5
Si(14)-C(40)-H(40A)	109.5
Si(14)-C(40)-H(40B)	109.5
H(40A)-C(40)-H(40B)	109.5
Si(14)-C(40)-H(40C)	109.5
H(40A)-C(40)-H(40C)	109.5
H(40B)-C(40)-H(40C)	109.5
Si(14)-C(41)-H(41A)	109.5
Si(14)-C(41)-H(41B)	109.5
H(41A)-C(41)-H(41B)	109.5
Si(14)-C(41)-H(41C)	109.5
H(41A)-C(41)-H(41C)	109.5
H(41B)-C(41)-H(41C)	109.5
Si(14)-C(42)-H(42A)	109.5
Si(14)-C(42)-H(42B)	109.5
H(42A)-C(42)-H(42B)	109.5
Si(14)-C(42)-H(42C)	109.5
H(42A)-C(42)-H(42C)	109.5
H(42B)-C(42)-H(42C)	109.5
Si(15)-C(43)-H(43A)	109.5
Si(15)-C(43)-H(43B)	109.5
H(43A)-C(43)-H(43B)	109.5
Si(15)-C(43)-H(43C)	109.5
H(43A)-C(43)-H(43C)	109.5
H(43B)-C(43)-H(43C)	109.5
Si(15)-C(44)-H(44A)	109.5
Si(15)-C(44)-H(44B)	109.5
H(44A)-C(44)-H(44B)	109.5
Si(15)-C(44)-H(44C)	109.5
H(44A)-C(44)-H(44C)	109.5
H(44B)-C(44)-H(44C)	109.5
Si(15)-C(45)-H(45A)	109.5
Si(15)-C(45)-H(45B)	109.5
H(45A)-C(45)-H(45B)	109.5
Si(15)-C(45)-H(45C)	109.5

H(45A)-C(45)-H(45C)	109.5
H(45B)-C(45)-H(45C)	109.5
Si(16)-C(46)-H(46A)	109.5
Si(16)-C(46)-H(46B)	109.5
H(46A)-C(46)-H(46B)	109.5
Si(16)-C(46)-H(46C)	109.5
H(46A)-C(46)-H(46C)	109.5
H(46B)-C(46)-H(46C)	109.5
Si(16)-C(47)-H(47A)	109.5
Si(16)-C(47)-H(47B)	109.5
H(47A)-C(47)-H(47B)	109.5
Si(16)-C(47)-H(47C)	109.5
H(47A)-C(47)-H(47C)	109.5
H(47B)-C(47)-H(47C)	109.5
Si(16)-C(48)-H(48A)	109.5
Si(16)-C(48)-H(48B)	109.5
H(48A)-C(48)-H(48B)	109.5
Si(16)-C(48)-H(48C)	109.5
H(48A)-C(48)-H(48C)	109.5
H(48B)-C(48)-H(48C)	109.5
Si(17)-C(49)-H(49A)	109.5
Si(17)-C(49)-H(49B)	109.5
H(49A)-C(49)-H(49B)	109.5
Si(17)-C(49)-H(49C)	109.5
H(49A)-C(49)-H(49C)	109.5
H(49B)-C(49)-H(49C)	109.5
Si(17)-C(50)-H(50A)	109.5
Si(17)-C(50)-H(50B)	109.5
H(50A)-C(50)-H(50B)	109.5
Si(17)-C(50)-H(50C)	109.5
H(50A)-C(50)-H(50C)	109.5
H(50B)-C(50)-H(50C)	109.5
Si(17)-C(51)-H(51A)	109.5
Si(17)-C(51)-H(51B)	109.5
H(51A)-C(51)-H(51B)	109.5
Si(17)-C(51)-H(51C)	109.5
H(51A)-C(51)-H(51C)	109.5
H(51B)-C(51)-H(51C)	109.5
Si(18)-C(52)-H(52A)	109.5
Si(18)-C(52)-H(52B)	109.5

H(52A)-C(52)-H(52B)	109.5
Si(18)-C(52)-H(52C)	109.5
H(52A)-C(52)-H(52C)	109.5
H(52B)-C(52)-H(52C)	109.5
Si(18)-C(53)-H(53A)	109.5
Si(18)-C(53)-H(53B)	109.5
H(53A)-C(53)-H(53B)	109.5
Si(18)-C(53)-H(53C)	109.5
H(53A)-C(53)-H(53C)	109.5
H(53B)-C(53)-H(53C)	109.5
Si(18)-C(54)-H(54A)	109.5
Si(18)-C(54)-H(54B)	109.5
H(54A)-C(54)-H(54B)	109.5
Si(18)-C(54)-H(54C)	109.5
H(54A)-C(54)-H(54C)	109.5
H(54B)-C(54)-H(54C)	109.5
Si(19)-C(55)-H(55A)	109.5
Si(19)-C(55)-H(55B)	109.5
H(55A)-C(55)-H(55B)	109.5
Si(19)-C(55)-H(55C)	109.5
H(55A)-C(55)-H(55C)	109.5
H(55B)-C(55)-H(55C)	109.5
Si(19)-C(56)-H(56A)	109.5
Si(19)-C(56)-H(56B)	109.5
H(56A)-C(56)-H(56B)	109.5
Si(19)-C(56)-H(56C)	109.5
H(56A)-C(56)-H(56C)	109.5
H(56B)-C(56)-H(56C)	109.5
Si(19)-C(57)-H(57A)	109.5
Si(19)-C(57)-H(57B)	109.5
H(57A)-C(57)-H(57B)	109.5
Si(19)-C(57)-H(57C)	109.5
H(57A)-C(57)-H(57C)	109.5
H(57B)-C(57)-H(57C)	109.5
Si(20)-C(58)-H(58A)	109.5
Si(20)-C(58)-H(58B)	109.5
H(58A)-C(58)-H(58B)	109.5
Si(20)-C(58)-H(58C)	109.5
H(58A)-C(58)-H(58C)	109.5
H(58B)-C(58)-H(58C)	109.5

Si(20)-C(59)-H(59A)	109.5
Si(20)-C(59)-H(59B)	109.5
H(59A)-C(59)-H(59B)	109.5
Si(20)-C(59)-H(59C)	109.5
H(59A)-C(59)-H(59C)	109.5
H(59B)-C(59)-H(59C)	109.5
Si(20)-C(60)-H(60A)	109.5
Si(20)-C(60)-H(60B)	109.5
H(60A)-C(60)-H(60B)	109.5
Si(20)-C(60)-H(60C)	109.5
H(60A)-C(60)-H(60C)	109.5
H(60B)-C(60)-H(60C)	109.5
N(11)-C(61)-C(66)	111.5(5)
N(11)-C(61)-C(62)	115.2(5)
C(66)-C(61)-C(62)	108.8(5)
N(11)-C(61)-H(61)	107.0
C(66)-C(61)-H(61)	107.0
C(62)-C(61)-H(61)	107.0
C(63)-C(62)-C(61)	115.0(6)
C(63)-C(62)-H(62A)	108.5
C(61)-C(62)-H(62A)	108.5
C(63)-C(62)-H(62B)	108.5
C(61)-C(62)-H(62B)	108.5
H(62A)-C(62)-H(62B)	107.5
C(64)-C(63)-C(62)	111.7(6)
C(64)-C(63)-H(63A)	109.3
C(62)-C(63)-H(63A)	109.3
C(64)-C(63)-H(63B)	109.3
C(62)-C(63)-H(63B)	109.3
H(63A)-C(63)-H(63B)	107.9
C(63)-C(64)-C(65)	111.5(6)
C(63)-C(64)-H(64A)	109.3
C(65)-C(64)-H(64A)	109.3
C(63)-C(64)-H(64B)	109.3
C(65)-C(64)-H(64B)	109.3
H(64A)-C(64)-H(64B)	108.0
C(66)-C(65)-C(64)	113.1(5)
C(66)-C(65)-H(65A)	109.0
C(64)-C(65)-H(65A)	109.0
C(66)-C(65)-H(65B)	109.0

C(64)-C(65)-H(65B)	109.0
H(65A)-C(65)-H(65B)	107.8
N(12)-C(66)-C(65)	113.8(5)
N(12)-C(66)-C(61)	109.5(5)
C(65)-C(66)-C(61)	111.0(5)
N(12)-C(66)-H(66)	107.4
C(65)-C(66)-H(66)	107.4
C(61)-C(66)-H(66)	107.4
N(11)-C(67)-H(67A)	109.5
N(11)-C(67)-H(67B)	109.5
H(67A)-C(67)-H(67B)	109.5
N(11)-C(67)-H(67C)	109.5
H(67A)-C(67)-H(67C)	109.5
H(67B)-C(67)-H(67C)	109.5
N(11)-C(68)-H(68A)	109.5
N(11)-C(68)-H(68B)	109.5
H(68A)-C(68)-H(68B)	109.5
N(11)-C(68)-H(68C)	109.5
H(68A)-C(68)-H(68C)	109.5
H(68B)-C(68)-H(68C)	109.5
N(12)-C(69)-H(69A)	109.5
N(12)-C(69)-H(69B)	109.5
H(69A)-C(69)-H(69B)	109.5
N(12)-C(69)-H(69C)	109.5
H(69A)-C(69)-H(69C)	109.5
H(69B)-C(69)-H(69C)	109.5
N(12)-C(70)-H(70A)	109.5
N(12)-C(70)-H(70B)	109.5
H(70A)-C(70)-H(70B)	109.5
N(12)-C(70)-H(70C)	109.5
H(70A)-C(70)-H(70C)	109.5
H(70B)-C(70)-H(70C)	109.5
N(13)-C(71)-C(72)	115.5(5)
N(13)-C(71)-C(76)	112.0(5)
C(72)-C(71)-C(76)	110.8(5)
N(13)-C(71)-H(71)	105.9
C(72)-C(71)-H(71)	105.9
C(76)-C(71)-H(71)	105.9
C(71)-C(72)-C(73)	113.8(6)
C(71)-C(72)-H(72A)	108.8

C(73)-C(72)-H(72A)	108.8
C(71)-C(72)-H(72B)	108.8
C(73)-C(72)-H(72B)	108.8
H(72A)-C(72)-H(72B)	107.7
C(74)-C(73)-C(72)	107.9(7)
C(74)-C(73)-H(73A)	110.1
C(72)-C(73)-H(73A)	110.1
C(74)-C(73)-H(73B)	110.1
C(72)-C(73)-H(73B)	110.1
H(73A)-C(73)-H(73B)	108.4
C(73)-C(74)-C(75)	112.8(6)
C(73)-C(74)-H(74A)	109.0
C(75)-C(74)-H(74A)	109.0
C(73)-C(74)-H(74B)	109.0
C(75)-C(74)-H(74B)	109.0
H(74A)-C(74)-H(74B)	107.8
C(74)-C(75)-C(76)	112.6(6)
C(74)-C(75)-H(75A)	109.1
C(76)-C(75)-H(75A)	109.1
C(74)-C(75)-H(75B)	109.1
C(76)-C(75)-H(75B)	109.1
H(75A)-C(75)-H(75B)	107.8
N(14)-C(76)-C(71)	111.6(5)
N(14)-C(76)-C(75)	115.7(5)
C(71)-C(76)-C(75)	112.9(6)
N(14)-C(76)-H(76)	105.2
C(71)-C(76)-H(76)	105.2
C(75)-C(76)-H(76)	105.2
N(13)-C(77)-H(77A)	109.5
N(13)-C(77)-H(77B)	109.5
H(77A)-C(77)-H(77B)	109.5
N(13)-C(77)-H(77C)	109.5
H(77A)-C(77)-H(77C)	109.5
H(77B)-C(77)-H(77C)	109.5
N(13)-C(78)-H(78A)	109.5
N(13)-C(78)-H(78B)	109.5
H(78A)-C(78)-H(78B)	109.5
N(13)-C(78)-H(78C)	109.5
H(78A)-C(78)-H(78C)	109.5
H(78B)-C(78)-H(78C)	109.5

N(14)-C(79)-H(79A)	109.5
N(14)-C(79)-H(79B)	109.5
H(79A)-C(79)-H(79B)	109.5
N(14)-C(79)-H(79C)	109.5
H(79A)-C(79)-H(79C)	109.5
H(79B)-C(79)-H(79C)	109.5
N(14)-C(80)-H(80A)	109.5
N(14)-C(80)-H(80B)	109.5
H(80A)-C(80)-H(80B)	109.5
N(14)-C(80)-H(80C)	109.5
H(80A)-C(80)-H(80C)	109.5
H(80B)-C(80)-H(80C)	109.5
N(15)-C(81)-C(82)	114.6(6)
N(15)-C(81)-C(86)	111.9(5)
C(82)-C(81)-C(86)	112.0(6)
N(15)-C(81)-H(81)	105.8
C(82)-C(81)-H(81)	105.8
C(86)-C(81)-H(81)	105.8
C(83)-C(82)-C(81)	113.4(7)
C(83)-C(82)-H(82A)	108.9
C(81)-C(82)-H(82A)	108.9
C(83)-C(82)-H(82B)	108.9
C(81)-C(82)-H(82B)	108.9
H(82A)-C(82)-H(82B)	107.7
C(84)-C(83)-C(82)	110.7(7)
C(84)-C(83)-H(83A)	109.5
C(82)-C(83)-H(83A)	109.5
C(84)-C(83)-H(83B)	109.5
C(82)-C(83)-H(83B)	109.5
H(83A)-C(83)-H(83B)	108.1
C(83)-C(84)-C(85)	110.1(7)
C(83)-C(84)-H(84A)	109.6
C(85)-C(84)-H(84A)	109.6
C(83)-C(84)-H(84B)	109.6
C(85)-C(84)-H(84B)	109.6
H(84A)-C(84)-H(84B)	108.1
C(84)-C(85)-C(86)	109.6(7)
C(84)-C(85)-H(85A)	109.8
C(86)-C(85)-H(85A)	109.8
C(84)-C(85)-H(85B)	109.8

C(86)-C(85)-H(85B)	109.8
H(85A)-C(85)-H(85B)	108.2
N(16)-C(86)-C(81)	111.3(5)
N(16)-C(86)-C(85)	112.6(6)
C(81)-C(86)-C(85)	111.5(6)
N(16)-C(86)-H(86)	107.0
C(81)-C(86)-H(86)	107.0
C(85)-C(86)-H(86)	107.0
N(15)-C(87)-H(87A)	109.5
N(15)-C(87)-H(87B)	109.5
H(87A)-C(87)-H(87B)	109.5
N(15)-C(87)-H(87C)	109.5
H(87A)-C(87)-H(87C)	109.5
H(87B)-C(87)-H(87C)	109.5
N(15)-C(88)-H(88A)	109.5
N(15)-C(88)-H(88B)	109.5
H(88A)-C(88)-H(88B)	109.5
N(15)-C(88)-H(88C)	109.5
H(88A)-C(88)-H(88C)	109.5
H(88B)-C(88)-H(88C)	109.5
N(16)-C(89)-H(89A)	109.5
N(16)-C(89)-H(89B)	109.5
H(89A)-C(89)-H(89B)	109.5
N(16)-C(89)-H(89C)	109.5
H(89A)-C(89)-H(89C)	109.5
H(89B)-C(89)-H(89C)	109.5
N(16)-C(90)-H(90A)	109.5
N(16)-C(90)-H(90B)	109.5
H(90A)-C(90)-H(90B)	109.5
N(16)-C(90)-H(90C)	109.5
H(90A)-C(90)-H(90C)	109.5
H(90B)-C(90)-H(90C)	109.5
N(17)-C(91)-C(92)	115.4(6)
N(17)-C(91)-C(96)	110.7(5)
C(92)-C(91)-C(96)	111.8(6)
N(17)-C(91)-H(91)	106.1
C(92)-C(91)-H(91)	106.1
C(96)-C(91)-H(91)	106.1
C(91)-C(92)-C(93)	113.1(7)
C(91)-C(92)-H(92A)	109.0

C(93)-C(92)-H(92A)	109.0
C(91)-C(92)-H(92B)	109.0
C(93)-C(92)-H(92B)	109.0
H(92A)-C(92)-H(92B)	107.8
C(92)-C(93)-C(94)	110.7(7)
C(92)-C(93)-H(93A)	109.5
C(94)-C(93)-H(93A)	109.5
C(92)-C(93)-H(93B)	109.5
C(94)-C(93)-H(93B)	109.5
H(93A)-C(93)-H(93B)	108.1
C(95)-C(94)-C(93)	109.2(7)
C(95)-C(94)-H(94A)	109.8
C(93)-C(94)-H(94A)	109.8
C(95)-C(94)-H(94B)	109.8
C(93)-C(94)-H(94B)	109.8
H(94A)-C(94)-H(94B)	108.3
C(96)-C(95)-C(94)	111.6(6)
C(96)-C(95)-H(95A)	109.3
C(94)-C(95)-H(95A)	109.3
C(96)-C(95)-H(95B)	109.3
C(94)-C(95)-H(95B)	109.3
H(95A)-C(95)-H(95B)	108.0
N(18)-C(96)-C(95)	114.8(6)
N(18)-C(96)-C(91)	111.6(5)
C(95)-C(96)-C(91)	113.0(6)
N(18)-C(96)-H(96)	105.5
C(95)-C(96)-H(96)	105.5
C(91)-C(96)-H(96)	105.5
N(17)-C(97)-H(97A)	109.5
N(17)-C(97)-H(97B)	109.5
H(97A)-C(97)-H(97B)	109.5
N(17)-C(97)-H(97C)	109.5
H(97A)-C(97)-H(97C)	109.5
H(97B)-C(97)-H(97C)	109.5
N(17)-C(98)-H(98A)	109.5
N(17)-C(98)-H(98B)	109.5
H(98A)-C(98)-H(98B)	109.5
N(17)-C(98)-H(98C)	109.5
H(98A)-C(98)-H(98C)	109.5
H(98B)-C(98)-H(98C)	109.5

N(18)-C(99)-H(99A)	109.5
N(18)-C(99)-H(99B)	109.5
H(99A)-C(99)-H(99B)	109.5
N(18)-C(99)-H(99C)	109.5
H(99A)-C(99)-H(99C)	109.5
H(99B)-C(99)-H(99C)	109.5
N(18)-C(100)-H(10D)	109.5
N(18)-C(100)-H(10E)	109.5
H(10D)-C(100)-H(10E)	109.5
N(18)-C(100)-H(10F)	109.5
H(10D)-C(100)-H(10F)	109.5
H(10E)-C(100)-H(10F)	109.5
C(301)-C(300)-H(30D)	109.5
C(301)-C(300)-H(30E)	109.5
H(30D)-C(300)-H(30E)	109.5
C(301)-C(300)-H(30F)	109.5
H(30D)-C(300)-H(30F)	109.5
H(30E)-C(300)-H(30F)	109.5
C(306)-C(301)-C(302)	116.8(7)
C(306)-C(301)-C(300)	120.8(7)
C(302)-C(301)-C(300)	122.4(8)
C(303)-C(302)-C(301)	124.1(8)
C(303)-C(302)-H(302)	117.9
C(301)-C(302)-H(302)	117.9
C(302)-C(303)-C(304)	119.0(7)
C(302)-C(303)-H(303)	120.5
C(304)-C(303)-H(303)	120.5
C(303)-C(304)-C(305)	118.3(7)
C(303)-C(304)-H(304)	120.8
C(305)-C(304)-H(304)	120.8
C(306)-C(305)-C(304)	119.4(7)
C(306)-C(305)-H(305)	120.3
C(304)-C(305)-H(305)	120.3
C(301)-C(306)-C(305)	122.3(7)
C(301)-C(306)-H(306)	118.9
C(305)-C(306)-H(306)	118.9
C(312)-C(311)-C(316)	120.0
C(312)-C(311)-C(310)	121.7(15)
C(316)-C(311)-C(310)	118.3(15)
C(313)-C(312)-C(311)	120.0

C(313)-C(312)-H(312)	120.0
C(311)-C(312)-H(312)	120.0
C(312)-C(313)-C(314)	120.0
C(312)-C(313)-H(313)	120.0
C(314)-C(313)-H(313)	120.0
C(315)-C(314)-C(313)	120.0
C(315)-C(314)-H(314)	120.0
C(313)-C(314)-H(314)	120.0
C(314)-C(315)-C(316)	120.0
C(314)-C(315)-H(315)	120.0
C(316)-C(315)-H(315)	120.0
C(315)-C(316)-C(311)	120.0
C(315)-C(316)-H(316)	120.0
C(311)-C(316)-H(316)	120.0
C(321)-C(320)-H(42D)	150.8
H(32D)-C(320)-H(42D)	64.4
H(32E)-C(320)-H(42D)	99.1
H(32F)-C(320)-H(42D)	53.2
C(321)-C(320)-H(42E)	130.1
H(32D)-C(320)-H(42E)	66.3
H(32F)-C(320)-H(42E)	118.8
H(42D)-C(320)-H(42E)	75.6
C(321)-C(320)-H(42F)	128.8
H(32D)-C(320)-H(42F)	119.8
H(32F)-C(320)-H(42F)	67.4
H(42D)-C(320)-H(42F)	70.5
H(42E)-C(320)-H(42F)	65.4
C(326)-C(321)-C(320)	118.0(11)
C(326)-C(321)-C(322)	117.1(9)
C(320)-C(321)-C(322)	124.9(10)
C(323)-C(322)-C(321)	118.0(9)
C(323)-C(322)-H(322)	121.0
C(321)-C(322)-H(322)	121.0
C(324)-C(323)-C(322)	124.7(12)
C(324)-C(323)-H(323)	117.7
C(322)-C(323)-H(323)	117.7
C(323)-C(324)-C(325)	115.3(12)
C(323)-C(324)-H(324)	122.3
C(325)-C(324)-H(324)	122.3
C(324)-C(325)-C(326)	125.4(11)

C(324)-C(325)-H(325)	117.3
C(326)-C(325)-H(325)	117.3
C(321)-C(326)-C(325)	119.3(10)
C(321)-C(326)-H(326)	120.4
C(325)-C(326)-H(326)	120.4
C(332)-C(331)-C(336)	119.5(9)
C(332)-C(331)-C(330)	119.1(8)
C(336)-C(331)-C(330)	121.5(8)
C(331)-C(330)-H(33D)	109.5
C(331)-C(330)-H(33E)	109.5
H(33D)-C(330)-H(33E)	109.5
C(331)-C(330)-H(33F)	109.5
H(33D)-C(330)-H(33F)	109.5
H(33E)-C(330)-H(33F)	109.5
C(331)-C(332)-C(333)	122.7(10)
C(331)-C(332)-H(332)	118.6
C(333)-C(332)-H(332)	118.6
C(334)-C(333)-C(332)	118.7(10)
C(334)-C(333)-H(333)	120.7
C(332)-C(333)-H(333)	120.7
C(335)-C(334)-C(333)	124.8(10)
C(335)-C(334)-H(334)	117.6
C(333)-C(334)-H(334)	117.6
C(334)-C(335)-C(336)	118.3(9)
C(334)-C(335)-H(335)	120.9
C(336)-C(335)-H(335)	120.9
C(331)-C(336)-C(335)	115.8(8)
C(331)-C(336)-H(336)	122.1
C(335)-C(336)-H(336)	122.1
C(342)-C(341)-C(343)	120.0
C(342)-C(341)-H(341)	120.0
C(343)-C(341)-H(341)	120.0
C(341)-C(342)-C(344)	120.0
C(341)-C(342)-H(342)	120.0
C(344)-C(342)-H(342)	120.0
C(345)-C(344)-C(342)	120.0
C(345)-C(344)-H(344)	120.0
C(342)-C(344)-H(344)	120.0
C(344)-C(345)-C(346)	120.0
C(344)-C(345)-H(345)	120.0

C(346)-C(345)-H(345)	120.0
C(345)-C(346)-C(343)	120.0
C(345)-C(346)-H(346)	120.0
C(343)-C(346)-H(346)	120.0
C(346)-C(343)-C(341)	120.0
C(346)-C(343)-C(340)	121.0(12)
C(341)-C(343)-C(340)	118.7(12)
C(401)-C(400)-C(405)	120.0
C(401)-C(400)-C(408)	120.7(14)
C(405)-C(400)-C(408)	118.9(14)
C(400)-C(401)-C(402)	120.0
C(400)-C(401)-H(401)	120.0
C(402)-C(401)-H(401)	120.0
C(403)-C(402)-C(401)	120.0
C(403)-C(402)-H(402)	120.0
C(401)-C(402)-H(402)	120.0
C(402)-C(403)-C(404)	120.0
C(402)-C(403)-H(403)	120.0
C(404)-C(403)-H(403)	120.0
C(403)-C(404)-C(405)	120.0
C(403)-C(404)-H(404)	120.0
C(405)-C(404)-H(404)	120.0
C(404)-C(405)-C(400)	120.0
C(404)-C(405)-H(405)	120.0
C(400)-C(405)-H(405)	120.0
C(411)-C(410)-C(415)	120.0
C(411)-C(410)-C(418)	117.0(9)
C(415)-C(410)-C(418)	122.2(9)
C(412)-C(411)-C(410)	120.0
C(412)-C(411)-H(411)	120.0
C(410)-C(411)-H(411)	120.0
C(411)-C(412)-C(413)	120.0
C(411)-C(412)-H(412)	120.0
C(413)-C(412)-H(412)	120.0
C(414)-C(413)-C(412)	120.0
C(414)-C(413)-H(413)	120.0
C(412)-C(413)-H(413)	120.0
C(413)-C(414)-C(415)	120.0
C(413)-C(414)-H(414)	120.0
C(415)-C(414)-H(414)	120.0

C(414)-C(415)-C(410)	120.0
C(414)-C(415)-H(415)	120.0
C(410)-C(415)-H(415)	120.0
C(420)-C(428)-H(42D)	109.8
C(420)-C(428)-H(42E)	116.4
H(42D)-C(428)-H(42E)	109.5
C(420)-C(428)-H(42F)	101.8
H(42D)-C(428)-H(42F)	109.5
H(42E)-C(428)-H(42F)	109.5
C(421)-C(420)-C(425)	120.0
C(421)-C(420)-C(428)	137(2)
C(425)-C(420)-C(428)	103(2)
C(420)-C(421)-C(422)	120.0
C(420)-C(421)-H(421)	120.0
C(422)-C(421)-H(421)	120.0
C(423)-C(422)-C(421)	120.0
C(423)-C(422)-H(422)	120.0
C(421)-C(422)-H(422)	120.0
C(422)-C(423)-C(424)	120.0
C(422)-C(423)-H(423)	120.0
C(424)-C(423)-H(423)	120.0
C(423)-C(424)-C(425)	120.0
C(423)-C(424)-H(424)	120.0
C(425)-C(424)-H(424)	120.0
C(424)-C(425)-C(420)	120.0
C(424)-C(425)-H(425)	120.0
C(420)-C(425)-H(425)	120.0
C(400)-C(408)-H(40D)	109.5
C(400)-C(408)-H(40E)	109.5
H(40D)-C(408)-H(40E)	109.5
C(400)-C(408)-H(40F)	109.5
H(40D)-C(408)-H(40F)	109.5
H(40E)-C(408)-H(40F)	109.5
C(410)-C(418)-H(41D)	109.5
C(410)-C(418)-H(41E)	109.5
H(41D)-C(418)-H(41E)	109.5
C(410)-C(418)-H(41F)	109.5
H(41D)-C(418)-H(41F)	109.5
H(41E)-C(418)-H(41F)	109.5

Symmetry transformations used to generate equivalent atoms:

Table 4. Anisotropic displacement parameters ($Å^2$x 10^3) for srgr1014. The anisotropic displacement factor exponent takes the form: $-2\pi^2$[h^2 $a^{*2}U^{11}$ + ... + 2 h k a* b* U^{12}]

	U^{11}	U^{22}	U^{33}	U^{23}	U^{13}	U^{12}
Li(1)	37(5)	37(6)	40(6)	-4(5)	-11(5)	0(5)
Li(2)	37(5)	35(5)	23(5)	1(4)	4(4)	6(4)
Li(3)	42(6)	39(6)	30(5)	-14(4)	3(5)	3(5)
Li(4)	35(5)	31(5)	22(5)	-7(4)	0(4)	3(4)
Li(5)	44(6)	35(6)	22(5)	4(4)	-6(4)	7(5)
Li(6)	44(6)	47(6)	24(5)	-8(4)	0(4)	3(5)
Li(7)	41(5)	37(6)	26(5)	-2(4)	-10(4)	9(5)
Li(8)	48(6)	36(6)	26(5)	1(4)	-5(5)	7(5)
Li(9)	23(4)	42(6)	23(5)	0(4)	-9(4)	6(4)
Li(10)	32(5)	55(7)	27(5)	-12(5)	-1(4)	5(5)
Li(11)	36(5)	46(6)	29(5)	-3(5)	-5(4)	-2(5)
Li(12)	40(5)	37(6)	31(5)	-3(4)	-4(4)	10(5)
Br(1)	46(1)	54(1)	22(1)	-11(1)	-12(1)	23(1)
Br(1A)	46(1)	54(1)	22(1)	-11(1)	-12(1)	23(1)
Br(2)	46(1)	45(1)	24(1)	-11(1)	-12(1)	16(1)
Br(2A)	46(1)	45(1)	24(1)	-11(1)	-12(1)	16(1)
Si(1)	32(1)	49(1)	22(1)	-6(1)	-5(1)	-2(1)
Si(2)	39(1)	37(1)	21(1)	5(1)	0(1)	6(1)
Si(3)	33(1)	35(1)	17(1)	-1(1)	-3(1)	0(1)
Si(4)	39(1)	43(1)	34(1)	-13(1)	-7(1)	-6(1)
Si(5)	28(1)	33(1)	28(1)	-5(1)	-4(1)	0(1)
Si(6)	37(1)	29(1)	37(1)	-6(1)	-3(1)	2(1)
Si(7)	39(1)	37(1)	21(1)	0(1)	-3(1)	0(1)
Si(8)	38(1)	36(1)	31(1)	-8(1)	-12(1)	2(1)
Si(9)	37(1)	46(1)	22(1)	-8(1)	-1(1)	5(1)
Si(10)	40(1)	35(1)	35(1)	-8(1)	-10(1)	7(1)
Si(11)	38(1)	44(1)	25(1)	-3(1)	2(1)	-1(1)
Si(12)	43(1)	36(1)	37(1)	-8(1)	-6(1)	4(1)
Si(13)	40(1)	38(1)	23(1)	5(1)	-5(1)	-6(1)
Si(14)	40(1)	40(1)	33(1)	-1(1)	-10(1)	-1(1)
Si(15)	32(1)	30(1)	36(1)	-2(1)	-8(1)	3(1)
Si(16)	34(1)	37(1)	48(1)	-5(1)	-10(1)	4(1)
Si(17)	53(1)	38(1)	50(1)	-17(1)	-24(1)	7(1)
Si(18)	59(1)	52(1)	24(1)	-3(1)	3(1)	4(1)
Si(19)	31(1)	32(1)	45(1)	2(1)	-10(1)	1(1)

Si(20)	26(1)	31(1)	35(1)	1(1)	-8(1)	0(1)
N(1)	37(3)	35(3)	10(2)	2(2)	-5(2)	4(2)
N(2)	28(2)	35(3)	17(2)	-2(2)	0(2)	-9(2)
N(3)	27(2)	28(3)	33(3)	1(2)	-2(2)	2(2)
N(4)	36(3)	37(3)	22(2)	-5(2)	-1(2)	0(2)
N(5)	34(3)	33(3)	19(2)	-3(2)	-2(2)	7(2)
N(6)	36(3)	37(3)	30(3)	-6(2)	-5(2)	4(2)
N(7)	40(3)	34(3)	21(2)	1(2)	-6(2)	-9(2)
N(8)	25(2)	30(3)	30(3)	-5(2)	-5(2)	2(2)
N(9)	45(3)	36(3)	24(2)	-7(2)	-10(2)	1(2)
N(10)	29(2)	28(3)	30(3)	2(2)	-4(2)	0(2)
N(11)	45(3)	31(3)	21(2)	1(2)	-2(2)	-1(2)
N(12)	41(3)	33(3)	19(2)	-3(2)	-7(2)	-1(2)
N(13)	39(3)	36(3)	44(3)	-3(2)	-8(3)	4(2)
N(14)	37(3)	34(3)	30(3)	0(2)	-12(2)	3(2)
N(15)	34(3)	51(3)	38(3)	4(3)	-5(2)	2(3)
N(16)	73(4)	37(3)	37(3)	0(2)	-24(3)	-5(3)
N(17)	34(3)	36(3)	34(3)	-1(2)	-10(2)	5(2)
N(18)	29(3)	39(3)	45(3)	-2(2)	0(2)	2(2)
C(1)	42(4)	59(4)	33(3)	-18(3)	3(3)	-5(3)
C(2)	40(4)	76(5)	32(3)	-15(3)	-18(3)	18(3)
C(3)	44(4)	63(5)	33(4)	-4(3)	-14(3)	0(3)
C(4)	48(4)	51(4)	26(3)	4(3)	2(3)	10(3)
C(5)	68(5)	46(4)	48(4)	1(3)	-3(4)	-15(4)
C(6)	66(5)	45(4)	48(4)	1(3)	-12(4)	25(4)
C(7)	43(4)	45(4)	34(3)	0(3)	-4(3)	-5(3)
C(8)	40(3)	57(4)	27(3)	-7(3)	1(3)	-2(3)
C(9)	48(4)	58(4)	19(3)	-4(3)	-4(3)	10(3)
C(10)	40(4)	59(4)	44(4)	-15(3)	-13(3)	-5(3)
C(11)	59(4)	51(4)	62(5)	-30(4)	-19(4)	4(4)
C(12)	46(4)	32(4)	70(5)	6(3)	-18(4)	-12(3)
C(13)	36(3)	36(3)	41(4)	-2(3)	-6(3)	-8(3)
C(14)	33(3)	58(4)	42(4)	-13(3)	-7(3)	-1(3)
C(15)	41(3)	47(4)	43(4)	-15(3)	-3(3)	-3(3)
C(16)	66(5)	35(4)	50(4)	-4(3)	3(4)	-14(3)
C(17)	46(4)	24(3)	48(4)	4(3)	-7(3)	9(3)
C(18)	49(4)	42(4)	39(4)	-12(3)	-12(3)	4(3)
C(19)	52(4)	47(4)	39(4)	10(3)	-13(3)	8(3)
C(20)	41(4)	43(4)	45(4)	-11(3)	-1(3)	2(3)
C(21)	53(4)	62(4)	19(3)	2(3)	0(3)	4(3)

C(22)	62(4)	43(4)	41(4)	-17(3)	-16(3)	2(3)
C(23)	49(4)	54(4)	44(4)	-10(3)	-25(3)	10(3)
C(24)	40(4)	49(4)	48(4)	-3(3)	-5(3)	-2(3)
C(25)	34(3)	65(5)	60(5)	-26(4)	7(3)	8(3)
C(26)	50(4)	64(5)	29(3)	-11(3)	0(3)	-3(3)
C(27)	54(4)	45(4)	23(3)	-1(3)	-7(3)	-7(3)
C(28)	44(4)	47(4)	62(5)	7(4)	-13(4)	-5(3)
C(29)	50(4)	49(4)	50(4)	-2(3)	-20(3)	19(3)
C(30)	64(5)	55(4)	49(4)	-21(4)	-18(4)	6(4)
C(31)	54(4)	69(5)	49(4)	-17(4)	-13(4)	-2(4)
C(32)	47(4)	96(6)	37(4)	-4(4)	7(3)	7(4)
C(33)	56(4)	49(4)	59(5)	-2(4)	-9(4)	-7(4)
C(34)	51(4)	36(4)	54(4)	7(3)	-9(3)	-5(3)
C(35)	45(4)	52(4)	66(5)	-27(4)	-14(4)	11(3)
C(36)	66(5)	58(5)	48(4)	-10(4)	-24(4)	-5(4)
C(37)	53(4)	54(4)	46(4)	17(3)	-10(3)	-15(4)
C(38)	41(4)	67(5)	40(4)	-8(4)	1(3)	1(3)
C(39)	59(5)	63(5)	45(4)	-5(4)	-14(4)	-19(4)
C(40)	61(4)	31(3)	42(4)	3(3)	-16(3)	-13(3)
C(41)	48(4)	55(4)	39(4)	6(3)	-22(3)	-3(3)
C(42)	49(4)	50(4)	52(4)	-2(3)	-6(3)	8(3)
C(43)	37(4)	50(4)	79(6)	-5(4)	-15(4)	2(3)
C(44)	64(5)	52(4)	51(5)	10(4)	-26(4)	7(4)
C(45)	45(4)	49(4)	71(5)	-28(4)	-4(4)	-7(3)
C(46)	44(4)	42(4)	44(4)	-1(3)	2(3)	-14(3)
C(47)	44(4)	42(4)	73(5)	-16(4)	-21(4)	1(3)
C(48)	59(5)	65(5)	50(4)	-22(4)	4(4)	9(4)
C(49)	64(5)	73(5)	55(5)	-22(4)	-21(4)	11(4)
C(50)	67(5)	43(4)	74(6)	-3(4)	7(4)	-11(4)
C(51)	100(6)	37(4)	65(5)	-10(4)	-16(5)	14(4)
C(52)	79(6)	84(6)	40(4)	-19(4)	2(4)	7(5)
C(53)	60(5)	84(6)	37(4)	9(4)	-5(4)	11(4)
C(54)	72(5)	80(6)	48(5)	7(4)	-15(4)	12(5)
C(55)	48(4)	55(4)	40(4)	5(3)	-17(3)	3(3)
C(56)	53(4)	49(4)	44(4)	-3(3)	-6(3)	-18(3)
C(57)	77(5)	41(4)	47(4)	0(3)	-29(4)	1(4)
C(58)	41(4)	52(4)	61(5)	-14(4)	-23(3)	4(3)
C(59)	56(4)	40(4)	43(4)	-8(3)	-15(3)	1(3)
C(60)	42(4)	73(5)	42(4)	-22(4)	-18(3)	-1(4)
C(61)	31(3)	41(3)	25(3)	1(3)	-13(2)	-5(3)

C(62)	41(3)	53(4)	28(3)	-5(3)	-15(3)	-3(3)
C(63)	65(5)	53(5)	62(5)	-6(4)	-36(4)	-9(4)
C(64)	68(5)	36(4)	51(4)	2(3)	-25(4)	-19(3)
C(65)	51(4)	36(3)	32(3)	0(3)	-9(3)	0(3)
C(66)	37(3)	33(3)	25(3)	-2(2)	-5(3)	-2(3)
C(67)	61(4)	56(4)	35(4)	-7(3)	-12(3)	-6(4)
C(68)	48(4)	52(4)	43(4)	-13(3)	-9(3)	4(3)
C(69)	40(4)	37(4)	38(4)	1(3)	1(3)	-6(3)
C(70)	65(4)	39(4)	35(4)	1(3)	-23(3)	0(3)
C(71)	34(3)	31(3)	39(3)	-9(3)	-4(3)	-1(3)
C(72)	38(4)	37(4)	85(6)	-9(4)	-14(4)	0(3)
C(73)	51(4)	41(4)	77(6)	-4(4)	-25(4)	-6(3)
C(74)	43(4)	48(4)	64(5)	-7(4)	-9(4)	-5(3)
C(75)	58(4)	33(4)	66(5)	-5(3)	-16(4)	5(3)
C(76)	43(4)	30(3)	39(4)	2(3)	-5(3)	2(3)
C(77)	43(4)	67(5)	67(5)	-30(4)	-9(4)	17(4)
C(78)	57(4)	42(4)	47(4)	3(3)	-19(3)	-1(3)
C(79)	46(4)	44(4)	41(4)	-3(3)	-1(3)	-7(3)
C(80)	41(3)	42(4)	45(4)	-12(3)	-20(3)	16(3)
C(81)	43(4)	45(4)	54(4)	-15(3)	-15(3)	-2(3)
C(82)	51(4)	50(4)	74(6)	-11(4)	-15(4)	-7(4)
C(83)	91(7)	49(5)	99(7)	-17(5)	-50(6)	-7(5)
C(84)	73(5)	66(5)	70(6)	-27(4)	-36(5)	4(4)
C(85)	64(5)	65(5)	68(5)	-33(4)	-36(4)	4(4)
C(86)	49(4)	53(4)	45(4)	-19(3)	-26(3)	24(3)
C(87)	58(4)	54(4)	43(4)	1(3)	-26(4)	-11(4)
C(88)	53(4)	58(5)	46(4)	-9(4)	8(4)	11(4)
C(89)	86(6)	64(5)	55(5)	4(4)	-41(5)	21(4)
C(90)	96(6)	61(5)	40(4)	-11(4)	8(4)	6(5)
C(91)	48(4)	37(4)	38(4)	-2(3)	-7(3)	-7(3)
C(92)	57(5)	61(5)	79(6)	-14(4)	-26(4)	-4(4)
C(93)	73(5)	56(5)	61(5)	6(4)	-25(4)	-22(4)
C(94)	78(6)	43(4)	71(6)	0(4)	-30(5)	-11(4)
C(95)	66(5)	39(4)	56(5)	2(3)	-24(4)	-5(4)
C(96)	45(4)	37(4)	39(4)	-6(3)	-17(3)	-6(3)
C(97)	78(5)	41(4)	50(4)	7(3)	-31(4)	-2(4)
C(98)	42(4)	64(5)	68(5)	-4(4)	-6(4)	14(4)
C(99)	61(5)	60(5)	62(5)	-23(4)	8(4)	8(4)
C(100)	59(5)	68(5)	66(5)	-2(4)	-24(4)	3(4)
C(300)	74(6)	57(5)	87(7)	-11(5)	-27(5)	-5(4)

C(301)	59(5)	67(5)	35(4)	-12(3)	-15(3)	2(4)
C(302)	44(4)	61(5)	54(5)	-15(4)	-10(3)	7(4)
C(303)	51(4)	77(6)	47(5)	-11(4)	-2(4)	-6(4)
C(304)	77(6)	39(4)	44(4)	-4(3)	-3(4)	-11(4)
C(305)	68(5)	67(6)	47(5)	-5(4)	-11(4)	20(4)
C(306)	40(4)	74(6)	61(5)	-13(4)	-32(4)	11(4)
C(331)	68(4)	70(5)	29(3)	-9(3)	-9(3)	13(3)
C(332)	87(5)	77(5)	103(7)	-31(5)	-48(5)	12(4)
C(333)	91(5)	72(5)	103(7)	-2(5)	-59(5)	15(4)
C(334)	88(5)	68(5)	69(5)	1(4)	-16(5)	-2(4)
C(335)	64(4)	99(5)	55(5)	-19(5)	-5(4)	-19(4)
C(336)	71(5)	129(6)	50(5)	-26(5)	-27(4)	33(5)

Table 5. Hydrogen coordinates (x 10^4) and isotropic displacement parameters ($Å^2$x 10^3) for srgr1014.

	x	y	z	U(eq)
H(1A)	6811	7311	6945	68
H(1B)	5686	7437	7481	68
H(1C)	6863	7484	7671	68
H(2A)	6187	9125	5875	71
H(2B)	5305	8505	6202	71
H(2C)	6539	8341	5810	71
H(3A)	5679	8957	7945	69
H(3B)	4750	8770	7577	69
H(3C)	5450	9479	7293	69
H(4A)	9632	9235	5409	68
H(4B)	8989	9915	5141	68
H(4C)	8389	9169	5321	68
H(5A)	9206	10103	7066	85
H(5B)	9880	10323	6290	85
H(5C)	10047	9568	6710	85
H(6A)	6685	10191	6094	83
H(6B)	7627	10754	6037	83
H(6C)	6916	10394	6770	83
H(7A)	11094	8965	5770	63
H(7B)	12374	8743	5659	63
H(7C)	11538	8512	6387	63
H(8A)	12683	7057	6024	65
H(8B)	13189	7470	5269	65
H(8C)	12411	6801	5382	65
H(9A)	10927	7638	4556	64
H(9B)	11787	8268	4430	64
H(9C)	10486	8383	4713	64
H(10A)	7712	7376	5821	69
H(10B)	8106	6899	5256	69
H(10C)	8700	7634	5170	69
H(11A)	10672	6489	4989	81
H(11B)	9741	5893	5294	81
H(11C)	10755	5928	5650	81
H(12A)	9115	5860	6921	76

H(12B)	8050	5935	6596	76
H(12C)	8224	6457	7080	76
H(13A)	13046	7847	6766	58
H(13B)	13794	7991	7268	58
H(13C)	12472	8077	7476	58
H(14A)	12644	6888	8869	67
H(14B)	13858	7188	8480	67
H(14C)	13633	6366	8650	67
H(15A)	14112	5919	7383	66
H(15B)	14753	6639	7037	66
H(15C)	13836	6368	6706	66
H(16A)	9630	5463	8762	79
H(16B)	9530	5146	8112	79
H(16C)	9335	5959	8114	79
H(17A)	12886	5139	8213	63
H(17B)	11830	4626	8456	63
H(17C)	11946	5219	8879	63
H(18A)	11165	5692	6736	64
H(18B)	11481	4937	7092	64
H(18C)	12418	5531	6801	64
H(19A)	10979	6000	9452	72
H(19B)	10301	5608	10181	72
H(19C)	11259	6156	10135	72
H(20A)	7743	6775	9816	66
H(20B)	8197	5995	9903	66
H(20C)	8566	6512	9186	66
H(21A)	9699	7002	11053	71
H(21B)	8849	6372	11155	71
H(21C)	8468	7156	10943	71
H(22A)	9655	8958	9946	70
H(22B)	10567	8910	10393	70
H(22C)	9577	8349	10595	70
H(23A)	11606	7391	10613	70
H(23B)	12486	8001	10236	70
H(23C)	12475	7314	9917	70
H(24A)	12522	8381	8720	71
H(24B)	12091	9051	9046	71
H(24C)	11440	8781	8553	71
H(25A)	5404	9439	9711	81
H(25B)	4619	8782	9785	81

H(25C)	5119	9236	9048	81
H(26A)	7258	7760	10275	73
H(26B)	5999	7962	10580	73
H(26C)	6980	8516	10461	73
H(27A)	5713	7865	8730	63
H(27B)	5707	7381	9452	63
H(27C)	6827	7481	8863	63
H(28A)	9934	9697	8913	79
H(28B)	9656	10395	8439	79
H(28C)	9807	9680	8157	79
H(29A)	7401	10256	7895	75
H(29B)	7475	10823	8355	75
H(29C)	6436	10299	8559	75
H(30A)	6880	10065	9905	80
H(30B)	8119	10359	9772	80
H(30C)	7841	9563	10115	80
H(31A)	14421	11027	11391	85
H(31B)	15266	11233	10662	85
H(31C)	14539	10534	10836	85
H(32A)	14009	10963	9582	96
H(32B)	14368	11766	9380	96
H(32C)	13110	11545	9439	96
H(33A)	13122	12747	10232	84
H(33B)	14317	12625	10407	84
H(33C)	13218	12578	11010	84
H(34A)	10710	9985	12028	74
H(34B)	10015	9772	11527	74
H(34C)	9877	10530	11724	74
H(35A)	13094	9863	10353	78
H(35B)	12126	9328	10786	78
H(35C)	12910	9664	11165	78
H(36A)	10499	11078	10092	83
H(36B)	10446	10257	10117	83
H(36C)	11534	10693	9687	83
H(37A)	9078	13567	9290	82
H(37B)	9875	14193	9293	82
H(37C)	8815	13983	9912	82
H(38A)	11783	12518	9530	77
H(38B)	11511	13142	8968	77
H(38C)	10742	12458	9203	77

H(39A)	10722	14099	10541	83
H(39B)	11756	13958	9948	83
H(39C)	11524	13454	10676	83
H(40A)	8558	11020	11079	67
H(40B)	7279	11232	11293	67
H(40C)	8149	11432	11697	67
H(41A)	8384	12434	9475	71
H(41B)	7683	11740	9842	71
H(41C)	9014	11723	9667	71
H(42A)	7018	12900	11299	79
H(42B)	6432	12562	10814	79
H(42C)	7217	13233	10505	79
H(43A)	10576	14673	12252	84
H(43B)	10514	14979	11482	84
H(43C)	10798	14176	11696	84
H(44A)	8845	14435	10888	85
H(44B)	8336	15128	11146	85
H(44C)	7592	14435	11329	85
H(45A)	7358	14897	12752	82
H(45B)	8352	15444	12409	82
H(45C)	8435	14872	13062	82
H(46A)	6985	12172	12468	69
H(46B)	6511	12074	13277	69
H(46C)	7820	12025	12968	69
H(47A)	6021	14147	12606	77
H(47B)	5370	13417	12817	77
H(47C)	6140	13639	12065	77
H(48A)	7576	13169	13981	89
H(48B)	6270	13211	14012	89
H(48C)	7024	13900	13751	89
H(49A)	11505	13409	14846	93
H(49B)	12548	13764	14278	93
H(49C)	12413	12935	14448	93
H(50A)	12905	13305	12905	100
H(50B)	12521	14100	12807	100
H(50C)	11931	13543	12519	100
H(51A)	9757	14192	13564	102
H(51B)	10783	14605	13665	102
H(51C)	9955	14162	14312	102
H(52A)	9534	13086	15217	104

H(52B)	8612	12490	15549	104
H(52C)	8423	13091	14942	104
H(53A)	8087	12002	14132	96
H(53B)	8216	11437	14781	96
H(53C)	8960	11383	14041	96
H(54A)	10992	11318	14644	104
H(54B)	10136	11354	15355	104
H(54C)	11147	11899	15071	104
H(55A)	13311	10113	14114	73
H(55B)	12231	9622	14391	73
H(55C)	12172	10408	14512	73
H(56A)	10175	10610	13812	75
H(56B)	10343	9796	13804	75
H(56C)	10314	10326	13113	75
H(57A)	12464	9833	12373	80
H(57B)	12390	9251	13043	80
H(57C)	13494	9716	12730	80
H(58A)	14799	10511	13196	74
H(58B)	15614	11122	12735	74
H(58C)	14893	10694	12391	74
H(59A)	14313	12088	11754	68
H(59B)	14909	12465	12207	68
H(59C)	13633	12625	12196	68
H(60A)	14334	12222	13619	74
H(60B)	13981	11467	14060	74
H(60C)	13046	12023	13916	74
H(61)	7271	18452	13509	39
H(62A)	6011	19478	14184	48
H(62B)	6775	18908	14518	48
H(63A)	7657	20006	14179	68
H(63B)	8376	19437	13798	68
H(64A)	8353	20416	13007	61
H(64B)	7026	20464	13235	61
H(65A)	7467	20003	12222	49
H(65B)	8232	19419	12538	49
H(66)	5861	19481	12967	39
H(67A)	6540	17520	14306	76
H(67B)	5237	17341	14507	76
H(67C)	5702	17932	14816	76
H(68A)	4527	18731	14484	71

H(68B)	3943	18262	14100	71
H(68C)	4488	18997	13703	71
H(69A)	7389	18218	11705	62
H(69B)	7874	18191	12368	62
H(69C)	8010	18884	11801	62
H(70A)	5180	19323	12098	68
H(70B)	5841	18895	11536	68
H(70C)	6383	19582	11648	68
H(71)	4506	16751	12121	42
H(72A)	2589	16618	12438	64
H(72B)	2638	16388	13220	64
H(73A)	2464	15380	12752	67
H(73B)	3579	15592	12171	67
H(74A)	4008	14769	13062	64
H(74B)	3465	15250	13603	64
H(75A)	5473	15551	12568	63
H(75B)	5387	15383	13373	63
H(76)	4508	16423	13522	47
H(77A)	3190	18119	13297	86
H(77B)	3214	17315	13636	86
H(77C)	2407	17570	13133	86
H(78A)	4399	17861	11627	73
H(78B)	3700	18408	12047	73
H(78C)	3094	17740	11943	73
H(79A)	6822	16288	12186	68
H(79B)	7329	17034	12171	68
H(79C)	6234	16980	11892	68
H(80A)	6099	16374	13889	61
H(80B)	7189	16788	13430	61
H(80C)	6930	16028	13319	61
H(81)	2598	11310	16767	55
H(82A)	3479	10044	17340	70
H(82B)	2208	10280	17526	70
H(83A)	2456	9471	16781	90
H(83B)	2005	10196	16440	90
H(84A)	3466	9792	15655	77
H(84B)	4273	9783	16166	77
H(85A)	3306	11031	15545	71
H(85B)	4591	10828	15324	71
H(86)	4861	10918	16411	55

H(87A)	4408	11245	18147	76
H(87B)	4813	10800	17555	76
H(87C)	3717	10571	18141	76
H(88A)	1888	11760	17693	84
H(88B)	2646	11931	18179	84
H(88C)	2176	11159	18269	84
H(89A)	3937	12763	15466	99
H(89B)	2962	12327	16026	99
H(89C)	3508	12048	15337	99
H(90A)	6116	11678	15721	104
H(90B)	5828	12469	15452	104
H(90C)	5501	11861	15105	104
H(91)	6206	13312	16409	50
H(92A)	7850	13364	16735	76
H(92B)	7212	13492	17477	76
H(93A)	7321	14464	16229	76
H(93B)	7926	14560	16818	76
H(94A)	6166	14654	17578	76
H(94B)	6263	15234	16902	76
H(95A)	4512	14650	17180	64
H(95B)	5219	14525	16451	64
H(96)	5036	13577	17702	47
H(97A)	6552	12539	18034	83
H(97B)	5944	11821	18065	83
H(97C)	5226	12508	18161	83
H(98A)	7596	12145	17051	90
H(98B)	7151	12273	16366	90
H(98C)	6738	11606	16944	90
H(99A)	2990	13833	17767	95
H(99B)	3326	13088	18133	95
H(99C)	2461	13141	17655	95
H(10D)	3162	13359	16421	96
H(10E)	4339	13751	16135	96
H(10F)	3338	14098	16610	96
H(30D)	15961	15957	9227	107
H(30E)	15894	16026	8447	107
H(30F)	17044	15824	8663	107
H(302)	14272	15132	8993	63
H(303)	13792	14001	9120	72
H(304)	15177	13131	9171	66

H(305)	17041	13484	9057	75
H(306)	17449	14659	8951	66
H(31D)	10145	5984	3316	158
H(31E)	10628	6123	2507	158
H(31F)	9312	6111	2814	158
H(312)	9294	6961	3874	69
H(313)	9325	8164	3828	53
H(314)	10112	8902	2802	60
H(315)	10867	8437	1821	61
H(316)	10836	7234	1867	60
H(32D)	2988	15540	15245	119
H(32E)	3140	14714	15379	119
H(32F)	3389	15143	14615	119
H(322)	5355	14413	14660	70
H(323)	7190	14738	14646	69
H(324)	7552	15580	15159	85
H(325)	6086	16267	15560	69
H(326)	4233	16049	15566	46
H(33D)	4424	3985	11451	84
H(33E)	5216	4040	10704	84
H(33F)	3894	4008	10806	84
H(332)	2904	5022	10771	100
H(333)	2674	6192	10742	101
H(334)	4144	6861	10712	92
H(335)	5825	6444	10728	88
H(336)	6079	5168	10904	97
H(34D)	145	14020	15821	164
H(34E)	1424	13916	15850	164
H(34F)	663	14431	16284	164
H(341)	715	12668	16105	50
H(342)	59	11739	16996	77
H(344)	-682	11947	18100	60
H(345)	-767	13084	18315	73
H(346)	-111	14013	17424	77
H(401)	10435	6668	2201	59
H(402)	10894	7804	1620	95
H(403)	10573	8732	2213	76
H(404)	9793	8525	3388	112
H(405)	9333	7389	3970	60
H(411)	-781	12777	18291	83

H(412)	-918	11593	18264	117
H(413)	-174	11178	17253	102
H(414)	706	11946	16270	143
H(415)	843	13130	16297	154
H(42D)	2662	15313	14839	90
H(42E)	2493	14939	15619	90
H(42F)	2844	14493	15024	90
H(421)	4985	14206	14622	70
H(422)	6770	14562	14628	35
H(423)	7003	15564	15054	112
H(424)	5451	16210	15473	65
H(425)	3666	15855	15466	62
H(40D)	9827	5866	3218	150
H(40E)	8885	6136	3791	150
H(40F)	10155	6108	3851	150
H(41D)	-800	13906	17583	164
H(41E)	48	14077	16851	164
H(41F)	509	13953	17531	164

X-ray Data for **5** **(PAGE 167 – 200)**

Table 1. Crystal data and structure refinement for srgr1101.

Identification code	srgr1101	
Empirical formula	C64 H160 I4 Li8 N12 Si8	
Formula weight	1885.88	
Temperature	123(2) K	
Wavelength	0.71073 Å	
Crystal system	Orthorhombic	
Space group	P 21 21 21	
Unit cell dimensions	a = 14.2443(3) Å	α= 90°.
	b = 19.9051(4) Å	β= 90°.
	c = 35.7460(8) Å	γ = 90°.
Volume	10135.2(4) $Å^3$	
Z	4	
Density (calculated)	1.236 Mg/m^3	
Absorption coefficient	1.361 mm^{-1}	
F(000)	3904	
Crystal size	0.16 x 0.16 x 0.12 mm^3	
Theta range for data collection	2.69 to 28.00°.	
Index ranges	-17<=h<=18, -26<=k<=25, -44<=l<=47	
Reflections collected	36095	
Independent reflections	19991 [R(int) = 0.0436]	
Completeness to theta = 27.00°	99.8 %	
Absorption correction	Semi-empirical from equivalents	
Max. and min. transmission	1.00000 and 0.87734	
Refinement method	Full-matrix least-squares on F^2	
Data / restraints / parameters	19991 / 0 / 905	
Goodness-of-fit on F^2	1.082	
Final R indices [I>2sigma(I)]	R1 = 0.0656, wR2 = 0.1131	
R indices (all data)	R1 = 0.0911, wR2 = 0.1235	
Absolute structure parameter	0.000(17)	
Largest diff. peak and hole	1.182 and -0.838 e.$Å^{-3}$	

Table 2. Atomic coordinates (x 10^4) and equivalent isotropic displacement parameters ($Å^2$x 10^3) for srgr1101. U(eq) is defined as one third of the trace of the orthogonalized U^{ij} tensor.

	x	y	z	U(eq)
I(1)	-5545(1)	-8450(1)	1064(1)	43(1)
I(2)	-3841(1)	-6740(1)	1220(1)	48(1)
I(3)	-598(1)	-3439(1)	1380(1)	47(1)
I(4)	1135(1)	-1736(1)	1339(1)	45(1)
Si(5)	4353(2)	-604(1)	1594(1)	28(1)
Si(6)	-699(2)	-5625(1)	875(1)	26(1)
Si(7)	4001(2)	-982(1)	801(1)	26(1)
Si(8)	1421(2)	648(1)	860(1)	24(1)
Si(9)	-970(2)	-5914(1)	1690(1)	26(1)
Si(10)	1323(2)	473(1)	1687(1)	26(1)
Si(11)	-3574(2)	-4299(1)	1673(1)	28(1)
Si(12)	-3720(2)	-4517(1)	851(1)	29(1)
N(4)	3698(4)	-565(3)	1198(1)	24(1)
N(3)	1862(4)	357(2)	1266(1)	20(1)
N(1)	-3157(4)	-4624(3)	1266(1)	23(1)
N(2)	-1286(4)	-5533(2)	1284(1)	20(1)
N(99)	-5360(4)	-7403(3)	2083(2)	28(1)
N(97)	-485(5)	-2326(3)	429(2)	29(1)
N(96)	-1610(4)	-1654(3)	974(1)	31(1)
N(94)	-2767(5)	-8589(3)	825(2)	35(2)
N(98)	-6674(4)	-6828(3)	1569(2)	31(1)
C(16)	4553(6)	-1831(4)	890(2)	43(2)
C(19)	845(5)	1506(3)	894(2)	33(2)
C(88)	-1396(6)	-2035(4)	313(2)	32(2)
C(4)	-490(6)	-6798(4)	1635(2)	41(2)
N(95)	-3617(4)	-7775(3)	263(1)	29(1)
C(98)	-5533(6)	-8120(3)	2153(2)	42(2)
C(20)	538(6)	65(4)	649(2)	39(2)
C(5)	-2020(6)	-6012(4)	2003(2)	34(2)
C(7)	-4192(6)	-3468(4)	1625(2)	44(2)
C(22)	1631(6)	1306(3)	1904(2)	38(2)
C(99)	-7398(6)	-7075(4)	1312(2)	46(2)
C(78)	-2022(6)	-8253(5)	1041(2)	50(2)
C(92)	-6111(5)	-6957(3)	2226(2)	26(2)
C(8)	-2617(5)	-4123(4)	2022(2)	36(2)

C(13)	5668(6)	-648(4)	1525(2)	48(2)
C(1)	-1130(6)	-6364(4)	597(2)	43(2)
C(14)	4211(6)	182(4)	1882(2)	46(2)
C(89)	-384(6)	-3028(4)	303(2)	44(2)
C(74)	-4345(5)	-8141(4)	61(2)	39(2)
C(86)	-2406(5)	-2083(4)	1099(2)	43(2)
C(21)	2380(5)	751(4)	502(2)	35(2)
C(23)	4(6)	433(4)	1676(2)	45(2)
C(9)	-4419(6)	-4891(4)	1910(2)	49(2)
C(84)	335(5)	-1941(4)	291(2)	45(2)
C(17)	2950(6)	-1151(4)	502(2)	44(2)
C(77)	-2527(6)	-8693(4)	424(2)	41(2)
C(96)	-6967(5)	-6955(3)	1965(2)	28(2)
C(2)	616(6)	-5722(4)	914(2)	51(2)
C(10)	-5050(6)	-4555(5)	871(2)	57(3)
C(3)	-842(7)	-4856(4)	571(2)	54(3)
C(97)	-4432(6)	-7226(4)	2231(2)	42(2)
C(15)	4029(7)	-1324(4)	1899(2)	56(3)
C(93)	-7190(6)	-6636(5)	2765(2)	54(2)
C(6)	-64(6)	-5419(4)	1960(2)	46(2)
C(24)	1654(6)	-184(4)	2038(2)	46(2)
C(11)	-3434(6)	-5203(4)	508(2)	47(2)
C(95)	-7725(6)	-6486(4)	2108(2)	46(2)
C(87)	-1672(6)	-1454(4)	573(2)	35(2)
C(18)	4843(6)	-496(4)	508(2)	40(2)
C(12)	-3394(7)	-3710(4)	613(2)	58(3)
C(94)	-8024(6)	-6671(5)	2509(2)	60(3)
C(76)	-2662(5)	-8056(4)	200(2)	32(2)
C(85)	-1534(7)	-1067(4)	1220(2)	47(2)
Li(4)	-1736(9)	-4567(6)	1293(3)	29(3)
C(79)	-3002(7)	-9227(4)	1013(3)	61(3)
C(75)	-3656(6)	-7063(3)	157(2)	41(2)
N(92)	1149(4)	-3072(3)	2234(2)	30(1)
N(93)	2231(4)	-3573(3)	1627(2)	33(2)
C(67)	2997(6)	-3271(5)	1418(2)	57(3)
C(62)	2518(5)	-3726(4)	2020(2)	33(2)
C(91)	-6416(6)	-7120(4)	2629(2)	42(2)
C(72)	-1413(7)	-8407(5)	-258(2)	66(3)
C(66)	1908(6)	-4163(4)	1417(2)	47(2)
C(80)	-2631(7)	-1161(4)	462(2)	51(2)

C(69)	1637(6)	-2467(4)	2375(2)	49(2)
C(73)	-2405(6)	-8150(4)	-214(2)	44(2)
C(83)	-1443(7)	-1817(4)	-95(2)	53(2)
C(61)	3052(6)	-4391(4)	2061(2)	51(2)
C(68)	2047(6)	-3818(4)	2692(2)	45(2)
Li(7)	2295(10)	-607(6)	1266(3)	34(3)
Li(3)	-2708(9)	-5587(6)	1267(3)	30(3)
C(63)	227(6)	-3117(4)	2415(2)	50(2)
C(60)	1701(5)	-3689(3)	2289(2)	31(2)
Li(2)	-3869(9)	-7912(6)	822(3)	30(3)
C(64)	2564(6)	-4485(4)	2724(2)	59(3)
C(71)	-1316(7)	-9068(5)	-59(2)	70(3)
Li(5)	1064(10)	-2959(6)	1669(3)	37(3)
C(65)	3381(7)	-4517(5)	2458(2)	60(3)
C(70)	-1540(7)	-8989(5)	353(2)	63(3)
C(90)	-6475(7)	-6114(4)	1489(2)	49(2)
C(81)	-2660(8)	-940(6)	58(3)	80(3)
Li(1)	-5433(9)	-7316(5)	1505(3)	30(3)
Li(6)	-447(9)	-2247(7)	1000(3)	36(3)
C(82)	-2395(8)	-1540(6)	-195(2)	85(4)
Li(8)	3277(9)	408(5)	1186(3)	28(3)

Table 3. Bond lengths [Å] and angles [°] for srgr1101.

I(1)-Li(2)	2.755(13)
I(1)-Li(1)	2.756(11)
I(1)-Li(8)#1	2.860(11)
I(2)-Li(2)	2.734(11)
I(2)-Li(1)	2.738(12)
I(2)-Li(3)	2.810(13)
I(3)-Li(6)	2.743(13)
I(3)-Li(5)	2.754(13)
I(3)-Li(4)	2.786(12)
I(4)-Li(5)	2.709(12)
I(4)-Li(6)	2.752(12)
I(4)-Li(7)	2.801(13)
Si(5)-N(4)	1.697(5)
Si(5)-C(15)	1.857(7)
Si(5)-C(14)	1.883(7)
Si(5)-C(13)	1.892(8)
Si(5)-Li(8)	2.922(12)
Si(5)-Li(7)	3.157(14)
Si(6)-N(2)	1.694(5)
Si(6)-C(1)	1.878(7)
Si(6)-C(2)	1.888(8)
Si(6)-C(3)	1.888(8)
Si(6)-Li(4)	2.974(12)
Si(6)-Li(3)	3.187(13)
Si(7)-N(4)	1.700(5)
Si(7)-C(18)	1.864(8)
Si(7)-C(17)	1.871(8)
Si(7)-C(16)	1.890(8)
Si(7)-Li(7)	3.037(14)
Si(8)-N(3)	1.684(5)
Si(8)-C(20)	1.870(7)
Si(8)-C(21)	1.883(7)
Si(8)-C(19)	1.900(7)
Si(8)-Li(8)	2.929(13)
Si(8)-Li(7)	3.147(12)
Si(9)-N(2)	1.701(5)
Si(9)-C(5)	1.876(8)
Si(9)-C(6)	1.888(8)

Si(9)-C(4)	1.897(7)
Si(9)-Li(3)	2.974(13)
Si(10)-N(3)	1.706(5)
Si(10)-C(24)	1.875(7)
Si(10)-C(22)	1.881(7)
Si(10)-C(23)	1.882(8)
Si(10)-Li(7)	2.967(13)
Si(11)-N(1)	1.700(5)
Si(11)-C(7)	1.881(8)
Si(11)-C(8)	1.882(7)
Si(11)-C(9)	1.886(8)
Si(11)-Li(4)	2.998(12)
Si(12)-N(1)	1.698(6)
Si(12)-C(12)	1.877(8)
Si(12)-C(11)	1.881(7)
Si(12)-C(10)	1.897(9)
Si(12)-Li(3)	2.970(12)
N(4)-Li(7)	2.015(15)
N(4)-Li(8)	2.028(12)
N(3)-Li(7)	2.016(14)
N(3)-Li(8)	2.037(15)
N(1)-Li(3)	2.021(13)
N(1)-Li(4)	2.031(14)
N(2)-Li(4)	2.027(13)
N(2)-Li(3)	2.029(15)
N(99)-C(97)	1.467(9)
N(99)-C(98)	1.470(8)
N(99)-C(92)	1.480(8)
N(99)-Li(1)	2.076(12)
N(97)-C(89)	1.475(8)
N(97)-C(88)	1.481(10)
N(97)-C(84)	1.482(9)
N(97)-Li(6)	2.050(12)
N(96)-C(85)	1.467(8)
N(96)-C(86)	1.488(9)
N(96)-C(87)	1.491(8)
N(96)-Li(6)	2.036(15)
N(94)-C(78)	1.472(9)
N(94)-C(79)	1.474(9)
N(94)-C(77)	1.488(9)

N(94)-Li(2)	2.069(14)
N(98)-C(99)	1.466(8)
N(98)-C(90)	1.476(9)
N(98)-C(96)	1.499(8)
N(98)-Li(1)	2.029(14)
C(16)-H(16A)	0.9800
C(16)-H(16B)	0.9800
C(16)-H(16C)	0.9800
C(19)-H(19A)	0.9800
C(19)-H(19B)	0.9800
C(19)-H(19C)	0.9800
C(88)-C(83)	1.523(9)
C(88)-C(87)	1.534(10)
C(88)-H(88A)	1.0000
C(4)-H(4A)	0.9800
C(4)-H(4B)	0.9800
C(4)-H(4C)	0.9800
N(95)-C(74)	1.457(8)
N(95)-C(75)	1.469(8)
N(95)-C(76)	1.487(9)
N(95)-Li(2)	2.047(12)
C(98)-H(98A)	0.9800
C(98)-H(98B)	0.9800
C(98)-H(98C)	0.9800
C(20)-H(20A)	0.9800
C(20)-H(20B)	0.9800
C(20)-H(20C)	0.9800
C(5)-H(5A)	0.9800
C(5)-H(5B)	0.9800
C(5)-H(5C)	0.9800
C(7)-H(7A)	0.9800
C(7)-H(7B)	0.9800
C(7)-H(7C)	0.9800
C(22)-H(22A)	0.9800
C(22)-H(22B)	0.9800
C(22)-H(22C)	0.9800
C(99)-H(99A)	0.9800
C(99)-H(99B)	0.9800
C(99)-H(99C)	0.9800
C(78)-H(78A)	0.9800

C(78)-H(78B)	0.9800
C(78)-H(78C)	0.9800
C(92)-C(96)	1.535(9)
C(92)-C(91)	1.539(9)
C(92)-H(92A)	1.0000
C(8)-H(8A)	0.9800
C(8)-H(8B)	0.9800
C(8)-H(8C)	0.9800
C(13)-H(13A)	0.9800
C(13)-H(13B)	0.9800
C(13)-H(13C)	0.9800
C(1)-H(1A)	0.9800
C(1)-H(1B)	0.9800
C(1)-H(1C)	0.9800
C(14)-H(14A)	0.9800
C(14)-H(14B)	0.9800
C(14)-H(14C)	0.9800
C(89)-H(89A)	0.9800
C(89)-H(89B)	0.9800
C(89)-H(89C)	0.9800
C(74)-H(74A)	0.9800
C(74)-H(74B)	0.9800
C(74)-H(74C)	0.9800
C(86)-H(86A)	0.9800
C(86)-H(86B)	0.9800
C(86)-H(86C)	0.9800
C(21)-H(21A)	0.9800
C(21)-H(21B)	0.9800
C(21)-H(21C)	0.9800
C(23)-H(23A)	0.9800
C(23)-H(23B)	0.9800
C(23)-H(23C)	0.9800
C(9)-H(9A)	0.9800
C(9)-H(9B)	0.9800
C(9)-H(9C)	0.9800
C(84)-H(84A)	0.9800
C(84)-H(84B)	0.9800
C(84)-H(84C)	0.9800
C(17)-H(17A)	0.9800
C(17)-H(17B)	0.9800

C(17)-H(17C)	0.9800
C(77)-C(76)	1.514(10)
C(77)-C(70)	1.545(12)
C(77)-H(77A)	1.0000
C(96)-C(95)	1.517(10)
C(96)-H(96A)	1.0000
C(2)-H(2A)	0.9800
C(2)-H(2B)	0.9800
C(2)-H(2C)	0.9800
C(10)-H(10A)	0.9800
C(10)-H(10B)	0.9800
C(10)-H(10C)	0.9800
C(3)-H(3A)	0.9800
C(3)-H(3B)	0.9800
C(3)-H(3C)	0.9800
C(97)-H(97A)	0.9800
C(97)-H(97B)	0.9800
C(97)-H(97C)	0.9800
C(15)-H(15A)	0.9800
C(15)-H(15B)	0.9800
C(15)-H(15C)	0.9800
C(93)-C(94)	1.503(11)
C(93)-C(91)	1.542(11)
C(93)-H(93A)	0.9900
C(93)-H(93B)	0.9900
C(6)-H(6A)	0.9800
C(6)-H(6B)	0.9800
C(6)-H(6C)	0.9800
C(24)-H(24A)	0.9800
C(24)-H(24B)	0.9800
C(24)-H(24C)	0.9800
C(11)-H(11A)	0.9800
C(11)-H(11B)	0.9800
C(11)-H(11C)	0.9800
C(95)-C(94)	1.538(10)
C(95)-H(95A)	0.9900
C(95)-H(95B)	0.9900
C(87)-C(80)	1.537(11)
C(87)-H(87A)	1.0000
C(18)-H(18A)	0.9800

C(18)-H(18B)	0.9800
C(18)-H(18C)	0.9800
C(12)-H(12A)	0.9800
C(12)-H(12B)	0.9800
C(12)-H(12C)	0.9800
C(94)-H(94A)	0.9900
C(94)-H(94B)	0.9900
C(76)-C(73)	1.537(9)
C(76)-H(76A)	1.0000
C(85)-H(85A)	0.9800
C(85)-H(85B)	0.9800
C(85)-H(85C)	0.9800
Li(4)-Li(3)	2.460(17)
C(79)-H(79A)	0.9800
C(79)-H(79B)	0.9800
C(79)-H(79C)	0.9800
C(75)-H(75A)	0.9800
C(75)-H(75B)	0.9800
C(75)-H(75C)	0.9800
N(92)-C(63)	1.465(9)
N(92)-C(60)	1.471(9)
N(92)-C(69)	1.479(9)
N(92)-Li(5)	2.037(13)
N(93)-C(67)	1.452(9)
N(93)-C(66)	1.468(9)
N(93)-C(62)	1.495(9)
N(93)-Li(5)	2.069(15)
C(67)-H(67A)	0.9800
C(67)-H(67B)	0.9800
C(67)-H(67C)	0.9800
C(62)-C(60)	1.512(10)
C(62)-C(61)	1.534(10)
C(62)-H(62A)	1.0000
C(91)-H(91A)	0.9900
C(91)-H(91B)	0.9900
C(72)-C(71)	1.502(13)
C(72)-C(73)	1.511(11)
C(72)-H(72A)	0.9900
C(72)-H(72B)	0.9900
C(66)-H(66A)	0.9800

C(66)-H(66B)	0.9800
C(66)-H(66C)	0.9800
C(80)-C(81)	1.512(11)
C(80)-H(80A)	0.9900
C(80)-H(80B)	0.9900
C(69)-H(69A)	0.9800
C(69)-H(69B)	0.9800
C(69)-H(69C)	0.9800
C(73)-H(73A)	0.9900
C(73)-H(73B)	0.9900
C(83)-C(82)	1.507(12)
C(83)-H(83A)	0.9900
C(83)-H(83B)	0.9900
C(61)-C(65)	1.514(11)
C(61)-H(61A)	0.9900
C(61)-H(61B)	0.9900
C(68)-C(64)	1.522(11)
C(68)-C(60)	1.546(9)
C(68)-H(68A)	0.9900
C(68)-H(68B)	0.9900
Li(7)-Li(8)	2.474(16)
C(63)-H(63A)	0.9800
C(63)-H(63B)	0.9800
C(63)-H(63C)	0.9800
C(60)-H(60A)	1.0000
Li(2)-Li(1)	3.512(17)
C(64)-C(65)	1.505(11)
C(64)-H(64A)	0.9900
C(64)-H(64B)	0.9900
C(71)-C(70)	1.518(11)
C(71)-H(71A)	0.9900
C(71)-H(71B)	0.9900
Li(5)-Li(6)	3.515(19)
C(65)-H(65A)	0.9900
C(65)-H(65B)	0.9900
C(70)-H(70A)	0.9900
C(70)-H(70B)	0.9900
C(90)-H(90A)	0.9800
C(90)-H(90B)	0.9800
C(90)-H(90C)	0.9800

C(81)-C(82)	1.546(13)
C(81)-H(81A)	0.9900
C(81)-H(81B)	0.9900
C(82)-H(82A)	0.9900
C(82)-H(82B)	0.9900
Li(8)-I(1)#2	2.860(11)

Li(2)-I(1)-Li(1)	79.2(3)
Li(2)-I(1)-Li(8)#1	149.9(3)
Li(1)-I(1)-Li(8)#1	126.7(3)
Li(2)-I(2)-Li(1)	79.9(4)
Li(2)-I(2)-Li(3)	137.4(4)
Li(1)-I(2)-Li(3)	142.7(3)
Li(6)-I(3)-Li(5)	79.5(4)
Li(6)-I(3)-Li(4)	133.4(3)
Li(5)-I(3)-Li(4)	145.3(4)
Li(5)-I(4)-Li(6)	80.1(4)
Li(5)-I(4)-Li(7)	141.6(4)
Li(6)-I(4)-Li(7)	137.7(4)
N(4)-Si(5)-C(15)	112.8(3)
N(4)-Si(5)-C(14)	111.0(3)
C(15)-Si(5)-C(14)	107.1(4)
N(4)-Si(5)-C(13)	115.9(3)
C(15)-Si(5)-C(13)	106.7(4)
C(14)-Si(5)-C(13)	102.5(4)
C(15)-Si(5)-Li(8)	133.9(4)
C(14)-Si(5)-Li(8)	69.1(3)
C(13)-Si(5)-Li(8)	119.1(4)
C(15)-Si(5)-Li(7)	89.2(4)
C(14)-Si(5)-Li(7)	96.0(4)
C(13)-Si(5)-Li(7)	150.6(3)
Li(8)-Si(5)-Li(7)	47.8(3)
N(2)-Si(6)-C(1)	112.2(3)
N(2)-Si(6)-C(2)	115.9(3)
C(1)-Si(6)-C(2)	106.4(4)
N(2)-Si(6)-C(3)	110.9(3)
C(1)-Si(6)-C(3)	107.2(4)
C(2)-Si(6)-C(3)	103.5(4)
C(1)-Si(6)-Li(4)	131.1(4)
C(2)-Si(6)-Li(4)	121.9(4)

C(3)-Si(6)-Li(4)	70.2(3)
C(1)-Si(6)-Li(3)	87.5(3)
C(2)-Si(6)-Li(3)	149.3(3)
C(3)-Si(6)-Li(3)	97.9(4)
Li(4)-Si(6)-Li(3)	46.9(3)
N(4)-Si(7)-C(18)	112.3(3)
N(4)-Si(7)-C(17)	111.3(3)
C(18)-Si(7)-C(17)	106.6(4)
N(4)-Si(7)-C(16)	113.7(3)
C(18)-Si(7)-C(16)	106.9(4)
C(17)-Si(7)-C(16)	105.5(4)
C(18)-Si(7)-Li(7)	134.0(3)
C(17)-Si(7)-Li(7)	73.6(3)
C(16)-Si(7)-Li(7)	117.4(3)
N(3)-Si(8)-C(20)	112.6(3)
N(3)-Si(8)-C(21)	110.6(3)
C(20)-Si(8)-C(21)	106.4(3)
N(3)-Si(8)-C(19)	114.5(3)
C(20)-Si(8)-C(19)	107.1(3)
C(21)-Si(8)-C(19)	105.0(3)
C(20)-Si(8)-Li(8)	131.8(3)
C(21)-Si(8)-Li(8)	68.5(3)
C(19)-Si(8)-Li(8)	120.7(3)
C(20)-Si(8)-Li(7)	87.7(3)
C(21)-Si(8)-Li(7)	96.5(3)
C(19)-Si(8)-Li(7)	148.8(3)
Li(8)-Si(8)-Li(7)	47.9(3)
N(2)-Si(9)-C(5)	110.1(3)
N(2)-Si(9)-C(6)	112.6(3)
C(5)-Si(9)-C(6)	107.1(4)
N(2)-Si(9)-C(4)	114.8(3)
C(5)-Si(9)-C(4)	104.7(3)
C(6)-Si(9)-C(4)	106.9(4)
C(5)-Si(9)-Li(3)	70.3(3)
C(6)-Si(9)-Li(3)	135.6(3)
C(4)-Si(9)-Li(3)	116.7(3)
N(3)-Si(10)-C(24)	112.5(3)
N(3)-Si(10)-C(22)	112.2(3)
C(24)-Si(10)-C(22)	106.3(4)
N(3)-Si(10)-C(23)	115.2(3)

C(24)-Si(10)-C(23)	103.6(4)
C(22)-Si(10)-C(23)	106.2(4)
C(24)-Si(10)-Li(7)	73.5(3)
C(22)-Si(10)-Li(7)	137.6(4)
C(23)-Si(10)-Li(7)	115.1(4)
N(1)-Si(11)-C(7)	114.9(3)
N(1)-Si(11)-C(8)	112.7(3)
C(7)-Si(11)-C(8)	103.6(3)
N(1)-Si(11)-C(9)	111.6(3)
C(7)-Si(11)-C(9)	106.9(4)
C(8)-Si(11)-C(9)	106.3(4)
C(7)-Si(11)-Li(4)	121.7(3)
C(8)-Si(11)-Li(4)	72.6(3)
C(9)-Si(11)-Li(4)	130.5(4)
N(1)-Si(12)-C(12)	112.7(3)
N(1)-Si(12)-C(11)	112.1(3)
C(12)-Si(12)-C(11)	105.8(4)
N(1)-Si(12)-C(10)	115.6(3)
C(12)-Si(12)-C(10)	107.3(4)
C(11)-Si(12)-C(10)	102.3(4)
C(12)-Si(12)-Li(3)	136.2(4)
C(11)-Si(12)-Li(3)	72.6(3)
C(10)-Si(12)-Li(3)	115.9(4)
Si(5)-N(4)-Si(7)	122.3(3)
Si(5)-N(4)-Li(7)	116.3(4)
Si(7)-N(4)-Li(7)	109.4(4)
Si(5)-N(4)-Li(8)	103.0(4)
Si(7)-N(4)-Li(8)	121.6(4)
Li(7)-N(4)-Li(8)	75.5(5)
Si(8)-N(3)-Si(10)	123.1(3)
Si(8)-N(3)-Li(7)	116.3(4)
Si(10)-N(3)-Li(7)	105.4(4)
Si(8)-N(3)-Li(8)	103.4(4)
Si(10)-N(3)-Li(8)	124.2(4)
Li(7)-N(3)-Li(8)	75.2(5)
Si(12)-N(1)-Si(11)	122.4(3)
Si(12)-N(1)-Li(3)	105.6(4)
Si(11)-N(1)-Li(3)	118.1(4)
Si(12)-N(1)-Li(4)	120.3(4)
Si(11)-N(1)-Li(4)	106.6(4)

Li(3)-N(1)-Li(4)	74.8(5)
Si(6)-N(2)-Si(9)	124.0(3)
Si(6)-N(2)-Li(4)	105.7(4)
Si(9)-N(2)-Li(4)	119.6(4)
Si(6)-N(2)-Li(3)	117.5(4)
Si(9)-N(2)-Li(3)	105.4(4)
Li(4)-N(2)-Li(3)	74.7(5)
C(97)-N(99)-C(98)	108.8(6)
C(97)-N(99)-C(92)	112.5(5)
C(98)-N(99)-C(92)	113.7(6)
C(97)-N(99)-Li(1)	112.7(6)
C(98)-N(99)-Li(1)	104.0(5)
C(92)-N(99)-Li(1)	104.9(5)
C(89)-N(97)-C(88)	111.8(6)
C(89)-N(97)-C(84)	108.2(6)
C(88)-N(97)-C(84)	113.3(5)
C(89)-N(97)-Li(6)	112.0(6)
C(88)-N(97)-Li(6)	105.8(6)
C(84)-N(97)-Li(6)	105.7(6)
C(85)-N(96)-C(86)	109.4(6)
C(85)-N(96)-C(87)	111.6(6)
C(86)-N(96)-C(87)	113.5(6)
C(85)-N(96)-Li(6)	111.9(6)
C(86)-N(96)-Li(6)	105.9(5)
C(87)-N(96)-Li(6)	104.3(5)
C(78)-N(94)-C(79)	108.5(6)
C(78)-N(94)-C(77)	113.7(6)
C(79)-N(94)-C(77)	111.8(6)
C(78)-N(94)-Li(2)	104.7(6)
C(79)-N(94)-Li(2)	113.0(6)
C(77)-N(94)-Li(2)	105.0(5)
C(99)-N(98)-C(90)	109.7(6)
C(99)-N(98)-C(96)	109.8(6)
C(90)-N(98)-C(96)	113.5(5)
C(99)-N(98)-Li(1)	112.5(6)
C(90)-N(98)-Li(1)	105.8(6)
C(96)-N(98)-Li(1)	105.5(5)
Si(7)-C(16)-H(16A)	109.5
Si(7)-C(16)-H(16B)	109.5
H(16A)-C(16)-H(16B)	109.5

Si(7)-C(16)-H(16C)	109.5
H(16A)-C(16)-H(16C)	109.5
H(16B)-C(16)-H(16C)	109.5
Si(8)-C(19)-H(19A)	109.5
Si(8)-C(19)-H(19B)	109.5
H(19A)-C(19)-H(19B)	109.5
Si(8)-C(19)-H(19C)	109.5
H(19A)-C(19)-H(19C)	109.5
H(19B)-C(19)-H(19C)	109.5
N(97)-C(88)-C(83)	114.7(6)
N(97)-C(88)-C(87)	110.5(6)
C(83)-C(88)-C(87)	110.7(6)
N(97)-C(88)-H(88A)	106.8
C(83)-C(88)-H(88A)	106.8
C(87)-C(88)-H(88A)	106.8
Si(9)-C(4)-H(4A)	109.5
Si(9)-C(4)-H(4B)	109.5
H(4A)-C(4)-H(4B)	109.5
Si(9)-C(4)-H(4C)	109.5
H(4A)-C(4)-H(4C)	109.5
H(4B)-C(4)-H(4C)	109.5
C(74)-N(95)-C(75)	109.1(6)
C(74)-N(95)-C(76)	112.8(5)
C(75)-N(95)-C(76)	110.9(6)
C(74)-N(95)-Li(2)	106.9(6)
C(75)-N(95)-Li(2)	112.0(5)
C(76)-N(95)-Li(2)	105.0(5)
N(99)-C(98)-H(98A)	109.5
N(99)-C(98)-H(98B)	109.5
H(98A)-C(98)-H(98B)	109.5
N(99)-C(98)-H(98C)	109.5
H(98A)-C(98)-H(98C)	109.5
H(98B)-C(98)-H(98C)	109.5
Si(8)-C(20)-H(20A)	109.5
Si(8)-C(20)-H(20B)	109.5
H(20A)-C(20)-H(20B)	109.5
Si(8)-C(20)-H(20C)	109.5
H(20A)-C(20)-H(20C)	109.5
H(20B)-C(20)-H(20C)	109.5
Si(9)-C(5)-H(5A)	109.5

Si(9)-C(5)-H(5B)	109.5
H(5A)-C(5)-H(5B)	109.5
Si(9)-C(5)-H(5C)	109.5
H(5A)-C(5)-H(5C)	109.5
H(5B)-C(5)-H(5C)	109.5
Si(11)-C(7)-H(7A)	109.5
Si(11)-C(7)-H(7B)	109.5
H(7A)-C(7)-H(7B)	109.5
Si(11)-C(7)-H(7C)	109.5
H(7A)-C(7)-H(7C)	109.5
H(7B)-C(7)-H(7C)	109.5
Si(10)-C(22)-H(22A)	109.5
Si(10)-C(22)-H(22B)	109.5
H(22A)-C(22)-H(22B)	109.5
Si(10)-C(22)-H(22C)	109.5
H(22A)-C(22)-H(22C)	109.5
H(22B)-C(22)-H(22C)	109.5
N(98)-C(99)-H(99A)	109.5
N(98)-C(99)-H(99B)	109.5
H(99A)-C(99)-H(99B)	109.5
N(98)-C(99)-H(99C)	109.5
H(99A)-C(99)-H(99C)	109.5
H(99B)-C(99)-H(99C)	109.5
N(94)-C(78)-H(78A)	109.5
N(94)-C(78)-H(78B)	109.5
H(78A)-C(78)-H(78B)	109.5
N(94)-C(78)-H(78C)	109.5
H(78A)-C(78)-H(78C)	109.5
H(78B)-C(78)-H(78C)	109.5
N(99)-C(92)-C(96)	111.4(5)
N(99)-C(92)-C(91)	113.7(6)
C(96)-C(92)-C(91)	110.1(6)
N(99)-C(92)-H(92A)	107.1
C(96)-C(92)-H(92A)	107.1
C(91)-C(92)-H(92A)	107.1
Si(11)-C(8)-H(8A)	109.5
Si(11)-C(8)-H(8B)	109.5
H(8A)-C(8)-H(8B)	109.5
Si(11)-C(8)-H(8C)	109.5
H(8A)-C(8)-H(8C)	109.5

H(8B)-C(8)-H(8C)	109.5
Si(5)-C(13)-H(13A)	109.5
Si(5)-C(13)-H(13B)	109.5
H(13A)-C(13)-H(13B)	109.5
Si(5)-C(13)-H(13C)	109.5
H(13A)-C(13)-H(13C)	109.5
H(13B)-C(13)-H(13C)	109.5
Si(6)-C(1)-H(1A)	109.5
Si(6)-C(1)-H(1B)	109.5
H(1A)-C(1)-H(1B)	109.5
Si(6)-C(1)-H(1C)	109.5
H(1A)-C(1)-H(1C)	109.5
H(1B)-C(1)-H(1C)	109.5
Si(5)-C(14)-H(14A)	109.5
Si(5)-C(14)-H(14B)	109.5
H(14A)-C(14)-H(14B)	109.5
Si(5)-C(14)-H(14C)	109.5
H(14A)-C(14)-H(14C)	109.5
H(14B)-C(14)-H(14C)	109.5
N(97)-C(89)-H(89A)	109.5
N(97)-C(89)-H(89B)	109.5
H(89A)-C(89)-H(89B)	109.5
N(97)-C(89)-H(89C)	109.5
H(89A)-C(89)-H(89C)	109.5
H(89B)-C(89)-H(89C)	109.5
N(95)-C(74)-H(74A)	109.5
N(95)-C(74)-H(74B)	109.5
H(74A)-C(74)-H(74B)	109.5
N(95)-C(74)-H(74C)	109.5
H(74A)-C(74)-H(74C)	109.5
H(74B)-C(74)-H(74C)	109.5
N(96)-C(86)-H(86A)	109.5
N(96)-C(86)-H(86B)	109.5
H(86A)-C(86)-H(86B)	109.5
N(96)-C(86)-H(86C)	109.5
H(86A)-C(86)-H(86C)	109.5
H(86B)-C(86)-H(86C)	109.5
Si(8)-C(21)-H(21A)	109.5
Si(8)-C(21)-H(21B)	109.5
H(21A)-C(21)-H(21B)	109.5

Si(8)-C(21)-H(21C)	109.5
H(21A)-C(21)-H(21C)	109.5
H(21B)-C(21)-H(21C)	109.5
Si(10)-C(23)-H(23A)	109.5
Si(10)-C(23)-H(23B)	109.5
H(23A)-C(23)-H(23B)	109.5
Si(10)-C(23)-H(23C)	109.5
H(23A)-C(23)-H(23C)	109.5
H(23B)-C(23)-H(23C)	109.5
Si(11)-C(9)-H(9A)	109.5
Si(11)-C(9)-H(9B)	109.5
H(9A)-C(9)-H(9B)	109.5
Si(11)-C(9)-H(9C)	109.5
H(9A)-C(9)-H(9C)	109.5
H(9B)-C(9)-H(9C)	109.5
N(97)-C(84)-H(84A)	109.5
N(97)-C(84)-H(84B)	109.5
H(84A)-C(84)-H(84B)	109.5
N(97)-C(84)-H(84C)	109.5
H(84A)-C(84)-H(84C)	109.5
H(84B)-C(84)-H(84C)	109.5
Si(7)-C(17)-H(17A)	109.5
Si(7)-C(17)-H(17B)	109.5
H(17A)-C(17)-H(17B)	109.5
Si(7)-C(17)-H(17C)	109.5
H(17A)-C(17)-H(17C)	109.5
H(17B)-C(17)-H(17C)	109.5
N(94)-C(77)-C(76)	111.4(6)
N(94)-C(77)-C(70)	114.8(7)
C(76)-C(77)-C(70)	110.4(7)
N(94)-C(77)-H(77A)	106.6
C(76)-C(77)-H(77A)	106.6
C(70)-C(77)-H(77A)	106.6
N(98)-C(96)-C(95)	114.4(6)
N(98)-C(96)-C(92)	110.7(6)
C(95)-C(96)-C(92)	111.2(6)
N(98)-C(96)-H(96A)	106.7
C(95)-C(96)-H(96A)	106.7
C(92)-C(96)-H(96A)	106.7
Si(6)-C(2)-H(2A)	109.5

Si(6)-C(2)-H(2B)	109.5
H(2A)-C(2)-H(2B)	109.5
Si(6)-C(2)-H(2C)	109.5
H(2A)-C(2)-H(2C)	109.5
H(2B)-C(2)-H(2C)	109.5
Si(12)-C(10)-H(10A)	109.5
Si(12)-C(10)-H(10B)	109.5
H(10A)-C(10)-H(10B)	109.5
Si(12)-C(10)-H(10C)	109.5
H(10A)-C(10)-H(10C)	109.5
H(10B)-C(10)-H(10C)	109.5
Si(6)-C(3)-H(3A)	109.5
Si(6)-C(3)-H(3B)	109.5
H(3A)-C(3)-H(3B)	109.5
Si(6)-C(3)-H(3C)	109.5
H(3A)-C(3)-H(3C)	109.5
H(3B)-C(3)-H(3C)	109.5
N(99)-C(97)-H(97A)	109.5
N(99)-C(97)-H(97B)	109.5
H(97A)-C(97)-H(97B)	109.5
N(99)-C(97)-H(97C)	109.5
H(97A)-C(97)-H(97C)	109.5
H(97B)-C(97)-H(97C)	109.5
Si(5)-C(15)-H(15A)	109.5
Si(5)-C(15)-H(15B)	109.5
H(15A)-C(15)-H(15B)	109.5
Si(5)-C(15)-H(15C)	109.5
H(15A)-C(15)-H(15C)	109.5
H(15B)-C(15)-H(15C)	109.5
C(94)-C(93)-C(91)	110.1(6)
C(94)-C(93)-H(93A)	109.6
C(91)-C(93)-H(93A)	109.7
C(94)-C(93)-H(93B)	109.7
C(91)-C(93)-H(93B)	109.7
H(93A)-C(93)-H(93B)	108.2
Si(9)-C(6)-H(6A)	109.5
Si(9)-C(6)-H(6B)	109.5
H(6A)-C(6)-H(6B)	109.5
Si(9)-C(6)-H(6C)	109.5
H(6A)-C(6)-H(6C)	109.5

H(6B)-C(6)-H(6C)	109.5
Si(10)-C(24)-H(24A)	109.5
Si(10)-C(24)-H(24B)	109.5
H(24A)-C(24)-H(24B)	109.5
Si(10)-C(24)-H(24C)	109.5
H(24A)-C(24)-H(24C)	109.5
H(24B)-C(24)-H(24C)	109.5
Si(12)-C(11)-H(11A)	109.5
Si(12)-C(11)-H(11B)	109.5
H(11A)-C(11)-H(11B)	109.5
Si(12)-C(11)-H(11C)	109.5
H(11A)-C(11)-H(11C)	109.5
H(11B)-C(11)-H(11C)	109.5
C(96)-C(95)-C(94)	111.4(7)
C(96)-C(95)-H(95A)	109.4
C(94)-C(95)-H(95A)	109.4
C(96)-C(95)-H(95B)	109.4
C(94)-C(95)-H(95B)	109.4
H(95A)-C(95)-H(95B)	108.0
N(96)-C(87)-C(88)	111.5(6)
N(96)-C(87)-C(80)	113.6(6)
C(88)-C(87)-C(80)	111.0(6)
N(96)-C(87)-H(87A)	106.8
C(88)-C(87)-H(87A)	106.8
C(80)-C(87)-H(87A)	106.8
Si(7)-C(18)-H(18A)	109.5
Si(7)-C(18)-H(18B)	109.5
H(18A)-C(18)-H(18B)	109.5
Si(7)-C(18)-H(18C)	109.5
H(18A)-C(18)-H(18C)	109.5
H(18B)-C(18)-H(18C)	109.5
Si(12)-C(12)-H(12A)	109.5
Si(12)-C(12)-H(12B)	109.5
H(12A)-C(12)-H(12B)	109.5
Si(12)-C(12)-H(12C)	109.5
H(12A)-C(12)-H(12C)	109.5
H(12B)-C(12)-H(12C)	109.5
C(93)-C(94)-C(95)	109.7(7)
C(93)-C(94)-H(94A)	109.7
C(95)-C(94)-H(94A)	109.7

C(93)-C(94)-H(94B)	109.7
C(95)-C(94)-H(94B)	109.7
H(94A)-C(94)-H(94B)	108.2
N(95)-C(76)-C(77)	110.5(6)
N(95)-C(76)-C(73)	114.2(6)
C(77)-C(76)-C(73)	112.2(6)
N(95)-C(76)-H(76A)	106.4
C(77)-C(76)-H(76A)	106.4
C(73)-C(76)-H(76A)	106.4
N(96)-C(85)-H(85A)	109.5
N(96)-C(85)-H(85B)	109.5
H(85A)-C(85)-H(85B)	109.5
N(96)-C(85)-H(85C)	109.5
H(85A)-C(85)-H(85C)	109.5
H(85B)-C(85)-H(85C)	109.5
N(2)-Li(4)-N(1)	105.1(6)
N(2)-Li(4)-Li(3)	52.7(4)
N(1)-Li(4)-Li(3)	52.4(4)
N(2)-Li(4)-I(3)	125.6(6)
N(1)-Li(4)-I(3)	129.1(6)
Li(3)-Li(4)-I(3)	175.5(5)
N(2)-Li(4)-Si(6)	33.3(2)
N(1)-Li(4)-Si(6)	115.6(5)
Li(3)-Li(4)-Si(6)	71.1(4)
I(3)-Li(4)-Si(6)	109.7(4)
N(2)-Li(4)-Si(11)	116.9(5)
N(1)-Li(4)-Si(11)	32.9(2)
Li(3)-Li(4)-Si(11)	70.9(4)
I(3)-Li(4)-Si(11)	108.3(4)
Si(6)-Li(4)-Si(11)	142.0(4)
N(94)-C(79)-H(79A)	109.5
N(94)-C(79)-H(79B)	109.5
H(79A)-C(79)-H(79B)	109.5
N(94)-C(79)-H(79C)	109.5
H(79A)-C(79)-H(79C)	109.5
H(79B)-C(79)-H(79C)	109.5
N(95)-C(75)-H(75A)	109.5
N(95)-C(75)-H(75B)	109.5
H(75A)-C(75)-H(75B)	109.5
N(95)-C(75)-H(75C)	109.5

H(75A)-C(75)-H(75C)	109.5
H(75B)-C(75)-H(75C)	109.5
C(63)-N(92)-C(60)	111.7(6)
C(63)-N(92)-C(69)	108.7(6)
C(60)-N(92)-C(69)	112.5(6)
C(63)-N(92)-Li(5)	113.0(6)
C(60)-N(92)-Li(5)	104.9(6)
C(69)-N(92)-Li(5)	105.9(5)
C(67)-N(93)-C(66)	107.7(6)
C(67)-N(93)-C(62)	111.1(6)
C(66)-N(93)-C(62)	113.9(6)
C(67)-N(93)-Li(5)	113.4(6)
C(66)-N(93)-Li(5)	105.0(6)
C(62)-N(93)-Li(5)	105.8(5)
N(93)-C(67)-H(67A)	109.5
N(93)-C(67)-H(67B)	109.5
H(67A)-C(67)-H(67B)	109.5
N(93)-C(67)-H(67C)	109.5
H(67A)-C(67)-H(67C)	109.5
H(67B)-C(67)-H(67C)	109.5
N(93)-C(62)-C(60)	112.2(6)
N(93)-C(62)-C(61)	113.6(6)
C(60)-C(62)-C(61)	111.3(6)
N(93)-C(62)-H(62A)	106.4
C(60)-C(62)-H(62A)	106.4
C(61)-C(62)-H(62A)	106.4
C(92)-C(91)-C(93)	111.5(6)
C(92)-C(91)-H(91A)	109.3
C(93)-C(91)-H(91A)	109.3
C(92)-C(91)-H(91B)	109.3
C(93)-C(91)-H(91B)	109.3
H(91A)-C(91)-H(91B)	108.0
C(71)-C(72)-C(73)	109.5(8)
C(71)-C(72)-H(72A)	109.8
C(73)-C(72)-H(72A)	109.8
C(71)-C(72)-H(72B)	109.8
C(73)-C(72)-H(72B)	109.8
H(72A)-C(72)-H(72B)	108.2
N(93)-C(66)-H(66A)	109.5
N(93)-C(66)-H(66B)	109.5

H(66A)-C(66)-H(66B)	109.5
N(93)-C(66)-H(66C)	109.5
H(66A)-C(66)-H(66C)	109.5
H(66B)-C(66)-H(66C)	109.5
C(81)-C(80)-C(87)	112.3(8)
C(81)-C(80)-H(80A)	109.1
C(87)-C(80)-H(80A)	109.1
C(81)-C(80)-H(80B)	109.1
C(87)-C(80)-H(80B)	109.1
H(80A)-C(80)-H(80B)	107.9
N(92)-C(69)-H(69A)	109.5
N(92)-C(69)-H(69B)	109.5
H(69A)-C(69)-H(69B)	109.5
N(92)-C(69)-H(69C)	109.5
H(69A)-C(69)-H(69C)	109.5
H(69B)-C(69)-H(69C)	109.5
C(72)-C(73)-C(76)	111.3(7)
C(72)-C(73)-H(73A)	109.4
C(76)-C(73)-H(73A)	109.4
C(72)-C(73)-H(73B)	109.4
C(76)-C(73)-H(73B)	109.4
H(73A)-C(73)-H(73B)	108.0
C(82)-C(83)-C(88)	111.8(7)
C(82)-C(83)-H(83A)	109.3
C(88)-C(83)-H(83A)	109.3
C(82)-C(83)-H(83B)	109.3
C(88)-C(83)-H(83B)	109.3
H(83A)-C(83)-H(83B)	107.9
C(65)-C(61)-C(62)	112.6(7)
C(65)-C(61)-H(61A)	109.1
C(62)-C(61)-H(61A)	109.1
C(65)-C(61)-H(61B)	109.1
C(62)-C(61)-H(61B)	109.1
H(61A)-C(61)-H(61B)	107.8
C(64)-C(68)-C(60)	111.6(7)
C(64)-C(68)-H(68A)	109.3
C(60)-C(68)-H(68A)	109.3
C(64)-C(68)-H(68B)	109.3
C(60)-C(68)-H(68B)	109.3
H(68A)-C(68)-H(68B)	108.0

N(4)-Li(7)-N(3)	105.3(6)
N(4)-Li(7)-Li(8)	52.5(5)
N(3)-Li(7)-Li(8)	52.8(4)
N(4)-Li(7)-I(4)	129.0(6)
N(3)-Li(7)-I(4)	125.7(6)
Li(8)-Li(7)-I(4)	177.9(6)
N(4)-Li(7)-Si(10)	119.6(5)
N(3)-Li(7)-Si(10)	33.7(3)
Li(8)-Li(7)-Si(10)	74.4(4)
I(4)-Li(7)-Si(10)	105.0(4)
N(4)-Li(7)-Si(7)	31.9(3)
N(3)-Li(7)-Si(7)	118.5(5)
Li(8)-Li(7)-Si(7)	71.6(4)
I(4)-Li(7)-Si(7)	109.0(4)
Si(10)-Li(7)-Si(7)	146.0(5)
N(4)-Li(7)-Si(8)	107.7(5)
N(3)-Li(7)-Si(8)	28.7(2)
Li(8)-Li(7)-Si(8)	61.4(4)
I(4)-Li(7)-Si(8)	116.5(5)
Si(10)-Li(7)-Si(8)	58.3(2)
Si(7)-Li(7)-Si(8)	105.0(4)
N(4)-Li(7)-Si(5)	28.8(2)
N(3)-Li(7)-Si(5)	106.4(5)
Li(8)-Li(7)-Si(5)	61.1(4)
I(4)-Li(7)-Si(5)	121.0(4)
Si(10)-Li(7)-Si(5)	104.1(4)
Si(7)-Li(7)-Si(5)	57.4(3)
Si(8)-Li(7)-Si(5)	122.5(4)
N(1)-Li(3)-N(2)	105.4(6)
N(1)-Li(3)-Li(4)	52.8(4)
N(2)-Li(3)-Li(4)	52.6(4)
N(1)-Li(3)-I(2)	126.3(6)
N(2)-Li(3)-I(2)	128.2(5)
Li(4)-Li(3)-I(2)	178.5(6)
N(1)-Li(3)-Si(12)	33.4(2)
N(2)-Li(3)-Si(12)	117.5(5)
Li(4)-Li(3)-Si(12)	72.6(4)
I(2)-Li(3)-Si(12)	106.1(4)
N(1)-Li(3)-Si(9)	118.2(5)
N(2)-Li(3)-Si(9)	33.4(2)

Li(4)-Li(3)-Si(9)	72.1(4)
I(2)-Li(3)-Si(9)	109.2(4)
Si(12)-Li(3)-Si(9)	144.7(5)
N(1)-Li(3)-Si(6)	107.8(5)
N(2)-Li(3)-Si(6)	28.1(2)
Li(4)-Li(3)-Si(6)	62.0(4)
I(2)-Li(3)-Si(6)	118.1(4)
Si(12)-Li(3)-Si(6)	103.5(3)
Si(9)-Li(3)-Si(6)	58.1(2)
N(92)-C(63)-H(63A)	109.5
N(92)-C(63)-H(63B)	109.5
H(63A)-C(63)-H(63B)	109.5
N(92)-C(63)-H(63C)	109.5
H(63A)-C(63)-H(63C)	109.5
H(63B)-C(63)-H(63C)	109.5
N(92)-C(60)-C(62)	111.5(6)
N(92)-C(60)-C(68)	115.8(6)
C(62)-C(60)-C(68)	109.8(6)
N(92)-C(60)-H(60A)	106.4
C(62)-C(60)-H(60A)	106.4
C(68)-C(60)-H(60A)	106.4
N(95)-Li(2)-N(94)	87.7(5)
N(95)-Li(2)-I(2)	113.0(5)
N(94)-Li(2)-I(2)	122.8(6)
N(95)-Li(2)-I(1)	120.7(6)
N(94)-Li(2)-I(1)	113.8(5)
I(2)-Li(2)-I(1)	100.4(4)
N(95)-Li(2)-Li(1)	138.1(6)
N(94)-Li(2)-Li(1)	134.2(5)
I(2)-Li(2)-Li(1)	50.1(3)
I(1)-Li(2)-Li(1)	50.4(3)
C(65)-C(64)-C(68)	111.3(7)
C(65)-C(64)-H(64A)	109.4
C(68)-C(64)-H(64A)	109.4
C(65)-C(64)-H(64B)	109.4
C(68)-C(64)-H(64B)	109.4
H(64A)-C(64)-H(64B)	108.0
C(72)-C(71)-C(70)	110.5(8)
C(72)-C(71)-H(71A)	109.6
C(70)-C(71)-H(71A)	109.6

C(72)-C(71)-H(71B)	109.6
C(70)-C(71)-H(71B)	109.6
H(71A)-C(71)-H(71B)	108.1
N(92)-Li(5)-N(93)	87.6(6)
N(92)-Li(5)-I(4)	122.0(5)
N(93)-Li(5)-I(4)	118.0(6)
N(92)-Li(5)-I(3)	112.6(6)
N(93)-Li(5)-I(3)	117.3(5)
I(4)-Li(5)-I(3)	100.4(4)
N(92)-Li(5)-Li(6)	139.1(7)
N(93)-Li(5)-Li(6)	132.9(5)
I(4)-Li(5)-Li(6)	50.5(3)
I(3)-Li(5)-Li(6)	50.1(3)
C(64)-C(65)-C(61)	110.2(7)
C(64)-C(65)-H(65A)	109.6
C(61)-C(65)-H(65A)	109.6
C(64)-C(65)-H(65B)	109.6
C(61)-C(65)-H(65B)	109.6
H(65A)-C(65)-H(65B)	108.1
C(71)-C(70)-C(77)	112.9(7)
C(71)-C(70)-H(70A)	109.0
C(77)-C(70)-H(70A)	109.0
C(71)-C(70)-H(70B)	109.0
C(77)-C(70)-H(70B)	109.0
H(70A)-C(70)-H(70B)	107.8
N(98)-C(90)-H(90A)	109.5
N(98)-C(90)-H(90B)	109.5
H(90A)-C(90)-H(90B)	109.5
N(98)-C(90)-H(90C)	109.5
H(90A)-C(90)-H(90C)	109.5
H(90B)-C(90)-H(90C)	109.5
C(80)-C(81)-C(82)	109.2(8)
C(80)-C(81)-H(81A)	109.8
C(82)-C(81)-H(81A)	109.8
C(80)-C(81)-H(81B)	109.8
C(82)-C(81)-H(81B)	109.8
H(81A)-C(81)-H(81B)	108.3
N(98)-Li(1)-N(99)	88.4(5)
N(98)-Li(1)-I(2)	124.2(5)
N(99)-Li(1)-I(2)	111.3(5)

N(98)-Li(1)-I(1)	113.9(5)
N(99)-Li(1)-I(1)	120.2(5)
I(2)-Li(1)-I(1)	100.3(4)
N(98)-Li(1)-Li(2)	142.4(5)
N(99)-Li(1)-Li(2)	129.2(6)
I(2)-Li(1)-Li(2)	50.0(3)
I(1)-Li(1)-Li(2)	50.4(3)
N(96)-Li(6)-N(97)	88.7(5)
N(96)-Li(6)-I(3)	117.4(6)
N(97)-Li(6)-I(3)	115.1(5)
N(96)-Li(6)-I(4)	118.2(5)
N(97)-Li(6)-I(4)	119.2(6)
I(3)-Li(6)-I(4)	99.6(4)
N(96)-Li(6)-Li(5)	139.8(5)
N(97)-Li(6)-Li(5)	131.5(6)
I(3)-Li(6)-Li(5)	50.4(3)
I(4)-Li(6)-Li(5)	49.4(3)
C(83)-C(82)-C(81)	111.3(8)
C(83)-C(82)-H(82A)	109.4
C(81)-C(82)-H(82A)	109.4
C(83)-C(82)-H(82B)	109.4
C(81)-C(82)-H(82B)	109.4
H(82A)-C(82)-H(82B)	108.0
N(4)-Li(8)-N(3)	104.0(5)
N(4)-Li(8)-Li(7)	52.0(4)
N(3)-Li(8)-Li(7)	52.0(4)
N(4)-Li(8)-I(1)#2	126.1(6)
N(3)-Li(8)-I(1)#2	129.9(5)
Li(7)-Li(8)-I(1)#2	177.3(6)
N(4)-Li(8)-Si(5)	34.5(2)
N(3)-Li(8)-Si(5)	114.5(5)
Li(7)-Li(8)-Si(5)	71.1(4)
I(1)#2-Li(8)-Si(5)	108.5(4)
N(4)-Li(8)-Si(8)	115.5(5)
N(3)-Li(8)-Si(8)	34.0(3)
Li(7)-Li(8)-Si(8)	70.7(4)
I(1)#2-Li(8)-Si(8)	109.8(4)
Si(5)-Li(8)-Si(8)	141.7(4)

Symmetry transformations used to generate equivalent atoms:

#1 x-1,y-1,z   #2 x+1,y+1,z

Table 4. Anisotropic displacement parameters ($Å^2$x 10^3) for srgr1101. The anisotropic displacement factor exponent takes the form: $-2\pi^2[h^2 a^{*2}U^{11} + ... + 2 h k a^* b^* U^{12}]$

	U^{11}	U^{22}	U^{33}	U^{23}	U^{13}	U^{12}
I(1)	48(1)	39(1)	42(1)	-14(1)	14(1)	-24(1)
I(2)	50(1)	41(1)	51(1)	-19(1)	20(1)	-27(1)
I(3)	47(1)	35(1)	60(1)	15(1)	-19(1)	-23(1)
I(4)	47(1)	30(1)	58(1)	11(1)	-16(1)	-19(1)
Si(5)	34(1)	26(1)	24(1)	1(1)	-6(1)	1(1)
Si(6)	23(1)	26(1)	30(1)	-3(1)	5(1)	-1(1)
Si(7)	25(1)	28(1)	26(1)	-2(1)	-1(1)	1(1)
Si(8)	22(1)	25(1)	25(1)	1(1)	-3(1)	1(1)
Si(9)	22(1)	24(1)	30(1)	2(1)	-2(1)	1(1)
Si(10)	28(1)	25(1)	26(1)	-2(1)	2(1)	-4(1)
Si(11)	19(1)	34(1)	29(1)	-10(1)	0(1)	2(1)
Si(12)	30(1)	28(1)	29(1)	-5(1)	-7(1)	4(1)
N(4)	23(4)	26(3)	22(2)	-2(2)	-2(2)	-4(3)
N(3)	14(3)	19(3)	28(3)	7(2)	2(2)	-3(2)
N(1)	18(3)	22(3)	29(3)	-7(2)	4(2)	-7(3)
N(2)	20(3)	17(3)	22(2)	0(2)	2(2)	-2(3)
N(99)	25(4)	32(3)	27(3)	-6(2)	-5(3)	4(3)
N(97)	28(4)	25(3)	34(3)	-5(2)	3(3)	-11(3)
N(96)	33(4)	23(3)	36(3)	-5(3)	5(3)	-7(3)
N(94)	36(4)	36(4)	31(3)	4(3)	-9(3)	-5(3)
N(98)	30(4)	33(3)	30(3)	3(3)	-5(3)	-8(3)
C(16)	56(6)	40(4)	33(3)	-8(3)	-2(4)	-2(5)
C(19)	34(5)	25(4)	40(3)	6(3)	-14(3)	-3(4)
C(88)	34(5)	31(4)	29(3)	-1(3)	-3(3)	-14(4)
C(4)	40(5)	37(4)	45(4)	5(4)	3(4)	13(4)
N(95)	25(4)	33(3)	28(3)	-6(2)	-9(3)	2(3)
C(98)	51(6)	31(4)	44(4)	0(3)	-7(4)	6(4)
C(20)	32(5)	41(4)	43(4)	1(3)	-18(4)	-7(4)
C(5)	38(5)	34(4)	31(4)	4(3)	0(3)	1(4)
C(7)	40(6)	52(5)	41(4)	-17(4)	-8(4)	16(5)
C(22)	32(5)	38(4)	45(4)	-7(4)	4(4)	-10(4)
C(99)	32(5)	64(6)	43(4)	-5(4)	-17(4)	-9(4)
C(78)	39(5)	69(6)	41(4)	-10(4)	-8(4)	-3(5)
C(92)	27(4)	19(3)	33(3)	-7(3)	1(3)	-7(3)
C(8)	27(5)	49(5)	33(4)	-10(4)	-9(3)	0(4)

C(13)	28(5)	65(6)	50(4)	4(4)	-12(4)	-2(5)
C(1)	29(5)	50(5)	48(4)	-22(4)	7(4)	-3(4)
C(14)	57(7)	40(4)	41(4)	-8(4)	-17(4)	8(5)
C(89)	58(6)	32(4)	43(4)	-5(3)	3(4)	-3(4)
C(74)	20(4)	53(5)	42(4)	-7(4)	0(3)	3(4)
C(86)	30(5)	39(5)	61(5)	-1(4)	5(4)	-10(4)
C(21)	33(5)	43(5)	30(3)	13(3)	0(3)	0(4)
C(23)	30(5)	59(6)	47(5)	-6(4)	3(4)	-13(5)
C(9)	41(5)	63(6)	43(4)	-8(4)	14(4)	-5(5)
C(84)	30(5)	56(5)	50(4)	-6(4)	12(4)	-12(4)
C(17)	43(6)	50(5)	40(4)	-15(4)	-6(4)	2(5)
C(77)	34(5)	46(5)	42(4)	-4(4)	-5(4)	-2(4)
C(96)	29(4)	27(4)	30(3)	-1(3)	2(3)	-12(3)
C(2)	26(5)	73(6)	54(5)	-9(4)	9(4)	-5(5)
C(10)	40(6)	81(7)	51(5)	-22(5)	-19(5)	7(6)
C(3)	67(7)	53(5)	42(4)	10(4)	29(5)	-2(5)
C(97)	34(5)	47(5)	45(4)	-13(4)	-9(4)	2(4)
C(15)	75(8)	51(5)	42(4)	17(4)	-3(5)	4(5)
C(93)	59(6)	74(7)	29(4)	-10(4)	13(4)	-17(6)
C(6)	41(5)	49(5)	48(4)	8(4)	-17(4)	-3(4)
C(24)	55(6)	44(5)	38(4)	9(4)	11(4)	5(5)
C(11)	57(6)	49(5)	36(4)	-17(4)	-11(4)	8(5)
C(95)	35(5)	54(5)	50(4)	-4(4)	-1(4)	6(5)
C(87)	38(5)	29(4)	39(4)	5(3)	-4(4)	-4(4)
C(18)	44(5)	47(5)	29(4)	1(3)	9(4)	9(4)
C(12)	77(8)	46(5)	52(5)	13(4)	-27(5)	-4(5)
C(94)	36(5)	92(8)	52(5)	-16(5)	16(4)	4(6)
C(76)	25(4)	40(4)	30(3)	0(3)	-5(3)	-8(4)
C(85)	54(6)	37(5)	51(5)	-15(4)	6(4)	-3(4)
Li(4)	22(7)	38(7)	29(6)	9(5)	1(5)	-3(6)
C(79)	79(8)	29(4)	74(6)	16(4)	14(6)	8(5)
C(75)	51(6)	30(4)	44(4)	0(3)	-5(4)	-1(4)
N(92)	29(4)	23(3)	38(3)	-3(2)	7(3)	-2(3)
N(93)	26(4)	37(4)	37(3)	-3(3)	11(3)	0(3)
C(67)	36(5)	77(7)	57(5)	1(5)	21(4)	9(5)
C(62)	19(4)	32(4)	49(4)	-5(3)	0(4)	-4(4)
C(91)	52(6)	39(4)	34(4)	-1(3)	1(4)	-16(4)
C(72)	51(6)	92(8)	55(5)	-11(6)	9(5)	13(6)
C(66)	50(6)	41(5)	51(5)	-16(4)	3(4)	7(4)
C(80)	48(6)	55(6)	52(5)	5(4)	-2(5)	25(5)

C(69)	66(7)	36(5)	44(4)	-10(4)	0(4)	-6(5)
C(73)	39(5)	59(5)	34(4)	-1(4)	5(4)	-1(4)
C(83)	67(7)	55(5)	35(4)	3(4)	-6(4)	6(5)
C(61)	39(6)	54(5)	62(5)	-5(4)	-4(5)	12(5)
C(68)	47(6)	50(5)	39(4)	4(4)	-4(4)	-5(5)
Li(7)	38(8)	31(7)	34(6)	2(5)	-3(6)	-9(6)
Li(3)	35(8)	26(6)	29(6)	2(5)	3(6)	2(6)
C(63)	41(6)	61(6)	48(5)	-6(4)	16(4)	6(5)
C(60)	29(4)	28(4)	37(4)	0(3)	4(3)	-7(3)
Li(2)	24(7)	26(6)	39(6)	-2(5)	0(6)	-8(6)
C(64)	58(7)	57(6)	62(5)	19(5)	-14(5)	9(5)
C(71)	43(6)	95(8)	72(6)	-18(6)	1(5)	42(6)
Li(5)	37(8)	31(6)	45(7)	-7(5)	7(7)	-19(7)
C(65)	44(6)	69(6)	67(6)	7(5)	-8(5)	24(5)
C(70)	47(6)	77(7)	63(6)	0(5)	-6(5)	29(5)
C(90)	59(6)	41(5)	48(5)	10(4)	0(4)	0(5)
C(81)	70(8)	98(8)	72(7)	19(6)	-19(6)	39(7)
Li(1)	36(8)	24(6)	31(6)	-7(5)	1(6)	-12(6)
Li(6)	29(8)	50(8)	28(6)	-3(5)	-3(6)	-9(7)
C(82)	98(10)	115(9)	43(5)	3(6)	-19(6)	41(8)
Li(8)	42(8)	16(5)	26(5)	2(4)	1(5)	-13(6)

Table 5. Hydrogen coordinates (x 10^4) and isotropic displacement parameters ($Å^2$x 10^3) for srgr1101.

	x	y	z	U(eq)
H(16A)	4629	-2069	652	65
H(16B)	4148	-2093	1057	65
H(16C)	5169	-1769	1008	65
H(19A)	603	1637	648	50
H(19B)	1308	1837	978	50
H(19C)	326	1487	1074	50
H(88A)	-1879	-2394	346	38
H(4A)	-359	-6988	1882	61
H(4B)	90	-6784	1488	61
H(4C)	-954	-7078	1506	61
H(98A)	-5500	-8208	2423	63
H(98B)	-5057	-8389	2024	63
H(98C)	-6158	-8241	2060	63
H(20A)	403	205	392	58
H(20B)	-41	77	797	58
H(20C)	789	-394	648	58
H(5A)	-1813	-6142	2254	51
H(5B)	-2435	-6360	1902	51
H(5C)	-2360	-5585	2016	51
H(7A)	-4503	-3357	1862	67
H(7B)	-4662	-3498	1426	67
H(7C)	-3734	-3118	1564	67
H(22A)	1463	1300	2169	57
H(22B)	1284	1664	1777	57
H(22C)	2307	1386	1878	57
H(99A)	-7990	-6840	1361	70
H(99B)	-7487	-7558	1350	70
H(99C)	-7202	-6992	1053	70
H(78A)	-1489	-8559	1071	75
H(78B)	-2264	-8126	1287	75
H(78C)	-1816	-7849	907	75
H(92A)	-5853	-6490	2227	31
H(8A)	-2895	-4051	2270	55
H(8B)	-2270	-3720	1947	55

H(8C)	-2186	-4507	2033	55
H(13A)	5985	-533	1760	71
H(13B)	5856	-329	1330	71
H(13C)	5845	-1104	1449	71
H(1A)	-943	-6312	335	64
H(1B)	-1816	-6389	614	64
H(1C)	-856	-6778	698	64
H(14A)	4469	107	2133	69
H(14B)	3543	294	1902	69
H(14C)	4547	553	1761	69
H(89A)	-351	-3041	29	67
H(89B)	-926	-3290	387	67
H(89C)	192	-3220	408	67
H(74A)	-4292	-8045	-207	58
H(74B)	-4964	-7998	151	58
H(74C)	-4268	-8624	104	58
H(86A)	-2975	-1808	1121	65
H(86B)	-2257	-2280	1343	65
H(86C)	-2511	-2442	916	65
H(21A)	2103	869	260	53
H(21B)	2730	330	478	53
H(21C)	2807	1110	581	53
H(23A)	-241	460	1932	68
H(23B)	-195	8	1562	68
H(23C)	-241	809	1529	68
H(9A)	-4671	-4678	2136	73
H(9B)	-4090	-5306	1978	73
H(9C)	-4935	-4997	1738	73
H(84A)	390	-1997	19	68
H(84B)	907	-2107	412	68
H(84C)	251	-1464	350	68
H(17A)	3153	-1348	263	67
H(17B)	2617	-730	454	67
H(17C)	2531	-1466	630	67
H(77A)	-2988	-9027	325	49
H(96A)	-7236	-7419	1972	34
H(2A)	763	-6167	1017	77
H(2B)	866	-5375	1081	77
H(2C)	901	-5676	666	77
H(10A)	-5294	-4128	968	86

H(10B)	-5243	-4922	1037	86
H(10C)	-5299	-4634	620	86
H(3A)	-696	-4972	311	81
H(3B)	-414	-4503	657	81
H(3C)	-1491	-4695	587	81
H(97A)	-4417	-7313	2501	63
H(97B)	-4309	-6749	2185	63
H(97C)	-3951	-7498	2107	63
H(15A)	4349	-1281	2140	84
H(15B)	4218	-1745	1778	84
H(15C)	3348	-1325	1939	84
H(93A)	-7382	-6760	3022	65
H(93B)	-6943	-6171	2771	65
H(6A)	11	-5614	2210	69
H(6B)	-271	-4952	1983	69
H(6C)	537	-5435	1827	69
H(24A)	1490	-28	2290	69
H(24B)	2332	-268	2025	69
H(24C)	1315	-601	1984	69
H(11A)	-3623	-5063	256	71
H(11B)	-3773	-5614	577	71
H(11C)	-2757	-5291	511	71
H(95A)	-8277	-6509	1941	56
H(95B)	-7487	-6019	2105	56
H(87A)	-1198	-1090	533	43
H(18A)	4984	-749	279	60
H(18B)	5424	-420	649	60
H(18C)	4563	-62	441	60
H(12A)	-3619	-3718	354	88
H(12B)	-2710	-3657	615	88
H(12C)	-3684	-3332	746	88
H(94A)	-8516	-6356	2596	72
H(94B)	-8289	-7131	2512	72
H(76A)	-2209	-7720	303	38
H(85A)	-2116	-805	1207	71
H(85B)	-1006	-786	1139	71
H(85C)	-1428	-1216	1478	71
H(79A)	-2448	-9519	1017	91
H(79B)	-3511	-9451	877	91
H(79C)	-3204	-9135	1270	91

H(75A)	-3585	-7022	-115	62
H(75B)	-3146	-6819	281	62
H(75C)	-4261	-6873	233	62
H(67A)	3521	-3589	1404	85
H(67B)	3204	-2861	1545	85
H(67C)	2783	-3160	1165	85
H(62A)	2965	-3363	2096	40
H(91A)	-5866	-7086	2797	50
H(91B)	-6653	-7587	2640	50
H(72A)	-1266	-8465	-527	79
H(72B)	-966	-8078	-152	79
H(66A)	2433	-4475	1381	71
H(66B)	1670	-4020	1172	71
H(66C)	1406	-4388	1556	71
H(80A)	-3123	-1504	505	62
H(80B)	-2773	-771	625	62
H(69A)	1711	-2499	2647	73
H(69B)	1266	-2067	2313	73
H(69C)	2256	-2433	2257	73
H(73A)	-2847	-8472	-331	53
H(73B)	-2468	-7715	-347	53
H(83A)	-960	-1469	-142	63
H(83B)	-1302	-2206	-258	63
H(61A)	2638	-4765	1982	62
H(61B)	3603	-4386	1892	62
H(68A)	2473	-3450	2769	55
H(68B)	1503	-3819	2865	55
H(63A)	305	-3101	2687	76
H(63B)	-76	-3540	2344	76
H(63C)	-164	-2739	2334	76
H(60A)	1276	-4072	2224	37
H(64A)	2793	-4544	2983	71
H(64B)	2124	-4857	2668	71
H(71A)	-667	-9238	-89	84
H(71B)	-1749	-9400	-172	84
H(65A)	3682	-4964	2473	72
H(65B)	3853	-4174	2528	72
H(70A)	-1065	-8691	469	75
H(70B)	-1496	-9433	476	75
H(90A)	-7063	-5859	1490	74

H(90B)	-6176	-6075	1243	74
H(90C)	-6052	-5935	1681	74
H(81A)	-2213	-566	18	96
H(81B)	-3298	-779	-6	96
H(82A)	-2872	-1898	-168	102
H(82B)	-2392	-1393	-460	102
